# Discovery of 4‐Quinazolinone‐Containing Phenylalanine Derivatives as Potent, Resistant‐Tolerant HIV Capsid Inhibitors

**DOI:** 10.1002/mco2.70746

**Published:** 2026-05-03

**Authors:** Xujie Zhang, Lin Sun, Laura Walsham, Yuexi Ma, Dang Ding, Mei Wang, Fabao Zhao, Jian Zhang, Zhao Wang, Shujing Xu, Xiangyi Jiang, Yang Zhou, Erik De Clercq, Christophe Pannecouque, Chin‐Ho Chen, David C. Goldstone, Xinyong Liu, Alexej Dick, Peng Zhan

**Affiliations:** ^1^ Department of Medicinal Chemistry School of Pharmaceutical Sciences Key Laboratory of Chemical Biology (Ministry of Education) Shandong University Jinan Shandong China; ^2^ School of Biological Sciences University of Auckland Auckland New Zealand; ^3^ Department of Microbiology and Infectious Disease Research Center School of Basic Medical Sciences Peking University Health Science Center Beijing China; ^4^ Rega Institute for Medical Research Laboratory of Virology and Chemotherapy K.U. Leuven Leuven Belgium; ^5^ Surgical Oncology Research Facility Duke University Medical Center Durham North Carolina USA; ^6^ Department of Biochemistry & Molecular Biology Drexel University College of Medicine Philadelphia Pennsylvania USA

**Keywords:** 4‐quinazolinone, drug design, HIV‐1 capsid, long‐acting drugs, phenylalanine derivatives

## Abstract

The HIV‐1 capsid (CA) is a validated antiviral target that plays critical roles in both the early and late stages of the viral life cycle. Using structure‐based strategy, we designed and synthesized a series of phenylalanine derivatives containing a 4‐quinazolinone scaffold as novel HIV‐1 CA inhibitors. Among them, **IC‐2i** exhibited potent antiviral activity in MT‐4 cells against HIV‐1 NL4‐3 (EC_50_ = 0.65 ± 0.27 nM) and effectively protected cells from HIV‐1 IIIB infection. SPR revealed that **IC‐2i** interacts strongly with CA hexamers (*K*_D_ = 2.7 ± 0.5 nM) with an extended residence time and competes with host factors CPSF6 and NUP153, disrupting CA assembly and disassembly. **IC‐2i** retained activity against lenacapavir (LEN)‐resistant strains, such as N74D (11‐fold shift vs. 20‐fold for LEN). Crystallographic analysis revealed that **IC‐2i** binds at the CA NTD–CTD interface and forms hydrogen bonds with Thr107 and Ser41 (NTD–NTD interface). Pharmacological evaluation demonstrated favorable properties, including good plasma stability, low toxicity (SI > 1571), and suitable pharmacokinetics with a prolonged half‐life following subcutaneous administration (*T*_1/2_ = 19.9 h). Overall, this study identifies 4‐quinazolinone‐based phenylalanine derivatives as promising HIV‐1 CA inhibitors and highlights **IC‐2i** as a potential long‐acting therapeutic candidate for HIV‐1 treatment.

## Graphical abstract

Novel phenylalanine derivatives were identified as HIV‐1 capsid inhibitors.

IC‐2i showed significant anti‐HIV‐1 activity at picomolar concentrations.

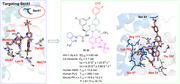

## 1 Introduction

Acquired immunodeficiency syndrome (AIDS) represents one of the most severe global health threats to human populations, mainly caused by human immunodeficiency virus type 1 (HIV‐1) [1]. HIV‐1 mainly infects CD4^+^ T lymphocytes, mononuclear macrophages, and dendritic cells, leading to functional defects in the human immune system, which increases the likelihood of tumorigenesis and opportunistic infections [2]. Since the end of 2022, approximately 39 million individuals worldwide have been living with HIV, with 1.3 million new infections and 630,000 AIDS‐related deaths reported [3, 4]. Despite the use of combination antiretroviral therapy (cART), the virus cannot be eradicated entirely due to a heterogeneous and tissue‐specific latent viral reservoir pool of HIV [5]. As a result, infected individuals must remain on medication for the remainder of their lives, which can lead to significant resistance to existing drugs [6, 7]. In addition, the use of cART can cause harmful and severe side effects, which cannot be ignored [8, 9, 10]. For these reasons, research on the development of novel, resistance‐tolerant anti‐HIV agents with innovative mechanisms and low toxicity are urgently needed.

The HIV‐1 capsid (CA) protein, an essential structural protein, facilitates many functions throughout the viral life cycle [11, 12]. Acting as a protective shield for the viral genome and critical enzymes, it regulates the uncoating [13, 14], reverse transcription [15], nuclear import [16, 17, 18], integration [19, 20], assembly of virus [21, 22], and maturation [22, 23, 24] with reacting with several host proteins, including cyclophilin A (CYPA) [25, 26], nucleoporin 153 (NUP153) [27], nucleoporin 358 [28], cleavage and polyadenylation‐specific factor 6 (CPSF6) [29], and Sec24C. Given the CA's versatility, it is indispensable for the virus to complete its life cycle [22, 30].

**FIGURE 1:**
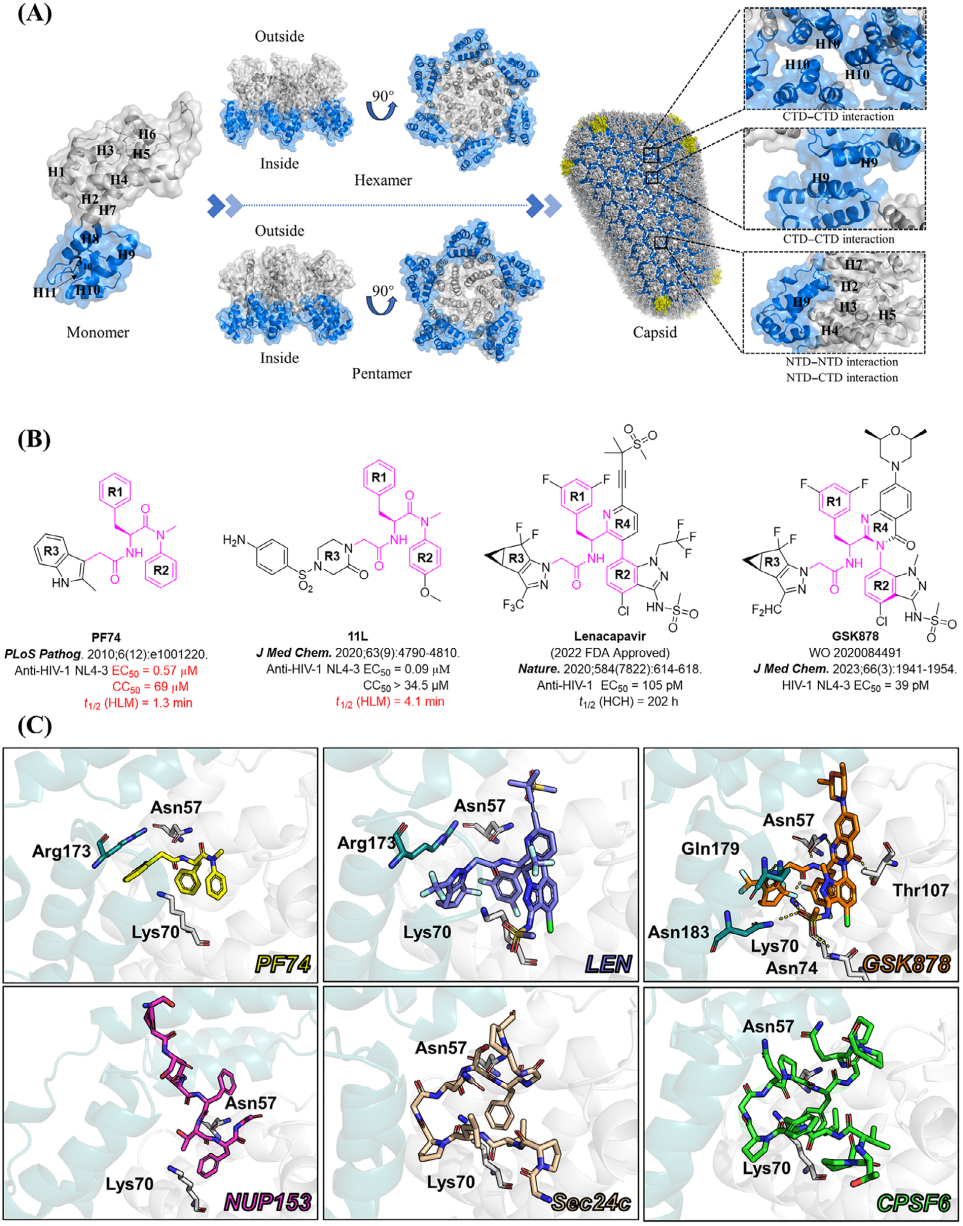
(A) Structure of HIV‐1 CA protein in monomeric (PDB ID: 4XFX) and oligomeric form (hexamer PDB ID: 4XFX, pentamer PDB ID: 3P05, the intact capsid PDB ID: 3J3Q). The NTD is shown in gray, and CTD is shown in blue. CA pentamers are shown in yellow to distinguish them from hexameric CA structures. (B) Representative HIV‐1 CA inhibitors. (C) Binding modes of representative HIV‐1 CA inhibitors and host factors within the NTD–CTD interface of HIV‐1 CA. (PF74 PDB ID: 4XFZ; LEN PDB ID: 6VKV; GSK878 PDB ID: 8FIU; NUP153 PDB ID: 6AYA; Sec24c PDB ID: 6PU1; CPSF6 PDB ID: 4WYM) The figures were generated in PyMOL 2.3.0 (www.pymol.org).

The intact mature HIV‐1 CA comprises about 1500 CA monomers assembled into 12 pentamers and ∼250 hexamers. CA monomers are composed of an *N*‐terminal domain (NTD; 1–146 aa), a *C*‐terminal domain (CTD; 151–231 aa), and a flexible linker between the two domains (Figure 1A). CA–NTD contains a CYPA binding loop, a *β*‐hairpin, and seven *α*‐helixes (H1–H7); CA–CTD contains a 3_10_ helix and 4 *α*‐helixes (H8–H11) [12, 30]. The stability of the CA hexamer relies on both NTD–CTD interactions and NTD–NTD interactions between monomers, with the NTD–CTD interactions within each monomer being critical for maintaining a native structure. In the mature CA lattice, CTD is the primary stabilizing domain between hexamers [31, 32, 33].

The small molecule inhibitor **PF74** (Figure 1B) effectively binds to the NTD–CTD interface, exhibiting inhibitory activity against various strains of HIV‐1 with EC_50_ values ranging from 0.113 to 0.362 µM [34]. **PF74** demonstrates a dual mechanism of action, displaying unique effects at different concentrations [35]. It competes with multiple host factors, including CPSF6, NUP153, and Sec24C, which share a similar binding site (Figure 1C) [29, 36, 37]. This distinct binding profile enables **PF74** to exert its multistage and bimodal mechanism of action.

Despite its unique mechanism, the antiviral potency limitations and unstable metabolism of **PF74** have hindered its progression into clinical research. In contrast, lenacapavir (**LEN**; Figure 1B), which is derived from multiple iterative modifications of **PF74**, has demonstrated remarkable anti‐HIV activity at the picomolar level and exhibits good metabolic stability [38, 39, 40]. Consequently, **LEN** has become the first United States Food and Drug Administration‐approved HIV‐1 CA inhibitor. However, in clinical trials and in vitro screenings, multiple resistant strains (L56I, M66I, K70R, Q67H/N74S, and Q67H/T107N) have emerged that render them insensitive to **LEN [** 41, 42]. Furthermore, the complex synthesis process and high cost associated with **LEN** present limitations to its widespread clinical application [43]. As a result, current research and development efforts are geared toward discovering efficient, drug‐resistant, and cost‐effective HIV‐1 CA inhibitors [44].

## 2 Results

### 2.1 Molecular Design

Gillis et al. from ViiV Health conducted a study replacing the R4 ring of **LEN** with a 4‐quinazolinone ring, leading to the discovery of **GSK878** (Figure 1B) [45, 46]. This compound exhibited potent antiviral activity, with an EC_50_ of 39 pM, and demonstrated long‐lasting pharmacokinetic properties in rats. However, the metabolite derived from the cis‐2,6‐dimethylmorpholine ring of this compound was found to cause significant liver damage, which led to the cessation of its further development. Despite this setback, introducing the 4‐quinazolinone ring in **GSK878** is a promising foundation for modifying novel HIV CA inhibitors. An interesting feature of **GSK878** is the newly introduced carbonyl group on the 4‐quinazolinone ring, which forms a hydrogen bond with Thr107 (Figure 1C). This interaction is unprecedented in previous HIV‐1 CA inhibitors and may contribute to the enhanced antiviral activity displayed by this compound.

**FIGURE 2:**
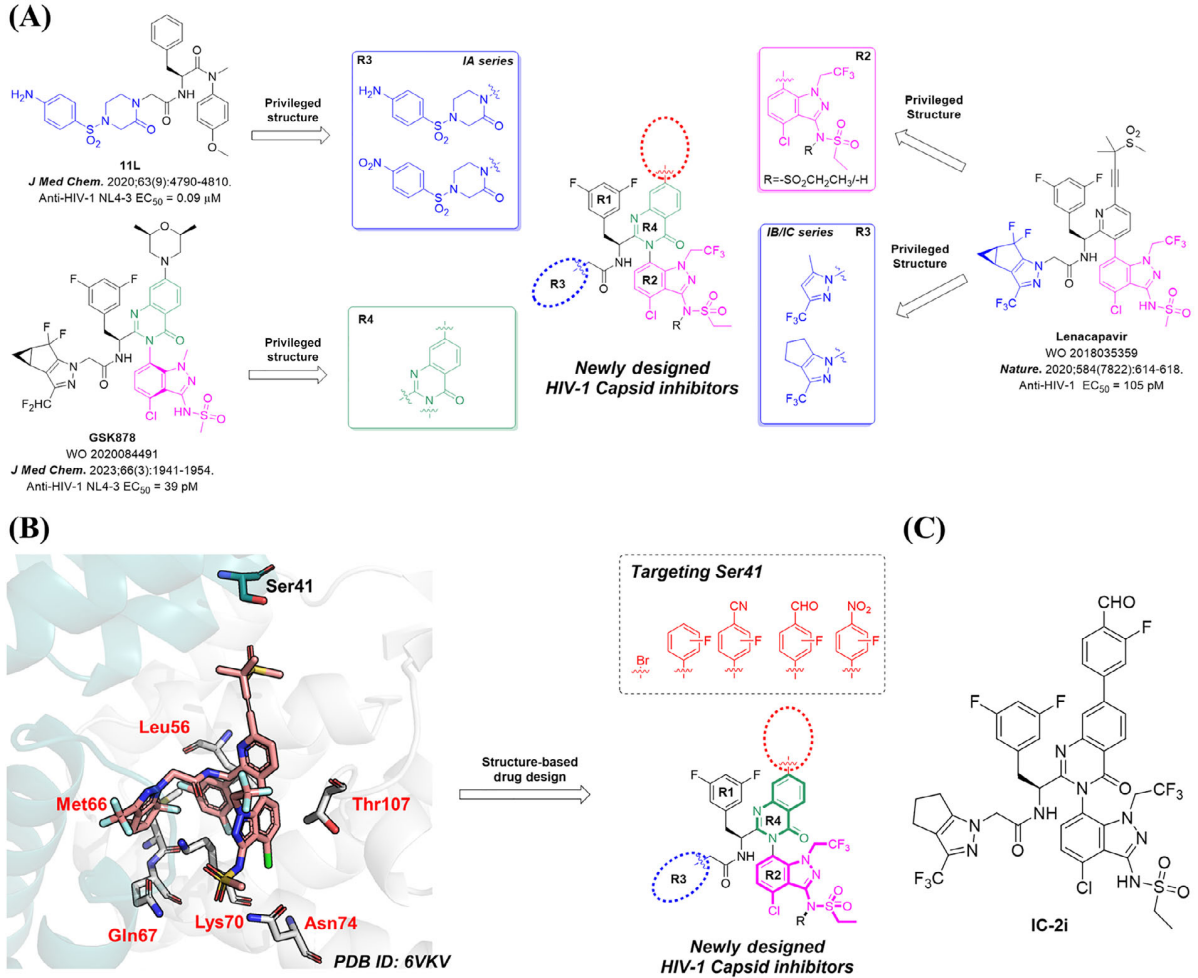
(A) Design strategy for novel HIV‐1 CA modulators with the potential to overcome clinically observed resistant mutations; (B) design strategies, including examples of functional groups (dotted box) to target a conserved Ser41 within the HIV‐1 CA NTD–CTD interface. The figure was generated in PyMOL 2.3.0 (www.pymol.org). (C) Structure of representative compound **IC‐2i**.

In 2020, our research group presented a series of HIV‐1 CA inhibitors based on phenylalanine, incorporating a benzene sulfonyl group piperazinone side chain, with **PF74** as the lead compound (Figure 1B) [47]. These inhibitors belong to a peptide‐like compound category, which means that the 2‐(2‐oxo‐4‐(phenylsulfonyl)piperazin‐1‐yl)acetyl of these inhibitors bears a resemblance to the main chain structure of several host factors, including NUP153, CPSF6, and Sec24c [29, 36, 37, 48], allowing for a flexible conformation that can better adapt to the dynamic structure of CA. However, both representative compounds **11L** and **PF74** exhibited a common issue, poor metabolic stability. To address this problem, we introduced the indazole ring from **LEN** and the 4‐quinazolinone ring from **GSK878**, incorporating the 2‐(2‐oxo‐4‐(phenylsulfonyl)piperazin‐1‐yl)acetyl of **11L** into the newly designed compounds. This led to the development of a new series of compounds, referred to as the IA series (Figure 2A), aiming to retain the flexible conformation of the original compound while improving metabolic stability. However, during the synthesis of this series, we observed that almost all target compounds exhibited low solubility in solvents like water and DMSO. This phenomenon may be attributed to excessive hydrogen bonding between the compound's donor and acceptor, leading us to abandon further modifications in this direction.

Therefore, regarding the choice of the R3 ring in the subsequent series, we decided to move away from the benzene–sulfonyl–piperazinone structure and instead drew inspiration from the structure of **LEN**. However, the Q67H mutation introduces an allosteric site in the binding pocket [49], where the mutated His67 can adopt two conformations: open and closed. In the closed conformation, the left‐wing structure of **LEN** clashes spatially with His67, resulting in decreased affinity between **LEN** and CA [49]. To overcome this challenge, we aimed to enhance the flexibility of the functional group at this position, enabling the inhibitor to adopt a more flexible conformation and reduce the impact of the Q67H mutation. Motivated by this insight, we narrowed down the R3 ring by eliminating the conflicting aliphatic ring at the end of the left‐wing while retaining a single methyl group to maintain its original hydrophobic effect. We removed some fluorine atoms with large atomic volumes to minimize the size of the R3 ring. As a result, we obtained 3‐methyl‐5‐trifluoromethylpyrazole as the side chain for the IB series (Figure 2). Additionally, to further enhance the hydrophobic effect of the R3 ring system, we eliminated the gem‐difluoro structure of cyclopropane and cyclopentane from the R3 ring of **LEN**, yielding 3‐(trifluoromethyl)‐1,4,5,6‐tetrahydrocyclopentane[*c*]pyrazole as the side chain for the IC series (Figure 2A).

Through our investigation of drug‐resistant mutations, we observed that the amino acids affected by these mutations are all located within the binding pocket of the NTD–CTD interface, specifically in the NTD (Figure 2B) [41, 49, 50, 51]. Notably, no drug‐resistant mutations were identified in the NTD–NTD interface region, which LEN initially explored. Based on this finding, we aimed to design novel CA inhibitors targeting this conserved region to minimize the impact of drug‐resistant mutations on the antiviral activity of the newly developed compounds. Further examination of the NTD–NTD interface revealed that **LEN** extends toward the adjacent subunit NTD's H4 helix, establishing hydrogen bonds with Ser41, which we sought to enhance the interaction between the new compounds with. To achieve this, we introduced a bromine atom with a large atomic volume at the 7‐position of the 4‐quinazolinone. Additionally, we introduced a benzene ring at the 7‐position and incorporated functional groups such as aldehyde, cyano, nitro, and fluorine atoms onto the benzene ring. These groups can form hydrogen or halogen bonds with Ser41 (Figure 2B).

### 2.2 Chemical Syntheses

The new compounds were synthesized using a modular synthesis strategy, where each compound component was synthesized separately as intermediate modules and then assembled into the final target compound.

**FIGURE 3:**
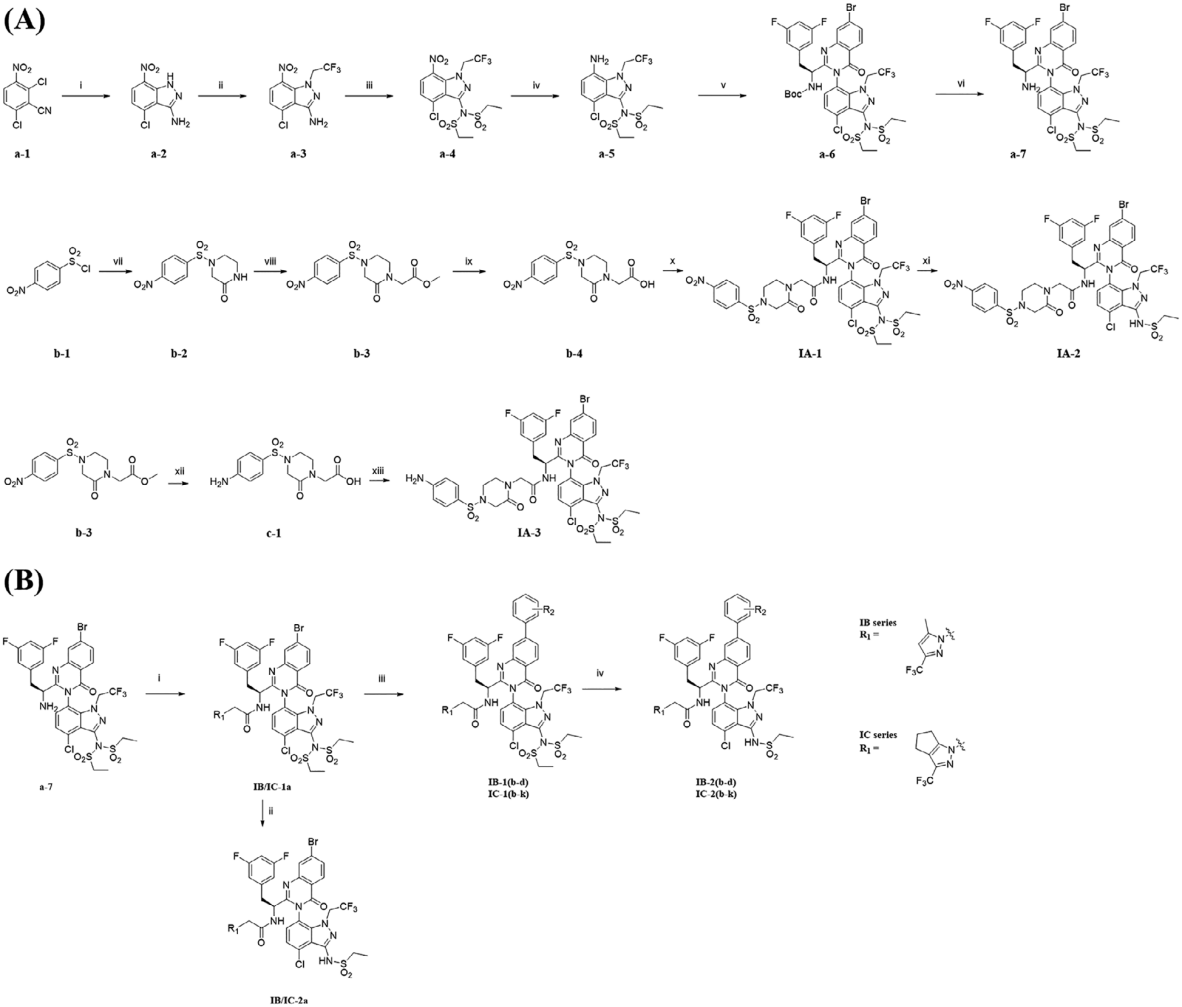
(A) Synthesis scheme for series IA: (i) NH_2_NH_2_·H_2_O, EtOH, r.t.; (ii) CF_3_CH_2_OTf, Cs_2_CO_3_, DMF, 0°C to r.t.; (iii) CH_3_CH_2_SO_2_Cl, DMAP, Et_3_N, DCM, 0°C; (iv) Zn, NH_4_Cl, THF, H_2_O, r.t.; (v) Boc‐3,5‐difluoro‐*L*‐phenylalanine, diphenyl phosphite, 2‐amino‐4‐bromobenzoic acid, pyridine, 75°C; (vi) TFA, DCM, 0°C to r.t.; (vii) piperazin‐2‐one, Et_3_N, DCM, 0°C to r.t.; (viii) methyl bromoacetate, NaH, THF, 0°C to r.t.; (ix) LiOH, THF, H_2_O, r.t.; (x) intermediate a‐7, HATU, DIEA, DCM, 0°C to r.t.; (xi) K_2_CO_3_, DMF, H_2_O, 75°C; (xii) Zn, HCl, THF, H_2_O, 75°C; (xiii) intermediate a‐7, HATU, DIEA, DCM, 0°C to r.t.; (B) synthesis scheme for series IB and IC: (i) 2‐[5‐methyl‐3‐(trifluoromethyl)pyrazol‐1‐yl]acetic acid or 2‐(3‐(trifluoromethyl)‐5,6‐dihydrocyclopenta[*c*]pyrazol‐1(4*H*)‐yl)acetic acid, HATU, DIEA, DCM, 0°C to r.t.; (ii) K_2_CO_3_, DMF, H_2_O, 75°C; (iii) different substituted phenylboric acid or pinacol ester of phenylboric acid, Pd(PPh_3_)_4_, K_3_PO_4_, toluene, H_2_O, 95°C; (iv) K_2_CO_3_, DMF, H_2_O, 75°C.

The synthetic route of the IA series is illustrated in Figure 3A. Initially, the target compounds’ three components (**a‐7**, **b‐4**, and **c‐1**) were synthesized through a series of multistep reactions. Finally, the target compounds were obtained through amide condensation. The synthesis of **a‐7**, one of the key intermediates, was based on 2,6‐dichloro‐3‐nitrophenylhydrazine (**a‐1**) and underwent several steps to obtain the final product. First, **a‐1** was used in a ring closure reaction with 50% hydrazine hydrate in ethanol to obtain intermediate **a‐2**. Subsequently, nucleophilic substitution with 2,2,2‐trifluoroethyl trifluoromethanesulfonate in *N*,*N*‐dimethylformamide afforded intermediate **a‐3**. In dichloromethane, intermediate **a‐3** was then reacted with ethanesulfonyl chloride to obtain intermediate **a‐4**. Intermediate **a‐5** was obtained in the presence of zinc powder and ammonium chloride. Through Niementowski 4‐quinazolone synthesis [45, 52], **a‐5**, 2‐amino‐4‐bromobenzoic acid, and *N*‐Boc‐*L*‐3,5‐difluorophenylalanine were condensed to obtain intermediate **a‐6**, and the Boc group was removed in dichloromethane to obtain the key intermediate **a‐7**. The synthesis of intermediate **b‐4** began with the reaction of 4‐nitrobenzenesulfonyl chloride (**b‐1**) with piperazin‐2‐one in dichloromethane, forming intermediate **b‐2**. Subsequently, intermediate **b‐2** was reacted with 2‐bromoacetic acid in tetrahydrofuran to afford intermediate **b‐3**. The ester group of intermediate **b‐3** was then hydrolyzed to get the intermediate **b‐4**, which was subsequently subjected to amide condensation reaction with intermediate **a‐7** to produce the target compound **IA‐1**. Finally, the hydrolysis of **IA‐1** with potassium carbonate resulted in the formation of the target compound **IA‐2**. The synthesis of intermediate **c‐1** was initiated from **b‐3**. The nitro group of **b‐3** was reduced to the amino group via zinc powder, and the ester group was simultaneously cleaved to generate intermediate **c‐1**. Intermediate **c‐1** then underwent amide condensation reaction with module **a‐7** to form the target compound **IA‐3**.

The synthetic route for the IB and IC series is presented in Figure 3B. Starting from intermediate **a‐7**, **IB‐1a** and **IC‐1a** were obtained via an amide condensation reaction. The remaining target compounds, **IB‐1(b–d)** and **IC‐1(b–k)**, were then synthesized via Suzuki reaction. Finally, under the action of potassium carbonate, **IB‐1(a–d)** and **IC‐1(a–k)** were hydrolyzed to produce the target compounds **IB‐2(a–d)** and **IC‐2(a–k)**.

### 2.3 Anti‐HIV Activity of Target Compounds

HIV‐1 NL4‐3 is a well‐known HIV‐1 subtype B molecular clone, commonly used in research to study viral replication [53]. To evaluate the newly synthesized compounds' in vitro antiviral activity and cytotoxicity, MT‐4 cells, which are are routinely employed in antiviral screening assays due to their high sensitivity to HIV‐1‐induced cytopathic effects, infected with HIV‐1 NL4‐3 strain were treated with the compounds and compared with positive controls, including **PF74**, **11L**, and **LEN**. The antiviral activity and cytotoxicity of the compounds were measured using EC_50_ and CC_50_, respectively, while the selective index (SI) was calculated to determine their safety profile.

Table S1 presents the results of testing the antiviral activity and cytotoxicity of the IA series, which comprises three synthesized compounds. Although **IA‐1** showed comparable antiviral activity to **PF74** in vitro, the other two compounds were discontinued due to their high toxicity. Despite the inclusion of a side chain of **11L**, the IA series lost the strong antiviral activity (EC_50_ = 105.23 ± 25.88 nM) and high selectivity (SI > 383.36, CC_50_ derived from Ref. [47]) of **11L**. The reduced antiviral activity of **IA‐1** may be due to its large molecular weight (1137.32 Da), numerous hydrogen bond donors or acceptors, and poor solubility. These factors likely impaired its membrane permeability, as indicated by ADMETlab 2.0 [54], which predicts a Caco‐2 permeability score of −5.318 (ideal value > −5.15). The high cytotoxicity of the IA series may be due to the presence of multiple toxicity warning groups in the molecules, including nitrobenzene, sulfonyl, and aromatic ammonia.

Table S2 displays the outcomes of the antiviral activity and cytotoxicity evaluations conducted on the IB series, which comprised eight specific compounds. All the compounds demonstrated anti‐HIV‐1 activity at submicromolar levels in cell‐based tests. Notably, the most potent compound, **IB‐2d**, had an EC_50_ value of 22.05 nM, surpassing that of **PF74** and **11L**, albeit inferior to **LEN**. Most compounds showed no cytotoxicity at the maximum test concentration of 1000 ng/mL. The R_1_ group was identified to play a crucial role in the antiviral activity of this series of compounds, as replacing it with an ethyl sulfonyl significantly decreased the activity of all compounds (**IB‐1a**, **IB‐1b**, **IB‐1c**, and **IB‐1d**) when compared with the unsubstituted compounds (**IB‐2a**, **IB‐2b**, **IB‐2c**, and **IB‐2d**).

All four compounds with an unsubstituted R_1_ exhibited strong antiviral activity, with EC_50_ values ranging between 20 and 40 nM. However, when R_1_ was substituted, introducing a large group at the 7‐position of the 4‐quinolinone ring enhanced its antiviral activity. Among the compounds tested, those with phenyl substitution (**IB‐2b**, **IB‐2c**, and **IB‐2d**) showed better antiviral activity than those with halogen substitution (**IB‐2a**). The compound with nitro substitution at R_3_ displayed the highest antiviral activity, while for R_3_ cyano‐substituted compounds, ortho monofluoro substitution was superior to difluoro substitution, although the difference was not significant (1.1 times).

The antiviral activity and cytotoxicity of the IC series are also shown in Table S2, revealing promising anti‐HIV‐1 activity (0.65–108.35 nM) for all new compounds tested, with **IC‐2i** emerging as the most potent compound (EC_50_ = 0.65 ± 0.27 nM). Most compounds showed acceptable safety profiles without inducing significant cytotoxicity at the highest test concentration (1000 ng/mL). Notably, **IC‐2a**, **IC‐2i** (Figure 2C), and **IC‐2k** exhibited the best safety profile with SI values above 1594.5, 1571.0, and 1067.8, respectively. The substitution of the R_1_ group played a pivotal role in the antiviral activity of the compounds, with unsubstituted compounds demonstrating 1.7–253.5 times higher antiviral activity than the substituted ones, as observed for the IB series.

With the substitution of ethyl sulfonyl for R_1_, the 7‐position bromine substitution in 4‐quinazolinone (**IC‐1a**) demonstrated significant antiviral activity (EC_50_ = 21.40 ± 2.72 nM). Further analysis of the structure–activity relationship (SAR) of the phenyl at the 7‐position revealed that the replacement of R_2_ and R_4_ with fluorine had a substantial impact on the antiviral activity of the compounds. Compounds with only one of R_2_ or R_4_ replaced by a fluorine atom showed the best antiviral activity, while those with no replacement or both R_2_ and R_4_ replaced with fluoride exhibited lower activity (**IC‐1h **> **IC‐1b **> **IC‐1f**, **IC‐1d **> **IC‐1c **> **IC‐1e**, **IC‐1k **> **IC‐1j**). Moreover, the replacement of R_3_ also significantly affected antiviral activity. When R_2_ and R_4_ were either not replaced or only one of them was replaced with a fluorine atom, the aldehyde was the best replacement for activity, while the nitro replacement exhibited the worst activity (**IC‐1i**> **IC‐1d**> **IC‐1h**> **IC‐1k**, **IC‐1c**> **IC‐1b**> **IC‐1j**). Additionally, in compounds where fluorine atoms replaced R_2_ and R_4_, the order of activity for different substitutions of R_3_ was cyan > fluorine > unsubstituted (**IC‐1e **> **IC‐1g **>** IC‐1f**).

When R_1_ was not substituted by ethyl sulfonyl, significant differences were observed in the activity of the compounds. Specifically, the compound (**IC‐2a**) in which the 7‐position of 4‐quinazolinone was substituted by bromine showed an EC_50_ value that was 31.9 times higher than that of the unmodified compound (**IC‐1a**), reaching 0.67 nM. Notably, the SAR of phenyl in the 7‐position was maintained. When R_2_ or R_4_ was substituted by fluorine, compounds were found to be more active. The compounds with neither R_2_ nor R_4_ substituted were the second most active, while those with both substituted showed the weakest activity (**IC‐2h **>** IC‐2b **> **IC‐2f**, **IC‐2k **> **IC‐2j**). When R_3_ was substituted by cyan, the most potent compound was the one with unsubstituted R_2_ and R_4_ (**IC‐2c**). Compounds with only one of the R_2_ and R_4_ substitutes were the second most potent, while compounds with both R_2_ and R_4_ substitutes showed the weakest activity (**IC‐2c** > **IC‐2d** > **IC‐2e**). Moreover, the active sequence of different R_3_ substitutions was as follows: aldehyde > nitro > unsubstitution > cyan (**IC‐2i **>** IC‐2k** >** IC‐2h** > **IC‐2d**).

In addition, we carefully selected representative compounds (**IA‐1**, **IB‐1a**, **IB‐2a**, **IB‐1d**, **IB‐2d**, **IC‐1a**, **IC‐2a**, **IC‐1i**, **IC‐2i**, **IC‐1k**, and **IC‐2k**) from each compound series and evaluated their antiviral activity against both HIV‐1 IIIB and HIV‐2 ROD in MT‐4 cells. The results of these tests are summarized in Table S3. Compared with the reference compound **PF74**, most selected compounds demonstrated more potent inhibitory effects against HIV‐1 and HIV‐2. Interestingly, the majority of the compounds exhibited stronger antiviral activity against HIV‐1 when compared with HIV‐2. Furthermore, through progressive optimization of the compound structures, we observed a steady increase in their antiviral activity against both strains, consistent with their inhibitory effect on HIV‐1 NL4‐3. Specifically, we noted a significant increase in activity ranging from 4.3 to 102.7 times for the IB and IC series compounds when the sulfonyl group did not replace the R_1_ group. However, although these representative compounds displayed improved antiviral activity, they still exhibited lower potency compared with **LEN**. Nevertheless, it is noteworthy that the majority of the compounds demonstrated excellent selectivity, with SIs exceeding 1000.

### 2.4 Affinity of Target Compounds With HIV‐1 CA Hexamer and Monomer

We focused on the IB and IC series, known for their potent antiviral activity, to advance our understanding of our novel antiviral agents. We assessed their binding affinity to the HIV‐1 CA hexamer and monomer using surface plasmon resonance (SPR), with **11L**, **PF74**, and **LEN** as positive controls.

**FIGURE 4:**
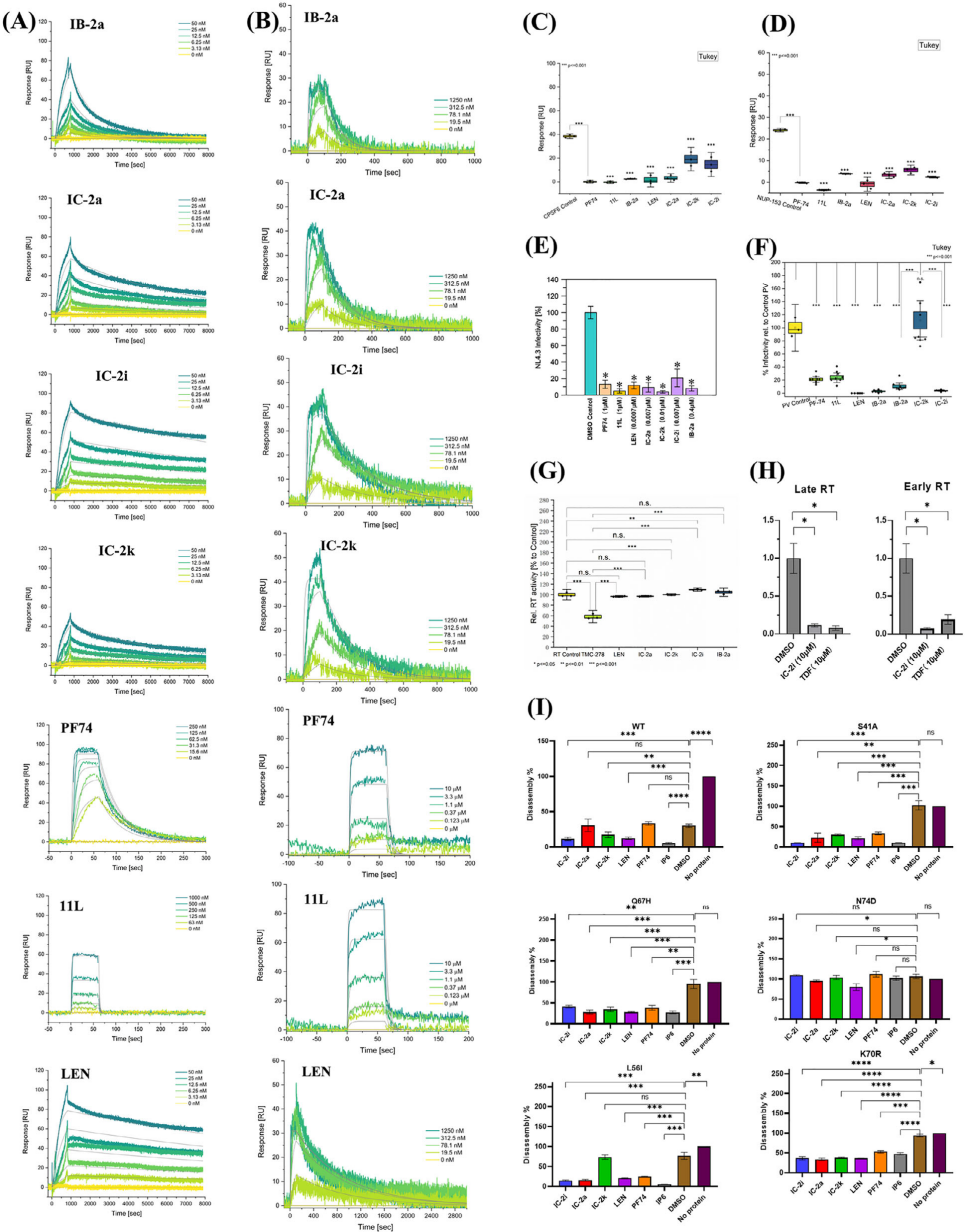
(A) SPR sensorgrams of test compounds binding to hexameric HIV‐1 CA, with **PF74**, **11L**, and **LEN** used as the controls (*n* = 3). (B) SPR sensorgrams of test compounds binding to monomeric HIV‐1 CA, with **PF74**, **11L**, and **LEN** used as the controls (*n* = 3, *n* = 1 for **LEN**). (C) Results of the competition assay with CPSF6 peptide (*n* = 4). (D) Results of the competition assay with NUP153 peptide (*n* = 3). (E) Results of the single‐round infection assay. Infections are an average of 3 replicates with error bars indicating the SEM. (F) Results of the antilate‐stage assay. Infections are an average of 3 replicates with error bars indicating the SEM (*n* = 3); panels (E) and (G) depict single‐dose, single‐cycle readouts to localize early versus late activity; quantitative EC_50_/CC_50_ values for fully infectious virus are reported in Tables S1–S3. (G) Results of anti‐RT assay. The results are an average of 3 replicates with error bars indicating the SEM. (H) Results of anti‐early and anti‐late RT assay. (*n* = 2) (I) Results of in vitro CANC disassembly assays (*n* = 3).

The SPR results for CA binding of representative compounds are shown in Figure 4A,B and Table S5. Notably, except for control **11L**, most compounds demonstrated rapid binding and slow dissociation kinetics with hexameric CA, suggesting a robust and stable interaction with the hexamer. Compound **11L** displayed fast binding and dissociation rates, likely due to structural characteristics such as significant flexibility and numerous rotatable bonds. **PF74** showed the fastest binding and dissociation rate, possibly due to its relatively weak affinity with the CA hexamer (*K*_D_ = 28.9 nM) comparing with the new compounds. Among the test compounds, representative compounds of the I series, including **IB‐2a**, **IC‐2a**, **IC‐2i**, and **IC‐2k**, showed binding and dissociation rates similar to **LEN** and had prolonged dissociation rates, with **IC‐2i** and **LEN** dissociating at the rate of 10^−5^ s^−1^, indicating the formation of a stable complex with the CA hexamer. **IC‐2i** and **LEN** exhibited the strongest affinity for the CA hexamer (*K*_D_ = 2.7 and 0.94 nM, respectively). However, their disparate EC_50_ values in inhibiting HIV‐1 NL4‐3 strains (differing by approximately ninefold) hint at underlying differences in membrane permeability and intracellular stability, factors critical in drug efficacy.

Except for **PF74** and **11L**, most compounds also showed a robust binding affinity with CA monomer. Different from the binding properties of hexamer, all the compounds bound fastly, but they had different dissociation rates, resulting in different affinities. The new compounds and LEN have similar slow dissociation rates. However, **11L** and **PF74** displayed much faster dissociation rates than other compounds, which leads to a low affinity with monomers. However, compared with hexamer binding, most compounds showed a four to seven times lower affinity with monomeric CA, indicating that the interprotomer pocket is critical to establish an extensive interaction network and stabilize compound binding. A sharp decline in affinity by **PF74** further proves this conjecture, which means relying on the indole rather succinct R1–R2 group, **PF74** is not able to maintain most interaction with CA.

We observed an interesting difference in affinity with CA hexamer between **IB‐2a** and **IC‐2a**. This is possibly attributed to structural nuances between those two compounds. In particular, variations in the substituent at the pyrazole ring (a methyl group in **IB‐2a** versus a cyclopentane in **IC‐2a**) led to a nearly fourfold difference in affinity. This suggests that minor structural differences can significantly impact the interaction dynamics with HIV‐1 CA hexamer, likely due to altered hydrophobic interactions. Overall, the SPR results underscore the promising kinetic properties of our new compounds. Like **LEN**, their slow dissociation rates and prolonged residence times reinforce their potential as potent and possibly long‐acting CA inhibitors. These align with current trends in antiviral drug design and development, emphasizing the importance of kinetic properties in the efficacy of antiviral agents.

### 2.5 Effect of Novel HIV‐1 CA Inhibitors on the Early‐Stage of the Viral Replication Cycle

The HIV‐1 CA protein plays a critical role in the early and late stages of the viral replication cycle. The HIV‐1 pNL4‐3‐Luc^+^R^−^E^−^ is a luciferase‐reporter, single‐round HIV‐1 clone derived from the NL4‐3 strain. It lacks the env and vpr genes, which means there is only one cycle of infection [55, 56]. This system provides a safe and quantitative model to evaluate compounds acting on early steps of the HIV‐1 life cycle. Therefore, we selected compounds that illustrated excellent antiviral activity to evaluate in a single‐round infection (SRI) assay their inhibitory effect on the early stage of the HIV‐1 replication cycle.

The results, presented in Figure 4E, offer insights into the efficacy of these compounds against early‐stage HIV‐1 replication. Overall, the SRI assay reveals that all representative compounds can impede HIV‐1 infection in its early stages. This is crucial for understanding the comprehensive antiviral mechanisms of these compounds and guidance toward optimization efforts.

### 2.6 The Effect of Representative Compounds on the Reverse Transcriptase Inhibition in Vitro

To investigate whether the inhibitory activity of the target compounds in the early stage of virus replication was due to the inhibition of reverse transcriptase (RT) activity, we conducted an RT inhibition assay with representative compounds, including **TMC‐278** (rilpivirine) and **LEN** as controls.

The antireverse‐transcriptase activity of the target compounds was shown in Figure 4G. The positive control, a second‐generation RT inhibitor **TMC278**, exhibited strong RT inhibitory activity at a concentration of 0.73 nM. However, none of the tested compounds showed significant inhibitory activity on RT at twice their EC_50_ concentration, indicating that the inhibitory effect of the novel compounds on the early stage of virus replication was not due to RT inhibition. However, in the cellular assays (Figure 4H), both **IC‐2i** and **TDF** (tenofovir disoproxil fumarate) showed inhibition on the early and late RT products. This is not due to direct RT inhibition (Figure 4G: no significant RT inhibitory activity at 2×EC_50_), but rather because **IC‐2i** stabilizes the CA lattice and blocks its normal disassembly—a process required to form the protected microenvironment for efficient RT and nuclear entry of the viral complex.

### 2.7 Impact of Target Compounds on Host Factors Interaction With CA Hexamer

The host factors CPSF6 and NUP153 are crucial in various HIV‐1 CA‐related replication processes, such as facilitating CA transport through the nuclear pore complex, regulating CA disassembly, and mediating the nuclear localization of integration. These functions are essential for the timely and precise formation of the preintegration complex, a critical step in the HIV‐1 lifecycle. Our novel compounds bind within the same binding site as CPSF6 and NUP153. Therefore, we aimed to elucidate whether our compounds could interfere with CPSF6 and NUP153's interaction with the CA hexamer. To achieve this, we employed an SPR‐based competition assay.

Our results, depicted in Figures 4C,D and S1 and Table S5, clearly show the compounds’ impact on CPSF6 and NUP153 displacement. At 200 nM (**11L** and **PF74** at 5 µM), these compounds significantly diminished the binding capacity of two kinds of peptide (at an equivalent concentration) to the CA hexamer. This finding suggests that our compounds might obstruct the binding of the CPSF6 and NUP153 to the CA hexamer during early viral replication. Such an interaction could potentially hinder the CA's entry into the nucleus, disrupt its proper disassembly and localization, and consequently reduce the infection rate in these initial stages.

### 2.8 Influence of Target Compounds on HIV‐1 CANC Disassembly

Timely assembly and disassembly of HIV‐1 CA is critical for the viral replication cycle. Therefore, we focused on the disassembly process of the HIV‐1 CA protein, a key event in the early stages of viral replication. The Capsid Assembly‐Competent Nucleocapsid (CANC) complex, consisting of CA, SP1, and NC, forms a CA tube in vitro under specific ion concentrations [21]. This lattice is composed of hexamers and differs from natural CA in that it lacks CA pentamers, preventing it from “bending” into a fullerene cone. By manipulating ion concentrations, we can induce the disassembly of these CA tubes, simulating the uncoating process of HIV‐1 CA [57, 58]. To study the effect of compounds on the uncoating process in the early stage, we employed fluorescence resonance energy transfer (FRET) to detect the content of single‐stranded DNA (tqON, an oligonucleotide labeled with the reporter dye fluorescein (FAM) as well as Black Hole Quencher (BHQ)) not encapsulated by CA tubes, thereby quantifying the extent of CA tube disassembly [58]. The assembly reaction was initiated by mixing HIV‐1 CA‐NC with tqON in assembly buffer (ASS). The mixture was then incubated to allow tqON incorporation into the particles. Subsequently, compounds were added, followed by a disassembly buffer (DIS) that induces destabilization of preassembled CANC tubes. After incubation, exonuclease I (ExoI) was introduced to digest unbound tqON, while tqON coassembled within the particles was protected from degradation. The degradation of free tqON led to the separation of FAM from BHQ, resulting in detectable fluorescence emission, which quantitatively reflects the effect of the compounds on CANC disassembly (Figure S2).

The insights presented in Figures 4I and S2 reveal varying effects of the compounds on CANC stability and disassembly. Notably, **PF74** and **IC‐2a** did not significantly stabilize the wild‐type CANC, while **IP6** demonstrated a strong stabilizing effect. Among the tested compounds, **IC‐2i** emerged as the most potent inhibitor of CA disassembly, outperforming both **LEN** and **IC‐2k**.

During the clinical utilization of LEN, numerous resistant‐associated mutations (RAMs) evolved that can increase resistance to **LEN** by sixfold to >1000‐fold compared with wild‐type HIV‐1 and, therefore, significantly negatively impact **LEN**s efficacy in the clinic [49]. Indeed, the effects of these compounds varied when tested against different HIV‐1 CA mutants. Despite the loss of self‐assembly ability in most mutant strains, the majority of tested compounds still promoted the assembly of these mutant CANCs in the dissociation buffer. In the case of the S41A mutant, **IC‐2i** showed a stabilization effect comparable to **IP6**, exceeding that of **LEN**, **IC‐2k**, and **PF74**. For the Q67H mutant, **IP6**, **IC‐2a**, and **LEN** demonstrated similar CANC assembly‐promoting effects, but **IC‐2i** was less effective. In contrast, most compounds (**IC‐2a**, **IC‐2i**, **IC‐2k**, **PF74**) failed to promote CANC assembly in the N74D mutant, except LEN. For the L56I mutant, **IC‐2i** and **IC‐2a** promoted CANC assembly, with effects slightly surpassing **LEN** and **PF74**, while **IC‐2k** showed no significant influence. For the K70R mutant, all compounds, barring **PF74** and **IP6**, facilitated similar assembly‐promoting effects.

Our results are critical and demonstrate the superior capability of our compounds to be active against clinically relevant RAMs, at least in the vital HIV‐1 CA disassembly context. While LEN has not been widely used on a large scale, the issue of drug resistance has started to surface. In comparison with other compounds, our compounds preserved the structural integrity of most of RAMs tested, effectively impeding disassembly during the early stage, which significantly emphasizes the potential of novel compounds in combating drug resistance and highlights a promising strategy for the continued development of potent CA inhibitors with strong resistance to RAMs.

### 2.9 Inhibitory Impact of Target Compounds on HIV‐1 Late‐Stage Replication

Compounds targeting HIV‐1 CA generally exhibit inhibitory activity in both the early and late stages of viral replication. To investigate the inhibitory activity of the compounds in the late stage, we tested the infectivity of pseudoviruses treated with representative compounds (during the late stage of pseudovirus production).

As shown in Figure 4F, the experimental results indicated that most representative compounds exhibited excellent inhibitory effects in the late stage at the tested concentration. Notably, **IC‐2k** appears to be an exception, as it lacks significant inhibitory activity in the late stage, indicating that the multicycle antiviral activity of **IC‐2k** relied solely on its inhibitory effect on early viral replication. Furthermore, regarding the early inhibition assay, **IC‐2i** and **IC‐2a** demonstrated lower early inhibition activity than late inhibition, indicating their potential inclination toward exerting their effects in the late stage of virus replication.

### 2.10 Impact of Target Compounds on HIV‐1 CA Assembly in Vitro

Next, we investigated the influence of target compounds on the assembly of the HIV‐1 CA in vitro, a critical process in the late stage of viral replication.

**FIGURE 5:**
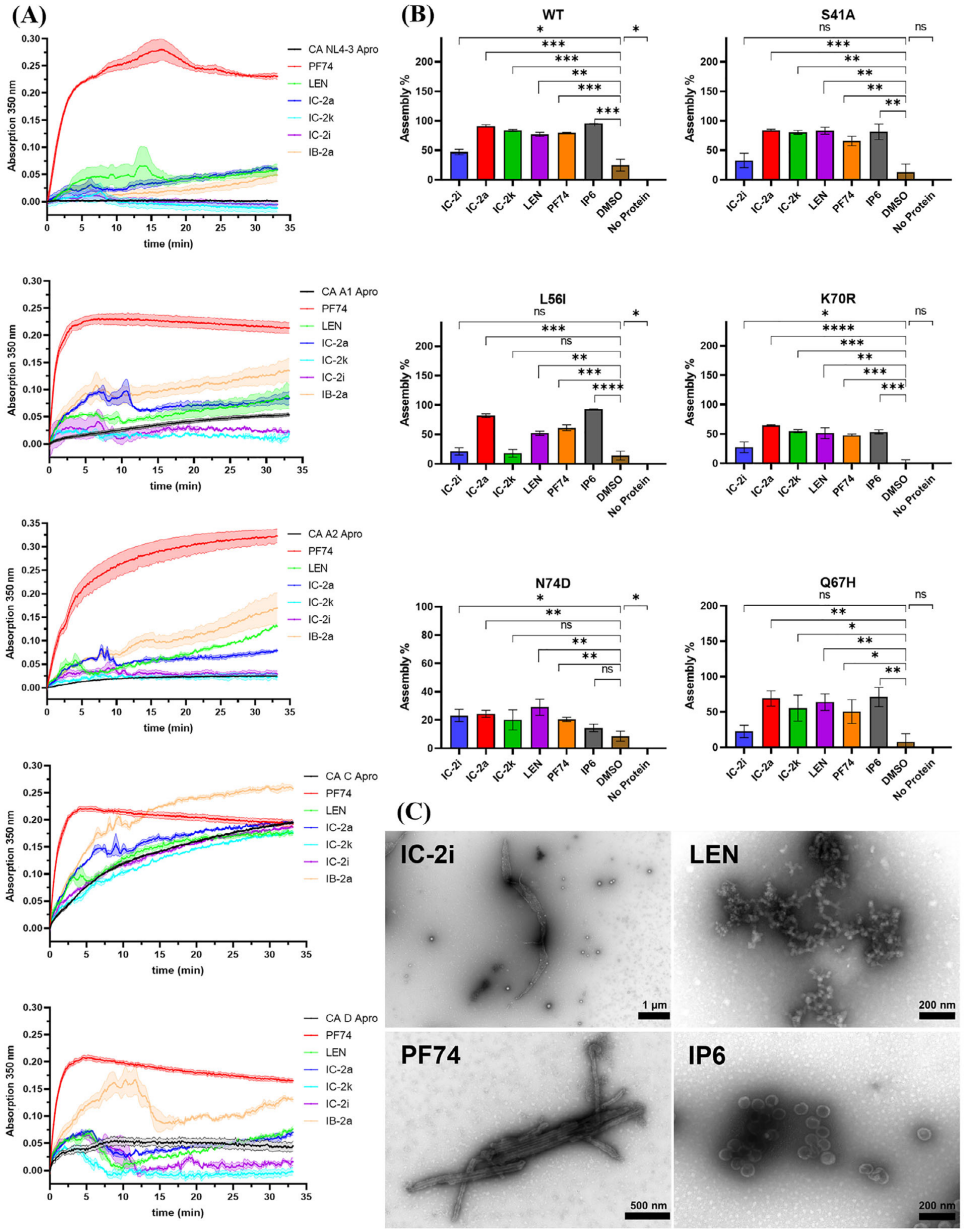
(A) Results of in vitro CA assembly assay (*n* = 3). (B) Results of in vitro CANC assembly assay (*n* = 3). (C) Representative TEM micrographs of compound‐induced (IC‐2i, LEN, PF74, and IP6) CANC assembly products.

Initially, we examined the effect of representative compounds on the assembly of CA using the HIV‐1 NL4‐3 strain, a subtype B virus. The results, depicted in Figure 5A, indicated distinct assembly responses. In agreement with previous findings [47], **PF74** notably enhanced CA assembly, compared with **LEN**, **IC‐2a**, and **IB‐2a**, which showed reduced assembly rates but still promoted minor assembly compared with the CA Apo form. **IC‐2i** and **IC‐2k**, in contrast, were unique in exhibiting no effect on CA assembly at the tested concentration.

HIV‐1 displays significant genetic diversity within and between subtypes, with differences ranging from 10 to 30% across its genome. Although CA is one of the most conserved proteins in HIV‐1, especially in functionally associated regions, outside of these regions, it exhibits lower conservation than other HIV‐1 proteins [59]. Furthermore, due to CA's high degree of flexibility, it can perform various functions. Consequently, considering conformational flexibility and amino acid sequence variations, CAs from different subtypes may exhibit different assembly properties. However, most current studies focus on the CA of subtype B [60]. Therefore, to investigate the effects of new compounds on the assembly of CA from different subtypes, we performed an assembly assay of five other subtype CAs in vitro. The results for these subtypes [59] are presented in Figure 5A.

For subtype A1, **PF74** demonstrated a robust stimulatory effect, significantly accelerating assembly, while **IB‐2a** showed a notable yet less pronounced effect. **IC‐2a** initially facilitated assembly but later led to decreased absorbance, hinting at potential issues related to protein concentration. **IC‐2i** and **IC‐2k** did not stimulate assembly, notably above Apo.

In the case of subtype A2, **PF74**’s efficiency was reduced, whereas **IB‐2a**, **IC‐2a**, and **LEN** all promoted CA assembly. **IC‐2i** and **IC‐2k** did not influence the assembly process, comparable to clade B and A1.

For subtype C, **PF74** and **IB‐2a** rapidly induced assembly, but **PF74**’s effect reversed after 5 min, suggesting a lack of stability in the assembled CAs. **LEN**, **IC‐2i**, and **IC‐2k** showed minimal to no impact on assembly.

Regarding subtype D, **PF74**’s effect was comparable to subtype C. **IB‐2a** similarly promoted assembly, while **LEN** and **IC‐2a** had no effect, and **IC‐2i** and **IC‐2k** inhibited assembly.

In order to obtain initial assembly velocities (AU/min), we focused on the first 2 min of the HIV‐1 CA assembly (linear range; Table S6). This analysis revealed that **PF74** significantly accelerated the assembly of various subtype CA clades, with rates ranging from 2.4 to 21.3 times faster than untreated CA. However, most compounds from our new series did not promote CA assembly.

### 2.11 Impact of Target Compounds on HIV‐1 CANC Assembly in Vitro

We extended our investigation to the assembly of the CANC complex and various CA mutant strains. This assay was adapted from the previously described disassembly assay. Compounds were added prior to the addition of the assembly buffer to assess their effects on CANC assembly. Lower fluorescence intensity indicates a higher degree of CANC tube assembly, which means the compound is able to promoting assembly or improving stability (Figure S3). The insights are depicted in Figures 5B and S3 and reveal that while **IC‐2i** demonstrates a weaker effect on CANC assembly, other compounds, particularly **IC‐2a** and **IC‐2k**, show more potent assembly‐promoting effects compared with **PF74** and **LEN**. Notably, **IC‐2a**’s effect on CANC assembly is almost as significant as that of **IP6**, a critical ligand in promoting the assembly and stability of CA in vivo.

We further expanded our investigation of CANC assembly using transmission electron microscopy (TEM), and representative images are shown in Figure 5C. We observed that coincubation with 50 µM **PF74** and 50 µM **IP6** led to the formation of CA tubes and spheres, respectively. In contrast, coincubation with **LEN** and **IC‐2i** induced abnormal CANC assemblies: LEN resulted in amorphous aggregations, while **IC‐2i** led to flat polymers or small spheroids with diameters around 100–150 nm. TEM and FRET results suggest that **LEN** and **IC‐2i** cause different abnormal assembly patterns in CANC due to their distinct chemical structures. We, therefore, hypothesize that **IC‐2i** may impair the flexibility of the CA hexamer, impeding its proper formation into a tube.

This may explain why the fluorescence value was higher than that of LEN in the CANC assembly assays (Figure S3) and variant from the results in section 2.10. The flat polymers formed by IC‐2i‐treated CANC might not interact efficiently with the reporter tqON, resulting in more exposure of tqON to ExoI and consequently leading to a higher fluorescence signal. Therefore, although the results of this assay may not be able to reflect the actual impact of **IC‐2i** on the CANC assembly, it also illustrates from the side the possibility of forming flat polymers rather than normal CA tubes.

Subsequently, we investigated the effects of compounds **IC‐2a**, **IC‐2i**, **IC‐2k**, and control compounds **PF74**, **LEN**, and **IP6** on the assembly of HIV‐1 CA mutant strains S41A, Q67H, N74D, L56I, and K70R as shown in Figure 5B.

Most RAMs showed reduced self‐assembly capabilities, with the K70R mutant displaying a complete assembly loss. At the test concentration, **IC‐2i** almost lost its promoting effect on the self‐assembly of L56I and N74D and showed reduced effectiveness on S41A and Q67H mutants. Interestingly, **IC‐2i** enhanced the self‐assembly of the K70R mutant to a greater extent than the wild‐type.

Among all compounds tested, **IC‐2a** stood out, showing the most significant resistance and ability to promote assembly in L56I, Q67H, and K70R mutants, surpassing that of **LEN** and **PF74**. For the L56I mutant, in particular, the effect of **IC‐2a** on assembly promotion was notably consistent. However, the effectiveness of **PF74** and **LEN** varied for different mutants, indicating a decrease in sensitivity to L56I, Q67H, K70R, and N74D mutants.

### 2.12 Antiviral Activity of IC‐2i Against HIV‐1 Mutants

Cell culture‐based viral breakthrough assays have identified RAMs located near the **LEN** binding site on CA. Among these, the Q67H and N74D substitutions in CA have emerged as the primary RAMs [61]. These two mutations have also been observed in clinical trials with participants receiving **LEN** [42].

To investigate the antimutant activity of **IC‐2i**, MT‐4 cells infected with several HIV‐1 CA mutants were used in antiviral assays, and the results are shown in Table S4. Overall, all mutations reduced the potency of the compounds. **IC‐2i** was more sensitive (69‐fold) to the Q67H mutation than **LEN** (∼fivefold). As previously reported [49], His67 in the Q67H mutant showed both closed and open conformations, and the closed conformation is preferred in the apo structures and is able to block compound entry (Figure 7B). For the N74D mutation, **IC‐2i** exhibited less sensitivity (11‐fold) to that of **LEN** (∼20‐fold). Both compounds contain a sulfonamide group (blue group in Figure 7B) at the same position, and Asp74 exerts electrostatic repulsion against the acidic nitrogen (Figure 7B), leading to a reduced binding affinity. However, the methane sulfonamide group in **LEN** is slightly more acidic than the ethane sulfonamide group in **IC‐2i** (blue in Figure 7B), because a methyl group is less electron‐donating than an ethyl group, which might be the reason why **IC‐2i** showed lower sensitivity.

K70R has little or no effect on **LEN** potency but it reduces susceptibility about 20‐fold in combination with Q67H. Unfortunately, not only cumulative effects of changes (147‐fold) but also K70R (87‐fold) were serious for **IC‐2i**. Although both arginine and lysine are basic amino acids, the side chain of arginine is much longer than that of lysine, which can cause severe steric hindrance and result in reduced potency of **IC‐2i**.

### 2.13 Crystallographic Studies of IC‐2i Bound to HIV‐1 CA Hexamer

**FIGURE 6:**
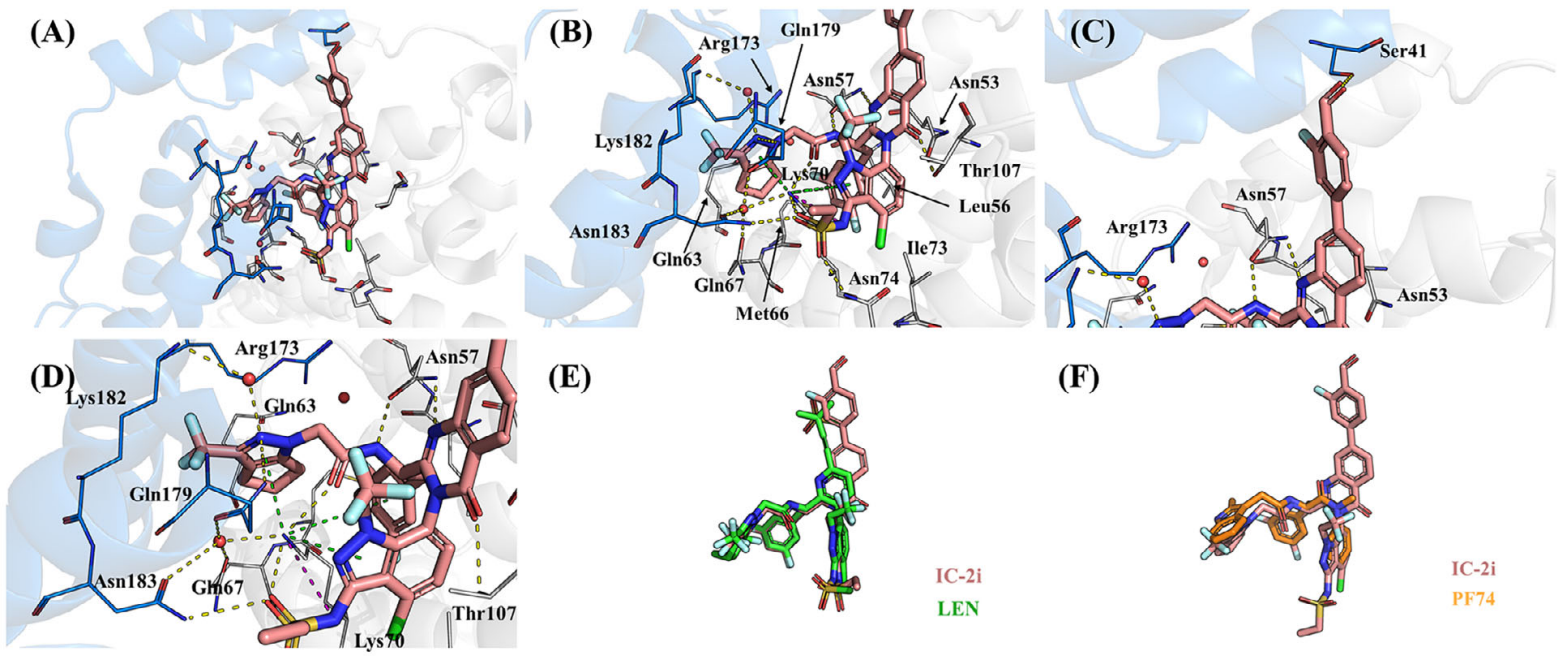
(A) Overview of Binding mode of **IC‐2i**; binding mode of R1, R2, and R3 ring (B), R4 ring (C); (D) the details of binding mode of R3 ring; merge of **IC‐2i** with **LEN** (E); **PF74** (F). Water molecules are shown as red circles. Hydrogen bonds are represented by yellow dashed lines, cation–π interactions as green dashed lines, and electrostatic contacts as purple dashed lines. The figures were generated in PyMOL 2.3.0 (www.pymol.org).

To gain atomic insight into our most potent compound, **IC‐2i**, bound to the HIV‐1 CA hexamer, we obtained the cocrystal structure of **IC‐2i** in complex with the CA hexamer (PDB ID: 8VRP) using X‐ray crystallography. Our structure provides valuable insights compared with the available **LEN**‐CA structure (PDB code:6VKV; Figure 6E) as well as PF74‐CA (PDB code: 4XFZ; Figure 6F). The overall binding position and mode are shared between IC‐2i and LEN. These compounds bind to the HIV‐1 CA with a similar palm‐like envelopment of Lys70 as depicted in Figure 6. The Lys70 sidechain makes hydrogen bond interactions to the sulfonic moiety of R2, the carbonyl of the peptide‐like backbone, and to a highly coordinated bridging water molecule that makes further interactions with Gln67 and Gln 179 from the adjacent monomer CTD.

Waters play a crucial role in facilitating the complex interaction network is of particular significance. These molecules act as vital bridges, firmly anchoring the ligands to the protein, enabling **IC‐2i** to establish a complex network of direct and indirect hydrogen bonds, along with salt bridges and cation–π interactions (Figure 6D).

The detailed binding mode of the main structure of the compound is shown in Figure 6B,D, and the distance between residues and ligand was shown in Figure S3. The difluorophenyl group is bound in the pocket formed by the sidechains of Leu56, Ile73, Met66, and the alkyl chain of Lys70. A highly coordinated water is adjacent to the fluorine within this pocket bound to Gln63 and Arg173. In the R2 system, the indazole is located between Lys 70 and Thr 107 with the sulfonic moiety forming hydrogen bonds with Lys70 and Asn74 as well as Asn183 from the adjacent monomer. The trifluoromethyl group is directed toward the solvent. The R3 pyrazole system is located between Lys70 and the side chain of Arg173 from the adjacent monomer. The pyrazole ring of **IC‐2i** demonstrates a potential cation–π interaction with Lys70 and forms a direct hydrogen bond with Gln179 as well as water‐mediated interactions with Lys182 and Asn183 of the adjacent chain C‐temrinal domain. The newly introduced R4 system with a 4‐quinazolinone is located adjacent to Asn53 with the ring system oriented at 90° to the indazole of R2. The R4 group extends into the space between the NTDs of adjacent monomers. A single hydrogen bond is formed with Thr107 sidechain. In Figure 6C, the newly introduced aldehyde group is within distance of forming a hydrogen bond with Ser41 on the adjacent monomer.

These interactions depict **IC‐2i**’s highly effective binding mode, predominantly driven by hydrogen bonds and cation–π interactions, both inherent and water mediated, resulting in a robust occupancy of the binding pocket.

### 2.14 Molecular Dynamic Simulation on IC‐2i

To investigate the reason why **IC‐2i** showed good activity and the possible mechanism for the loss of sensitivity on the mutants, based on the costructure of **IC‐2i** and CA hexamer, the models for several mutants were built and simulated by molecular dynamic simulation.

**FIGURE 7:**
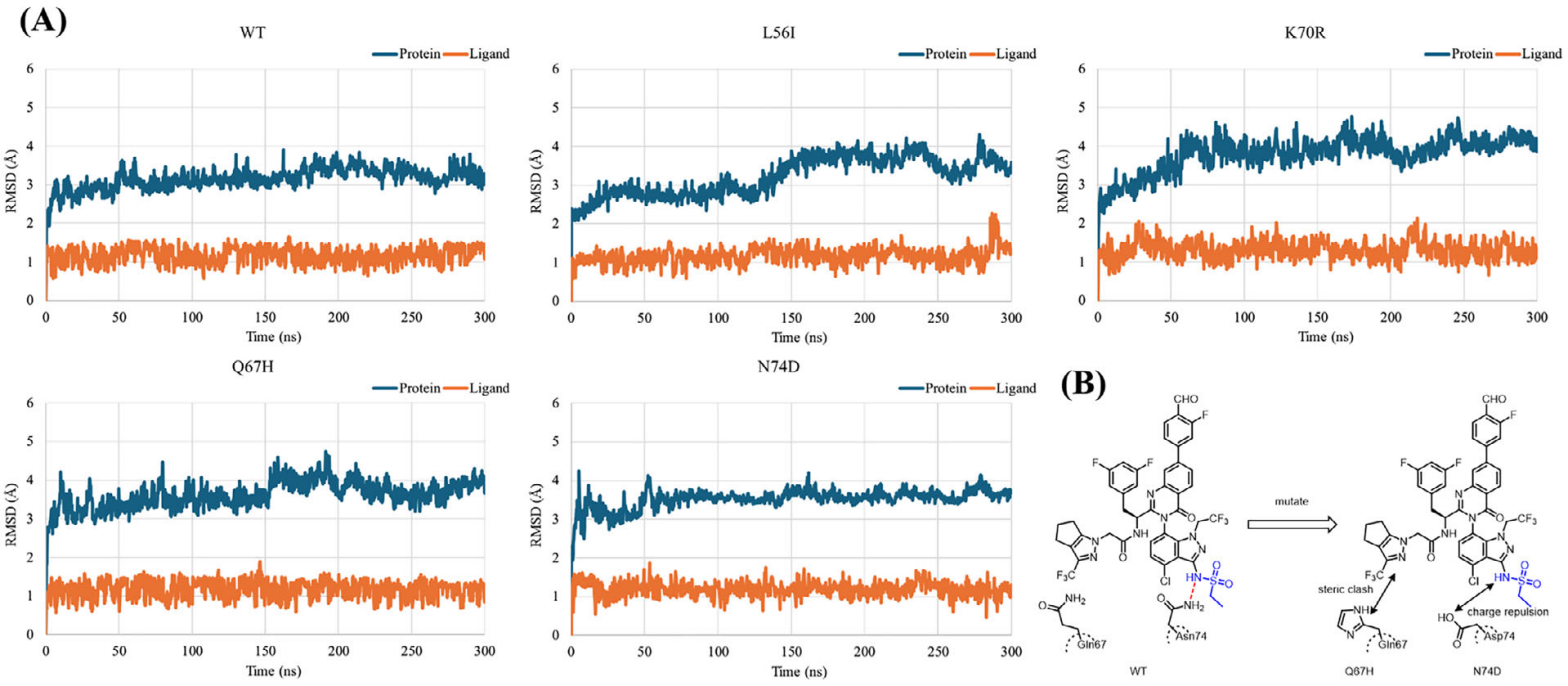
(A) RMSDs of MD simulation for both protein and ligand. (B) 2D schematic diagram of resistance mechanisms of Q67H and N74D.

The RMSDs (root mean square deviation) of the proteins and ligands and MM/GBSA (molecular mechanics/generalized born surface area) are shown in Figure 7A and Table S8. During the 300 ns molecular dynamics simulations, both the wild‐type and mutant complexes reached convergence, indicating that the systems were equilibrated and dynamically stable. However, the RMSD profiles of different mutants showed significant variations. The K70R mutant exhibited a higher RMSD compared with the WT protein, suggesting that the K70R mutant maintained structural stability while adopting a conformational state distinct from that of the WT. The average MM/GBSA binding free energy of the K70R complex was lower than that of the WT complex, implying that the mutation induced local conformational rearrangements that strengthened interactions. The enhanced binding affinity observed in the simulation suggests that the compound may promote a more stable or compact assembled state of the K70R mutant complex. This simulation result is consistent with the results from the CANC disassembly and assembly assays, where the compound reduced CANC disassembly and induced a higher proportion of K70R assembly. Although these results seem to contrast with the outcomes of antimutagenic cellular assays, such discrepancies can be attributed to the inherent complexity of cellular environments and the multifaceted nature of antiviral mechanisms. Therefore, the MD simulation and MM/GBSA analyses should be regarded as a structural‐level interpretation that complements, rather than directly predicts, cellular activity.

The L56I and N74D mutants exhibited higher RMSD and MM/GBSA values compared with the WT, suggesting less stable conformations and weaker compound binding. Notably, the interaction between Asn74 and the sulfone amide of **IC‐2i** almost disappeared in the N74D mutant simulation (Figure S4), which is consistent with the reduced potency of the compound observed in cellular antimutant assays. This structural destabilization is also consistent with the experimental observation that these mutants failed to interfere with the disassembly or assembly of CANC. For the Q67H mutant, the increased RMSD likewise reflected conformational divergence from the WT structure, which may weaken compound binding and consequently diminish its effect on CANC disassembly or assembly.

### 2.15 Stability in Human Liver Microsomes

The liver is the primary organ responsible for drug metabolism in the human body, and within liver cells, liver microsomes play a crucial role in drug metabolism [62, 63]. These microsomes contain various enzyme systems involved in hydrogen peroxide‐related reactions, enabling the oxidation process of different drugs. In our study, we selected representative compounds to assess their metabolic stability in human liver microsomes.

The results, presented in Table S13, were compared with control compounds, including testosterone, diclofenac, and propafenone. **PF74** exhibited rapid metabolism in liver microsomes, while the stability of **IC‐2a** and **IC‐2i** in liver microsomes improved approximately 23‐fold compared with **PF74**. However, a notable difference remained compared with **LEN**, which exhibited a half‐life (*T*_1/2_) exceeding 145 min. Upon analyzing the compounds’ structures, it can be observed that the 1,4,5,6‐tetrahydrocyclopenta[*c*]pyrazole ring of **IC‐2a** and **IC‐2i** possesses three potential oxidizable methylene groups, and the ethyl sulfonyl group may serve as another easily oxidizable site. In the case of **LEN**, these two sites were modified through fluorination, cyclization, and sulfonylation to overcome issues related to poor metabolic stability. Additionally, the aldehyde group in **IC‐2i** might also be a critical site for metabolism, providing insights for further optimization of metabolic stability, such as the preparation of semi‐acetal prodrugs.

### 2.16 Stability in Human Plasma

Plasma contains a variety of drug metabolism enzymes, including cholinesterase, lipase, aldolase, dehydropeptidase, and others. Among the functional groups susceptible to degradation in plasma are amide groups, ester groups, lactams, sulfonamides, lactones, and peptide structures. Given the presence of amide bonds and sulfonamide groups in our compounds, we conducted plasma stability testing on representative compounds.

**FIGURE 8:**
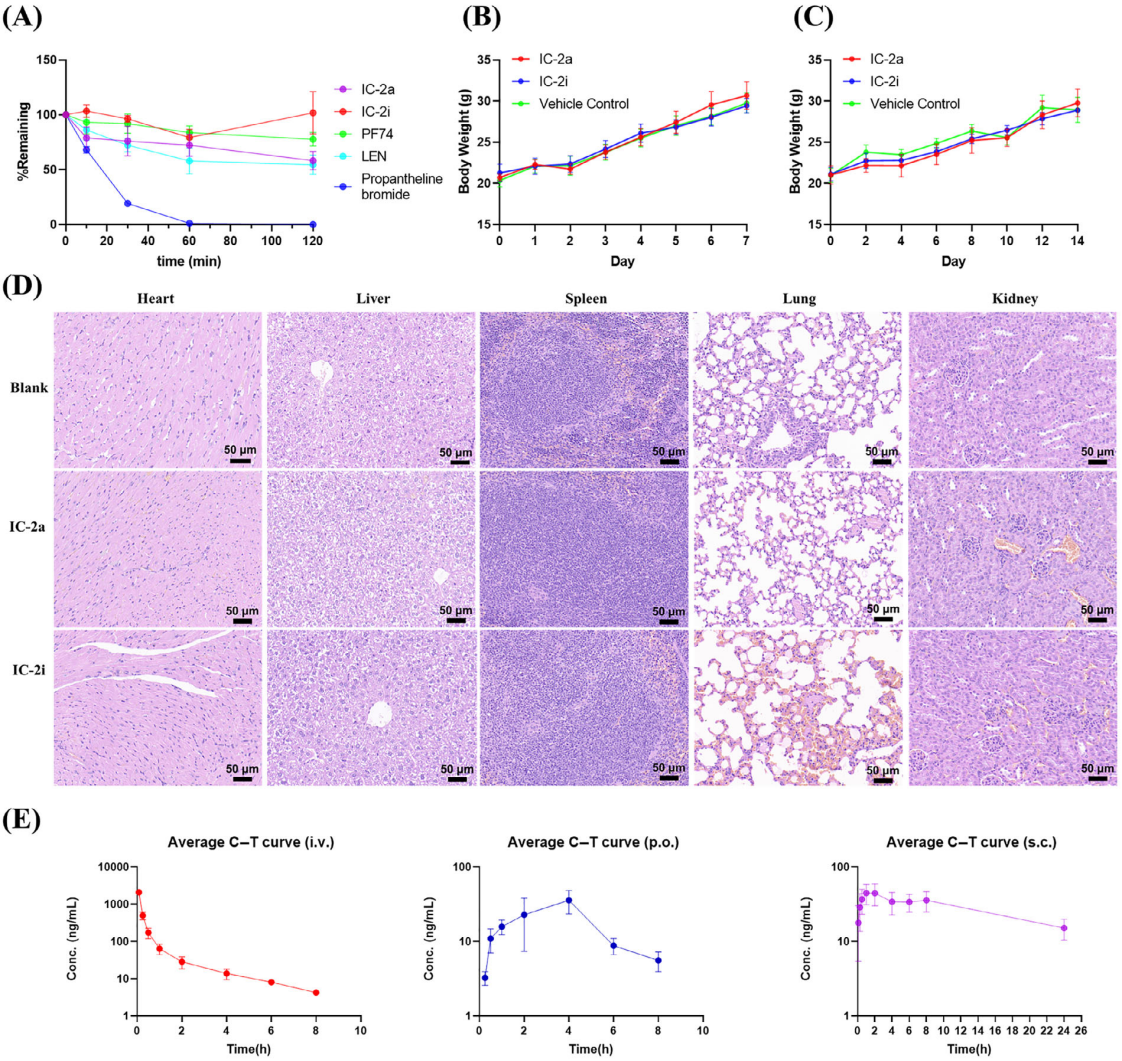
(A) Residual amounts of representative compounds in human plasma at different time points (*n* = 5). (B) Weight of mice in 7 days of acute toxicity assay (*n* = 5). (C) Weight of mice in 14 days of subacute toxicity assay (*n* = 5). (D) Histopathology study of the mice in subacute toxicity assay. The tissues were sectioned and stained with hematoxylin and eosin. (E) Plasma concentration–time profiles of **IC‐2i** in rats following oral or i.v. administration (*n* = 3).

The results are shown in Figure 8A and Table S16. Notably, **IC‐2i** demonstrated exceptional plasma stability and remained nearly intact even at the maximum incubation time of 120 min. Compared with the control compound **PF74**, which also exhibited high stability in plasma, **IC‐2i** had a significantly higher residual amount at the 120‐min mark. Regarding plasma metabolic stability, **LEN** demonstrated a moderate performance with a half‐life (*T*_1/2_) of 140.2 min, falling behind the compounds **IC‐2i** and **IC‐2a** in terms of stability.

### 2.17 Acute and Subacute Toxicity of Representative Compounds

We selected representative compounds **IC‐2a** and **IC‐2i**, which displayed the most potent antiviral activity in vitro, to assess their in vivo toxicity in mice. Overall, all animals remained 100% viable under all tested conditions (data not shown).

At high doses (1000 mg/kg), **IC‐2a** and **IC‐2i** did not exhibit acute toxicity and all the mice remained viable (data not shown). Furthermore, there was no significant difference in weight gain between the experimental and control group mice (Figure 8B), indicating low acute toxicity and good safety of the compounds in vivo. These findings are consistent with the high SI observed in vitro.

No mice died after a dose of 50 mg/kg every 2 days (data not shown), and there was no significant difference in weight gain between the experimental group and the control group over a period of 14 days (Figure 8C). These results indicate that **IC‐2a** and **IC‐2i** have lower subacute toxicity.

Microscopic images of key organs from each group of mice are presented in Figure 8D. Pathological analysis revealed mild hepatic ballooning in the experimental and control group mice, with no significant damage observed in other organs. In conclusion, **IC‐2a** and **IC‐2i** demonstrate in vivo safety that aligns with the high selectivity observed in in vitro experiments.

### 2.18 In Vivo Pharmacokinetics Study

We selected compound **IC‐2i** with excellent antiviral activity for pharmacokinetic testing. The plasma drug concentration–time curves in rats under different administration schemes are depicted in Figure 8E. It is worth noting that the drug concentration at 24 h in the i.v. group and p.o. group fell below the detection limit. Hence, they are not indicated in the figure. The doses administered for the i.v. group, p.o. group, and s.c. group were 2, 10, and 5 mg/kg, respectively. The pharmacokinetic parameters of **IC‐2i** in rats are presented in Table S17.

The oral bioavailability of **IC‐2i** was low (*F* = 4.20 ± 0.388%, *C*_max_ = 37.7 ± 11.0 ng/mL, *T*_1/2_ = 1.60 ± 0.47 h), indicating poor oral absorption. In contrast, subcutaneous (s.c.) injection significantly improved its pharmacokinetic profile: bioavailability increased ∼15‐fold to 61.73 ± 16.1%, and the in vivo half‐life was extended to 19.9 ± 14.2 h (10‐fold longer than oral administration). Additionally, s.c. injection showed favorable sustained‐release characteristics (MRT_0−∞_ = 28.2 ± 21.0 h), supporting its potential as a long‐acting therapeutic candidate.

## 3 Discussion and Conclusion

In this study, we aimed to optimize and overcome emerging clinically relevant resistant mutations in the **LEN** and **GSK878** molecules by innovating on the azole ring, drawing inspiration from the 4‐quinazolinone identified in **GSK878**. Simultaneously, we simplified and modified the azole ring for use in the design of the I‐series of compounds. Employing a structure‐based drug design approach, we examined the drug‐resistant residues of **LEN** and pinpointed the conservative NTD–NTD interface region, focusing on Ser41 as the target for modifications. This led to the synthesis of a novel series of 4‐quinazolinone‐bearing potent anti‐HIV‐1 agents with shorter synthesis scheme (8–9 steps) and low cost building blocks.

The new compounds, particularly **IC‐2a**, **IC‐2i**, and **IC‐2k**, displayed potent antiviral activity against HIV‐1, with anti‐HIV_NL4‐3_ EC_50_ below 1 nM. The compounds also demonstrated a high affinity for CA hexamers, particularly **IC‐2i,** with a *K*_D_ of 2.7 ± 0.5 nM. Moreover, as expected, all tested compounds showed reduced HIV‐1 CA monomer binding due to the lack of a defined interprotomer pocket and reduced interaction network. **IC‐2i** showed dual‐stage inhibition, indicating interference with the CPSF6 and NUP153 binding and inhibition on the both early and late RT without inhibiting RT. This points toward a potential impact on nuclear import and CA assembly disruption in the early stage, as well as interference with CA/polyGag plasma membrane translocation/assembly in the late stage. Besides, IC‐2i showed better tolerance for N74D mutant. Crystallographic studies revealed the binding mode of **IC‐2i** and emphasized the importance of water molecules within the interaction network. MD simulation based on crystallography results revealed a potential mechanism of stronger accelerating effect on K70R CANC assembly and mechanism for drug resistance. Preliminary studies indicated good drug‐likeness and safety profiles, with **IC‐2i** showing longer‐acting pharmacokinetic properties.

One of the limitations we acknowledge is that CPSF6 and NUP153 bind isolated CA hexamers with low‐to‐mid micromolar affinity, whereas engagement of assembled CA lattices/cones can reach sub‐micromolar to apparent nanomolar avidity due to multivalent interactions. This avidity arises when multiple FG/low‐complexity motifs in these factors sample repeated copies of the NTD–CTD pocket and/or condense with CA assemblies, phenomena not captured by a 1:1 hexamer format or readily modeled by SPR [64]. Accordingly, our SPR experiments were designed to quantify intrinsic pocket competition at the hexameric interface (i.e., whether inhibitors displace CPSF6/NUP153 FG‐containing peptides from the shared site), rather than to reproduce absolute lattice avidity. We therefore interpret the SPR data together with our cell‐based single‐cycle and multicycle infection assays, which report the net phenotypic outcome in the native lattice context and provide EC_50_ values for fully infectious virus.

Additional limitations of this study are that the antiviral activity of **IC‐2i** still has a gap with the clinical drug **LEN**. At the same time, we have to see that the oral bioavailability of **IC‐2i** is poor, which means the novel compounds in the future should focus on the finding and modification of the easy‐metabolized groups and further improve the oral bioavailability, good for patient compliance. Furthermore, as LEN is now clinically used, more CA mutants and drug‐resistant strains are expected to emerge. Although **IC‐2i** shows better inhibitory activity against the N74D mutant compared with LEN, further investigations into its activity against a broader range of mutants are needed. The limited activity of **IC‐2i** against drug‐resistant variants currently restricts its clinical potential. Nevertheless, the insights gained from characterizing the inhibitory profile of **IC‐2i** toward various CA mutants may help guide the design of more potent antimutant compounds and RAMs found in the future should be tested in a timely manner for both **IC‐2i** and future analogs.

Overall, this study reports the design and development of 4‐quinazolinone‐containing phenylalanine derivatives as novel HIV‐1 CA inhibitors. **IC‐2i**, the lead compound, exhibits potent antiviral activity, resistance tolerance, and favorable pharmacokinetic properties. Its unique binding mode targeting both NTD–CTD and NTD–NTD interfaces provides a new strategy for overcoming drug resistance. With further optimization of metabolic stability and oral bioavailability, **IC‐2i** holds great potential as a long‐acting therapeutic agent for HIV‐1 infection, addressing unmet clinical needs in current antiretroviral therapy.

## 4 Methods

### 4.1 Chemistry

^1^H NMR, ^13^C NMR, and ^19^F NMR spectra were acquired using Bruker AV‐400 or AV‐600 spectrometer, with the solvents specified (DMSO‐*d_6_* or chloroform‐*d*). Chemical shifts are expressed as *δ* values (ppm) and referenced to tetramethylsilane as an internal standard, while coupling constants (*J*) are given in hertz (Hz). Melting points were measured using a micromelting point apparatus without correction. Thin‐layer chromatography analyses were carried out on Silica Gel GF254 plates (Merck), and visualization was achieved either under UV light at 254 nm or by exposure to iodine vapor. Flash column chromatography was conducted using Silica Gel 60 (200–300 mesh) as the stationary phase. All solvents were of reagent grade and, when required, were dried and purified according to standard procedures. Reaction mixtures were concentrated under reduced pressure using a rotary evaporator. Dichloromethane, triethylamine (Et_3_N), methanol, and related solvents were supplied by Sinopharm Chemical Reagent Co., Ltd (SCRC) and were of analytical reagent (AR) grade. Key starting materials were obtained from Bide Pharmatech Co., Ltd or Shanghai Haohong Scientific Co., Ltd. The purity of the final compounds was determined using a Shimadzu LC‐20A HPLC system. The chromatographic conditions were as follows: an Agilent ZORBAX SB‐C18 column (250 mm × 4.6 mm, 5 µm); isocratic elution with methanol (mobile phase A) and ultrapure water (mobile phase B) in a ratio of 80:20 (v/v); flow rate of 1.0 mL/min; detection wavelength set at 254 nm; column temperature maintained at 30°C; injection volume of 10 µL; and a total run time of 10 min. Purity was calculated based on the area normalization method. Elemental analyses (C, H, N, S) were performed on Elementar UNICUBE (Elementar Analysensysteme GmbH). The synthesis steps and compounds characterization can be found in the supporting information.

### 4.2 In Vitro Anti‐HIV Assay in MT‐4 Cells and Cytotoxicity Assay

#### 4.2.1 Anti‐HIV‐1‐NL4‐3 Activity in MT‐4 Cells and Cytotoxicity Assay

MT4 cells (1 × 10^5^ cells/mL) were infected in 96‐well plates with HIV‐1 NL4‐3 Δnef‐secNluc at an infectious dose of 50 TCID_50_ per well in the presence of test compounds at different concentrations. The recombinant reporter virus HIV‐1 NL4‐3 Nanoluc‐sec was generated by replacing the Nef coding region (nucleotides 8796–8892) in the pNL4‐3 plasmid (GenBank: AF324493.2) with the secNluc fragment derived from pNL1.3[secNluc] (Promega; Cat# N1021). This substitution was achieved using *Not* I and *Xho* I restriction sites. The *Not* I site was introduced into pNL4‐3 via site‐directed mutagenesis, while the *Xho* I site is inherently present as a unique restriction site within the plasmid. The reporter construct was generously provided by Hal Bogerd from the laboratory of Dr. Brian Cullen at Duke University. At 72 h postinfection, culture supernatants were collected and analyzed for luciferase activity using the Promega Nano‐Glo Luciferase Assay System. Antiviral activity was quantified by calculating the EC_50_ value, defined as the concentration of compound required to achieve a 50% reduction in luciferase signal.

Cytotoxicity of the synthesized BA derivatives was evaluated using the CytoTox‐Glo assay (Promega). MT4 cells were incubated with varying concentrations of the compounds for 72 h. The assay was performed according to the manufacturer's instructions, and cell viability was assessed accordingly. The CC_50_ value was defined as the compound concentration leading to a 50% decrease in viable cells.

#### 4.2.2 Anti‐HIV‐IIIB and Anti‐HIV‐ROD Activity in MT‐4 Cells and Cytotoxicity Assay

The anti‐HIV activity of the compounds in MT‐4 cells was evaluated using an MTT‐based assay as previously described [65]. Test compounds were prepared as stock solutions at 10‐fold the desired final concentrations, and 25 µL aliquots were dispensed into two sets of triplicate wells to simultaneously assess their effects on both HIV‐infected and mock‐infected cells at the start of the experiment. Serial dilutions (fivefold) of the compounds were carried out directly in flat‐bottom 96‐well microtiter plates with the assistance of a Biomek 3000 automated workstation (Beckman Instruments, Fullerton, CA). Control wells containing untreated HIV‐infected and mock‐infected cells were included in parallel. Subsequently, 50 µL of HIV suspension (100–300 CCID_50_) or culture medium was added to the designated infected or mock‐infected wells, respectively. Mock‐infected cells served to evaluate compound‐associated cytotoxicity in the absence of viral infection. Exponentially growing MT‐4 cells were collected by centrifugation at 220×*g* for 5 min, after which the supernatant was removed. The cell pellet was resuspended to a density of 6 × 10^5^ cells/mL, and 50 µL portions were distributed into each well of the microtiter plate. After a 5‐day incubation period postinfection, cell viability in both infected and mock‐infected wells was determined spectrophotometrically using the MTT assay. This assay relies on the enzymatic reduction of the yellow tetrazolium salt 3‐(4,5‐dimethylthiazol‐2‐yl)‐2,5‐diphenyltetrazolium bromide (MTT; Acros Organics) by mitochondrial dehydrogenases in viable cells, generating a blue‐purple formazan product detectable by absorbance measurement. Absorbance values were recorded at 540 and 690 nm using an eight‐channel automated microplate reader (Infinite M1000; Tecan). Data analysis was performed based on the median absorbance obtained from three replicate wells. The CC_50_ value was defined as the concentration of compound causing a 50% reduction in OD_540_ in mock‐infected control cells. The EC_50_ value was defined as the concentration required to achieve 50% protection against virus‐induced cytopathic effects in infected cells.

### 4.3 Determination of the Mechanism of Representative Compounds

#### 4.3.1 Cells

Human embryonic kidney 293T cells (kindly provided by Dr. Irwin Chaiken, Drexel University, Philadelphia, PA) were maintained in Dulbecco's modified Eagle's medium (DMEM) supplemented with 10% fetal bovine serum (FBS), 100 U/mL penicillin, 100 µg/mL streptomycin, and 2 mM l‐glutamine. Human astroglioma U87 cells stably expressing CD4 and CXCR4 (obtained from Prof. Hongkui Deng, Peking University, and Prof. Dan Littman, New York University, USA, via the AIDS Research and Reference Reagent Program, Division of AIDS, NIAID, NIH) [66, 67] were cultured in DMEM containing 10% FBS, 100 U/mL penicillin, 100 µg/mL streptomycin, and 2 mM l‐glutamine, supplemented with 300 µg/mL G418 (Thermo Scientific, Waltham, MA) and 1 µg/mL puromycin (Thermo Scientific). Unless otherwise specified, all cells were incubated at 37°C in a humidified atmosphere of 5% CO_2_ and 95% air.

#### 4.3.2 Proteins

The monoclonal antibody IgG b12 against HIV‐1 gp120 was obtained through the NIH AIDS Reagent Program (Division of AIDS, NIAID, NIH; provided by Dr. Dennis Burton and Carlos Barbas). The HIV‐1 CA protein p24 was produced in‐house following previously reported procedures [68]. In brief, a plasmid encoding C‐terminal His‐tagged HIV‐1 NL4‐3 CA (a gift from Dr. Eric Barklis, Oregon Health and Science University, Portland, OR) was transformed into BL21‐Codon Plus (DE3)‐RIL competent cells (Agilent Technologies, Wilmington, DE). The transformed cells were cultured overnight in autoinduction ZYP‐5052 medium at 30°C with shaking at 225 rpm [69]. Cells were harvested by centrifugation at 8200 × g, and the pellets were resuspended in PBS followed by lysis via sonication. The clarified lysate was immediately loaded onto a Talon cobalt affinity resin column (Clontech Laboratories, Mountain View, CA). Bound proteins were eluted using PBS containing 250 mM imidazole. Purified CA‐H_6_ monomers were dialyzed overnight at 4°C against 20 mM Tris–HCl (pH 8.0), concentrated to 120 µM, flash‐frozen in liquid nitrogen, aliquoted, and stored at −80°C. To generate CA hexamers, mutations (A14C, E45C, W184A, and M185A) were introduced by site‐directed mutagenesis (Stratagene). Expression and purification of the hexameric construct followed the same procedure as described above. After purification, CA‐H_6_ hexamers were dialyzed into 200 mM β‐mercaptoethanol (β‐ME), followed by stepwise dialysis to gradually remove β‐ME and promote hexamer assembly.

#### 4.3.3 Production of Pseudotyped Viruses

Single‐cycle infectious pseudotyped viruses carrying a luciferase reporter were generated by cotransfecting two plasmids into 293T cells seeded in six‐well plates (1 × 10^6^ cells per well) [67]. Plasmid 1 (3 µg) consisted of the envelope‐deficient HIV‐1 pNL4‐3‐Luc+R−E construct encoding the luciferase reporter gene [55], while plasmid 2 (4 µg) encoded the HIV‐1 BG505 gp160 Env protein [56]. Transfection was performed using a calcium phosphate‐based method (ProFection Mammalian Transfection System; Promega, Madison, WI) for 5 h. Following incubation, the transfection medium containing DNA was removed, and the cells were washed with DMEM before adding fresh culture medium. Virus‐containing supernatants were harvested at 72 h posttransfection, clarified, filtered, aliquoted, and stored at −80°C until further use.

#### 4.3.4 SPR Interaction Analysis

For kinetic SPR, we immobilized disulfide‐stabilized HIV‐1 CA hexamers [70], a homogeneous construct that preserves the NTD–CTD interprotomer pocket engaged by CPSF6, NUP153, and PF74 and is widely used for quantitative ligand/peptide (FG‐containing peptides) assessment. We selected the hexamer rather than extended CA lattices because lattices introduce multivalency‐driven avidity and mass‐transport limitations that complicate kinetic interpretation, whereas the stabilized hexamer supports well‐behaved 1:1 fits and direct pocket‐competition measurements. All experiments were conducted using a ProteOn XPR36 SPR Protein Interaction Array System (Bio‐Rad Laboratories, Hercules, CA) at a constant temperature of 25°C. ProteOn GLH sensor chips were pretreated with two 10 s injections each of 50 mM NaOH, 100 mM HCl, and 0.5% sodium dodecyl sulfate, followed by equilibration in PBS‐T buffer (20 mM sodium phosphate, 150 mM NaCl, 0.005% polysorbate 20, pH 7.4). Chip surfaces were activated using a 1:100 dilution of a freshly prepared 1:1 mixture of 0.2 M 1‐ethyl‐3‐(3‐dimethylaminopropyl) carbodiimide hydrochloride and 0.05 M sulfo‐N‐hydroxysuccinimide. Immediately after activation, HIV‐1 NL4‐3 CA protein constructs (100 µg/mL in 10 mM sodium acetate, pH 5.0) were injected across the ligand channels at 30 µL/min for 5 min. Residual active esters were subsequently quenched with 1 M ethanolamine HCl (pH 8.0) for 5 min at 5 µL/min. A reference surface was generated in parallel by immobilizing a nonspecific protein (IgG b12 anti‐HIV‐1 gp120; obtained from the NIH AIDS Reagent Program, Division of AIDS, NIAID, NIH, originally from Dr. Dennis Burton and Carlos Barbas), which served to correct for nonspecific interactions. Both HIV‐1 CA NL4.3 monomer and the IgG b12 reference were immobilized to approximately 6000 RU. For analyte preparation, compound stock solutions were combined with DMSO and adjusted to a total volume of 1 mL using PBS (pH 7.4), ensuring a final DMSO concentration equivalent to that in the running buffer (3% DMSO). Serial dilutions were prepared in running buffer (PBS supplemented with 3% DMSO and 0.005% polysorbate 20, pH 7.4). Samples were injected at 100 µL/min with a 1 min association phase followed by up to 5 min dissociation using the “one‐shot kinetics” mode of the instrument. Data processing was carried out using ProteOn Manager Software (version 3.0, Bio‐Rad). Responses from the reference channel were subtracted to account for nonspecific binding and bulk effects. Where applicable, data were fitted using a 1:1 binding model. Kinetic (kd) and equilibrium parameters were calculated from three independent replicates and used to determine dissociation rates and equilibrium dissociation constants (*K*_D_).

Competition assays with CPSF6 peptides were performed using the coanalyte feature of the ProteOn system. Compounds (200 nM; PF74 and 11L at 5 µM) were first injected to occupy the CA binding sites. Without allowing dissociation, a second injection was applied containing the same compound concentration together with either 100 µM CPSF6 or 150 µM NUP153 peptides at a flow rate of 100 µL/min for a 1 min association phase. Equilibrium responses were recorded and compared with control injections of peptide alone. At least three independent injections were analyzed for each CA hexamer surface. Statistical analysis was conducted using paired comparisons, and group means were evaluated by Tukey's method. Box plots display the mean (central line), individual data points (three or four per condition), standard error of the mean (box), and 90% confidence intervals (whiskers).

#### 4.3.5 SRI Assay

The single‐round HIV‐1 infection assay was carried out as previously described [55, 71, 72]. U87.CD4.CXCR4 target cells (1.2 × 10^4^ cells per well) were seeded into 96‐well luminometer‐compatible plates (Greiner Bio‐One). After 24 h, compounds or DMSO (vehicle control; Sigma) were mixed with pseudotyped virus preparations normalized based on p24 levels. The mixture was then added to the cells and incubated at 37°C for 48 h. Following incubation, culture medium was removed, and cells were lysed by adding 50 µL per well of luciferase lysis buffer (Promega) and subjecting the plate to one freeze–thaw cycle. Luciferase activity was quantified using a GloMax 96 microplate luminometer (Promega) after addition of 50 µL per well of luciferase substrate (Promega).

#### 4.3.6 Viral Late‐Stage Infection Assay

For late‐stage infection analysis, single‐round infectious envelope‐pseudotyped luciferase reporter viruses were generated in 293T cells [67] in the presence of either 100 µM test compound or DMSO (vehicle control; Sigma). After incubation for 48 h at 37°C, supernatants containing pseudotyped viruses were collected and diluted 20‐fold prior to infection. The diluted virus preparations were used to infect U87.CD4.CXCR4 target cells, which were then incubated for an additional 48 h at 37°C. Subsequently, the culture medium was removed, and cells were lysed with 50 µL luciferase lysis buffer (Promega), followed by one freeze–thaw cycle. Luciferase signals were measured using a GloMax 96 luminometer (Promega) after addition of 50 µL luciferase substrate per well. Compound effects were evaluated as reductions in viral infectivity, reflected by decreased luciferase activity in target cells, and normalized to the infectivity of viruses produced from DMSO‐treated control samples.

#### 4.3.7 Protocol for RT Inhibiting Assay

##### 4.3.7.1 Anti‐HIV‐1 RT Assay

HIV RT inhibitory activity of all tested compounds was performed using the HIV‐1 Reverse Transcriptase Assay Kit (XpressBio, MD, USA) according to manufacturer protocol.

##### 4.3.7.2 HIV Reverse Transcription Inhibition Assay

HeLa cells (5 × 10^5^ cells/well) were seeded into 24‐well plates. After cell attachment, 100 µL of virus stock pseudotyped with VSV‐G and pNL4‐3‐ΔE‐ΔR‐Luc was added to each well. Cells were treated simultaneously with 10 µM of the test compound **IC‐2i**, the corresponding concentration of DMSO (negative control), or TDF (positive control for reverse transcription inhibition). After 48 h of incubation, cells were harvested, and genomic DNA was extracted. Reverse transcription products were quantified by real‐time PCR (qPCR) using SYBR Green chemistry.

#### 4.3.8 Protocol for CA Assembly Assay

In vitro assembly of monomeric HIV‐1 CA proteins was carried out using a modified turbidity‐based assay as previously reported [73, 74, 75, 76, 77]. This method is based on the principle that elevated salt concentrations induce CA self‐assembly, which can be monitored as a time‐dependent increase in turbidity, as described by Kortagere et al. [68, 78]. Assembly experiments were performed using CA proteins from multiple HIV‐1 subtypes, including subtype A1 (92UG037.1), subtype A2 (CDKTB48), subtype C (92BR025.8), and subtype D (94UG114.1). For each reaction, 75 µL of assembly buffer containing 50 mM NaH_2_PO_4_ (pH 8.0) and 3 M NaCl was prepared to initiate assembly upon addition of protein. Subsequently, 25 µL of purified CA protein (120 µM) was added to yield a final CA concentration of 30 µM, with DMSO concentrations kept consistent across samples. For compound‐treated conditions, the reaction mixture consisted of 50 mM NaH_2_PO_4_ (pH 8.0), 30 µM CA protein, 50 µM inhibitor, 3% DMSO, and 3 M NaCl. Turbidity changes were monitored by measuring absorbance at 350 nm at 10 s intervals over a total period of 35 min. To determine the initial assembly rate, OD_350_ values recorded during the first 2 min were plotted and fitted by linear regression, and the slope was used to calculate the initial velocity expressed as AU/min.

#### 4.3.9 Effects on Assembly and Disassembly of CANC

##### 4.3.9.1 Effects on Disassembly of CANC

The protocol was referenced from the Refs. [57, 58]. CANC protein was availed from Shenzhen Kangti Biomedical Technology Co., Ltd. 12.5 µL 60 µg CANC solution (20 mM Tris–HCl, pH 8.0, 0.5 M NaCl, 50 µM ZnCl_2_, 10 mM DTT, 1 mM PMSF) was taken, 1.25 µL 20% (v/v) DMSO was added, and was incubated for 1 h on the ice. 10 µL assembly buffer (62.5 mM Tris–HCl, pH 8.0, 125 mM NaCl, 1.25 µM ZnCl_2_) and 1.25 µL 20 µM tqON (Azenta Life Sciences) were mixed in advance and was added to the mixture, so that the final mixture was 25 µL. It was incubated for 3 h at room temperature. Then, 2.5 µL test compounds were added with end concentration of 50 µM and incubated for 3 h at room temperature. Then, 22.5 µL disassembly buffer (55.6 mM Tris, pH 7.0, 1.11 µM ZnCl_2_) was added and incubated for 16 h at room temperature. Finally, 1 µL 20 U/µL exonuclease 1 (NEB) and 5.5 µL 10× MgCl_2_ were added, and it immediately detected 120 min by fluorescent reader (SpectraMax iD5, Molecular Devices), the excitation and emission wavelengths for which corresponded to 480 and 520 nm, respectively.

##### 4.3.9.2 Effects on Assembly of CANC

The protocol was referenced from the Refs. [57, 58]. 25 µL 60 µg CANC solution (20 mM Tris–HCl, pH 8.0, 0.5 M NaCl, 50 µM ZnCl_2_, 10 mM DTT, 1 mM PMSF) was added; then, 2.5 µL test compounds in 20% (v/v) DMSO with end concentration of 50 µM was added and was incubated for 1 h on the ice. 20 µL assembly buffer (62.5 mM Tris–HCl, pH 8.0, 125 mM NaCl, 1.25 µM ZnCl_2_) and 2.5 µL 20 µM tqON (Azenta Life Sciences) were mixed in advance, and was added to the mixture, so that the final mixture was 50 µL. It was incubated for 3 h at room temperature. Finally, 1 µL 20 U/µL exonuclease 1 (NEB) and 5.5 µL 10× MgCl_2_ were added, and it immediately detected 120 min by fluorescent reader (SpectraMax iD5; Molecular Devices), the excitation and emission wavelengths for which corresponded to 480 and 520 nm, respectively. The particles formed during the assembly reaction were visualized using negative staining techniques. Photomicrographs were captured using HT‐7800 electron microscope.

#### 4.3.10 Antiviral Activity of Compound Against HIV‐1 Mutants

HEK293T cells were transfected with VSV‐G and either wild‐type or mutant pNL4‐3‐ΔE‐ΔR‐Luc plasmids. At 48 h posttransfection, culture supernatants were collected and clarified by centrifugation at 1600 × g for 15 min at 4°C to remove cellular debris. The resulting wild‐type or mutant viral stocks were filtered through a 0.45‐µm membrane and stored at −80°C until use.

MT4 cells (1 × 10^5^ cells/well) were seeded into 96‐well plates and treated with the indicated concentrations of the antiviral compound IC‐2i. Subsequently, 50 µL of viral supernatant and polybrene (10 µg/mL) were added to each well. After 48 h of incubation, plates were centrifuged at 1200 × *g*, and culture supernatants were removed. Cells were lysed in 50 µL of lysis buffer on ice for 10 min, followed by centrifugation to collect the clarified lysates. Firefly luciferase activity was measured by mixing lysates with luciferase substrate and recording luminescence.

#### 4.3.11 Protocol for HIV‐1 p24 Quantification

The p24 levels in pseudovirus preparations were quantified using an HIV‐1 Gag p24 Antibody Pair ELISA kit (Novus Biologicals, USA). Briefly, ELISA plates were coated overnight at 4°C with 2 µg/mL mouse anti‐HIV‐1 p24 monoclonal antibody in PBS (pH 7.4). Plates were then blocked with 3% BSA for 2 h at room temperature and washed with PBST (PBS containing 0.05% Tween‐20). Pseudovirus samples were lysed with 0.1% Triton X‐100 (Sigma–Aldrich, St. Louis, MO) at 37°C for 1 h, followed by incubation on the coated plates overnight at 4°C. In parallel, purified p24 protein (Novus Biologicals) was included to generate a standard calibration curve. After incubation, plates were washed with PBST and incubated for 1 h at room temperature with 0.1 µg/mL HRP‐conjugated mouse anti‐HIV‐1 p24 monoclonal detection antibody prepared in PBS containing 50% HRP Protector (pH 7.4, stored at 4°C). Excess antibody was removed by washing with PBST. Subsequently, substrate solution consisting of 0.4 mg/mL o‐phenylenediamine in phosphate–citrate buffer with sodium perborate (Sigma–Aldrich) was added and incubated in the dark for 30 min. Absorbance was then measured at 450 nm using a Multiskan GO microplate spectrophotometer (Thermo Scientific).

### 4.4 Acute Toxicity Experiment

Kunming mice (17–20 g) were obtained from Beijing Vital River Laboratory Animal Technology Co., Ltd. All procedures were conducted in accordance with the guidelines of the Animal Care and Use Committee of Shandong University. Test compounds were formulated in a mixture of polyethylene glycol (PEG) 400, normal saline, and DMSO (75:20:5, v/v/v) to a maximum concentration of 100 mg/mL. Following a 12 h fasting period, mice received the formulation via intragastric gavage. A dose of 1000 mg kg^−1^ was administered to groups of five male mice. Animals were monitored for signs of toxicity, including mortality, changes in body weight, tremors, convulsions, body jerks, reduced activity, hunched posture, and piloerection.

### 4.5 Crystallization and X‐Ray Data Collection of HIV‐1 CA

For crystallization experiments, disulfide‐linked hexamers of HIV‐1 CA were prepared using the full length HIV‐1 A14C/E45C/W184A/M185A construct as described previously [79]. Briefly, the full‐length HIV‐1 CA protein was expressed with an C‐terminal his‐tag from a pET22b plasmid in Escherichia coli LOBSTR(DE3) expression cells. Cells were grown to an OD_600_ of 0.6 and expression was induced overnight at 28°C by the addition of 0.1 mM IPTG. Cells were harvested by centrifugation and resuspended in lysis buffer of 20 mM Tris/HCl pH 7.8, 300 mM NaCl, and 20 mM BME. Cells were lysed using a cell disruptor and lysate cleared by centrifugation. Protein was purified by immobilized metal affinity chromatography and size‐exclusion chromatography in 10 mM Tris/HCl pH 7.8, 150 mM NaCl, and 20 mM BME.

To generate disulfide‐linked hexamers, purified HIV‐1 A14C/E45C/W184A/M185A protein was dialysed against high‐salt buffer in the presence of 20 mM BME (20 mM BME, 150 mM NaCl, 20 mM TRIS/HCl pH 8.0), before being transferred to a high‐salt solution (20 mM BME, 1 M NaCl, 20 mM TRIS/HCl pH 8.0) solution overnight at 4°C. This promotes the formation of tubular assemblies that mimic the mature CA lattice seen in HIV‐1 virions. These assemblies were then dialysed (1 M NaCl, 20 mM TRIS/HCl pH 8.0) to remove the reducing agent and promote the formation of disulfide bonds between adjacent CA monomers. In the final dialysis excess salt is removed (150 mM NaCl, 20 mM TRIS/HC pH 8.0) causing tubes to disassemble resulting in disulfide cross‐linked hexameric CA (HIV‐1 CA‐Hex). Disulfide crosslinked hexameric CA was purified by SEC using an S200 16/60 column in 10 mM Tris/HCl pH 8.0, 150 mM NaCl. The assembly was confirmed by nonreducing SDS‐page. Purified hexamers were concentrated to 10 mg/mL and stored frozen at −20°C.

Crystallization screens were undertaken with a 1:1.1 ratio of protein to IC‐2i compound (added from a 50 mM stock in 100% DMSO). Crystals were obtained in the Morpheus screen condition B2 (10% w/v PEG 8000, 20% v/v ethylene glycol 0.03 M of each halide 0.1 M MES/imidazole pH 6.5). For data collection, crystals were harvested in a small nylon loop and directly frozen in liquid nitrogen. Data were collected at the Australian Synchrotron on beamline MX2 at a wavelength of 0.95373 Å. Crystals belong to the spacegroup P6 and diffracted to a resolution of 1.8 Å. The structure was determined by molecular replacement using a monomer of HIV‐1 CA as a search model (PDBid: 8V17) in PHASER [80]. Three equivalent molecules are present within the asymmetric unit (see Table S7 for data collection and refinement statistics). The model consists of residues Pro1‐Gly222 (Ch A), Pro1‐Gly220 (Ch B), and Pro1‐Val221 of Chain C. The remaining C‐terminal residues are not visible in the electron density map and have therefore not been modelled. The structure was refined to a final R/Rfree of 19.2/22.4 with iterative refinement in REFMAC5.0 [81] and model building in COOT [82]. The refined structure is available in the PDB with the accession code 8VRP. The IC‐2i molecule was generated using the coot ligand builder with initial restraints then generated in phenix.elbow [83]. Ligand restraints were further refined by comparing the refined molecule with the ccdc small molecule database using mogul [84].

### 4.6 Molecular Dynamic Simulation

The system preparation and simulation were performed in Schrödinger Suite Release 2021–4. The cocrystal structure of IC‐2i bound to CA obtained from the crystallization assay was prepared using the default settings of the Protein Preparation Wizard and subsequently mutated. The resulting complexes were used as the initial coordinates, placed in an appropriately sized simulation box, and solvated with TIP3P water. Counterions (Na^+^ or Cl^−^) were added to neutralize the system, and 0.15 M NaCl was added to mimic physiological ionic strength. The entire system was relaxed using the default relaxation protocol, and production molecular dynamics simulations were performed for 300 ns at 300 K and 1 bar. The resulting trajectories were analyzed to calculate the RMSD values, and the binding free energy was estimated using the MM‐GB/SA method.

### 4.7 Subacute Toxicity Experiment

Kunming mice (17–20 g) were sourced from Beijing Vital River Laboratory Animal Technology Co., Ltd., and all experimental procedures complied with institutional ethical guidelines of Shandong University. Compounds were prepared in PEG 400/normal saline/DMSO (75:20:5, v/v/v) at concentrations up to 100 mg/mL and administered by gavage following 12 h fasting. A dose of 50 mg kg^−1^ was given to groups of five male mice every 2 days over a 2‐week period. Throughout the study, animals were observed for mortality and clinical signs, including body weight changes, tremors, convulsions, body jerks, hypoactivity, hunched posture, and piloerection. After 14 days, animals were sacrificed, and major organs (heart, liver, spleen, lungs, and kidneys) were collected. Tissues were fixed, embedded in paraffin, sectioned, and subjected to hematoxylin and eosin staining for histopathological examination.

### 4.8 Pharmacokinetics Study

Male SD rats (SPF, 180–220 g) were purchased from Beijing Vital River Laboratory Animal Technology Co., Ltd. The research protocol complied strictly with the institutional guidelines of the Animal Care and Use Committee at Shandong University. The nine rats were randomly divided into three groups, i.v. group, p.o. group, and s.c. group, and the dosage was 2, 10, and 5 mg/kg, respectively. After the administration, the blood vein was taken from the cervical vein, and the blood collection time is 5 min, 15 min, 30 min, 1 h, 2 h, 4 h, 6 h, 8 h, and 24 h after administration. Heparin sodium anticoagulant tube was used to take blood; then, it was placed on the ice for more than 15 min, was centrifuged at 3400 × g, for 3 min, plasma was separated, and was stored at −20°C to be tested. The LC–MS/MS was used to determine the plasma concentration of each animal per time in time. Samples with a concentration higher than the detection limit were diluted with blank plasma to the quantitative concentration range. Pharmacokinetic parameters were calculated with the non‐room model of Winnolin 8.2 software.

### 4.9 Statistical Analysis

Statistical analyses were carried out using GraphPad Prism software or Origin software, utilizing either a *t*‐test or one‐way analysis of variance with Tukey's test as appropriate.

## Author Contributions

Xujie Zhang, Lin Sun, Dang Ding, Shujing Xu, Fabao Zhao, and Xiangyi Jiang synthesized the compounds. Alexej Dick performed the SPR, anti‐RT, SRI assays, and CA assembly assays. Laura Walsham and David C. Goldstone performed the crystalliazion assays. Xujie Zhang, Dang Ding, and Yang Zhou performed the CANC assembly and disassembly assays. Jian Zhang, Xujie Zhang, and Zhao Wang tested the acute and subacute toxicity assays. Mei Wang performed the MD simulation. Yuexi Ma performed the early and late RT inhibition assays and antimutant assays. Chin‐Ho Chen performed the HIV‐1 NL4‐3 infection assays. Erik De Clercq and Christophe Pannecouque evaluated the anti‐HIV activity. Peng Zhan and Xinyong Liu contributed to the conceptualization and methodology. The manuscript was written through contributions of all authors. All of the authors approved the final version of the manuscript.

## Funding

This work was supported by the National Natural Science Foundation of China (NSFC No. 82504573, 82173677, 82204196), the Key Research and Development Program, Ministry of Science and Technology of the People's Republic of China (Grant No. 2023YFC2606500), the Key Project of NSFC for International Cooperation (No. 81420108027), Science Foundation for Outstanding Young Scholars of Shandong Province (ZR2020JQ31), Shandong Provincial Natural Science Foundation (ZR2022QH015), Shandong Laboratory Program (SYS202205) and NIH/NIAID grant R01AI150491 (Loll, PI).

## Ethics Statement

All animal experiments were reviewed and approved by the Laboratory Animal Ethical and Welfare Committee of Shandong University Cheeloo College of Medicine (Approval No. 21056).

## Conflicts of Interest

The authors declare no conflicts of interest.

## Supporting information




**Supporting File 1**: mco270746‐sup‐0001‐SuppMat.pdf

## Data Availability

The datasets generated and analyzed during the current study are available from the corresponding author upon reasonable request.
