# Supplementary material for: Discovery of 4‐Quinazolinone‐Containing Phenylalanine Derivatives as Potent, Resistant‐Tolerant HIV Capsid Inhibitors
Source: MedComm (2020). 2026 May 3;7(5):e70746. doi: 10.1002/mco2.70746 (PMC13136073; doi:10.1002/mco2.70746)
Supplement: Supplementary file 1 — Supporting File 1: mco270746‐sup‐0001‐SuppMat.pdf [file MCO2-7-e70746-s001.pdf]

Discovery of 4-Quinazolinone-Containing Phenylalanine Derivatives as Potent, Resistant-Tolerant HIV Capsid Inhibitors

Xujie Zhang[1], Lin Sun[1], Laura Walsham[2], Yuexi Ma[3], Dang Ding[1], Mei Wang[1], Fabao Zhao[1], Shujing Xu[1], Xiangyi Jiang[1], Yang Zhou[1], Erik De Clercq[4], Christophe Pannecouque[4], Chin-Ho Chen[5], David C. Goldstone[2,*], Xinyong Liu[1,*], Alexej Dick[6,*], Peng Zhan[1,*]

[1] Department of Medicinal Chemistry, Key Laboratory of Chemical Biology (Ministry of Education), School of Pharmaceutical Sciences, Shandong University, 44 West Culture Road, 250012 Jinan, Shandong, PR China.

[2] School of Biological Sciences, University of Auckland, Auckland, 1010, New Zealand

[3] Department of Microbiology and Infectious Disease Research Center, School of Basic Medical Sciences, Peking University Health Science Center, Beijing, 100191, PR China.
[4] Rega Institute for Medical Research, Laboratory of Virology and Chemotherapy, K.U. Leuven, Herestraat 49 Postbus 1043 (09.A097), 3000, Leuven, Belgium

[5] Surgical Oncology Research Facility, Duke University Medical Center, Durham, NC 27710, Box 2926, USA.

[6] Department of Biochemistry & Molecular Biology, Drexel University College of Medicine, Philadelphia, Pennsylvania, PA 19102, USA.

*Corresponding authors. E-mail address: zhanpeng1982@sdu.edu.cn (Zhan P.); ad3474@drexel.edu (Dick A.); xinyongl@sdu.edu.cn (Liu X.Y.); d.goldstone@auckland.ac.nz (Goldstone D).

Content

SI. Synthesis steps and compounds characterization..3
SII. ^{1}H NMR, ^{13}C NMR, ^{19}F NMR and HRMS of representative compounds 39
SIII. SPR assays...73
SIV. The effects of target compounds for assembly of capsid *in vitro* 80
SV. The effects of target compounds for CANC Disassembly 81
SVI. The effects of target compounds for CANC Assembly.......................................82
SVII. Data collection and refinement statistics in crystallisation research..................83
SVIII. 2D View of Binding mode for IC-2i..84
SIX. Results for MD simulation 85
SX. Stability in Human Liver Microsomes...86
SXI. Stability in Human Plasma 90
SXII. *In Vivo* Pharmacokinetics Study..93
References..94

SI. Synthesis steps and compounds characterization

^{1}H NMR, ^{13}C NMR and ^{19}F NMR spectra were recorded on Bruker AV-400 spectrometer or Bruker AV-600 spectrometer using solvents as indicated (DMSO-d_6 *or* Chloroform-*d*). Chemical shifts were reported in δ values (ppm) with tetramethylsilane (TMS) as the internal reference, and *J* values were reported in hertz (Hz). Melting points (mp) were determined on a micromelting point apparatus and were uncorrected. TLC was performed on Silica Gel GF254 for TLC (Merck), and spots were visualized by iodine vapor or irradiation with UV light (λ = 254 nm). Flash column chromatography was performed on a column packed with Silica Gel60 (200-300 mesh). Solvents were of reagent grade and were purified and dried by standard methods when necessary. The concentration of the reaction solutions involved the use of a rotary evaporator at reduced pressure. The solvents of dichloromethane, Et_3N and methanol *etc.,* were obtained from Sinopharm Chemical Reagent Co., Ltd (SCRC), which were of AR grade. The key reactants were purchased from Bide Pharmatech Co., Ltd or Shanghai Haohong Scientific Co., Ltd. The purity of target compounds was evaluated on Shimadzu LC-20A. HPLC conditions were as follows: Agilent ZORBAX, SB-C18column (250 mm × 4.6 mm × 5 um); isocratic elution method:mobile phase A: methanol (80%); mobile phase B: water (20%); flowrate: 1.0 mL/min; wavelength: 254 nm, temperature: 30 °C, injectionvolume: 10 uL. Elemental analyses (C, H, N, S) were performed on Elementar UNICUBE (Elementar Analysensysteme GmbH).

S1.1 4-chloro-7-nitro-1*H*-indazol-3-amine (a-2)

Dissolve 2,6-dichlori-3-nitrobenal (10.00 g, 46.08 mmol, 1.0 eq.) with 400 mL ethanol, and slowly add 50% hydrophilicon (13.84 g, 138.25 mmol, 3.0 eq.). Then the mixture was stirred at room temperature for about 3 h (monitored by TLC). Add 300 mL water to the reaction bottle when the reaction was over and stir for 1 hour. Then filter and wash the filter cake with cold ethanol, orthopatane: acetone (1: 1) and water. Dry the cake in the vacuum to get crude product **a-2**. Red solid, yield 86%. ^{1}H NMR (600 MHz, DMSO-d_6) δ 12.71 (s, 1H, indazole-H), 8.20 (d, *J* = 8.4 Hz, 1H, indazole-H), 7.12 (d, *J* = 8.5 Hz, 1H, indazole-H), 5.66 (s, 2H, indazole-NH_2). MS: m/z 212.90 $(M+H)^+$. $C_7H_5ClN_4O_2$ [212.01].

S1.2 4-chloro-7-nitro-1-(2,2,2-trifluoroethyl)-1*H*-indazol-3-amine (a-3)

Under the ice bath, dissolve the intermediate **a-2** (8.40 g, 39.51 mmol) with 80 mL *N*,*N*-dimethylformamide (DMF), and then slowly add Cs_2CO_3 (25.75 g, 79.02 mmol). Then slow drip 2,2,2-trifluoroethyl trifluoromethanesulfonate (10.09 g, 43.46 mmol) and remove ice when completing. The mixture was stirred for 8 hour at room temperature (monitored by TLC). Slowly add cold water to the mixture and stir for 8 h. Then filter and wash the filter cake with hexane and water. Dry the cake in the vacuum to get crude product **a-3**. Red solid, yield 96%. ^1H NMR (400 MHz, DMSO-d_6) δ 8.20 (d, J = 8.4 Hz, 1H, indazole-H), 7.30 (d, J = 8.5 Hz, 1H, indazole-H), 6.02 (s, 2H, indazole-NH_2), S19 (q, J = 9.0 Hz, 2H, CF_3CH_2). MS: m/z 318.18 $(M + Na)^+$. $C_9H_6ClF_3N_4O_2$ [294.62].

S1.3 *N*-(4-chloro-7-nitro-1-(2,2,2-trifluoroethyl)-1*H*-indazol-3-yl)-*N*-(ethylsulfonyl)ethanesulfonamide (a-4)

Under the ice bath, the intermediate **a-3** (8.80 g, 29.87 mmol) was dissolved in 200 ml of dichloromethane (DCM). Subsequently, triethylamine (45.34 g, 448.04 mmol) and 4-dimethylaminopyridine (DMAP) (0.73 g, 5.97 mmol) were added sequentially. Then, ethyl sulfonic chloride (23.04 g,179.21 mmol) was slowly added dropwise, and the mixture was stirred for 1 hour under the ice bath while monitoring the reaction progress using TLC. Once the reaction was complete, 200 mL of saturated sodium chloride solution was added to the reaction flask. After stirring for 15 minutes, the mixture was transferred to a separating funnel and the upper layer was washed with dichloromethane. After that, the organic layer was dried with sodium sulfate and the volume of the filtrate was reduced under reduced pressure to remove the organic solvent. Finally, the residue was crystallized using methanol to obtain the intermediate **a-4**. Yellow solid, yield 85%. ^1H NMR (600 MHz, DMSO-d_6) δ 8.42 (d, J = 8.4 Hz, 1H, indazole-H), 7.72 (d, J = 8.4 Hz, 1H, indazole-H), 5.64 (q, J = 8.8 Hz, 2H, CF_3CH_2), 3.91 (dq, J = 14.8, 7.4 Hz, 2H, SO_2CH_2), 3.82 (dq, J = 14.7, 7.4 Hz, 2H, SO_2CH_2), 1.40 (t, J = 7.4 Hz, 6H, $CH_3 \times 2$). MS: m/z 478.62 $(M+H)^+$. $C_{13}H_{14}ClF_3N_4O_6S_2$ [478.00].

S1.4 *N*-(7-amino-4-chloro-1-(2,2,2-trifluoroethyl)-1*H*-indazol-3-yl)-*N*-(ethylsulfonyl)ethanesulfonamide (a-5)

Dissolve NH_4Cl (13.40 g, 250.60 mmol) and zinc powder (16.38 g, 250.60 mmol) in 240 mL of water and 120 mL of tetrahydrofuran (THF), and stir the mixture for 15 minutes. Next, dissolve the intermediate **a-4** (12.00 g, 25.06 mmol) in 240 mL of THF and slowly add it to the reaction flask. The mixture was stirred at room temperature for 24 hours, with the reaction progress monitored using TLC. Once the reaction was complete, add 120 mL of water and 240 mL of ethyl acetate to the reaction flask. After stirring for 15 minutes, filter the mixture and wash the filtrate with 240 mL of saturated NaCl solution. Separate the organic phase and concentrate it under vacuum. Finally, crystallize the residue using ethyl acetate and petroleum ether to obtain the intermediate **a-5**. Yellow solid, yield 91%. ^1H NMR (400 MHz, DMSO-d_6) δ 7.11 (d, J = 8.0 Hz, 1H, indazole-H), 6.77 (d, J = 8.1 Hz, 1H, indazole-H), 5.63 (q, J = 8.6 Hz, 2H, CF_3CH_2), 5.58 (s, 2H, indazole-NH_2), 3.86 (dq, J = 14.9, 7.5 Hz, 2H, SO_2CH_2), 3.74 (dq, J = 14.6, 7.4 Hz, 2H, SO_2CH_2), 1.38 (t, J = 7.4 Hz, 6H, CH_3×2). MS: m/z 470.70 $(M + Na)^+$. $C_{13}H_{16}ClF_3N_4O_4S_2$ [448.03].

S1.5 *tert*-butyl (*S*)-(1-(7-bromo-3-(4-chloro-3-(*N*-(ethylsulfonyl)ethylsulfonamido)-1-(2,2,2-trifluoroethyl)-1*H*-indazol-7-yl)-4-oxo-3,4-dihydroquinazolin-2-yl)-2-(3,5-difluorophenyl)ethyl)carbamate (a-6)

Dissolve the intermediate **a-5** (8.00 g, 17.82 mmol), *N*-Boc-*L*-3,5-difluorophenylalanine (5.37 g, 17.82 mmol), 2-amino-4-bromobenzoic acid (3.85 g, 17.82 mmol), and diphenyl phosphite (16.70 g, 71.29 mmol) in pyridine. Stir the mixture at 75 °C for 24 hours while monitoring the progress using TLC. Once the reaction was complete, add 200 mL of ethyl acetate and 300 mL of 0.5 M citric acid solution to the mixture. Stir the mixture for 15 minutes and then transfer it to a separating funnel to separate the organic phase. Wash the organic phase with saturated NaCl solution. The organic phase can be further purified using fast column chromatography. The obtained crude product was crystallized using a mixed solvent of ethyl acetate and petroleum ether to obtain the intermediate **a-6**. White solid, yield 26%. ^1H NMR (400 MHz, DMSO-d_6) δ 8.10 (d, J = 8.5 Hz, 1H, NH), 7.98 (d, J = 8.7 Hz,

2H, quinazolinone-H), 7.83 (d, J = 8.4 Hz, 1H, quinazolinone-H), 7.76 (d, J = 8.0 Hz, 1H, indazole-H), 7.65 (d, J = 8.1 Hz, 1H, indazole-H), 7.01 (t, J = 9.5 Hz, 1H, Ph-H), 6.53 (d, J = 7.7 Hz, 2H, Ph-H), 5.05 (dq, J = 17.2, 8.7 Hz, 1H, CF_3CH_2), 4.71 (dq, J = 16.8, 8.6 Hz, 1H, CF_3CH_2), 4.10 (t, J = 9.2 Hz, 1H, CH), 3.95 – 3.71 (m, 4H, SO_2CH_2×2), 3.23 (d, J = 13.8 Hz, 1H, Ph-CH_2), 2.95 (dd, J = 14.0, 10.8 Hz, 1H, Ph-CH_2), 1.39 (q, J = 7.5 Hz, 6H, CH_3×2), 1.21 (s, 9H, $C(CH_3)_3$). MS: m/z 912.15 $(M+H)^+$, 934.54 $(M + Na)^+$. $C_{34}H_{33}BrClF_5N_6O_7S_2$ [910.06].

S1.6 (*S*)-*N*-(7-(2-(1-amino-2-(3,5-difluorophenyl)ethyl)-7-bromo-4-oxoquinazolin-3(4*H*)-yl)-4-chloro-1-(2,2,2-trifluoroethyl)-1*H*-indazol-3-yl)-*N*-(ethylsulfonyl)ethanesulfonamide (a-7)

Trifluoroacetic acid (5.0 eq.) was added dropwise to the corresponding substituted intermediate **a-6** (1.0 eq.) in 30 mL DCM and stirred at room temperature for 1 h (monitored by TLC). Then, the resulting mixture solution was alkalized to pH ~7 with saturated sodium bicarbonate solution and then extracted with dichloromethane (3 × 30 mL), dried over anhydrous Na_2SO_4, filtered, and concentrated under reduced pressure to afford corresponding crude product **a-7**. Then the crude was crystallized using a mixed solvent of ethyl acetate and petroleum ether to obtain the intermediate **a-7**. White solid, yield 96%. ^1H NMR (600 MHz, DMSO-d_6) δ 8.09 – 8.05 (m, 2H, quinazolinone-H, indazole-H), 7.81 (dd, J = 8.4, 2.0 Hz, 1H, quinazolinone-H), 7.65 (s, 2H, quinazolinone-H, indazole-H), 6.99 (tt, J = 9.4, 2.4 Hz, 1H, Ph-H), 6.71 (d, J = 7.7 Hz, 2H, Ph-H), S17 (dq, J = 17.4, 8.8 Hz, 1H, CF_3CH_2), 4.77 (dq, J = 16.9, 8.6 Hz, 1H, CF_3CH_2), 3.95 – 3.75 (m, 4H, SO_2CH_2×2), 3.53 (s, 1H, CH), 3.26 (dd, J = 13.5, 4.9 Hz, 1H, Ph-CH_2), 2.84 (dd, J = 13.5, 8.1 Hz, 1H, Ph-CH_2), 1.40 (td, J = 7.4, 1.4 Hz, 6H, CH_3×2). MS: m/z 812.61 $(M+H)^+$, 834.45 $(M + Na)^+$. $C_{29}H_{25}BrClF_5N_6O_5S_2$ [810.01].

S1.7 4-((4-nitrophenyl)sulfonyl)piperazin-2-one (b-2)

Under ice bath, dissolve piperazin-2-one (1.00 g, 10.00 mmol) in dichloromethane (DCM) and then add Et_3N (2.02 g, 20.01 mmol). Dissolve the intermediate **b-1** (2.66 g, 12.00 mmol) in DCM and slowly add it to the mixture. Then, remove the ice bath and

stir the mixture for approximately 2 hours while monitoring the reaction using TLC. Once the reaction was complete, perform a filtration under reduced pressure, wash the filter cake with DCM, and finally obtain the intermediate **b-2** after drying. White solid, yield 96%. ^{1}H NMR (600 MHz, DMSO-d_6) δ 8.45 – 8.40 (m, 2H, Ph-H), 8.09 – 8.06 (m, 3H, Ph-H, NH), 3.59 (s, 2H, piperazone), 3.28 (dd, J = 6.5, 4.3 Hz, 2H, piperazone), 3.19 (td, J = 5.4, 4.8, 2.5 Hz, 2H, piperazone). MS: m/z 285.81 $(M+H)^+$. $C_{10}H_{14}N_3O_5S$ [285.04].

S1.8 methyl 2-(4-((4-nitrophenyl)sulfonyl)-2-oxopiperazin-1-yl)acetate (b-3)

Under ice bath, dissolve the intermediate **b-2** (2.70 g, 9.46 mmol) in THF. Next, slowly add NaH (0.68 g, 28.39 mmol) and stir the mixture in the ice bath for 30 minutes. Then, slowly add methyl bromoacetate (1.74 g, 11.36 mmol) and transfer the reaction to room temperature. Stir the mixture for 2 hours while monitoring the reaction using TLC. Once the reaction was complete, perform filtration under reduced pressure and sequentially wash the filter cake with ethyl acetate and water. Finally, dry the filter cake to obtain the intermediate **b-3**. White solid, yield 93%. ^{1}H NMR (400 MHz, DMSO-d_6) δ 8.45 (d, J = 8.8 Hz, 2H, Ph-H), 8.11 (d, J = 8.9 Hz, 2H, Ph-H), 4.08 (s, 2H), 3.75 (s, 2H, piperazone), 3.62 (s, 3H, OCH_3), 3.42 (s, 4H, piperazone). MS: m/z 357.83 $(M+H)^+$, 379.94 $(M + Na)^+$. $C_{13}H_{15}N_3O_7S$ [357.06].

S1.9 2-(4-((4-nitrophenyl)sulfonyl)-2-oxopiperazin-1-yl)acetic acid (b-4)

Dissolve the intermediate **b-3** (2.00 g, 5.60 mmol) in a mixture of 20 mL THF and 20 mL water. Slowly add LiOH (0.40 g, 16.79 mmol) and stir the mixture at room temperature. Once the reaction was complete, remove THF under vacuum and adjust the remaining solution to pH 2-3 by adding 1 M HCl. Then perform filtration under reduced pressure and wash the filter cake with water. Finally, dry the filter cake to obtain the intermediate **b-4**. White solid, yield 92%. ^{1}H NMR (600 MHz, DMSO-d_6) δ 12.77 (s, 1H, COOH), 8.44 – 8.40 (m, 2H, Ph-H), 8.10 – 8.06 (m, 2H, Ph-H), 3.95 (s, 2H, $COCH_2$), 3.72 (s, 2H, piperazone), 3.40 (dd, J = 7.0, 4.4 Hz, 2H, piperazone), 3.37

(dd, *J* = 6.9, 4.6 Hz, 2H, piperazone). MS: m/z 343.98 $(M+H)^+$, 366.01 $(M + Na)^+$. $C_{13}H_{15}N_3O_7S$ [343.31].

S1.10 2-(4-((4-aminophenyl)sulfonyl)-2-oxopiperazin-1-yl)acetic acid (c-1)

Mix zinc powder (1.83 g, 27.98 mmol) with 10 mL THF and 5 mL water, and add 1 M HCl to adjust the pH to 1-2. Next, dissolve intermediate **b-3** (1.00 g, 2.80 mmol) in 5 mL THF and add it to the mixture. Raise the temperature to 75 °C and stir for 24 hours, monitoring the progress using TLC. Once the reaction was complete, remove most of the organic solvents under vacuum. Then, adjust the pH using 1 M NaOH until amount of solid appeared. Filter the mixture and wash the cake with water. Finally, dry the filter cake to obtain the intermediate **c-1**. Yellow solid, yield 27%. MS: m/z 313.92 $(M+H)^+$, 336.09 $(M + Na)^+$, 351.93 $(M + K)^+$. $C_{12}H_{13}N_3O_7S$ [313.07].

S1.11 (*S*)-*N*-(1-(7-bromo-3-(4-chloro-3-(*N*-(ethylsulfonyl)ethylsulfonamido)-1-(2,2,2-trifluoroethyl)-1*H*-indazol-7-yl)-4-oxo-3,4-dihydroquinazolin-2-yl)-2-(3,5-difluorophenyl)ethyl)-2-(4-((4-nitrophenyl)sulfonyl)-2-oxopiperazin-1-yl)acetamide (IA-1)

Under ice bath, intermediate **b-3** (101.5 mg, 295.6 μmol) and HATU (140.5 mg, 369.5 μmol) were mixed in 15 mL DCM and stirred for 0.5 h. Then, the intermediate **a-7** (200.0 mg, 246.3 μmol) and DIEA (63.7 mg, 492.6 μmol) were slowly added to the mixture at 0 ºC. The reaction system was then stirred at room temperature for an 2 h (monitored by TLC). Once the reaction was complete, the mixture was initially washed with saturated NaCl and extracted with DCM (3 × 20 mL), and dried over anhydrous Na_2SO_4, filtered, and concentrated under reduced pressure to afford a corresponding crude product, purified by flash column chromatography to afford the crude. Finally, the crude was crystallized using a mixed solvent of ethyl acetate and petroleum ether to obtain the target compound **IA-1**. White solid, yield 70%. m.p. 147 – 150℃. ^1H NMR (400 MHz, DMSO-d_6) δ 8.71 (d, *J* = 8.2 Hz, 1H, NH), 8.44 (d, *J* = 8.5 Hz, 2H, Ph-H), 8.09 (dd, *J* = 8.6, 4.0 Hz, 3H, quinazolinone-H, Ph-H), 8.02 (d, *J* = 1.9 Hz, 1H, quinazolinone-H), 7.84 (dd, *J* = 8.3, 2.0 Hz, 2H, quinazolinone-H, indazole-H), 7.71 (d,

J = 8.0 Hz, 1H, indazole-H), 7.00 (tt, *J* = 9.5, 2.3 Hz, 1H, Ph-H), 6.61 (d, *J* = 6.9 Hz, 2H, Ph-H), S11 (dq, *J* = 17.3, 8.8 Hz, 1H, CF_3CH_2), 4.57 (td, *J* = 9.0, 3.7 Hz, 1H, CH), 4.39 (dt, *J* = 16.6, 8.3 Hz, 1H, CF_3CH_2), 3.81 (ttd, *J* = 28.4, 14.3, 7.3 Hz, 6H, $COCH_2$, $SO_2CH_2{\times}2$), 3.69 – 3.56 (m, 2H, piperazone), 3.44 (d, *J* = 16.5 Hz, 1H, Ph-CH_2), 3.29 (dd, *J* = 13.4, 6.5 Hz, 2H, piperazone), 3.25 – 3.14 (m, 2H, piperazone), 2.97 (dd, *J* = 14.1, 10.0 Hz, 1H, Ph-CH_2), 1.37 (dt, *J* = 1S1, 7.4 Hz, 6H, $CH_3{\times}2$). ^{13}C NMR (100 MHz, DMSO-d_6) δ 168.03 (C=O), 163.72 (C=O), 162.34 (d, $^1J_{CF}$ = 228.0 Hz), 161.37, 157.48, 150.77, 147.82, 142.09 (t, $^3J_{CF}$ = 9.8 Hz), 140.81, 138.81, 135.98, 132.35, 131.66, 129.99, 129.73, 129.20, 127.16, 125.31, 12S15, 120.97, 119.96, 119.24, 112.60 (dd, $^2J_{CF}$ = 18.1, $^4J_{CF}$ = 6.9 Hz), 102.74 (t, $^2J_{CF}$ = 26.0 Hz), 52.72, 50.91, 50.83, 48.64, 47.07, 42.97, 37.91, 7.84, 7.77. ^{19}F NMR (376 MHz, DMSO-d_6) δ -68.20 (t, *J* = 8.7 Hz, indazole-CH_2CF_3), -110.10 (t, *J* = 8.5 Hz, Ph-F × 2). HRMS found m/z 1136.0560 $[M+H]^+$, calculated for $C_{41}H_{36}BrClF_5N_9O_{11}S_3$, $[M+H]^+$: 1136.0561. HPLC purity: 98.59 %.

S1.12 (*S*)-*N*-(1-(7-bromo-3-(4-chloro-3-(ethylsulfonamido)-1-(2,2,2-trifluoroethyl)-1*H*-indazol-7-yl)-4-oxo-3,4-dihydroquinazolin-2-yl)-2-(3,5-difluorophenyl)ethyl)-2-(4-((4-nitrophenyl)sulfonyl)-2-oxopiperazin-1-yl) acetamide (IA-2)

Dissolve **IA-1** (100.00 mg, 87.93 μmol) and K_2CO_3 (36.46 mg, 263.78 μmol) in 10 mL DMF and 1 mL water. Then raise the temperature to 75 °C and stir for 6 h (monitored by TLC). Once the reaction was complete, add 20 mL of ethyl acetate and 30 mL of saturated NaCl to the mixture. Next, separate the organic phase and wash the aqueous phase with ethyl acetate. Combine the organic phases, dry them with sodium sulfate, and filter. Remove the organic solvents under reduced pressure and the residue was purified by column chromatography. Finally, crystallize the product from a hybrid solvent of ethyl acetate and petroleum ether to obtain the target compound **IA-2**. White solid, yield 68%. m.p. 146 – 149°C. ^1H NMR (600 MHz, DMSO-d_6) δ 10.06 (s, 1H, SO_2NH), 8.75 (d, *J* = 8.4 Hz, 1H, NH), 8.44 – 8.41 (m, 2H, Ph-H), 8.09 (d, *J* = 8.5 Hz,

1H, quinazolinone-H), 8.08 – 8.05 (m, 2H, Ph-H), 8.00 (d, *J* = 1.9 Hz, 1H, quinazolinone-H), 7.83 (dd, *J* = 8.4, 1.9 Hz, 1H, quinazolinone-H), 7.74 (d, *J* = 8.0 Hz, 1H, indazole-H), 7.55 (d, *J* = 8.0 Hz, 1H, indazole-H), 6.99 (tt, *J* = 9.4, 2.4 Hz, 1H, Ph-H), 6.63 – 6.58 (m, 2H, Ph-H), 4.90 (dq, *J* = 16.9, 8.6 Hz, 1H, CF_3CH_2), 4.56 – 4.51 (m, 1H, CH), 4.22 (dq, *J* = 16.9, 8.6 Hz, 1H, CF_3CH_2), 3.83 (d, *J* = 16.5 Hz, 1H, $COCH_2$), 3.66 – 3.57 (m, 2H, $COCH_2$), 3.51 (d, *J* = 16.5 Hz, 1H, $COCH_2$), 3.35 (td, *J* = 12.7, 11.2, 7.3 Hz, 3H, piperazone, Ph-CH_2), 3.26 (q, *J* = 6.1 Hz, 2H, piperazone), 3.09 (ddt, *J* = 33.6, 11.7, 5.4 Hz, 2H, piperazone), 2.94 (dd, *J* = 14.2, 10.0 Hz, 1H, Ph-CH_2), 1.33 (t, *J* = 7.3 Hz, 3H, CH_3). ^{13}C NMR (150 MHz, DMSO-d_6) δ 167.83 (C=O), 163.62 (C=O), 162.53 (dd, $^1J_{CF}$ = 246.1, $^3J_{CF}$ = 13.5 Hz), 161.23, (C=O) 157.85, 150.79, 147.94, 142.04 (t, $^3J_{CF}$ = 9.7 Hz), 141.14, 138.39, 131.79, 131.56, 129.94, 129.65, 129.58, 129.21, 128.35, 125.26, 123.49, 123.28 (d, $^1J_{CF}$ = 280.9 Hz), 120.05, 119.40, 118.57, 112.53 (dd, $^2J_{CF}$ = 19.7, $^4J_{CF}$ = 4.9 Hz), 102.68 (t, $^2J_{CF}$ = 25.8 Hz), 52.57, 50.82, 48.59, 48.53, 48.14, 47.11, 43.02, 38.18, 8.42. HRMS found m/z 1144.0632 $[M+H]^+$, calculated for $C_{39}H_{32}BrClF_5N_9O_9S_3$, $[M+H]^+$: 1144.0629. HPLC purity: 99.53 %.

S1.13 (*S*)-2-(4-((4-aminophenyl)sulfonyl)-2-oxopiperazin-1-yl)-*N*-(1-(7-bromo-3-(4-chloro-3-(*N*-(ethylsulfonyl)ethylsulfonamido)-1-(2,2,2-trifluoroethyl)-1*H*-indazol-7-yl)-4-oxo-3,4-dihydroquinazolin-2-yl)-2-(3,5-difluorophenyl)ethyl) acetamide (IA-3)

Under ice bath, intermediate **c-1** (46.3 mg, 147.78 μmol) and HATU (70.2 mg, 184.7 μmol) were mixed in 15 mL DCM and stirred for 0.5 h. Then, the intermediate **a-7** (100.0 mg, 123.15 μmol) and DIEA (31.83 mg, 246.30 μmol) were slowly added to the mixture at 0 ºC. The reaction system was then stirred at room temperature for an 2 h (monitored by TLC). Once the reaction was complete, the mixture was initially washed with saturated NaCl and extracted with DCM (3 × 20 mL), and dried over anhydrous Na_2SO_4, filtered, and concentrated under reduced pressure to afford a corresponding crude product, purified by flash column chromatography to afford the crude. Finally, the crude was crystallized using a mixed solvent of ethyl acetate and petroleum ether

to obtain the target compound **IA-3**. White solid, yield 34%. m.p. 188 – 191°C. ^{1}H NMR (600 MHz, DMSO-d_6) δ 8.61 (d, J = 8.4 Hz, 1H, NH), 8.08 (d, J = 8.5 Hz, 1H, quinazolinone-H), 8.02 (d, J = 1.9 Hz, 1H, quinazolinone-H), 7.86 – 7.80 (m, 2H, quinazolinone-H, indazole-H), 7.68 (d, J = 8.0 Hz, 1H, indazole-H), 7.40 (d, J = 8.7 Hz, 2H, Ph-H), 6.98 (tt, J = 9.3, 2.3 Hz, 1H, Ph-H), 6.68 (d, J = 8.6 Hz, 2H, Ph-H), 6.63 – 6.58 (m, 2H, Ph-H), 6.16 (s, 2H, NH_2), S12 (dq, J = 17.2, 8.7 Hz, 1H, CF_3CH_2), 4.64 (td, J = 9.0, 4.0 Hz, 1H, CH), 4.40 (dq, J = 16.7, 8.4 Hz, 1H, CF_3CH_2), 3.92 – 3.69 (m, 6H, $COCH_2$, SO_2CH_2×2), 3.65 – 3.58 (m, 2H, piperazone), 3.14 (dtt, J = 16.5, 9.0, 3.7 Hz, 3H, piperazone, Ph-CH_2), 3.01 – 2.91 (m, 3H, piperazone, Ph-CH_2), 1.39 (t, J = 7.4 Hz, 3H, CH_3), 1.35 (t, J = 7.4 Hz, 3H, CH_3). ^{13}C NMR (150 MHz, DMSO-d_6) δ 168.02 (C=O), 164.35 (C=O), 162.51 (dd, $^1J_{CF}$ = 246.1, $^3J_{CF}$ = 13.5 Hz), 161.36 (C=O), 157.37, 154.19, 147.82, 142.11 (t, $^3J_{CF}$ = 9.0 Hz), 138.87, 136.09, 132.30, 131.65, 130.26, 130.22, 130.03, 129.65, 129.17, 127.19, 12S12, 123.11 (d, $^1J_{CF}$ = 281.1 Hz), 121.02, 119.97, 119.23, 118.83, 113.39, 112.63 (dd, $^2J_{CF}$ = 19.8, $^4J_{CF}$ = 4.7 Hz), 102.66 (t, $^2J_{CF}$ = 26.2 Hz), 54.16, 50.78, 49.22, 43.18, 42.36, 38.71, 18.59, 17.24, 12.89, 7.84, 7.78. HRMS found m/z 1106.0823 $[M+H]^+$, calculated for $C_{41}H_{38}BrClF_5N_9O_9S_3$, $[M+H]^+$: 1106.0819. HPLC purity: 89.88 %.

S1.14 (*S*)-*N*-(1-(7-bromo-3-(4-chloro-3-(*N*-(ethylsulfonyl)ethylsulfonamido)-1-(2,2,2-trifluoroethyl)-1*H*-indazol-7-yl)-4-oxo-3,4-dihydroquinazolin-2-yl)-2-(3,5-difluorophenyl)ethyl)-2-(5-methyl-3-(trifluoromethyl)-1*H*-pyrazol-1-yl)acetamide (IB-1a)

Under ice bath, intermediate 2-[5-methyl-3-(trifluoromethyl)pyrazol-1-yl]acetic acid (307.6 mg, 1.48 mmol) and HATU (702.4 mg, 1.85 mmol) were mixed in 30 mL DCM and stirred for 0.5 h. Then, the intermediate **a-7** (1.00 g, 1.23 mmol) and DIEA (318.3 mg, 2.46 mmol) were slowly added to the mixture at 0 ºC. The reaction system was then stirred at room temperature for an 2 h (monitored by TLC). Once the reaction was complete, the mixture was initially washed with saturated NaCl and extracted with DCM (3 × 20 mL), and dried over anhydrous Na_2SO_4, filtered, and concentrated under

reduced pressure to afford a corresponding crude product, purified by flash column chromatography to afford the crude. Finally, the crude was crystallized using a mixed solvent of ethyl acetate and petroleum ether to obtain the target compound **IB-1a**. White solid, yield 79%. m.p. 97 – 99°C. ^{1}H NMR (600 MHz, DMSO-d_6) δ 9.08 (d, J = 8.0 Hz, 1H, NH), 8.10 (d, J = 8.5 Hz, 1H, quinazolinone-H), 8.01 (d, J = 1.9 Hz, 1H, quinazolinone-H), 7.86 (d, J = 7.9 Hz, 1H, indazole-H), 7.85 (d, J = 8.5 Hz, 1H, quinazolinone-H), 7.72 (d, J = 8.0 Hz, 1H, indazole-H), 7.02 (tt, J = 9.3, 2.3 Hz, 1H, Ph-H), 6.63 – 6.58 (m, 2H, Ph-H), 6.44 (s, 1H, pyrazole-H), 4.90 (dq, J = 17.1, 8.6 Hz, 1H, CF_3CH_2), 4.72 – 4.56 (m, 2H, $COCH_2$), 4.53 (ddd, J = 10.0, 8.0, 3.7 Hz, 1H, CH), 4.39 (dq, J = 16.8, 8.5 Hz, 1H, CF_3CH_2), 3.90 – 3.67 (m, 4H, $SO_2CH_2\times2$), 3.35 (dd, J = 14.3, 3.7 Hz, 1H, Ph-CH_2), 3.00 (dd, J = 14.2, 10.0 Hz, 1H, Ph-CH_2), 2.05 (s, 3H, pyrazole-CH_3), 1.39 (t, J = 7.4 Hz, 3H, CH_3), 1.32 (t, J = 7.4 Hz, 3H, CH_3). ^{13}C NMR (150 MHz, DMSO-d_6) δ 166.66 (C=O), 162.61 (dd, $^1J_{CF}$ = 246.1, $^3J_{CF}$ = 13.9 Hz), 161.31 (C=O), 157.49, 147.87, 142.63, 141.80 (t, $^3J_{CF}$ = 9.0 Hz), 138.79, 136.11, 132.31, 131.66, 129.98, 129.68, 129.22, 127.36, 125.09, 122.99 (d, $^1J_{CF}$ = 280.3 Hz), 121.97 (d, $^1J_{CF}$ = 268.6 Hz), 121.02, 119.96, 119.18, 112.54 (dd, $^2J_{CF}$ = 19.9, $^4J_{CF}$ = 4.8 Hz), 104.14, 102.82 (t, $^2J_{CF}$ = 25.3 Hz), 52.96, 52.05, 51.01, 50.63, 38.71, 38.09, 10.74, 7.83, 7.66. HRMS found m/z 1001.0549 $[M+H]^+$, calculated for $C_{36}H_{30}BrClF_8N_8O_6S_2$, $[M+H]^+$: 1001.0547. HPLC purity: 99.13 %.

S1.15 (*S*)-*N*-(1-(7-bromo-3-(4-chloro-3-(ethylsulfonamido)-1-(2,2,2-trifluoroethyl)-1*H*-indazol-7-yl)-4-oxo-3,4-dihydroquinazolin-2-yl)-2-(3,5-difluorophenyl)ethyl)-2-(5-methyl-3-(trifluoromethyl)-1*H*-pyrazol-1-yl)acetamide (IB-2a)

Dissolve **IB-1a** (100.0 mg, 99.79 μmol) and K_2CO_3 (41.4 mg, 299.36 μmol) in 10 mL DMF and 1 mL water. Then raise the temperature to 75 °C and stir for 6 h (monitored by TLC). Once the reaction was complete, add 20 mL of ethyl acetate and 30 mL of saturated NaCl to the mixture. Next, separate the organic phase and wash the aqueous phase with ethyl acetate. Combine the organic phases, dry them with sodium sulfate,

and filter. Remove the organic solvents under reduced pressure and the residue was purified by column chromatography. Finally, crystallize the product from a hybrid solvent of ethyl acetate and petroleum ether to obtain the target compound **IB-2a**. White solid, yield 82%. m.p. 95 – 97°C. ^{1}H NMR (600 MHz, Chloroform-*d*) δ 8.13 (d, J = 8.4 Hz, 1H, quinazolinone-H), 7.93 (d, J = 1.9 Hz, 1H, quinazolinone-H), 7.71 (dd, J = 8.4, 1.9 Hz, 1H, quinazolinone-H), 7.47 (s, 1H, SO_2NH), 7.28 (s, 1H, NH), 6.98 (d, J = 8.2 Hz, 1H, indazole-H), 6.90 (d, J = 7.9 Hz, 1H, indazole-H), 6.67 (tt, J = 8.8, 2.3 Hz, 1H, Ph-H), 6.38 (s, 1H, pyrazole-H), 6.30 – 6.23 (m, 2H, Ph-H), 4.64 – 4.53 (m, 3H, $COCH_2$, CH), 4.47 (dq, J = 16.2, 8.1 Hz, 1H, CF_3CH_2), 4.24 (dq, J = 16.3, 8.1 Hz, 1H, CF_3CH_2), 3.58 (ddp, J = 21.8, 14.6, 7.3 Hz, 2H, SO_2CH_2), 3.14 (dd, J = 13.9, 5.6 Hz, 1H, Ph-CH_2), 2.79 (dd, J = 13.9, 8.1 Hz, 1H, Ph-CH_2), 2.18 (s, 3H, pyrazole-CH_3), 1.48 (t, J = 7.4 Hz, 3H, CH_3). ^{13}C NMR (150 MHz, Chloroform-*d*) δ 165.13 (C=O), 162.93 (dd, $^1J_{CF}$ = 250.6, $^3J_{CF}$ = 12.9 Hz), 161.07 (C=O), 155.94, 147.07, 143.26 (d, $^2J_{CF}$ = 38.9 Hz), 141.82, 140.89, 138.69 (t, $^3J_{CF}$ = 8.9 Hz), 137.93, 131.98, 130.94, 130.83, 130.54, 128.97, 128.77, 123.38, 121.98, 120.80 (d, $^1J_{CF}$ = 214.8 Hz), 118.92, 117.27, 116.53, 112.05 (dd, $^2J_{CF}$ = 19.8, $^4J_{CF}$ = 4.9 Hz), 104.81, 103.12 (t, $^2J_{CF}$ = 25.2 Hz), 52.09, 51.66, 51.26 (d, $^2J_{CF}$ = 36.0 Hz), 47.96, 40.36, 10.84, 8.10. HRMS found m/z 909.0613 $[M+H]^+$, calculated for $C_{34}H_{26}BrClF_8N_8O_4S$, $[M+H]^+$: 909.0615. HPLC purity: 99.25 %.

S1.16 General procedure for the synthesis of IB-1(b – d)

Dissolve **IB-1a** (1.0 eq.), different substituted phenylboric acid or pinacol ester of phenylboric acid (1.2 equiv), K_3PO_4 (3.0 eq.) and $Pd(PPh_3)_4$ (0.2 eq.) in 10 mL toluene and 2 mL water. Replace the air with nitrogen and increase the temperature to 95 °C. Then the mixture was stirred for 12 hours (monitored by TLC). Once the reaction was complete, remove most of the solvent under vacuum. Next, dissolve the residue in 20 mL of ethyl acetate, then add 20 mL of saturated NaCl. Separate the organic phase and wash the aqueous phase with EA for three times. Remove the organic solvents under reduced pressure and the residue was purified by column chromatography. Finally,

crystallize the product from a hybrid solvent of ethyl acetate and petroleum ether to obtain the target compounds **IB-1(b – d)**.

S1.16.1 (*S*)-*N*-(1-(3-(4-chloro-3-(*N*-(ethylsulfonyl)ethylsulfonamido)-1-(2,2,2-trifluoroethyl)-1*H*-indazol-7-yl)-7-(4-cyano-3-fluorophenyl)-4-oxo-3,4-dihydroquinazolin-2-yl)-2-(3,5-difluorophenyl)ethyl)-2-(5-methyl-3-(trifluoromethyl)-1*H*-pyrazol-1-yl)acetamide (IB-1b)

White solid, yield 68%. m.p. 124 – 128°C. ^{1}H NMR (600 MHz, DMSO-d_6) δ 9.04 (d, J = 8.0 Hz, 1H, NH), 8.29 (d, J = 8.3 Hz, 1H, quinazolinone-H), 8.19 (d, J = 1.8 Hz, 1H, quinazolinone-H), 8.15 – 8.14 (m, 1H, Ph-H), 8.13 (d, J = 7.7 Hz, 1H, Ph-H), 8.08 (dd, J = 8.4, 1.9 Hz, 1H, quinazolinone-H), 7.96 (dd, J = 8.1, 1.7 Hz, 1H, Ph-H), 7.84 (d, J = 8.0 Hz, 1H, indazole-H), 7.71 (d, J = 8.2 Hz, 1H, indazole-H), 7.03 (tt, J = 9.4, 2.3 Hz, 1H, Ph-H), 6.66 – 6.61 (m, 2H, Ph-H), 6.44 (s, 1H, pyrazole-H), 4.93 (dq, J = 17.0, 8.5 Hz, 1H, CF_3CH_2), 4.65 (d, J = 16.7 Hz, 1H, $COCH_2$), 4.61 (ddd, J = 9.8, 8.1, 3.8 Hz, 1H, CH), 4.55 (d, J = 16.7 Hz, 1H, $COCH_2$), 4.42 (dq, J = 16.7, 8.4 Hz, 1H, CF_3CH_2), 3.90 – 3.68 (m, 4H, SO_2CH_2×2), 3.41 (dd, J = 14.2, 3.7 Hz, 1H, Ph-CH_2), 3.04 (dd, J = 14.2, 9.9 Hz, 1H, Ph-CH_2), 2.03 (s, 3H, pyrazole-CH_3), 1.39 (t, J = 7.4 Hz, 3H, CH_3), 1.31 (t, J = 7.4 Hz, 3H, CH_3). ^{13}C NMR (150 MHz, DMSO-d_6) δ 166.55 (C=O), 162.62 (dd, $^1J_{CF}$ = 246.1, $^3J_{CF}$ = 13.5 Hz), 161.42 (C=O), 156.68, 147.22, 146.32 (t, $^3J_{CF}$ = 8.7 Hz), 144.39, 142.66, 138.89, 136.11, 13S12, 132.28, 128.20, 127.47, 127.26, 126.20, 12S11, 124.90, 124.88, 120.98, 120.92, 115.87 (d, $^2J_{CF}$ = 20.8 Hz), 112.57 (dd, $^2J_{CF}$ = 19.9, $^4J_{CF}$ = 4.4 Hz), 102.82 (t, $^2J_{CF}$ = 25.5 Hz), 100.66 (d, $^3J_{CF}$ = 15.4 Hz), 52.90, 52.04, 51.02, 50.60, 40.62, 38.23, 10.74, 7.84, 7.66. HRMS found m/z 1042.1615 $[M+H]^+$, calculated for $C_{34}H_{26}BrClF_8N_8O_4S$, $[M+H]^+$: 1042.1613. HPLC purity: 98.28 %.

S1.16.2 (*S*)-*N*-(1-(3-(4-chloro-3-(*N*-(ethylsulfonyl)ethylsulfonamido)-1-(2,2,2-trifluoroethyl)-1*H*-indazol-7-yl)-7-(4-cyano-3,5-difluorophenyl)-4-oxo-3,4-dihydroquinazolin-2-yl)-2-(3,5-difluorophenyl)ethyl)-2-(5-methyl-3-(trifluoromethyl)-1*H*-pyrazol-1-yl)acetamide (IB-1c)

White solid, yield 53%. m.p. 132 – 136°C. ^{1}H NMR (600 MHz, DMSO-d_6) δ 9.02 (d, J = 8.0 Hz, 1H, NH), 8.29 (d, J = 8.3 Hz, 1H, quinazolinone-H), 8.24 (d, J = 1.9 Hz, 1H, quinazolinone-H), 8.11 (dd, J = 8.3, 1.9 Hz, 1H, quinazolinone-H), 8.08 (d, J = 9.3 Hz, 2H, Ph-H), 7.83 (d, J = 8.0 Hz, 1H, indazole-H), 7.71 (d, J = 8.0 Hz, 1H, indazole-H), 7.03 (tt, J = 9.4, 2.4 Hz, 1H, Ph-H), 6.66 – 6.61 (m, 2H, Ph-H), 6.44 (s, 1H, pyrazole-H), 4.94 (dq, J = 17.0, 8.6 Hz, 1H, CF_3CH_2), 4.66 – 4.50 (m, 3H, $COCH_2$, CH), 4.41 (dq, J = 16.7, 8.4 Hz, 1H, CF_3CH_2), 3.90 – 3.67 (m, 4H, SO_2CH_2×2), 3.42 (dd, J = 14.2, 3.8 Hz, 1H, Ph-CH_2), 3.04 (dd, J = 14.2, 9.9 Hz, 1H, Ph-CH_2), 2.03 (s, 3H, pyrazole-CH_3), 1.39 (t, J = 7.4 Hz, 3H, CH_3), 1.31 (t, J = 7.4 Hz, 3H, CH_3). ^{13}C NMR (150 MHz, DMSO-d_6) δ 166.51 (C=O), 163.22 (dd, $^1J_{CF}$ = 259.5, $^3J_{CF}$ = 6.6 Hz), 162.59 (dd, $^1J_{CF}$ = 248.2, $^3J_{CF}$ = 15.3 Hz), 161.37 (C=O), 156.70, 147.31 (t, $^3J_{CF}$ = 11.0 Hz), 147.15, 143.34, 142.66, 141.87 (t, $^3J_{CF}$ = 9.7 Hz), 140.32, 140.07, 138.84, 136.06, 132.23, 128.17, 127.44, 127.23, 126.46, 12S12, 123.72 (d, $^1J_{CF}$ = 269.1 Hz), 122.99 (d, $^1J_{CF}$ = 280.6 Hz), 121.30, 119.33, 112.54 (dd, $^2J_{CF}$ = 19.9, $^4J_{CF}$ = 4.9 Hz), 112.20 (dd, $^2J_{CF}$ = 21.2, $^4J_{CF}$ = 3.1 Hz), 110.06, 104.18, 102.81 (t, $^2J_{CF}$ = 25.3 Hz), 91.43 (t, $^2J_{CF}$ = 19.7 Hz), 52.89, 51.97, 50.99, 50.55, 40.54, 38.17, 10.71, 7.82, 7.64. HRMS found m/z 1060.1524 $[M+H]^+$, calculated for $C_{43}H_{32}ClF_{10}N_9O_6S_2$, $[M+H]^+$: 1060.1519. HPLC purity: 95.81 %.

S1.16.3 (*S*)-*N*-(1-(3-(4-chloro-3-(*N*-(ethylsulfonyl)ethylsulfonamido)-1-(2,2,2-trifluoroethyl)-1*H*-indazol-7-yl)-7-(4-nitrophenyl)-4-oxo-3,4-dihydroquinazolin-2-yl)-2-(3,5-difluorophenyl)ethyl)-2-(5-methyl-3-(trifluoromethyl)-1*H*-pyrazol-1-yl)acetamide (IB-1d)

White solid, yield 68%. m.p. 161 – 165°C. ^{1}H NMR (600 MHz, DMSO-d_6) δ 9.06 (d, J = 8.1 Hz, 1H, NH), 8.41 – 8.37 (m, 2H, Ph-H), 8.30 (d, J = 8.3 Hz, 1H, quinazolinone-H), 8.18 – 8.13 (m, 3H, Ph-H, quinazolinone-H), 8.07 (dd, J = 8.3, 1.8 Hz, 1H, quinazolinone-H), 7.83 (d, J = 7.9 Hz, 1H, indazole-H), 7.71 (d, J = 8.0 Hz, 1H, indazole-H), 7.02 (tt, J = 9.4, 2.4 Hz, 1H, Ph-H), 6.61 (dd, J = 8.2, 2.2 Hz, 2H, Ph-H), 6.42 (s, 1H, pyrazole-H), 4.91 (dq, J = 17.0, 8.6 Hz, 1H, CF_3CH_2), 4.66 (d, J = 16.7 Hz,

1H, $COCH_2$), 4.61 – 4.57 (m, 1H, CH), 4.55 (d, *J* = 16.5 Hz, 1H, $COCH_2$), 4.41 (dq, *J* = 16.7, 8.4 Hz, 1H, CF_3CH_2), 3.88 – 3.66 (m, 4H, SO_2CH_2×2), 3.39 (dd, *J* = 14.2, 3.7 Hz, 1H, Ph-CH_2), 3.03 (dd, *J* = 14.2, 10.0 Hz, 1H, Ph-CH_2), 2.02 (s, 3H, pyrazole-CH_3), 1.38 (t, *J* = 7.4 Hz, 3H, CH_3), 1.30 (t, *J* = 7.4 Hz, 3H, CH_3). ^{13}C NMR (150 MHz, DMSO-d_6) δ 166.56 (C=O), 162.61 (dd, $^1J_{CF}$ = 246.4, $^3J_{CF}$ = 13.6 Hz), 161.46 (C=O), 156.74, 148.12, 147.27, 14S19, 14S13, 142.64, 141.89 (t, $^3J_{CF}$ = 9.7 Hz), 140.35, 138.88, 136.08, 132.29, 129.20, 128.29, 127.48, 127.26, 126.06, 12S11, 124.79, 123.02 (d, $^1J_{CF}$ = 280.5 Hz), 121.96 (d, $^1J_{CF}$ = 268.1 Hz), 120.98, 120.68, 119.40, 112.57 (dd, $^2J_{CF}$ = 19.9, $^4J_{CF}$ = 4.8 Hz), 104.17, 102.82 (t, $^2J_{CF}$ = 26.1 Hz), 52.87, 52.05, 51.58, 51.01, 50.59, 38.27, 10.74, 7.84, 7.66. HRMS found m/z 1044.1604 $[M+H]^+$, calculated for $C_{42}H_{34}ClF_8N_9O_8S_2$, $[M+H]^+$: 1044.1605. HPLC purity: 98.84 %.

S1.17 General procedure for the synthesis of IB-2(b – d)

Dissolve **IB-1(b – d)** (1.0 eq.) and K_2CO_3 (3.0 eq.) in 10 mL DMF and 1 mL water. Then raise the temperature to 75 °C and stir for 6 h (monitored by TLC). Once the reaction was complete, add 20 mL of ethyl acetate and 30 mL of saturated NaCl to the mixture. Next, separate the organic phase and wash the aqueous phase with ethyl acetate. Combine the organic phases, dry them with sodium sulfate, and filter. Remove the organic solvents under reduced pressure and the residue was purified by column chromatography. Finally, crystallize the product from a hybrid solvent of ethyl acetate and petroleum ether to obtain the target compound **IB-2(b – d)**.

S1.17.1 (*S*)-*N*-(1-(3-(4-chloro-3-(ethylsulfonamido)-1-(2,2,2-trifluoroethyl)-1*H*-indazol-7-yl)-7-(4-cyano-3-fluorophenyl)-4-oxo-3,4-dihydroquinazolin-2-yl)-2-(3,5-difluorophenyl)ethyl)-2-(5-methyl-3-(trifluoromethyl)-1*H*-pyrazol-1-yl)acetamide (IB-2b)

White solid, yield 62%. m.p. 93 – 96°C. ^1H NMR (600 MHz, DMSO-d_6) δ 10.05 (s, 1H, SO_2NH), 9.13 (d, *J* = 8.4 Hz, 1H, NH), 8.31 (d, *J* = 8.2 Hz, 1H, quinazolinone-H), 8.18 (d, *J* = 1.8 Hz, 1H, quinazolinone-H), 8.15 – 8.13 (m, 1H, Ph-H), 8.12 (d, *J* = 7.3 Hz, 1H, Ph-H), 8.08 (dd, *J* = 8.3, 1.8 Hz, 1H, quinazolinone-H), 7.95 (dd, *J* = 8.1, 1.7 Hz,

1H, Ph-H), 7.77 (d, J = 7.9 Hz, 1H, indazole-H), 7.57 (d, J = 7.9 Hz, 1H, indazole-H), 7.00 (tt, J = 9.4, 2.4 Hz, 1H, Ph-H), 6.67 – 6.61 (m, 2H, Ph-H), 6.40 (s, 1H, pyrazole-H), 4.77 (dq, J = 17.0, 8.6 Hz, 1H, CF_3CH_2), 4.66 (s, 2H, $COCH_2$), 4.60 (ddd, J = 10.2, 8.4, 3.6 Hz, 1H, CH), 4.28 (dq, J = 16.9, 8.9 Hz, 1H, CF_3CH_2), 3.40 (dd, J = 14.2, 3.7 Hz, 1H, Ph-CH_2), 3.36 – 3.31 (m, 2H, SO_2CH_2), 3.03 (dd, J = 14.2, 10.1 Hz, 1H, Ph-CH_2), 2.01 (s, 3H, pyrazole-CH_3), 1.32 (t, J = 7.3 Hz, 3H, CH_3). ^{13}C NMR (150 MHz, DMSO-d_6) δ 166.40 (C=O), 163.42 (d, $^1J_{CF}$ = 255.5 Hz), 162.60 (dd, $^1J_{CF}$ = 246.1, $^3J_{CF}$ = 13.4 Hz), 161.33 (C=O), 157.07, 147.32, 146.33 (d, $^3J_{CF}$ = 8.4 Hz), 144.35, 142.57, 141.85 (t, $^3J_{CF}$ = 9.6 Hz), 140.25, 138.46, 13S12, 131.84, 128.41, 128.22, 127.43, 126.13, 124.89 (d, $^4J_{CF}$ = 2.9 Hz), 123.57, 123.20 (d, $^1J_{CF}$ = 280.9 Hz), 121.96 (d, $^1J_{CF}$ = 268.0 Hz), 121.03, 119.53, 118.74, 115.87 (d, $^2J_{CF}$ = 20.9 Hz), 114.35, 112.52 (dd, $^2J_{CF}$ = 19.7, $^4J_{CF}$ = 4.7 Hz), 104.01, 102.78, 100.66 (d, $^2J_{CF}$ = 15.4 Hz), 52.66, 51.98, 48.10, 40.63, 38.52, 10.78, 8.35. HRMS found m/z 950.1682 $[M+H]^+$, calculated for $C_{41}H_{29}ClF_9N_9O_4S$, $[M+H]^+$: 950.1681. HPLC purity: 98.83 %.

S1.17.2 (*S*)-*N*-(1-(3-(4-chloro-3-(ethylsulfonamido)-1-(2,2,2-trifluoroethyl)-1*H*-indazol-7-yl)-7-(4-cyano-3,5-difluorophenyl)-4-oxo-3,4-dihydroquinazolin-2-yl)-2-(3,5-difluorophenyl)ethyl)-2-(5-methyl-3-(trifluoromethyl)-1*H*-pyrazol-1-yl)acetamide (IB-2c)

White solid, yield 76%. m.p.100 – 103°C. ^{1}H NMR (600 MHz, DMSO-d_6) δ 10.05 (s, 1H, SO_2NH), 9.11 (d, J = 8.4 Hz, 1H, NH), 8.31 (d, J = 8.3 Hz, 1H, quinazolinone-H), 8.22 (d, J = 1.9 Hz, 1H, quinazolinone-H), 8.10 (dd, J = 8.3, 1.9 Hz, 1H, quinazolinone-H), 8.07 (d, J = 9.3 Hz, 2H, Ph-H), 7.76 (d, J = 7.9 Hz, 1H, indazole-H), 7.57 (d, J = 8.0 Hz, 1H, indazole-H), 7.00 (tt, J = 9.4, 2.4 Hz, 1H, Ph-H), 6.66 – 6.62 (m, 2H, Ph-H), 6.40 (s, 1H, pyrazole-H), 4.78 (dq, J = 17.0, 8.6 Hz, 1H, CF_3CH_2), 4.65 (s, 2H, $COCH_2$), 4.61 (ddd, J = 10.1, 8.3, 3.7 Hz, 1H, CH), 4.27 (dq, J = 16.9, 8.6 Hz, 1H, CF_3CH_2), 3.41 (dd, J = 14.2, 3.7 Hz, 1H, Ph-CH_2), 3.34 (td, J = 7.2, 1.9 Hz, 2H, SO_2CH_2), 3.03 (dd, J = 14.2, 10.1 Hz, 1H, Ph-CH_2), 2.01 (s, 3H, pyrazole-CH_3), 1.32 (t, J = 7.3 Hz, 3H, CH_3). ^{13}C NMR (150 MHz, DMSO-d_6) δ 166.38 (C=O), 163.25 (dd,

$^{1}J_{CF}$ = 257.3, $^{3}J_{CF}$ = 5.2 Hz), 162.61 (dd, $^{1}J_{CF}$ = 246.5, $^{3}J_{CF}$ = 13.3 Hz),161.29 (C=O), 157.13, 147.28, 143.33, 142.59, 141.84, 140.26, 138.46, 131.83, 130.12, 128.42, 128.22, 127.43, 126.43, 123.59, 123.20 (d, $^{1}J_{CF}$ = 280.3 Hz), 121.96 (d, $^{1}J_{CF}$ = 268.2 Hz), 121.44, 119.53, 118.71, 112.52 (dd, $^{2}J_{CF}$ = 20.3, $^{4}J_{CF}$ = 4.6 Hz), 112.23 (dd, $^{2}J_{CF}$ = 21.2, $^{4}J_{CF}$ = 3.0 Hz), 110.04, 104.02, 102.79 (t, $^{2}J_{CF}$ = 25.8 Hz), 91.44, 52.67, 51.95, 48.10, 38.50, 22.49, 10.78, 8.35. HRMS found m/z 968.1585 $[M+H]^+$, calculated for $C_{41}H_{28}ClF_{10}N_9O_4S$, $[M+H]^+$: 968.1587. HPLC purity: 99.54 %.

S1.17.3 (*S*)-*N*-(1-(3-(4-chloro-3-(ethylsulfonamido)-1-(2,2,2-trifluoroethyl)-1*H*-indazol-7-yl)-7-(4-nitrophenyl)-4-oxo-3,4-dihydroquinazolin-2-yl)-2-(3,5-difluorophenyl)ethyl)-2-(5-methyl-3-(trifluoromethyl)-1*H*-pyrazol-1-yl)acetamide (IB-2d)

White solid, yield 84%. m.p. 168 – 172°C. ^{1}H NMR (600 MHz, DMSO-d_6) δ 10.06 (s, 1H, SO_2NH), 9.16 (d, J = 8.4 Hz, 1H, NH), 8.41 (d, J = 8.7 Hz, 2H, Ph-H), 8.33 (d, J = 8.2 Hz, 1H, quinazolinone-H), 8.18 (d, J = 8.7 Hz, 3H, Ph-H, quinazolinone-H), 8.08 (dd, J = 8.2, 1.8 Hz, 1H, quinazolinone-H), 7.78 (d, J = 7.9 Hz, 1H, indazole-H), 7.59 (d, J = 7.9 Hz, 1H, indazole-H), 7.01 (tt, J = 9.4, 2.4 Hz, 1H, Ph-H), 6.66 – 6.61 (m, 2H, Ph-H), 6.40 (s, 1H, pyrazole-H), 4.77 (dq, J = 17.0, 8.6 Hz, 1H, CF_3CH_2), 4.73 – 4.64 (m, 2H, $COCH_2$), 4.60 (ddd, J = 10.1, 8.4, 3.6 Hz, 1H, CH), 4.29 (dq, J = 16.9, 8.6 Hz, 1H, CF_3CH_2), 3.40 (dd, J = 14.2, 3.6 Hz, 1H, Ph-CH_2), 3.33 (q, J = 7.4 Hz, 2H, SO_2CH_2), 3.04 (dd, J = 14.2, 10.2 Hz, 1H, Ph-CH_2), 2.02 (s, 3H, pyrazole-CH_3), 1.33 (t, J = 7.3 Hz, 3H, CH_3). ^{13}C NMR (150 MHz, DMSO-d_6) δ 166.41 (C=O), 162.61 (dd, $^{1}J_{CF}$ = 246.5, $^{3}J_{CF}$ = 13.3 Hz), 161.37 (C=O), 157.13, 148.14, 147.38, 14S16, 14S14, 142.55, 141.86 (t, $^{3}J_{CF}$ = 9.4 Hz), 140.25, 140.01, 138.47, 131.85, 129.19, 128.42, 128.31, 127.43, 126.00, 124.79, 123.58, 123.21 (d, $^{1}J_{CF}$ = 280.6 Hz), 121.96 (d, $^{1}J_{CF}$ = 268.1 Hz), 120.79, 119.55, 118.77, 112.53 (dd, $^{2}J_{CF}$ = 19.7, $^{4}J_{CF}$ = 4.9 Hz), 104.01, 102.78 (t, $^{2}J_{CF}$ = 25.8 Hz), 52.64, 52.00, 51.06, 48.10, 38.58, 10.78, 8.35. ^{19}F NMR (376 MHz, DMSO-d_6) δ -60.79 (pyrazole-CF_3), -68.66 (t, J = 8.6 Hz, indazole-CH_2CF_3),

-109.96 (t, J = 8.5 Hz, Ph-F × 2). HRMS found m/z 952.1677 $[M+H]^+$, calculated for $C_{40}H_{30}ClF_8N_9O_6S$, $[M+H]^+$: 952.1673. HPLC purity: 99.57 %.

S1.18 (*S*)-*N*-(1-(7-bromo-3-(4-chloro-3-(*N*-(ethylsulfonyl)ethylsulfonamido)-1-(2,2,2-trifluoroethyl)-1*H*-indazol-7-yl)-4-oxo-3,4-dihydroquinazolin-2-yl)-2-(3,5-difluorophenyl)ethyl)-2-(3-(trifluoromethyl)-5,6-dihydrocyclopenta[*c*]pyrazol-1(4*H*)-yl)acetamide (IC-1a)

Under ice bath, intermediate (3-trifluoromethyl-5,6-dihydro-4h-cyclopenta-pyrazol-1-yl)-acetic acid (0.97 g, 4.14 mmol) and HATU (1.97 g, S17 mmol) were mixed in 30 mL DCM and stirred for 0.5 h. Then, the intermediate **a-7** (2.80 g, 3.45 mmol) and DIEA (0.89 g, 6.90 mmol) were slowly added to the mixture at 0 ºC. The reaction system was then stirred at room temperature for an 2 h (monitored by TLC). Once the reaction was complete, the mixture was initially washed with saturated NaCl and extracted with DCM (3 × 20 mL), and dried over anhydrous Na_2SO_4, filtered, and concentrated under reduced pressure to afford a corresponding crude product, purified by flash column chromatography to afford the crude. Finally, the crude was crystallized using a mixed solvent of ethyl acetate and petroleum ether to obtain the target compound **IC-1a**. White solid, yield 76%. m.p. 121 – 123°C. ^{1}H NMR (600 MHz, DMSO-d_6) δ 9.05 (d, J = 8.0 Hz, 1H, NH), 8.10 (d, J = 8.5 Hz, 1H, quinazolinone-H), 8.01 (d, J = 1.9 Hz, 1H, quinazolinone-H), 7.87 (d, J = 8.0 Hz, 1H, indazole-H), 7.85 (dd, J = 8.5, 1.9 Hz, 1H, quinazolinone-H), 7.72 (d, J = 8.0 Hz, 1H, indazole-H), 7.02 (tt, J = 9.4, 2.4 Hz, 1H, Ph-H), 6.63 – 6.57 (m, 2H, Ph-H), 4.98 (dq, J = 17.0, 8.6 Hz, 1H, CF_3CH_2), 4.58 – 4.44 (m, 3H, $COCH_2$, CH), 4.40 (dq, J = 16.8, 8.5 Hz, 1H, CF_3CH_2), 3.82 (dddt, J = 63.4, 28.8, 14.3, 7.3 Hz, 4H, SO_2CH_2×2), 3.36 (dd, J = 14.3, 3.6 Hz, 1H, Ph-CH_2), 3.00 (dd, J = 14.3, 10.1 Hz, 1H, Ph-CH_2), 2.57 (t, J = 6.7 Hz, 2H, pyrazole-CH_2), 2.48 – 2.37 (m, 4H, pyrazole-CH_2, CH_2), 1.39 (t, J = 7.4 Hz, 3H, CH_3), 1.33 (t, J = 7.4 Hz, 3H, CH_3). ^{13}C NMR (150 MHz, DMSO-d_6) δ 166.47 (C=O), 162.58 (dd, $^1J_{CF}$ = 246.5, $^3J_{CF}$ = 13.5 Hz), 161.33 (C=O), 157.46, 153.81, 147.84, 141.86 (t, $^3J_{CF}$ = 9.3 Hz), 138.80, 136.10, 134.24, 132.32, 131.67, 129.99, 129.68, 129.21, 127.30,

125.27, 12S13, 123.02 (d, $^{1}J_{CF}$ = 280.9 Hz), 122.13 (d, $^{1}J_{CF}$ = 268.2 Hz), 121.00, 119.95, 119.22, 112.55 (dd, $^{2}J_{CF}$ = 20.1, $^{4}J_{CF}$ = 5.0 Hz), 102.78 (t, $^{2}J_{CF}$ = 25.4 Hz), 53.15, 52.94, 50.93, 50.80, 38.71, 37.97, 30.74, 23.70, 23.02, 7.82, 7.67. HRMS found m/z 1027.0706 $[M+H]^+$, calculated for $C_{38}H_{32}BrClF_8N_8O_6S_2$, $[M+H]^+$: 1027.0703. HPLC purity: 100 %.

S1.19 (*S*)-*N*-(1-(7-bromo-3-(4-chloro-3-(ethylsulfonamido)-1-(2,2,2-trifluoroethyl)-1*H*-indazol-7-yl)-4-oxo-3,4-dihydroquinazolin-2-yl)-2-(3,5-difluorophenyl)ethyl)-2-(3-(trifluoromethyl)-5,6-dihydrocyclopenta[*c*]pyrazol-1(4*H*)-yl)acetamide (IC-2a)

IC-1a (100.0 mg, 97.26 μmol) and K_2CO_3 (40.3 mg, 291.78 μmol) were dissolved in 10 mL DMF and 1 mL water. Then the temperature rose to 75 °C and stir for 6 h (monitored by TLC). Once the reaction was complete, add 20 mL of ethyl acetate and 30 mL of saturated NaCl to the mixture. Next, separate the organic phase and wash the aqueous phase with ethyl acetate. Combine the organic phases, dry them with sodium sulfate, and filter. Remove the organic solvents under reduced pressure and the residue was purified by column chromatography. Finally, crystallize the product from a hybrid solvent of ethyl acetate and petroleum ether to obtain the target compound **IC-2a**. White solid, yield 73%. m.p. 121 – 125°C. ^{1}H NMR (600 MHz, Chloroform-*d*) δ 8.13 (d, *J* = 8.5 Hz, 1H, quinazolinone-H), 7.95 (d, *J* = 1.8 Hz, 1H, quinazolinone-H), 7.70 (dd, *J* = 8.4, 1.9 Hz, 1H, quinazolinone-H), 7.51 (s, 1H, SO_2NH), 7.40 (d, *J* = 8.2 Hz, 1H, indazole-H), 7.25 (d, *J* = 8.0 Hz, 1H, indazole-H), 6.85 (dd, *J* = 7.9, 1.9 Hz, 1H, NH), 6.67 (tt, *J* = 8.8, 2.3 Hz, 1H, Ph-H), 6.31 – 6.26 (m, 2H, Ph-H), 4.59 (td, *J* = 8.0, 5.8 Hz, 1H, CH), 4.56 – 4.41 (m, 3H, $COCH_2$, CF_3CH_2), 4.20 (dq, *J* = 16.4, 8.2 Hz, 1H, CF_3CH_2), 3.57 (dp, *J* = 24.6, 7.2 Hz, 2H, SO_2CH_2), 3.16 (dd, *J* = 13.8, 5.9 Hz, 1H, Ph-CH_2), 2.88 – 2.80 (m, 1H, Ph-CH_2), 2.74 (hept, *J* = 8.4, 7.8 Hz, 2H, pyrazole-CH_2), 2.67 – 2.54 (m, 4H, pyrazole-CH_2-CH_2), 1.47 (t, *J* = 7.4 Hz, 3H, CH_3). ^{13}C NMR (150 MHz, Chloroform-*d*) δ 165.26 (C=O), 162.93 (dd, $^{1}J_{CF}$ = 250.4, $^{3}J_{CF}$ = 12.8 Hz), 161.07 (C=O), 155.98, 153.58, 147.12, 140.83, 138.89 (t, $^{3}J_{CF}$ = 9.1 Hz), 137.93, 137.30,

137.56, 131.95, 130.97, 130.78, 130.57, 128.89, 128.74, 122.43 (q, J = 280.9 Hz), 122.03, 121.20 (d, J = 268.8 Hz), 118.94, 117.33, 116.53, 112.09 (dd, $^2J_{CF}$ = 19.8, $^4J_{CF}$ =5.0 Hz), 103.02 (t, $^2J_{CF}$ = 25.0 Hz), 53.22, 51.76, 51.20 (q, $^2J_{CF}$ = 35.9 Hz), 47.93, 40.28, 30.75, 23.51, 23.02, 8.08. ^{19}F NMR (376 MHz, DMSO-d_6) δ -60.28 (pyrazole-CF_3), -68.71 (t, J = 8.5 Hz, indazole-CH_2CF_3), -109.96 (t, J = 8.5 Hz, Ph-F × 2). HRMS found m/z 935.0774 $[M+H]^+$, calculated for $C_{36}H_{28}BrClF_8N_8O_4S$, $[M+H]^+$: 935.0771. HPLC purity: 99.13 %. Elemental analyses found: C, 45.432; H, 2.974; N, 11.689; S, 3.345 %. Calc. for $C_{36}H_{28}BrClF_8N_8O_4S$: C, 46.19; H, 3.02; N, 11.97; S, 3.42 %.

S1.20 General procedure for the synthesis of IC-1(b – k)

Dissolve **IC-1a** (1.0 eq.), different substituted phenylboric acid or pinacol ester of phenylboric acid (1.2 equiv), K_3PO_4 (3.0 eq.) and $Pd(PPh_3)_4$ (0.2 eq.) in 10 mL toluene and 2 mL water. Replace the air with nitrogen and increase the temperature to 95 °C. Then the mixture was stirred for 12 hours (monitored by TLC). Once the reaction was complete, remove most of the solvent under vacuum. Next, dissolve the residue in 20 mL of ethyl acetate, then add 20 mL of saturated NaCl. Separate the organic phase and wash the aqueous phase with EA for three times. Remove the organic solvents under reduced pressure and the residue was purified by column chromatography. Finally, crystallize the product from a hybrid solvent of ethyl acetate and petroleum ether to obtain the target compound **IC-1(b – k)**.

S1.20.1 (*S*)-*N*-(1-(3-(4-chloro-3-(*N*-(ethylsulfonyl)ethylsulfonamido)-1-(2,2,2-trifluoroethyl)-1*H*-indazol-7-yl)-4-oxo-7-phenyl-3,4-dihydroquinazolin-2-yl)-2-(3,5-difluorophenyl)ethyl)-2-(3-(trifluoromethyl)-5,6-dihydrocyclopenta[*c*]pyrazol-1(4*H*)-yl)acetamide (IC-1b)

White solid, yield 60%. m.p. 131 – 134°C. ^{1}H NMR (600 MHz, DMSO-d_6) δ 9.07 (d, J = 8.1 Hz, 1H, NH), 8.26 (d, J = 8.2 Hz, 1H, quinazolinone-H), 8.06 (d, J = 1.8 Hz, 1H, quinazolinone-H), 8.00 (dd, J = 8.2, 1.8 Hz, 1H, quinazolinone-H), 7.91 – 7.87 (m, 3H, Ph-H, indazole-H), 7.73 (d, J = 7.9 Hz, 1H, indazole-H), 7.59 (t, J = 7.6 Hz, 2H, Ph-H), 7.52 (t, J = 7.3 Hz, 1H, Ph-H), 7.04 (tt, J = 9.4, 2.4 Hz, 1H, Ph-H), 6.66 – 6.60

(m, 2H, Ph-H), 5.01 (dq, *J* = 17.0, 8.6 Hz, 1H, CF_3CH_2), 4.61 (ddd, *J* = 10.1, 8.1, 3.7 Hz, 1H, CH), 4.57 – 4.40 (m, 3H, CF_3CH_2, $COCH_2$), 3.88 (ddt, *J* = 27.1, 14.8, 7.4 Hz, 2H, SO_2CH_2), 3.78 (ddt, *J* = 30.8, 14.2, 7.3 Hz, 2H, SO_2CH_2), 3.41 (dd, *J* = 14.2, 3.6 Hz, 1H, Ph-CH_2), 3.05 (dd, *J* = 14.2, 10.1 Hz, 1H, Ph-CH_2), 2.56 (t, *J* = 6.8 Hz, 2H, pyrazole-CH_2), 2.49 – 2.41 (m, 2H, CH_2), 2.40 (t, *J* = 4.8 Hz, 2H, pyrazole-CH_2), 1.40 (t, *J* = 7.4 Hz, 3H, CH_3), 1.33 (t, *J* = 7.4 Hz, 3H, CH_3). ^{13}C NMR (150 MHz, DMSO-d_6) δ 166.37 (C=O), 162.57 (dd, $^{1}J_{CF}$ = 246.2, $^{3}J_{CF}$ = 13.2 Hz), 161.56 (C=O), 156.32, 153.82, 147.62, 147.27, 142.00 (t, $^{3}J_{CF}$ = 9.7 Hz), 138.94, 138.81, 136.05, 134.45, 132.34, 129.81, 129.47, 127.99, 127.73, 127.23, 127.13, 125.28, 12S13, 125.04, 123.05 (d, $^{1}J_{CF}$ = 280.6 Hz), 122.12 (d, $^{1}J_{CF}$ = 268.3 Hz), 120.94, 119.58, 119.55, 112.58 (dd, $^{2}J_{CF}$ = 19.6, $^{4}J_{CF}$ = 4.9 Hz), 102.75 (t, $^{2}J_{CF}$ = 25.7 Hz), 53.13, 52.83, 50.92, 50.78, 38.18, 30.73, 23.68, 23.01, 7.84, 7.68. HRMS found m/z 102S1909 $[M+H]^+$, calculated for $C_{44}H_{37}ClF_8N_8O_6S_2$, $[M+H]^+$: 102S1911. HPLC purity: 95.61 %.

S1.20.2 (*S*)-*N*-(1-(3-(4-chloro-3-(*N*-(ethylsulfonyl)ethylsulfonamido)-1-(2,2,2-trifluoroethyl)-1*H*-indazol-7-yl)-7-(4-cyanophenyl)-4-oxo-3,4-dihydroquinazolin-2-yl)-2-(3,5-difluorophenyl)ethyl)-2-(3-(trifluoromethyl)-5,6-dihydrocyclopenta [*c*]pyrazol-1(4*H*)-yl)acetamide (IC-1c)

White solid, yield 64%. m.p. 191 – 195°C. ^{1}H NMR (400 MHz, DMSO-d_6) δ 9.02 (d, *J* = 8.1 Hz, 1H, NH), 8.29 (d, *J* = 8.3 Hz, 1H, quinazolinone-H), 8.12 (d, *J* = 10.7 Hz, 2H, Ph-H), 8.07 (d, *J* = 11.7 Hz, 3H, Ph-H, quinazolinone-H), 8.04 (d, *J* = 2.3 Hz, 1H, quinazolinone-H), 7.86 (d, *J* = 7.9 Hz, 1H, indazole-H), 7.72 (d, *J* = 7.4 Hz, 1H, indazole-H), 7.02 (t, *J* = 9.6 Hz, 1H, Ph-H), 6.63 (d, *J* = 7.5 Hz, 2H, Ph-H), 4.99 (dq, *J* = 17.0, 8.5 Hz, 1H, CF_3CH_2), 4.66 – 4.58 (m, 1H, CH), 4.55 – 4.36 (m, 3H, CF_3CH_2, $COCH_2$), 3.88 (ddd, *J* = 19.5, 13.4, 7.4 Hz, 2H, SO_2CH_2), 3.75 (ddt, *J* = 21.4, 14.5, 7.5 Hz, 2H, SO_2CH_2), 3.43 – 3.38 (m, 1H, Ph-CH_2), 3.04 (dd, *J* = 14.2, 10.1 Hz, 1H, Ph-CH_2), 2.54 (d, *J* = 6.0 Hz, 2H, pyrazole-CH_2), 2.45 (d, *J* = 17.9 Hz, 2H, CH_2), 2.40 (d, *J* = 7.5 Hz, 2H, pyrazole-CH_2), 1.40 (t, *J* = 7.4 Hz, 3H, CH_3), 1.32 (t, *J* = 7.4 Hz, 3H, CH_3). ^{13}C NMR (150 MHz, DMSO-d_6) δ 166.37 (C=O), 162.59 (dd, $^{1}J_{CF}$ = 246.4, $^{3}J_{CF}$

= 12.7 Hz), 161.49 (C=O), 156.57, 153.84, 147.24, 145.66, 143.27, 138.91, 136.08, 133.62, 132.31, 128.78, 128.20, 127.41, 127.18, 125.86, 12S15, 123.01, 121.30 (d, $^{1}J_{CF}$ = 247.3 Hz), 120.22 (d, $^{1}J_{CF}$ = 224.3 Hz), 112.58 (dd, $^{2}J_{CF}$ = 20.2, $^{4}J_{CF}$ = 4.5 Hz), 112.01, 53.10, 52.83, 50.93, 50.78, 30.73, 23.68, 23.00, 7.84, 7.68. HRMS found m/z 1050.1861 $[M+H]^+$, calculated for $C_{45}H_{36}ClF_8N_9O_6S_2$, $[M+H]^+$: 1050.1864. HPLC purity: 99.19 %. Elemental analyses found: C, 51.712; H, 3.669; N, 11.518; S, 5.694 %. Calc. for $C_{45}H_{36}ClF_8N_9O_6S_2$: C, 51.46; H, 3.45; N, 12.00; S, 6.10 %.

S1.20.3 (*S*)-*N*-(1-(3-(4-chloro-3-(*N*-(ethylsulfonyl)ethylsulfonamido)-1-(2,2,2-trifluoroethyl)-1*H*-indazol-7-yl)-7-(4-cyano-3-fluorophenyl)-4-oxo-3,4-dihydroquinazolin-2-yl)-2-(3,5-difluorophenyl)ethyl)-2-(3-(trifluoromethyl)-5,6-dihydrocyclopenta[*c*]pyrazol-1(4*H*)-yl)acetamide (IC-1d)

White solid, yield 54%. m.p. 130 – 134°C. ^{1}H NMR (600 MHz, DMSO-d_6) δ 9.03 (d, J = 8.1 Hz, 1H, NH), 8.30 (d, J = 8.3 Hz, 1H, quinazolinone-H), 8.20 (d, J = 1.8 Hz, 1H, quinazolinone-H), 8.14 (d, J = 8.1 Hz, 1H, Ph-H), 8.13 – 8.12 (m, 1H, Ph-H), 8.09 (dd, J = 8.3, 1.8 Hz, 1H, quinazolinone-H), 7.97 (dd, J = 8.1, 1.7 Hz, 1H, Ph-H), 7.87 (d, J = 8.0 Hz, 1H, indazole-H), 7.73 (d, J = 8.0 Hz, 1H, indazole-H), 7.03 (tt, J = 9.3, 2.4 Hz, 1H, Ph-H), 6.67 – 6.62 (m, 2H, Ph-H), 5.02 (dq, J = 17.0, 8.6 Hz, 1H, CF_3CH_2), 4.65 (ddd, J = 10.0, 8.1, 3.8 Hz, 1H, CH), 4.52 – 4.39 (m, 3H, $COCH_2$, CF_3CH_2), 3.88 (ddd, J = 28.5, 14.5, 7.4 Hz, 2H, SO_2CH_2), 3.77 (ddt, J = 37.9, 14.7, 7.4 Hz, 2H, SO_2CH_2), 3.42 (dd, J = 14.2, 3.7 Hz, 1H, Ph-CH_2), 3.05 (dd, J = 14.2, 10.0 Hz, 1H, Ph-CH_2), 2.56 (d, J = 6.0 Hz, 2H, pyrazole-CH_2), 2.49 – 2.41 (m, 2H, CH_2), 2.41 – 2.37 (m, 2H, pyrazole-CH_2), 1.40 (t, J = 7.4 Hz, 3H, CH_3), 1.33 (t, J = 7.4 Hz, 3H, CH_3). ^{13}C NMR (150 MHz, DMSO-d_6) δ 166.36 (C=O), 163.42 (d, $^{1}J_{CF}$ = 255.4 Hz), 162.59 (dd, $^{1}J_{CF}$ = 246.2, $^{3}J_{CF}$ = 13.4 Hz), 161.46 (C=O), 156.63, 153.85, 147.20, 146.27 (d, $^{3}J_{CF}$ = 8.7 Hz), 144.37, 141.95 (t, $^{3}J_{CF}$ = 8.7 Hz), 138.91, 136.09, 13S10, 134.47, 132.28, 128.19, 127.46, 127.20, 126.22, 125.29, 12S16, 124.88 (d, $^{4}J_{CF}$ = 3.3 Hz), 123.05 (d, $^{1}J_{CF}$ = 280.4 Hz), 122.11 (d, $^{1}J_{CF}$ = 268.1 Hz), 120.94 (d, $^{3}J_{CF}$ = 8.3 Hz), 119.44, 115.93, 115.79, 114.36, 112.58 (dd, $^{2}J_{CF}$ = 20.1, $^{4}J_{CF}$ = 4.8 Hz), 102.76 (t, $^{2}J_{CF}$ = 25.2 Hz),

100.65 (d, $^{2}J_{CF}$ = 15.3 Hz), 53.09, 52.86, 51.35, 50.94, 50.77, 38.14, 30.74, 23.68, 23.01, 7.83, 7.67. HRMS found m/z 1068.1769 $[M+H]^+$, calculated for $C_{45}H_{35}ClF_9N_9O_6S_2$, $[M+H]^+$: 1068.1769. HPLC purity: 98.74 %.

S1.20.4 (*S*)-*N*-(1-(3-(4-chloro-3-(*N*-(ethylsulfonyl)ethylsulfonamido)-1-(2,2,2-trifluoroethyl)-1*H*-indazol-7-yl)-7-(4-cyano-3,5-difluorophenyl)-4-oxo-3,4-dihydroquinazolin-2-yl)-2-(3,5-difluorophenyl)ethyl)-2-(3-(trifluoromethyl)-5,6-dihydrocyclopenta[*c*]pyrazol-1(4*H*)-yl)acetamide (IC-1e)

White solid, yield 77%. m.p. 140 – 144°C. ^{1}H NMR (600 MHz, DMSO-d_6) δ 9.01 (d, J = 8.1 Hz, 1H, NH), 8.30 (d, J = 8.3 Hz, 1H, quinazolinone-H), 8.25 (d, J = 1.6 Hz, 1H, quinazolinone-H), 8.12 (dd, J = 8.3, 1.9 Hz, 1H, quinazolinone-H), 8.10 – 8.07 (m, 2H, Ph-H), 7.87 (d, J = 8.0 Hz, 1H, indazole-H), 7.72 (d, J = 8.0 Hz, 1H, indazole-H), 7.03 (tt, J = 9.4, 2.3 Hz, 1H, Ph-H), 6.67 – 6.62 (m, 2H, Ph-H), 5.03 (dq, J = 17.0, 8.6 Hz, 1H, CF_3CH_2), 4.66 (ddd, J = 9.9, 8.1, 3.8 Hz, 1H, CH), 4.50 – 4.39 (m, 3H, $COCH_2$, CF_3CH_2), 3.89 (ddq, J = 21.9, 14.8, 7.4 Hz, 2H, SO_2CH_2), 3.78 (ddt, J = 30.7, 14.2, 7.3 Hz, 2H, SO_2CH_2), 3.43 (dd, J = 14.2, 3.8 Hz, 1H, Ph-CH_2), 3.05 (dd, J = 14.2, 10.0 Hz, 1H, Ph-CH_2), 2.56 (d, J = 6.7 Hz, 2H, pyrazole-CH_2), 2.49 – 2.41 (m, 2H, CH_2), 2.40 (t, J = 4.3 Hz, 2H, pyrazole-CH_2), 1.40 (t, J = 7.4 Hz, 3H, CH_3), 1.33 (t, J = 7.4 Hz, 3H, CH_3). ^{13}C NMR (150 MHz, DMSO-d_6) δ 166.34 (C=O), 163.24 (dd, $^{1}J_{CF}$ = 257.2, $^{3}J_{CF}$ = 5.2 Hz), 162.60 (dd, $^{1}J_{CF}$ = 246.5, $^{3}J_{CF}$ = 13.4 Hz), 161.42 (C=O), 156.69, 153.87, 147.32 (t, $^{3}J_{CF}$ = 10.0 Hz), 147.17, 143.35, 141.94 (t, $^{3}J_{CF}$ = 9.5 Hz), 138.90, 136.10, 134.48, 134.23, 132.26, 128.17, 127.45, 127.21, 126.50, 125.30, 12S17, 123.05 (d, $^{1}J_{CF}$ = 280.4 Hz), 122.12 (d, $^{1}J_{CF}$ = 268.1 Hz), 121.33, 120.96, 119.41, 112.58 (dd, $^{2}J_{CF}$ = 20.1, $^{4}J_{CF}$ = 4.6 Hz), 112.21 (dd, $^{2}J_{CF}$ = 21.0, $^{4}J_{CF}$ = 3.0 Hz), 102.76 (t, $^{2}J_{CF}$ = 25.8 Hz), 91.45 (t, $^{2}J_{CF}$ = 19.5 Hz), 53.06, 52.87, 51.34, 50.94, 50.77, 38.12, 30.74, 23.68, 23.00, 7.83, 7.67. HRMS found m/z 1086.1676 $[M+H]^+$, calculated for $C_{45}H_{34}ClF_{10}N_9O_6S_2$, $[M+H]^+$: 1086.1675. HPLC purity: 95.89 %.

S1.20.5 (*S*)-*N*-(1-(3-(4-chloro-3-(*N*-(ethylsulfonyl)ethylsulfonamido)-1-(2,2,2-trifluoroethyl)-1*H*-indazol-7-yl)-7-(3,5-difluorophenyl)-4-oxo-3,4-

dihydroquinazolin-2-yl)-2-(3,5-difluorophenyl)ethyl)-2-(3-(trifluoromethyl)-5,6-dihydrocyclopenta[*c*]pyrazol-1(4*H*)-yl)acetamide (IC-1f)

White solid, yield 83%. m.p. 115 – 120°C. ^{1}H NMR (600 MHz, DMSO-d_6) δ 9.03 (d, J = 8.1 Hz, 1H, NH), 8.26 (d, J = 8.3 Hz, 1H, quinazolinone-H), 8.14 (d, J = 1.8 Hz, 1H, quinazolinone-H), 8.05 (dd, J = 8.3, 1.9 Hz, 1H, quinazolinone-H), 7.87 (d, J = 7.9 Hz, 1H, indazole-H), 7.73 (d, J = 8.0 Hz, 1H, indazole-H), 7.71 – 7.65 (m, 2H, Ph-H), 7.41 (tt, J = 9.3, 2.3 Hz, 1H, Ph-H), 7.03 (tt, J = 9.4, 2.4 Hz, 1H, Ph-H), 6.66 – 6.61 (m, 2H, Ph-H), 5.02 (dq, J = 17.1, 8.6 Hz, 1H, CF_3CH_2), 4.63 (ddd, J = 10.0, 8.1, 3.8 Hz, 1H, CH), 4.54 – 4.40 (m, 3H, $COCH_2$, CF_3CH_2), 3.89 (ddq, J = 21.7, 14.9, 7.4 Hz, 2H, SO_2CH_2), 3.77 (ddq, J = 29.0, 14.8, 7.4 Hz, 2H, SO_2CH_2), 3.42 (dd, J = 14.2, 3.7 Hz, 1H, Ph-CH_2), 3.04 (dd, J = 14.2, 10.0 Hz, 1H, Ph-CH_2), 2.56 (t, J = 6.9 Hz, 2H, pyrazole-CH_2), 2.49 – 2.41 (m, 2H, CH_2), 2.41 – 2.37 (m, 2H, pyrazole-CH_2), 1.40 (t, J = 7.4 Hz, 3H, CH_3), 1.33 (t, J = 7.4 Hz, 3H, CH_3). ^{13}C NMR (150 MHz, DMSO-d_6) δ 166.38 (C=O), 163.45 (dd, $^1J_{CF}$ = 246.6, $^3J_{CF}$ = 13.3 Hz), 162.59 (dd, $^1J_{CF}$ = 246.3, $^3J_{CF}$ = 13.6 Hz), 161.48 (C=O), 156.46, 153.85, 147.20, 144.94, 142.40 (t, $^3J_{CF}$ = 9.3 Hz), 141.97 (t, $^3J_{CF}$ = 9.3 Hz), 138.92, 136.09, 134.47, 134.23, 132.32, 128.03, 127.31, 127.17, 125.71, 125.29, 12S15, 123.05 (d, $^1J_{CF}$ = 280.4 Hz), 122.12 (d, $^1J_{CF}$ = 268.3 Hz), 120.96, 120.46, 119.47, 112.57 (dd, $^2J_{CF}$ = 19.9, $^4J_{CF}$ = S1 Hz), 111.16 (dd, $^2J_{CF}$ = 20.4, $^4J_{CF}$ = 5.6 Hz), 104.72 (t, $^2J_{CF}$ = 26.5 Hz), 102.76 (t, $^2J_{CF}$ = 25.5 Hz), 53.11, 52.89, 51.35, 50.94, 50.78, 38.13, 30.73, 23.69, 23.00, 7.83, 7.67. HRMS found m/z 1061.1721 $[M+H]^+$, calculated for $C_{44}H_{35}ClF_{10}N_8O_6S_2$, $[M+H]^+$: 1061.1723. HPLC purity: 98.59 %.

S1.20.6 (*S*)-*N*-(1-(3-(4-chloro-3-(*N*-(ethylsulfonyl)ethylsulfonamido)-1-(2,2,2-trifluoroethyl)-1*H*-indazol-7-yl)-4-oxo-7-(3,4,5-trifluorophenyl)-3,4-dihydroquinazolin-2-yl)-2-(3,5-difluorophenyl)ethyl)-2-(3-(trifluoromethyl)-5,6-dihydrocyclopenta[*c*]pyrazol-1(4*H*)-yl)acetamide (IC-1g)

White solid, yield 85%. m.p. 101 – 102°C. ^{1}H NMR (600 MHz, DMSO-d_6) δ 9.00 (d, J = 8.1 Hz, 1H, NH), 8.25 (d, J = 8.3 Hz, 1H, quinazolinone-H), 8.13 (d, J = 1.2 Hz,

1H, quinazolinone-H), 8.03 (dd, J = 8.3, 1.9 Hz, 1H, quinazolinone-H), 7.95 (dd, J = 9.1, 6.6 Hz, 2H, Ph-H), 7.85 (d, J = 8.0 Hz, 1H, indazole-H), 7.71 (d, J = 7.9 Hz, 1H, indazole-H), 7.02 (tt, J = 9.3, 2.3 Hz, 1H, Ph-H), 6.67 – 6.61 (m, 2H, Ph-H), 5.00 (dq, J = 17.0, 8.6 Hz, 1H, CF_3CH_2), 4.64 (ddd, J = 10.0, 8.0, 3.8 Hz, 1H, CH), 4.52 – 4.38 (m, 3H, $COCH_2$, CF_3CH_2), 3.87 (ddt, J = 19.5, 14.8, 7.4 Hz, 2H, SO_2CH_2), 3.77 (ddq, J = 29.0, 14.6, 7.4 Hz, 2H, SO_2CH_2), 3.42 (dd, J = 14.3, 3.8 Hz, 1H, Ph-CH_2), 3.04 (dd, J = 14.2, 10.0 Hz, 1H, Ph-CH_2), 2.57 (d, J = 7.7 Hz, 2H, pyrazole-CH_2), 2.48 – 2.41 (m, 2H, CH_2), 2.40 (q, J = 4.9 Hz, 2H, pyrazole-CH_2), 1.40 (t, J = 7.4 Hz, 3H, CH_3), 1.33 (t, J = 7.4 Hz, 3H, CH_3). ^{13}C NMR (150 MHz, DMSO-d_6) δ 166.35 (C=O), 162.60 (dd, $^1J_{CF}$ = 246.3, $^3J_{CF}$ = 13.5 Hz), 161.46 (C=O), 156.50, 153.85, 147.20, 144.28, 141.97 (t, $^3J_{CF}$ = 9.4 Hz), 138.94, 136.11, 134.25, 132.28, 128.02, 127.21, 127.18, 125.68, 125.29, 12S14, 123.05 (d, $^1J_{CF}$ = 280.3 Hz), 122.13 (d, $^1J_{CF}$ = 268.5 Hz), 120.97, 120.35, 119.48, 112.75 (dd, $^2J_{CF}$ = 17.5, $^4J_{CF}$ = 4.6 Hz), 112.58 (dd, $^2J_{CF}$ = 19.8, $^4J_{CF}$ = 4.6 Hz), 102.75 (t, $^2J_{CF}$ = 2S1 Hz), 53.10, 52.86, 51.58, 50.95, 50.79, 38.16, 30.74, 23.69, 23.01, 7.82, 7.67. HRMS found m/z 1079.1630 $[M+H]^+$, calculated for $C_{44}H_{34}ClF_{11}N_8O_6S_2$, $[M+H]^+$: 1079.1628. HPLC purity: 96.56 %.

S1.20.7 (*S*)-*N*-(1-(3-(4-chloro-3-(*N*-(ethylsulfonyl)ethylsulfonamido)-1-(2,2,2-trifluoroethyl)-1*H*-indazol-7-yl)-7-(3-fluorophenyl)-4-oxo-3,4-dihydroquinazolin-2-yl)-2-(3,5-difluorophenyl)ethyl)-2-(3-(trifluoromethyl)-5,6-dihydrocyclopenta[*c*]pyrazol-1(4*H*)-yl)acetamide (IC-1h)

White solid, yield 79%. m.p. 111 – 115°C. ^{1}H NMR (600 MHz, DMSO-d_6) δ 9.02 (d, J = 8.1 Hz, 1H, NH), 8.25 (d, J = 8.2 Hz, 1H, quinazolinone-H), 8.07 (d, J = 1.8 Hz, 1H, quinazolinone-H), 8.01 (dd, J = 8.3, 1.9 Hz, 1H, quinazolinone-H), 7.85 (d, J = 7.9 Hz, 1H, indazole-H), 7.75 – 7.69 (m, 3H, indazole-H, Ph-H), 7.61 (td, J = 8.2, 6.3 Hz, 1H, Ph-H), 7.34 (td, J = 8.7, 2.6 Hz, 1H, Ph-H), 7.00 (tt, J = 9.4, 2.4 Hz, 1H, Ph-H), 6.61 (dd, J = 8.1, 2.0 Hz, 2H, Ph-H), 4.99 (dq, J = 17.0, 8.6 Hz, 1H, CF_3CH_2), 4.61 (ddd, J = 9.9, 8.1, 3.7 Hz, 1H, CH), 4.52 (d, J = 16.2 Hz, 1H, $COCH_2$), 4.47 – 4.38 (m, 2H, CF_3CH_2, $COCH_2$), 3.81 (dddt, J = 65.2, 29.3, 14.2, 7.3 Hz, 4H, $SO_2CH_2 \times 2$), 3.39

(dd, J = 14.2, 3.8 Hz, 1H, Ph-CH_2), 3.03 (dd, J = 14.2, 10.0 Hz, 1H, Ph-CH_2), 2.54 (t, J = 6.6 Hz, 2H, pyrazole-CH_2), 2.47 – 2.34 (m, 4H, pyrazole-CH_2CH_2), 1.38 (t, J = 7.4 Hz, 3H, CH_3), 1.31 (t, J = 7.4 Hz, 3H, CH_3). ^{13}C NMR (150 MHz, DMSO-d_6) δ 166.36 (C=O), 163.24 (d, $^1J_{CF}$ = 244.1 Hz), 162.56 (dd, $^1J_{CF}$ = 246.4, $^3J_{CF}$ = 13.3 Hz), 161.49 (C=O), 156.38, 153.81, 147.21, 146.12 (d, $^4J_{CF}$ = 2.0 Hz), 141.96 (t, $^3J_{CF}$ = 9.4 Hz), 141.20 (d, $^3J_{CF}$ = 7.8 Hz), 138.91, 136.05, 134.44, 132.31, 131.75 (d, $^3J_{CF}$ = 8.7 Hz), 128.00, 127.27, 127.13, 125.38, 125.26, 12S11, 123.87 (d, $^4J_{CF}$ = 2.5 Hz), 123.03 (d, $^1J_{CF}$ = 280.6 Hz), 122.10 (d, $^1J_{CF}$ = 268.4 Hz), 120.93, 120.01, 119.49, 116.18 (d, $^2J_{CF}$ = 21.2 Hz), 114.57 (d, $^2J_{CF}$ = 22.3 Hz), 112.56 (dd, $^2J_{CF}$ = 19.6, $^4J_{CF}$ = 4.8 Hz), 102.73 (t, $^2J_{CF}$ = 25.6 Hz), 55.32, 53.10, 52.84, 50.91, 50.76, 38.13, 30.71, 23.67, 22.98, 7.81, 7.65. HRMS found m/z 1043.1816 $[M+H]^+$, calculated for $C_{44}H_{36}ClF_9N_8O_6S_2$, $[M+H]^+$: 1043.1817. HPLC purity: 96.09 %.

S1.20.8 (*S*)-*N*-(1-(3-(4-chloro-3-(*N*-(ethylsulfonyl)ethylsulfonamido)-1-(2,2,2-trifluoroethyl)-1*H*-indazol-7-yl)-7-(3-fluoro-4-formylphenyl)-4-oxo-3,4-dihydroquinazolin-2-yl)-2-(3,5-difluorophenyl)ethyl)-2-(3-(trifluoromethyl)-5,6-dihydrocyclopenta[*c*]pyrazol-1(4*H*)-yl)acetamide (IC-1i)

White solid, yield 62%. m.p. 197 – 200°C. ^{1}H NMR (600 MHz, DMSO-d_6) δ 10.31 (s, 1H, CHO), 9.04 (d, J = 8.1 Hz, 1H, NH), 8.30 (d, J = 8.3 Hz, 1H, quinazolinone-H), 8.19 (d, J = 1.6 Hz, 1H, quinazolinone-H), 8.10 (dd, J = 8.3, 1.7 Hz, 1H, quinazolinone-H), 8.03 (t, J = 7.7 Hz, 1H, Ph-H), 8.00 (dd, J = 11.8, 1.1 Hz, 1H, Ph-H), 7.93 (dd, J = 7.8, 1.3 Hz, 1H, Ph-H), 7.88 (d, J = 8.0 Hz, 1H, indazole-H), 7.73 (d, J = 8.0 Hz, 1H, indazole-H), 7.03 (tt, J = 9.4, 2.4 Hz, 1H, Ph-H), 6.66 – 6.61 (m, 2H, Ph-H), 5.02 (dq, J = 17.0, 8.5 Hz, 1H, CF_3CH_2), 4.64 (ddd, J = 9.9, 8.1, 3.7 Hz, 1H, CH), 4.55 – 4.40 (m, 3H, $COCH_2$, CF_3CH_2), 3.88 (ddt, J = 19.4, 14.8, 7.4 Hz, 2H, SO_2CH_2), 3.77 (ddq, J = 29.0, 14.6, 7.4 Hz, 2H, SO_2CH_2), 3.42 (dd, J = 14.2, 3.7 Hz, 1H, Ph-CH_2), 3.05 (dd, J = 14.2, 10.0 Hz, 1H, Ph-CH_2), 2.56 (t, J = 6.7 Hz, 2H, pyrazole-CH_2), 2.48 – 2.41 (m, 2H, CH_2), 2.40 (q, J = 6.6, S1 Hz, 2H, pyrazole-CH_2), 1.40 (t, J = 7.4 Hz, 3H, CH_3), 1.33 (t, J = 7.5 Hz, 3H, CH_3). ^{13}C NMR (150 MHz, DMSO-d_6) δ 188.03 (d, $^3J_{CF}$ = 4.8

Hz, C=O), 166.38 (C=O), 164.14 (d, $^{1}J_{CF}$ = 257.8 Hz), 162.59 (dd, $^{1}J_{CF}$ = 246.3, $^{3}J_{CF}$ = 13.6 Hz), 161.47 (C=O), 156.61, 153.84, 147.22, 146.71 (d, $^{3}J_{CF}$ = 8.9 Hz), 144.84, 141.95 (t, $^{3}J_{CF}$ = 9.9 Hz), 138.91, 136.09, 132.30, 130.77, 128.16, 127.42, 127.20, 126.05, 125.29, 12S15, 124.41 (d, $^{4}J_{CF}$ = 3.2 Hz), 124.06 (d, $^{3}J_{CF}$ = 8.9 Hz), 123.05 (d, $^{1}J_{CF}$ = 280.4 Hz), 122.12 (d, $^{1}J_{CF}$ = 268.0 Hz), 120.97, 120.77, 119.45, 116.03 (d, $^{2}J_{CF}$ = 22.1 Hz), 112.58 (dd, $^{2}J_{CF}$ = 19.8, $^{4}J_{CF}$ = 4.5 Hz), 102.76 (t, $^{2}J_{CF}$ = 25.6 Hz), 53.11, 52.86, 51.37, 50.94, 50.78, 38.16, 30.74, 23.69, 23.01, 7.83, 7.68. HRMS found m/z 1071.1771 $[M+H]^+$, calculated for $C_{45}H_{36}ClF_9N_8O_7S_2$, $[M+H]^+$: 1071.1766. HPLC purity: 99.62 %.

S1.20.9 (*S*)-*N*-(1-(3-(4-chloro-3-(*N*-(ethylsulfonyl)ethylsulfonamido)-1-(2,2,2-trifluoroethyl)-1*H*-indazol-7-yl)-7-(4-nitrophenyl)-4-oxo-3,4-dihydroquinazolin-2-yl)-2-(3,5-difluorophenyl)ethyl)-2-(3-(trifluoromethyl)-5,6-dihydrocyclopenta[*c*]pyrazol-1(4*H*)-yl)acetamide (IC-1j)

Yellow solid, yield 52%. m.p. 229 – 231°C. ^{1}H NMR (600 MHz, DMSO-d_6) δ 9.03 (d, J = 8.1 Hz, 1H, NH), 8.41 (d, J = 8.8 Hz, 2H, Ph-H), 8.32 (d, J = 8.2 Hz, 1H, quinazolinone-H), 8.20 – 8.14 (m, 3H, quinazolinone-H, Ph-H), 8.08 (dd, J = 8.3, 1.8 Hz, 1H, quinazolinone-H), 7.86 (d, J = 8.0 Hz, 1H, indazole-H), 7.72 (d, J = 8.0 Hz, 1H, indazole-H), 7.02 (tt, J = 9.3, 2.4 Hz, 1H, Ph-H), 6.66 – 6.61 (m, 2H, Ph-H), 4.99 (dq, J = 17.0, 8.6 Hz, 1H, CF_3CH_2), 4.64 (ddd, J = 10.0, 8.1, 3.8 Hz, 1H, CH), 4.55 – 4.40 (m, 3H, $COCH_2$, CF_3CH_2), 3.88 (dp, J = 23.1, 7.4 Hz, 2H, SO_2CH_2), 3.77 (ddt, J = 30.6, 14.2, 7.3 Hz, 2H, SO_2CH_2), 3.41 (dd, J = 14.3, 3.8 Hz, 1H, Ph-CH_2), 3.05 (dd, J = 14.2, 10.0 Hz, 1H, Ph-CH_2), 2.56 (t, J = 6.8 Hz, 2H, pyrazole-CH_2), 2.48 – 2.41 (m, 2H, CH_2), 2.41 – 2.37 (m, 2H, pyrazole-CH_2), 1.40 (t, J = 7.4 Hz, 3H, CH_3), 1.33 (t, J = 7.4 Hz, 3H, CH_3). ^{13}C NMR (150 MHz, DMSO-d_6) δ 166.37 (C=O), 162.60 (dd, $^{1}J_{CF}$ = 246.4, $^{3}J_{CF}$ = 13.1 Hz), 161.48 (C=O), 156.71, 153.83, 148.14, 147.26, 14S19, 14S13, 141.94 (t, $^{3}J_{CF}$ = 9.6 Hz), 138.93, 136.11, 132.27, 129.19, 128.27, 127.48, 127.22, 126.08, 125.28, 12S13, 124.77, 123.05 (d, $^{1}J_{CF}$ = 280.3 Hz), 122.12 (d, $^{1}J_{CF}$ = 268.0 Hz), 120.99, 120.68, 119.46, 112.58 (dd, $^{2}J_{CF}$ = 19.8, $^{4}J_{CF}$ = 4.8 Hz), 102.75 (t, $^{2}J_{CF}$ =

25.8 Hz), 53.13, 52.82, 50.95, 50.79, 40.63, 38.21, 30.74, 23.69, 23.01, 7.83, 7.67. HRMS found m/z 1070.1763 $[M+H]^+$, calculated for $C_{44}H_{36}ClF_8N_9O_8S_2$, $[M+H]^+$: 1070.1762. HPLC purity: 92.48 %.

S1.20.10 (*S*)-N-(1-(3-(4-chloro-3-(*N*-(ethylsulfonyl)ethylsulfonamido)-1-(2,2,2-trifluoroethyl)-1*H*-indazol-7-yl)-7-(3-fluoro-4-nitrophenyl)-4-oxo-3,4-dihydroquinazolin-2-yl)-2-(3,5-difluorophenyl)ethyl)-2-(3-(trifluoromethyl)-5,6-dihydrocyclopenta[*c*]pyrazol-1(4*H*)-yl)acetamide (IC-1k)

Yellow solid, yield 67%. m.p. > 250°C. ^{1}H NMR (600 MHz, DMSO-d_6) δ 9.02 (d, J = 8.1 Hz, 1H, NH), 8.33 (t, J = 8.3 Hz, 1H, Ph-H), 8.31 (d, J = 8.2 Hz, 1H, quinazolinone-H), 8.22 (d, J = 1.8 Hz, 1H, quinazolinone-H), 8.19 (dd, J = 12.5, 1.9 Hz, 1H, Ph-H), 8.11 (dd, J = 8.3, 1.8 Hz, 1H, quinazolinone-H), 7.98 (dd, J = 8.5, 1.4 Hz, 1H, Ph-H), 7.86 (d, J = 8.0 Hz, 1H, indazole-H), 7.72 (d, J = 7.9 Hz, 1H, indazole-H), 7.02 (tt, J = 9.4, 2.3 Hz, 1H, Ph-H), 6.67 – 6.62 (m, 2H, Ph-H), 5.01 (dq, J = 17.0, 8.6 Hz, 1H, CF_3CH_2), 4.65 (ddd, J = 9.9, 8.0, 3.7 Hz, 1H, CH), 4.53 – 4.39 (m, 3H, CF_3CH_2, $COCH_2$), 3.88 (ddq, J = 18.4, 14.8, 7.4 Hz, 2H, SO_2CH_2), 3.77 (ddq, J = 29.0, 14.6, 7.4 Hz, 2H, SO_2CH_2), 3.45 – 3.40 (m, 1H, Ph-CH_2), 3.05 (dd, J = 14.2, 10.0 Hz, 1H, Ph-CH_2), 2.56 (d, J = 6.1 Hz, 2H, pyrazole-CH_2), 2.49 – 2.42 (m, 2H, CH_2), 2.40 (dd, J = 6.7, 3.9 Hz, 2H, pyrazole-CH_2), 1.40 (t, J = 7.4 Hz, 3H, CH_3), 1.33 (t, J = 7.4 Hz, 3H, CH_3). ^{13}C NMR (150 MHz, DMSO-d_6) δ 166.36 (C=O), 162.60 (dd, $^1J_{CF}$ = 246.3, $^3J_{CF}$ = 13.6 Hz), 161.45 (C=O), 156.71, 155.54 (d, $^1J_{CF}$ = 261.3 Hz), 153.85, 147.21, 146.55 (d, $^2J_{CF}$ = 8.6 Hz), 143.96, 141.94 (t, $^3J_{CF}$ = 9.0 Hz), 138.92, 137.15 (d, $^2J_{CF}$ = 7.7 Hz), 136.12, 134.49, 134.24, 132.26, 128.22, 127.52 (d, $^3J_{CF}$ = 4.3 Hz), 127.22, 126.33, 125.29, 12S15, 124.49 (d, $^4J_{CF}$ = 3.7 Hz), 123.05 (d, $^1J_{CF}$ = 280.3 Hz), 122.12 (d, $^1J_{CF}$ = 268.1 Hz), 121.00 (d, $^3J_{CF}$ = 4.0 Hz), 119.44, 117.77 (d, $^2J_{CF}$ = 22.2 Hz), 112.58 (dd, $^2J_{CF}$ = 19.9, $^4J_{CF}$ = 4.7 Hz), 102.76 (t, $^2J_{CF}$ = 25.6 Hz), 53.10, 52.84, 51.37, 50.95, 50.79, 38.18, 30.74, 23.69, 23.01, 7.83, 7.67. HRMS found m/z 1088.1670 $[M+H]^+$, calculated for $C_{44}H_{35}ClF_9N_9O_8S_2$, $[M+H]^+$: 1088.1668. HPLC purity: 99.81 %.

S1.21 General procedure for the synthesis of IC-2(b – k)

Dissolve **IC-1(b – k)** (1.0 eq.) and K_2CO_3 (3.0 eq.) in 10 mL DMF and 1 mL water. Then raise the temperature to 75 °C and stir for 6 h (monitored by TLC). Once the reaction was complete, add 20 mL of ethyl acetate and 30 mL of saturated NaCl to the mixture. Next, separate the organic phase and wash the aqueous phase with ethyl acetate. Combine the organic phases, dry them with sodium sulfate, and filter. Remove the organic solvents under reduced pressure and the residue was purified by column chromatography. Finally, crystallize the product from a hybrid solvent of ethyl acetate and petroleum ether to obtain the target compound **IC-2(b – k)**.

S1.21.1 (*S*)-*N*-(1-(3-(4-chloro-3-(ethylsulfonamido)-1-(2,2,2-trifluoroethyl)-1*H*-indazol-7-yl)-4-oxo-7-phenyl-3,4-dihydroquinazolin-2-yl)-2-(3,5-difluorophenyl)ethyl)-2-(3-(trifluoromethyl)-5,6-dihydrocyclopenta[*c*]pyrazol-1(4*H*)-yl)acetamide (IC-2b)

White solid, yield 96%. m.p. 120 – 124°C. ^{1}H NMR (600 MHz, DMSO-d_6) δ 10.08 (s, 1H, SO_2NH), 9.14 (d, J = 8.4 Hz, 1H, NH), 8.27 (d, J = 8.3 Hz, 1H, quinazolinone-H), 8.05 (d, J = 1.7 Hz, 1H, quinazolinone-H), 7.99 (dd, J = 8.3, 1.8 Hz, 1H, quinazolinone-H), 7.88 (d, J = 7.0 Hz, 2H, Ph-H), 7.78 (d, J = 8.0 Hz, 1H, indazole-H), 7.61 – 7.56 (m, 3H, indazole-H, Ph-H), 7.52 (t, J = 7.4 Hz, 1H, Ph-H), 7.02 (tt, J = 9.4, 2.4 Hz, 1H, Ph-H), 6.66 – 6.61 (m, 2H, Ph-H), 4.78 (dq, J = 17.0, 8.6 Hz, 1H, CF_3CH_2), 4.62 – 4.55 (m, 3H, $COCH_2$, CH), 4.26 (dq, J = 16.9, 8.6 Hz, 1H, CF_3CH_2), 3.40 (dd, J = 14.2, 3.6 Hz, 1H, Ph-CH_2), 3.35 (qd, J = 7.2, 2.0 Hz, 2H, SO_2CH_2), 3.04 (dd, J = 14.2, 10.2 Hz, 1H, Ph-CH_2), 2.55 (t, J = 7.1 Hz, 2H, pyrazole-CH_2), 2.47 – 2.44 (m, 2H, CH_2), 2.43 – 2.37 (m, 2H, pyrazole-CH_2), 1.34 (t, J = 7.4 Hz, 3H, CH_3). ^{13}C NMR (150 MHz, DMSO-d_6) δ 166.32 (C=O), 162.59 (dd, $^{1}J_{CF}$ = 246.3, $^{3}J_{CF}$ = 12.9 Hz), 161.43 (C=O), 156.76, 153.78, 147.58, 147.39, 141.93 (t, $^{3}J_{CF}$ = 9.6 Hz), 140.19, 138.85, 138.53, 134.38, 134.13, 131.85, 129.81, 129.44, 128.31, 128.01, 127.71, 127.17, 12S14, 124.97, 123.53, 123.22 (d, $^{1}J_{CF}$ = 280.7 Hz), 122.12 (d, $^{1}J_{CF}$ = 268.1 Hz), 119.68, 119.50, 118.91, 112.54 (dd, $^{2}J_{CF}$ = 19.8, $^{4}J_{CF}$ = 4.9 Hz), 102.75 (t, $^{2}J_{CF}$ = 25.8 Hz), 53.11, 52.62, 48.15, 38.51, 30.75, 23.69, 23.01, 14.38, 8.36. HRMS found m/z 933.1976 $[M+H]^+$,

calculated for $C_{42}H_{33}ClF_8N_8O_4S$, $[M+H]^+$: 933.1979. HPLC purity: 97.28 %. Elemental analyses found: C, 53.757; H, 3.749; N, 11.504; S, 3.379 %. Calc. for $C_{42}H_{33}ClF_8N_8O_4S$: C, 54.05; H, 3.56; N, 12.01; S, 3.44 %.

S1.21.2 (*S*)-*N*-(1-(3-(4-chloro-3-(ethylsulfonamido)-1-(2,2,2-trifluoroethyl)-1*H*-indazol-7-yl)-7-(4-cyanophenyl)-4-oxo-3,4-dihydroquinazolin-2-yl)-2-(3,5-difluorophenyl)ethyl)-2-(3-(trifluoromethyl)-5,6-dihydrocyclopenta[*c*]pyrazol-1(4*H*)-yl)acetamide (IC-2c)

White solid, yield 70%. m.p. 150 – 152℃. ^{1}H NMR (600 MHz, DMSO-d_6) δ 10.08 (s, 1H, SO_2NH), 9.14 (d, J = 8.3 Hz, 1H, NH), 8.31 (d, J = 8.2 Hz, 1H, quinazolinone-H), 8.13 (d, J = 1.3 Hz, 1H, quinazolinone-H), 8.10 (d, J = 8.4 Hz, 2H, Ph-H), 8.07 – 8.03 (m, 3H, quinazolinone-H, Ph-H), 7.77 (d, J = 7.9 Hz, 1H, indazole-H), 7.58 (d, J = 7.9 Hz, 1H, indazole-H), 7.02 (tt, J = 9.4, 2.4 Hz, 1H, Ph-H), 6.66 – 6.61 (m, 2H, Ph-H), 4.79 (dq, J = 16.9, 8.6 Hz, 1H, CF_3CH_2), 4.60 (ddd, J = 10.0, 8.4, 3.6 Hz, 1H, CH), 4.57 (s, 2H, $COCH_2$), 4.26 (dq, J = 16.9, 8.6 Hz, 1H, CF_3CH_2), 3.40 (dd, J = 14.2, 3.6 Hz, 1H, Ph-CH_2), 3.35 (qd, J = 7.1, 2.6 Hz, 2H, SO_2CH_2), 3.04 (dd, J = 14.2, 10.2 Hz, 1H, Ph-CH_2), 2.55 (t, J = 6.9 Hz, 2H, pyrazole-CH_2), 2.48 – 2.43 (m, 2H, CH_2), 2.41 (q, J = 6.3 Hz, 2H, pyrazole-CH_2), 1.33 (t, J = 7.3 Hz, 3H, CH_3). ^{13}C NMR (150 MHz, DMSO-d_6) δ 166.33 (C=O), 162.60 (dd, $^1J_{CF}$ = 246.4, $^3J_{CF}$ = 13.4 Hz), 161.36 (C=O), 157.02, 153.80, 147.35, 145.61, 143.31, 141.89 (t, $^3J_{CF}$ = 9.2 Hz), 138.49, 134.38, 133.61, 131.82, 128.76, 128.38, 128.23, 127.34, 125.78, 12S14, 123.53, 123.22 (d, $^1J_{CF}$ = 280.8 Hz), 122.11 (d, $^1J_{CF}$ = 268.1 Hz), 120.59, 119.52, 119.05, 118.80, 112.53 (dd, $^2J_{CF}$ = 19.9, $^4J_{CF}$ = 4.9 Hz), 112.01, 102.76 (t, $^2J_{CF}$ = 26.0 Hz), 53.08, 52.64, 48.13, 38.47, 30.75, 23.68, 23.00, 22.50, 8.37. HRMS found m/z 958.1936 $[M+H]^+$, calculated for $C_{43}H_{32}ClF_8N_9O_4S$, $[M+H]^+$: 958.1932. HPLC purity: 99.60 %.

S1.21.3 (*S*)-*N*-(1-(3-(4-chloro-3-(ethylsulfonamido)-1-(2,2,2-trifluoroethyl)-1*H*-indazol-7-yl)-7-(4-cyano-3-fluorophenyl)-4-oxo-3,4-dihydroquinazolin-2-yl)-2-(3,5-difluorophenyl)ethyl)-2-(3-(trifluoromethyl)-5,6-dihydrocyclopenta[*c*]pyrazol-1(4*H*)-yl)acetamide (IC-2d)

White solid, yield 41%. m.p. 116 – 119°C. ^{1}H NMR (600 MHz, DMSO-d_6) δ 10.08 (s, 1H, SO_2NH), 9.13 (s, 1H, NH), 8.31 (d, J = 8.3 Hz, 1H, quinazolinone-H), 8.18 (d, J = 1.8 Hz, 1H, quinazolinone-H), 8.15 – 8.12 (m, 1H, Ph-H), 8.12 – 8.11 (m, 1H, Ph-H), 8.08 (dd, J = 8.3, 1.9 Hz, 1H, quinazolinone-H), 7.96 (dd, J = 8.1, 1.7 Hz, 1H, Ph-H), 7.77 (d, J = 7.9 Hz, 1H, indazole-H), 7.58 (d, J = 7.9 Hz, 1H, indazole-H), 7.02 (tt, J = 9.4, 2.4 Hz, 1H, Ph-H), 6.67 – 6.61 (m, 2H, Ph-H), 4.80 (dq, J = 16.9, 8.6 Hz, 1H, CF_3CH_2), 4.61 (ddd, J = 10.1, 8.3, 3.7 Hz, 1H, CH), 4.55 (d, J = 1.7 Hz, 2H, $COCH_2$), 4.26 (dq, J = 16.9, 8.6 Hz, 1H, CF_3CH_2), 3.41 (dd, J = 14.2, 3.6 Hz, 1H, Ph-CH_2), 3.35 (qd, J = 7.1, 2.7 Hz, 2H, SO_2CH_2), 3.03 (dd, J = 14.2, 10.1 Hz, 1H, Ph-CH_2), 2.55 (t, J = 7.0 Hz, 2H, pyrazole-CH_2), 2.43 (dq, J = 18.3, 6.5 Hz, 4H, pyrazole-CH_2, CH_2), 1.33 (t, J = 7.3 Hz, 3H, CH_3). ^{13}C NMR (150 MHz, DMSO-d_6) δ 166.33 (C=O), 163.42 (d, $^1J_{CF}$ = 255.7 Hz), 162.60 (dd, $^1J_{CF}$ = 246.4, $^3J_{CF}$ = 13.3 Hz), 161.33 (C=O), 157.08, 153.82, 147.31, 146.31 (d, $^3J_{CF}$ = 8.7 Hz), 144.34, 141.87 (d, $^2J_{CF}$ = 19.1 Hz), 140.22, 138.48, 13S11, 131.83, 128.38, 128.22, 127.42, 126.15, 12S14, 124.87 (d, $^4J_{CF}$ = 3.3 Hz), 123.58, 123.22 (d, $^1J_{CF}$ = 280.8 Hz), 122.11 (d, $^1J_{CF}$ = 267.8 Hz), 121.02, 119.51, 118.78, 115.86 (d, $^2J_{CF}$ = 20.9 Hz), 114.35, 112.53 (dd, $^2J_{CF}$ = 19.8, $^4J_{CF}$ = S1 Hz), 102.77 (t, $^2J_{CF}$ = 26.1 Hz), 100.66 (d, $^2J_{CF}$ = 15.2 Hz), 53.06, 52.66, 48.16, 38.45, 30.75, 23.69, 23.00, 8.36. HRMS found m/z 976.1834 $[M+H]^+$, calculated for $C_{43}H_{31}ClF_9N_9O_4S$, $[M+H]^+$: 976.1837. HPLC purity: 99.60 %. Elemental analyses found: C, 53.148; H, 3.541; N, 11.981; S, 3.186 %. Calc. for $C_{43}H_{31}ClF_9N_9O_4S$: C, 52.90; H, 3.20; N, 12.91; S, 3.28 %.

S1.21.4 (*S*)-*N*-(1-(3-(4-chloro-3-(*N*-(ethylsulfonyl)ethylsulfonamido)-1-(2,2,2-trifluoroethyl)-1*H*-indazol-7-yl)-7-(4-cyano-3,5-difluorophenyl)-4-oxo-3,4-dihydroquinazolin-2-yl)-2-(3,5-difluorophenyl)ethyl)-2-(3-(trifluoromethyl)-5,6-dihydrocyclopenta[*c*]pyrazol-1(4*H*)-yl)acetamide(IC-2e)

White solid, yield 46%. m.p. 107 – 108°C. ^{1}H NMR (600 MHz, DMSO-d_6) δ 10.08 (s, 1H, SO_2NH), 9.10 (d, J = 8.3 Hz, 1H, NH), 8.31 (d, J = 8.3 Hz, 1H, quinazolinone-H), 8.22 (d, J = 1.8 Hz, 1H, quinazolinone-H), 8.10 (dd, J = 8.3, 1.9 Hz, 1H, quinazolinone-

H), 8.07 (d, J = 9.6 Hz, 2H, Ph-H), 7.76 (d, J = 7.9 Hz, 1H, indazole-H), 7.57 (d, J = 7.9 Hz, 1H, indazole-H), 7.02 (tt, J = 9.4, 2.4 Hz, 1H, Ph-H), 6.67 – 6.60 (m, 2H, Ph-H), 4.81 (dq, J = 16.9, 8.7 Hz, 1H, CF_3CH_2), 4.61 (ddd, J = 10.0, 8.3, 3.7 Hz, 1H, CH), 4.58 – 4.49 (m, 2H, $COCH_2$), 4.25 (dq, J = 16.9, 8.6 Hz, 1H, CF_3CH_2), 3.41 (dd, J = 14.2, 3.7 Hz, 1H, Ph-CH_2), 3.34 (qd, J = 7.1, 3.4 Hz, 2H, SO_2CH_2), 3.03 (dd, J = 14.2, 10.1 Hz, 1H, Ph-CH_2), 2.55 (t, J = 6.8 Hz, 2H, pyrazole-CH_2), 2.46 – 2.37 (m, 4H, pyrazole-CH_2, CH_2), 1.33 (t, J = 7.3 Hz, 3H, CH_3). ^{13}C NMR (150 MHz, DMSO-d_6) δ 166.29 (C=O), 163.22 (dd, $^1J_{CF}$ = 257.7, $^3J_{CF}$ = 5.5 Hz), 162.57 (dd, $^1J_{CF}$ = 246.1, $^3J_{CF}$ = 12.9 Hz), 161.27 (C=O), 157.12, 153.82, 147.34, 147.24, 143.28, 140.18, 138.42, 134.08, 131.79, 130.09, 128.37, 128.19, 127.40, 126.41, 12S11, 123.19 (d, $^1J_{CF}$ = 280.8 Hz), 122.08 (d, $^1J_{CF}$ = 268.1 Hz), 121.40, 119.47, 118.69, 116.80, 112.50 (dd, $^2J_{CF}$ = 19.6, $^4J_{CF}$ = 4.8 Hz), 112.20 (dd, $^2J_{CF}$ = 21.1, $^4J_{CF}$ = 3.0 Hz), 110.05, 102.77 (t, $^2J_{CF}$ = 25.7 Hz), 91.41 (t, $^2J_{CF}$ = 19.7 Hz), 52.99, 52.64, 48.09, 40.55, 38.38, 30.72, 23.65, 22.98, 8.35. HRMS found m/z 994.1748 $[M+H]^+$, calculated for $C_{43}H_{30}ClF_{10}N_9O_4S$, $[M+H]^+$: 994.1743. HPLC purity: 99.14 %.

S1.21.5 (*S*)-*N*-(1-(3-(4-chloro-3-(ethylsulfonamido)-1-(2,2,2-trifluoroethyl)-1*H*-indazol-7-yl)-7-(3,5-difluorophenyl)-4-oxo-3,4-dihydroquinazolin-2-yl)-2-(3,5-difluorophenyl)ethyl)-2-(3-(trifluoromethyl)-5,6-dihydrocyclopenta[*c*]pyrazol-1(4*H*)-yl)acetamide (IC-2f)

White solid, yield 78%. m.p. 110 – 114°C. ^{1}H NMR (600 MHz, DMSO-d_6) δ 10.08 (s, 1H, SO_2NH), 9.11 (d, J = 8.3 Hz, 1H, NH), 8.27 (d, J = 8.3 Hz, 1H, quinazolinone-H), 8.11 (d, J = 1.8 Hz, 1H, quinazolinone-H), 8.04 (dd, J = 8.3, 1.8 Hz, 1H, quinazolinone-H), 7.77 (d, J = 7.9 Hz, 1H, indazole-H), 7.70 – 7.65 (m, 2H, Ph-H), 7.58 (d, J = 7.9 Hz, 1H, indazole-H), 7.40 (tt, J = 9.2, 2.3 Hz, 1H, Ph-H), 7.02 (tt, J = 9.4, 2.4 Hz, 1H, Ph-H), 6.67 – 6.60 (m, 2H, Ph-H), 4.80 (dq, J = 17.0, 8.6 Hz, 1H, CF_3CH_2), 4.59 (ddd, J = 10.1, 8.3, 3.6 Hz, 1H, CH), 4.56 (s, 2H, $COCH_2$), 4.25 (dq, J = 16.9, 8.6 Hz, 1H, CF_3CH_2), 3.40 (dd, J = 14.2, 3.7 Hz, 1H, Ph-CH_2), 3.35 (qd, J = 7.2, 2.7 Hz, 2H, SO_2CH_2), 3.03 (dd, J = 14.2, 10.2 Hz, 1H, Ph-CH_2), 2.55 (t, J = 7.1 Hz, 2H, pyrazole-

CH_2), 2.44 (d, *J* = 6.0 Hz, 2H, CH_2), 2.40 (dt, *J* = 10.7, 4.9 Hz, 2H, pyrazole-CH_2), 1.33 (t, *J* = 7.3 Hz, 3H, CH_3). ^{13}C NMR (150 MHz, DMSO-d_6) δ 166.33 (C=O), 163.46 (dd, $^1J_{CF}$ = 248.0, $^3J_{CF}$ = 14.0 Hz), 162.56 (dd, $^1J_{CF}$ = 233.3, $^3J_{CF}$ = 13.4 Hz), 161.35 (C=O), 156.91, 153.82, 147.31, 138.50, 131.87, 128.35, 128.05, 127.28, 125.64, 12S14, 123.57, 122.12 (d, $^1J_{CF}$ = 268.1 Hz), 121.47 (d, $^1J_{CF}$ = 250.0 Hz), 120.57, 119.50, 118.81, 112.53 (dd, $^2J_{CF}$ = 19.5, $^4J_{CF}$ = 4.5 Hz), 111.15 (dd, $^2J_{CF}$ = 20.9, $^4J_{CF}$ = 5.4 Hz), 102.77, 53.08, 52.67, 48.16, 40.62, 38.44, 30.75, 23.69, 23.00, 8.36. HRMS found m/z 969.1793 $[M+H]^+$, calculated for $C_{42}H_{31}ClF_{10}N_8O_4S$, $[M+H]^+$: 969.1791. HPLC purity: 95.58 %.

S1.21.6 (*S*)-*N*-(1-(3-(4-chloro-3-(*N*-(ethylsulfonyl)ethylsulfonamido)-1-(2,2,2-trifluoroethyl)-1*H*-indazol-7-yl)-4-oxo-7-(3,4,5-trifluorophenyl)-3,4-dihydroquinazolin-2-yl)-2-(3,5-difluorophenyl)ethyl)-2-(3-(trifluoromethyl)-5,6-dihydrocyclopenta[*c*]pyrazol-1(4*H*)-yl)acetamide (IC-2g)

White solid, yield 99%. m.p. 112 – 115°C. ^{1}H NMR (600 MHz, DMSO-d_6) δ 10.08 (s, 1H, SO_2NH), 9.11 (d, *J* = 8.3 Hz, 1H, NH), 8.27 (d, *J* = 8.3 Hz, 1H, quinazolinone-H), 8.12 (d, *J* = 1.9 Hz, 1H, quinazolinone-H), 8.03 (dd, *J* = 8.3, 1.9 Hz, 1H, quinazolinone-H), 7.95 (dd, *J* = 9.0, 6.6 Hz, 2H, Ph-H), 7.76 (d, *J* = 7.9 Hz, 1H, indazole-H), 7.57 (d, *J* = 7.9 Hz, 1H, indazole-H), 7.01 (tt, *J* = 9.4, 2.4 Hz, 1H, Ph-H), 6.68 – 6.60 (m, 2H, Ph-H), 4.80 (dq, *J* = 16.8, 8.5 Hz, 1H, CF_3CH_2), 4.61 (ddd, *J* = 10.1, 8.3, 3.7 Hz, 1H, CH), 4.59 – 4.52 (m, 2H, $COCH_2$), 4.25 (dq, *J* = 16.8, 8.6 Hz, 1H, CF_3CH_2), 3.41 (dd, *J* = 14.2, 3.7 Hz, 1H, Ph-CH_2), 3.35 (qd, *J* = 7.2, 2.8 Hz, 2H, SO_2CH_2), 3.04 (dd, *J* = 14.2, 10.1 Hz, 1H, Ph-CH_2), 2.56 (t, *J* = 6.9 Hz, 2H, pyrazole-CH_2), 2.46 (dd, *J* = 11.4, 4.1 Hz, 2H, CH_2), 2.41 (dt, *J* = 10.5, 4.8 Hz, 2H, pyrazole-CH_2), 1.34 (t, *J* = 7.3 Hz, 3H, CH_3). ^{13}C NMR (150 MHz, DMSO-d_6) δ 166.31 (C=O), 162.61 (dd, $^1J_{CF}$ = 246.5, $^3J_{CF}$ = 13.3 Hz), 161.34 (C=O), 156.94, 153.83, 151.24 (ddd, $^1J_{CF}$ = 247.3, $^2J_{CF}$ = 9.2, $^3J_{CF}$ =3.4 Hz), 147.30, 144.23, 141.89 (t, $^3J_{CF}$ = 9.2 Hz), 140.25, 138.89 (t, $^2J_{CF}$ = 14.8 Hz), 138.51, 135.62, 134.40, 134.15, 131.82, 128.36, 128.05, 127.18, 125.61, 12S13, 123.54, 123.22 (d, $^1J_{CF}$ = 280.7 Hz), 122.11 (d, $^1J_{CF}$ = 268.2 Hz), 120.46, 119.50,

118.81, 112.64 (ddd, ${}^2J_{CF}$ = 32.5, ${}^3J_{CF}$ = 18.6, ${}^4J_{CF}$ = 4.6 Hz), 102.76 (t, ${}^2J_{CF}$ = 25.7 Hz), 53.05, 52.66, 48.15, 38.46, 36.21, 30.75, 23.69, 23.00, 8.35. HRMS found m/z 987.1700 $[M+H]^+$, calculated for $C_{42}H_{30}ClF_{11}N_8O_4S$, $[M+H]^+$: 987.1696. HPLC purity: 95.42 %. Elemental analyses found: C, 50.318; H, 3.171; N, 10.888; S, 3.652 %. Calc. for $C_{42}H_{30}ClF_{11}N_8O_4S$: C, 51.10; H, 3.06; N, 11.35; S, 3.25 %.

S1.21.7 (*S*)-*N*-(1-(3-(4-chloro-3-(ethylsulfonamido)-1-(2,2,2-trifluoroethyl)-1*H*-indazol-7-yl)-7-(3-fluorophenyl)-4-oxo-3,4-dihydroquinazolin-2-yl)-2-(3,5-difluorophenyl)ethyl)-2-(3-(trifluoromethyl)-5,6-dihydrocyclopenta[*c*]pyrazol-1(4*H*)-yl)acetamide (IC-2h)

White solid, yield 70%. m.p. 115 – 118°C. ^{1}H NMR (600 MHz, DMSO-d_6) δ 10.08 (s, 1H, SO_2NH), 9.13 (d, J = 8.4 Hz, 1H, NH), 8.27 (d, J = 8.2 Hz, 1H, quinazolinone-H), 8.08 (d, J = 1.8 Hz, 1H, quinazolinone-H), 8.02 (dd, J = 8.3, 1.8 Hz, 1H, quinazolinone-H), 7.77 (d, J = 7.9 Hz, 1H, indazole-H), 7.76 – 7.72 (m, 2H, Ph-H), 7.63 (td, J = 8.1, 6.1 Hz, 1H, Ph-H), 7.58 (d, J = 7.9 Hz, 1H, indazole-H), 7.38 – 7.33 (m, 1H, Ph-H), 7.02 (tt, J = 9.3, 2.3 Hz, 1H, Ph-H), 6.66 – 6.61 (m, 2H, Ph-H), 4.79 (dq, J = 16.9, 8.6 Hz, 1H, CF_3CH_2), 4.61 – 4.58 (m, 1H, CH), 4.57 (s, 2H, $COCH_2$), 4.26 (dq, J = 16.9, 8.6 Hz, 1H, CF_3CH_2), 3.40 (dd, J = 14.2, 3.6 Hz, 1H, Ph-CH_2), 3.35 (qd, J = 7.2, 2.3 Hz, 2H, SO_2CH_2), 3.03 (dd, J = 14.2, 10.1 Hz, 1H, Ph-CH_2), 2.55 (t, J = 7.0 Hz, 2H, pyrazole-CH_2), 2.45 (dt, J = 6.9, 3.3 Hz, 2H, CH_2), 2.40 (dt, J = 10.1, 4.8 Hz, 2H, pyrazole-CH_2), 1.33 (t, J = 7.3 Hz, 3H, CH_3). ^{13}C NMR (150 MHz, DMSO-d_6) δ 166.33 (C=O), 163.27 (d, ${}^1J_{CF}$ = 244.3 Hz), 162.60 (dd, ${}^1J_{CF}$ = 246.1, ${}^3J_{CF}$ = 13.2 Hz), 161.38 (C=O), 156.85, 153.80, 147.35, 146.11 (d, ${}^4J_{CF}$ = 2.1 Hz), 141.91 (t, ${}^3J_{CF}$ = 9.5 Hz), 141.27 (d, ${}^3J_{CF}$ = 7.6 Hz), 138.51, 131.83 (d, ${}^3J_{CF}$ = 6.5 Hz), 131.76, 128.34, 128.05, 127.25, 125.33, 12S14, 123.89, 123.87, 123.54, 123.22 (d, ${}^1J_{CF}$ = 280.8 Hz), 122.12 (d, ${}^1J_{CF}$ = 267.7 Hz), 120.14, 119.50, 118.85, 116.19 (d, ${}^2J_{CF}$ = 21.2 Hz), 114.65, 114.50, 112.53 (dd, ${}^2J_{CF}$ = 19.9, ${}^4J_{CF}$ = 4.7 Hz), 102.76 (t, ${}^2J_{CF}$ = 25.9 Hz), 53.10, 52.64, 51.04, 48.15, 38.48, 30.75, 23.69, 23.00, 8.36. HRMS found m/z 951.1885 $[M+H]^+$, calculated for $C_{42}H_{32}ClF_9N_8O_4S$, $[M+H]^+$: 951.1885. HPLC purity: 99.65 %.

S1.21.8 (*S*)-*N*-(1-(3-(4-chloro-3-(ethylsulfonamido)-1-(2,2,2-trifluoroethyl)-1*H*-indazol-7-yl)-7-(3-fluoro-4-formylphenyl)-4-oxo-3,4-dihydroquinazolin-2-yl)-2-(3,5-difluorophenyl)ethyl)-2-(3-(trifluoromethyl)-5,6-dihydrocyclopenta[*c*]pyrazol-1(4*H*)-yl)acetamide (IC-2i)

White solid, yield 77%. m.p. 140 – 142°C. ^{1}H NMR (600 MHz, Chloroform-*d*) δ 10.45 (s, 1H, CHO), 8.38 (d, *J* = 8.2 Hz, 1H, quinazolinone-H), 8.06 (t, *J* = 7.5 Hz, 1H, Ph-H), 8.02 (d, *J* = 1.8 Hz, 1H, quinazolinone-H), 7.83 (dd, *J* = 8.3, 1.8 Hz, 1H, quinazolinone-H), 7.67 (dd, *J* = 8.0, 1.7 Hz, 1H, Ph-H), 7.57 (dd, *J* = 11.1, 1.7 Hz, 1H, Ph-H), 7.54 (d, *J* = 8.1 Hz, 1H, indazole-H), 7.50 (s, 1H, SO_2NH), 7.26 (s, 1H, NH), 6.86 (d, *J* = 7.9 Hz, 1H, indazole-H), 6.68 (tt, *J* = 8.8, 2.3 Hz, 1H, Ph-H), 6.31 (h, *J* = 4.9 Hz, 2H, Ph-H), 4.64 (td, *J* = 7.8, 6.1 Hz, 1H, CH), 4.59 – 4.47 (m, 2H, $COCH_2$), 4.43 (dq, *J* = 16.3, 8.1 Hz, 1H, CF_3CH_2), 4.24 (dq, *J* = 16.2, 8.1 Hz, 1H, CF_3CH_2), 3.58 (dp, *J* = 21.8, 7.3 Hz, 2H, SO_2CH_2), 3.21 (dd, *J* = 13.8, 6.1 Hz, 1H, Ph-CH_2), 2.89 (dd, *J* = 13.8, 7.5 Hz, 1H, Ph-CH_2), 2.71 (ddt, *J* = 29.8, 15.3, 8.3 Hz, 3H, pyrazole-CH_2-CH_2), 2.63 – 2.55 (m, 3H, pyrazole-CH_2-CH_2), 1.48 (t, *J* = 7.4 Hz, 3H, CH_3). ^{13}C NMR (150 MHz, Chloroform-*d*) δ 186.69 (d, $^3J_{CF}$ = 6.2 Hz, C=O), 165.14 (C=O), 164.96 (d, $^1J_{CF}$ = 259.4 Hz), 162.93 (dd, $^1J_{CF}$ = 250.1, $^3J_{CF}$ = 12.8 Hz), 161.16 (C=O), 155.50, 153.62, 147.16 (d, $^3J_{CF}$ = 8.6 Hz), 146.74, 145.75, 140.90, 138.96 (t, $^3J_{CF}$ = 8.8 Hz), 137.99, 131.05, 129.59 (d, $^4J_{CF}$ = 2.3 Hz), 128.86, 128.39, 127.16, 126.55, 126.54, 126.36, 123.89 (d, $^3J_{CF}$ = 8.4 Hz), 123.71 (d, $^4J_{CF}$ = 3.2 Hz), 122.46 (d, $^1J_{CF}$ = 280.9 Hz), 122.05, 121.28 (d, $^1J_{CF}$ = 268.3 Hz), 120.05, 120.02, 117.43, 116.51, 115.50 (d, $^2J_{CF}$ = 22.0 Hz), 112.17 (dd, $^2J_{CF}$ = 19.8, $^4J_{CF}$ = 4.9 Hz), 103.00 (t, $^2J_{CF}$ = 25.0 Hz), 53.27, 51.71, 51.16 (d, $^2J_{CF}$ = 35.6 Hz), 47.93, 40.45, 30.75, 23.51, 23.02, 8.10. ^{19}F NMR (376 MHz, DMSO-d_6) *δ* -60.28 (pyrazole-CF_3), -68.63 (t, *J* = 8.6 Hz, indazole-CH_2CF_3), -109.98 (t, *J* = 8.5 Hz, Ph-F × 2), -119.65 (dd, *J* = 11.8, 7.4 Hz, Ph-F). HRMS found m/z 979.1833 $[M+H]^+$, calculated for $C_{43}H_{32}ClF_9N_8O_5S$, $[M+H]^+$: 979.1834. HPLC purity: 99.81 %. Elemental analyses found: C, 52.583; H, 2.669; N, 10.682; S, 4.345 %. Calc. for $C_{43}H_{32}ClF_9N_8O_5S$: C, 52.05; H, 3.22; N, 11.56; S, 3.31 %.

S1.21.9 (*S*)-*N*-(1-(3-(4-chloro-3-(ethylsulfonamido)-1-(2,2,2-trifluoroethyl)-1*H*-indazol-7-yl)-7-(4-nitrophenyl)-4-oxo-3,4-dihydroquinazolin-2-yl)-2-(3,5-difluorophenyl)ethyl)-2-(3-(trifluoromethyl)-5,6-dihydrocyclopenta[*c*]pyrazol-1(4*H*)-yl)acetamide (IC-2j)

Yellow solid, yield 70%. m.p. 152 – 154°C. ^{1}H NMR (600 MHz, DMSO-d_6) δ 10.12 (s, 1H, SO_2NH), 9.18 (d, J = 8.4 Hz, 1H, NH), 8.43 – 8.39 (m, 2H, Ph-H), 8.33 (d, J = 8.3 Hz, 1H, quinazolinone-H), 8.19 – 8.17 (m, 2H, Ph-H), 8.17 (d, J = 1.7 Hz, 1H, quinazolinone-H), 8.09 (dd, J = 8.3, 1.9 Hz, 1H, quinazolinone-H), 7.80 (d, J = 8.0 Hz, 1H, indazole-H), 7.59 (d, J = 8.0 Hz, 1H, indazole-H), 7.03 (tt, J = 9.4, 2.4 Hz, 1H, Ph-H), 6.66 – 6.61 (m, 2H, Ph-H), 4.80 (dq, J = 17.0, 8.6 Hz, 1H, CF_3CH_2), 4.63 – 4.58 (m, 1H, CH), 4.57 (s, 2H, $COCH_2$), 4.26 (dq, J = 16.8, 8.6 Hz, 1H, CF_3CH_2), 3.41 (dd, J = 14.2, 3.4 Hz, 1H, Ph-CH_2), 3.35 – 3.32 (m, 2H, SO_2CH_2), 3.04 (dd, J = 14.2, 10.3 Hz, 1H, Ph-CH_2), 2.55 (t, J = 7.0 Hz, 2H, pyrazole-CH_2), 2.46 – 2.39 (m, 4H, pyrazole-CH_2, CH_2), 1.33 (t, J = 7.3 Hz, 3H, CH_3). ^{13}C NMR (150 MHz, DMSO-d_6) δ 166.34 (C=O), 162.58 (dd, $^{1}J_{CF}$ = 246.3, $^{3}J_{CF}$ = 13.4 Hz), 161.36 (C=O), 157.15, 153.80, 148.10, 147.35, 14S12, 141.87 (t, $^{3}J_{CF}$ = 9.7 Hz), 140.23, 138.44, 134.33, 134.08, 131.83, 129.18, 128.38, 128.31, 127.43, 125.99, 12S12, 124.80, 123.56, 123.22 (d, $^{1}J_{CF}$ = 280.8 Hz), 122.10 (d, $^{1}J_{CF}$ = 268.0 Hz), 120.77, 119.50, 118.77, 112.53 (dd, $^{2}J_{CF}$ = 20.3, $^{4}J_{CF}$ = 4.5 Hz), 102.79 (t, $^{2}J_{CF}$ = 25.4 Hz), 53.05, 52.63, 51.01, 48.11, 38.46, 30.74, 23.66, 23.00, 8.38. HRMS found m/z 978.1829 $[M+H]^+$, calculated for $C_{42}H_{32}ClF_8N_9O_6S$, $[M+H]^+$: 978.1830. HPLC purity: 98.31 %.

S1.21.10 (*S*)-*N*-(1-(3-(4-chloro-3-(ethylsulfonamido)-1-(2,2,2-trifluoroethyl)-1*H*-indazol-7-yl)-7-(3-fluoro-4-nitrophenyl)-4-oxo-3,4-dihydroquinazolin-2-yl)-2-(3,5-difluorophenyl)ethyl)-2-(3-(trifluoromethyl)-5,6-dihydrocyclopenta[*c*]pyrazol-1(4*H*)-yl)acetamide (IC-2k)

Yellow solid, yield 41%. m.p. 131 – 134°C. ^{1}H NMR (600 MHz, DMSO-d_6) δ 10.12 (s, 1H, SO_2NH), 9.18 (d, J = 8.4 Hz, 1H, NH), 8.43 – 8.39 (m, 2H, Ph-H), 8.33 (d, J = 8.3 Hz, 1H, quinazolinone-H), 8.19 – 8.17 (m, 2H, Ph-H), 8.17 (d, J = 1.7 Hz, 1H,

quinazolinone-H), 8.09 (dd, J = 8.3, 1.9 Hz, 1H, quinazolinone-H), 7.80 (d, J = 8.0 Hz, 1H, indazole-H), 7.59 (d, J = 8.0 Hz, 1H, indazole-H), 7.03 (tt, J = 9.4, 2.4 Hz, 1H, Ph-H), 6.66 – 6.61 (m, 2H, Ph-H), 4.80 (dq, J = 17.0, 8.6 Hz, 1H, CF_3CH_2), 4.63 – 4.58 (m, 1H, CH), 4.57 (s, 2H, $COCH_2$), 4.26 (dq, J = 16.8, 8.6 Hz, 1H, CF_3CH_2), 3.41 (dd, J = 14.2, 3.4 Hz, 1H, Ph-CH_2), 3.35 – 3.32 (m, 2H, SO_2CH_2), 3.04 (dd, J = 14.2, 10.3 Hz, 1H, Ph-CH_2), 2.55 (t, J = 7.0 Hz, 2H, pyrazole-CH_2), 2.46 – 2.39 (m, 4H, pyrazole-CH_2, CH_2), 1.33 (t, J = 7.3 Hz, 3H, CH_3). ^{13}C NMR (150 MHz, DMSO-d_6) δ 166.33 (C=O), 162.61 (dd, $^{1}J_{CF}$ = 246.2, $^{3}J_{CF}$ = 13.4 Hz), 161.32 (C=O), 157.15, 155.54 (d, $^{1}J_{CF}$ = 261.4 Hz), 153.82, 147.31, 146.58 (d, $^{3}J_{CF}$ = 8.6 Hz), 143.92, 141.87 (t, $^{3}J_{CF}$ = 8.8 Hz), 140.24, 138.48, 137.15 (d, $^{3}J_{CF}$ = 7.7 Hz), 134.14, 131.82, 128.32 (d, $^{2}J_{CF}$ = 21.5 Hz), 127.54, 127.53, 127.46, 126.25, 12S14, 124.48 (d, $^{4}J_{CF}$ = 3.5 Hz), 123.57, 123.22 (d, $^{1}J_{CF}$ = 280.5 Hz), 122.11 (d, $^{1}J_{CF}$ = 268.1 Hz), 121.11, 119.52, 118.77, 117.77 (d, $^{2}J_{CF}$ = 22.0 Hz), 112.53 (dd, $^{2}J_{CF}$ = 19.7, $^{4}J_{CF}$ = 4.3 Hz), 102.78 (t, $^{2}J_{CF}$ = 26.3 Hz), 53.06, 52.64, 51.04, 48.15, 38.47, 30.75, 23.69, 23.01, 8.36. ^{19}F NMR (376 MHz, DMSO-d_6) δ -60.28 (pyrazole-CF_3), -68.62 (t, J = 8.6 Hz, indazole-CH_2CF_3), -109.97 (t, J = 8.4 Hz, Ph-F × 2), -117.68 (dd, J = 12.5, 7.9 Hz, Ph-F). HRMS found m/z 996.1739 $[M+H]^+$, calculated for $C_{42}H_{32}ClF_8N_9O_6S$, $[M+H]^+$: 996.1736. HPLC purity: 99.62 %.

SII. ^{1}H NMR, ^{13}C NMR, ^{19}F NMR and HRMS of representative compounds

^{1}H NMR, ^{13}C NMR, ^{19}F NMR, HRMS and HPLC of IA-1

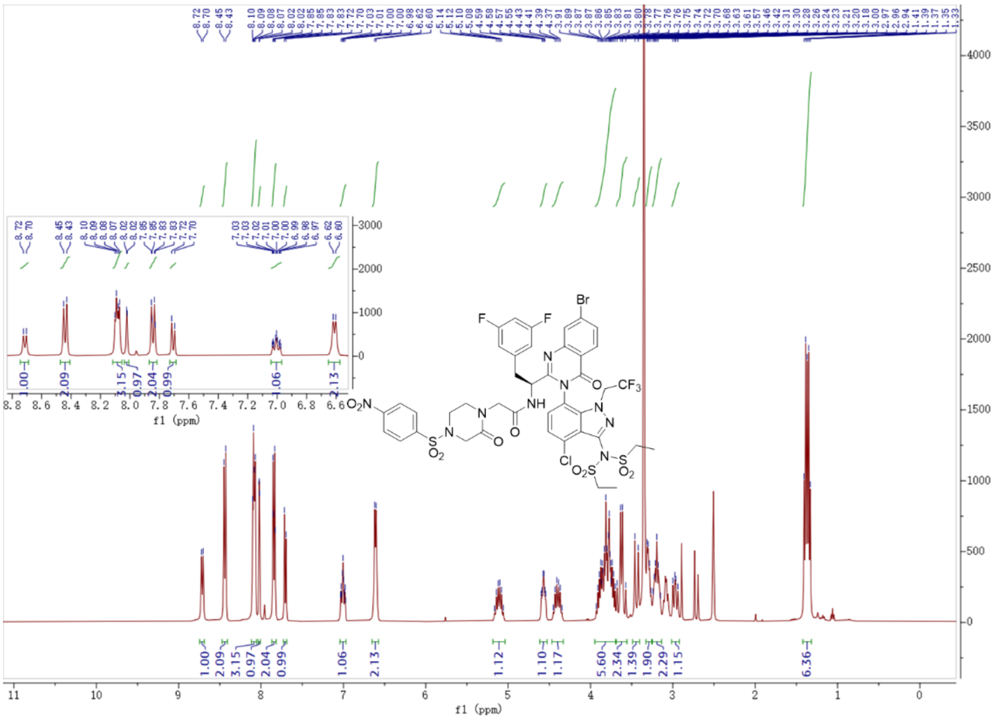

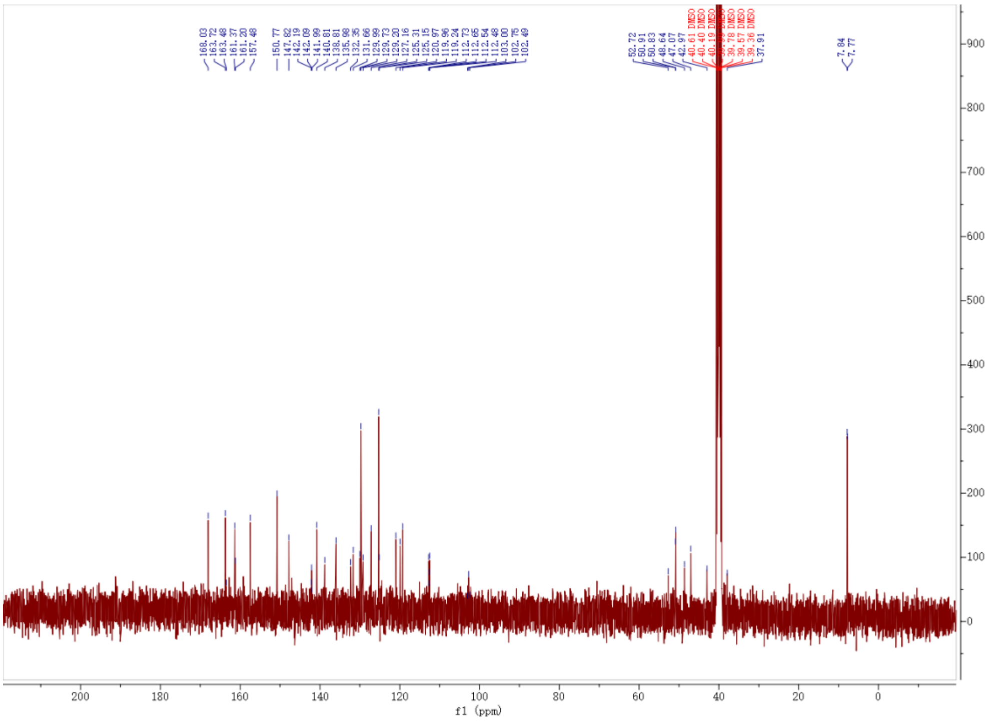

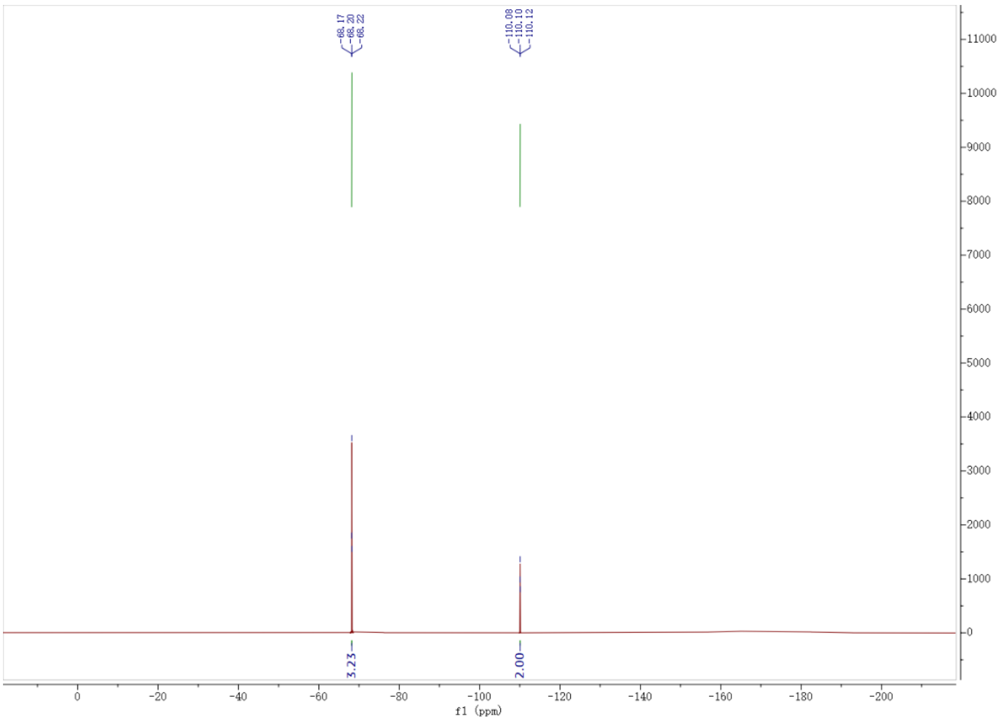
-68.17
-68.20
-68.22
-110.08
-110.10
-110.12
3.23
2.00
f1 (ppm)

1

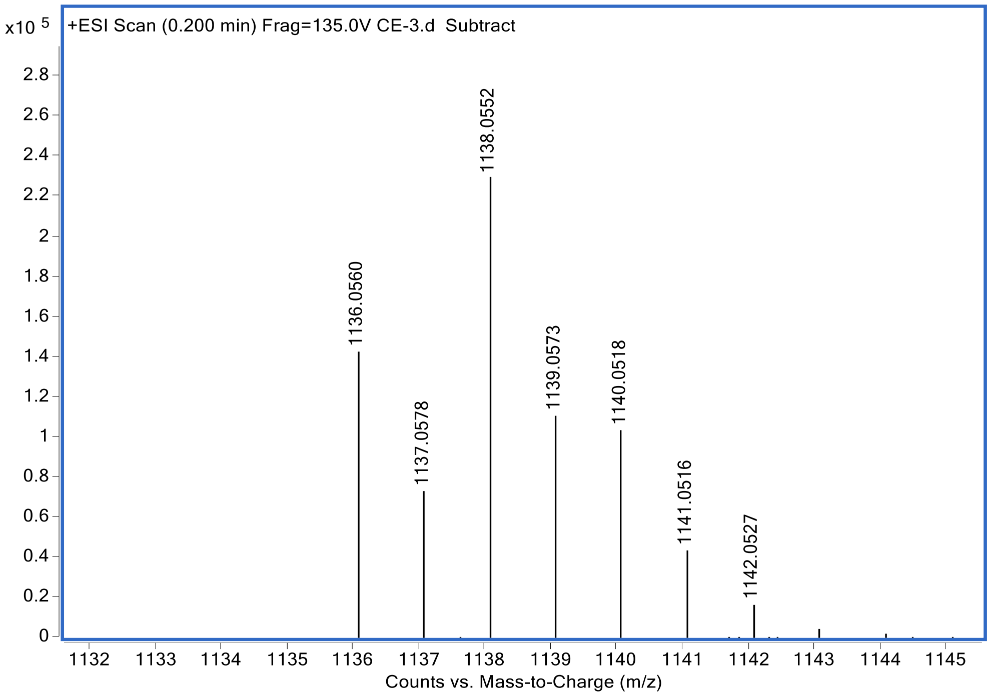
+ESI Scan (0.200 min) Frag=135.0V CE-3.d Subtract
x10 5
1136.0560
1137.0578
1138.0552
1139.0573
1140.0518
1141.0516
1142.0527
Counts vs. Mass-to-Charge (m/z)

2

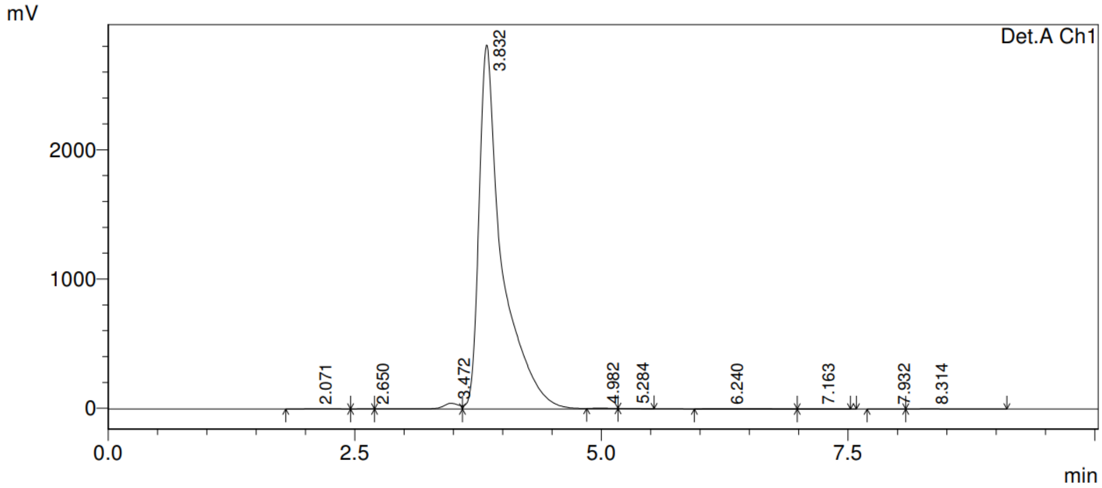

1 Det.A Ch1/254nm

PeakTable

¼ì²âÆ÷ A Ch1 254nm

Peak#	Ret. Time	Area	Height	Area %	Height %
1	2.071	25873	1485	0.056	0.052
2	2.650	9900	772	0.021	0.027
3	3.472	516806	43583	1.114	1.520
4	3.832	45719407	2814903	98.587	98.154
5	4.982	28791	2802	0.062	0.098
6	5.284	21470	1772	0.046	0.062
7	6.240	37305	1761	0.080	0.061
8	7.163	1763	95	0.004	0.003
9	7.932	1294	95	0.003	0.003
10	8.314	11890	585	0.026	0.020
Total		46374498	2867854	100.000	100.000

^{1}H NMR, ^{13}C NMR, HRMS and HPLC of IB-1a

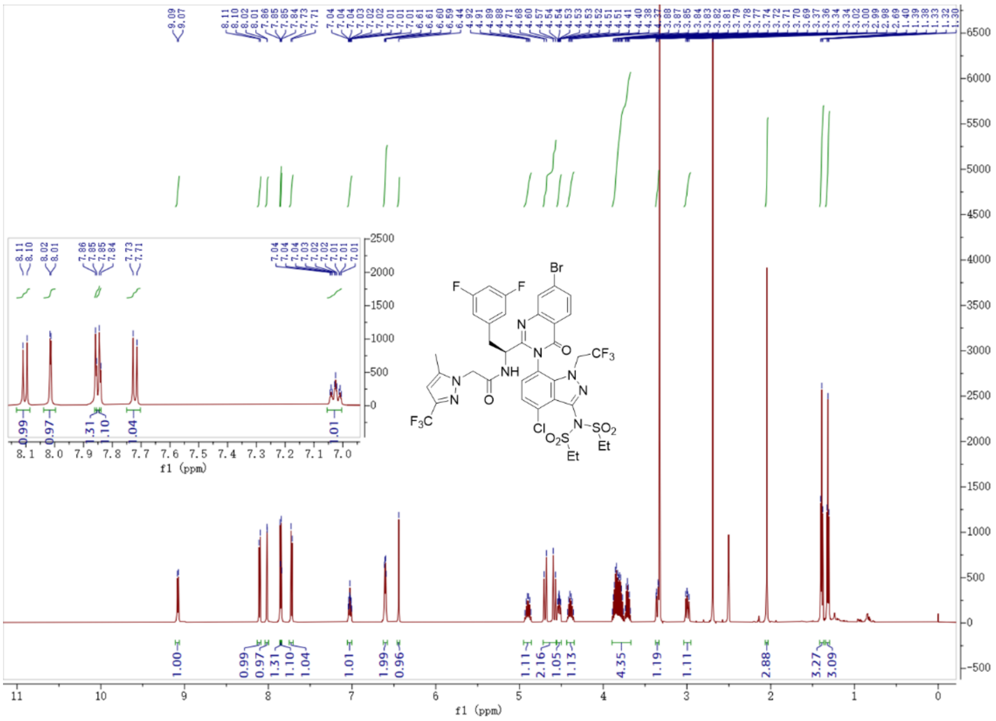

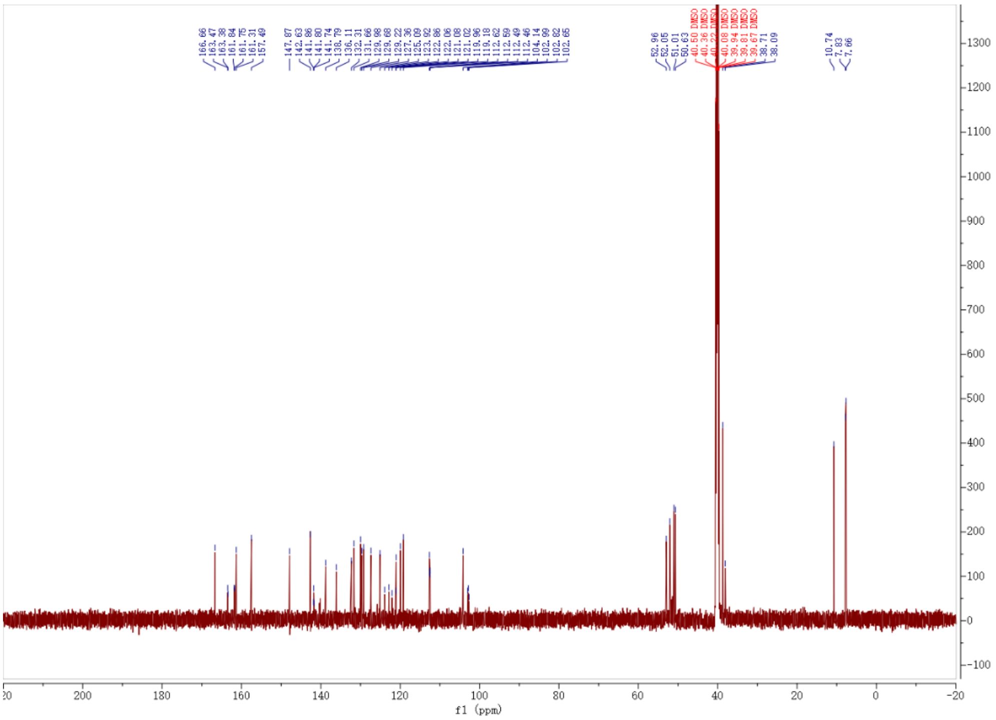

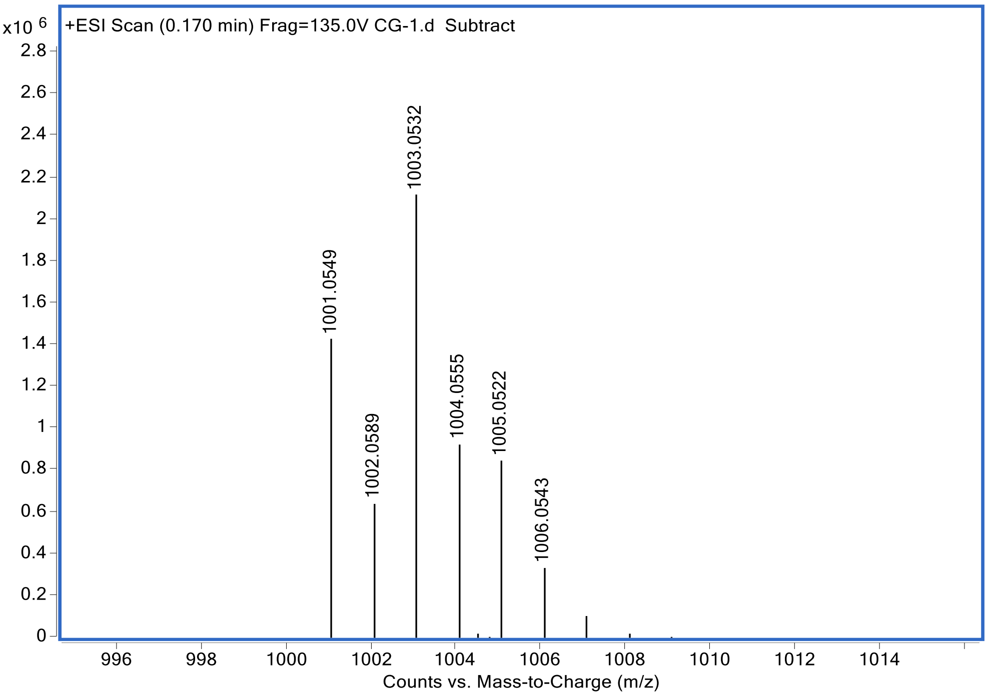

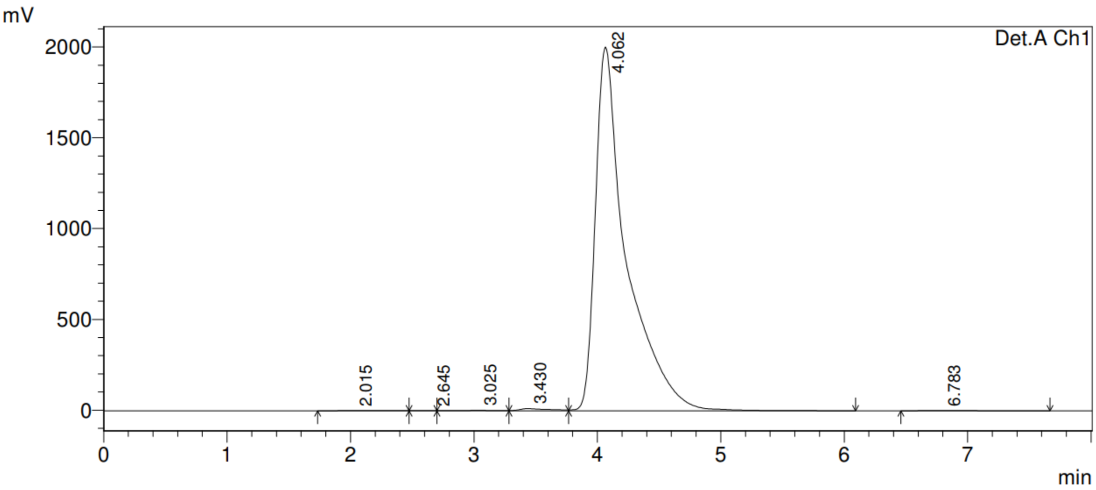

1 Det.A Ch1/254nm

PeakTable

¼ì²âÆ÷ A Ch1 254nm

Peak#	Ret. Time	Area	Height	Area %	Height %
1	2.015	32013	1598	0.090	0.079
2	2.645	8297	702	0.023	0.035
3	3.025	52569	2173	0.148	0.108
4	3.430	197275	11895	0.557	0.589
5	4.062	35102942	2003090	99.130	99.151
6	6.783	18099	783	0.051	0.039
Total		35411195	2020242	100.000	100.000

^1H NMR, ^{13}C NMR, HRMS and HPLC of IB-1d

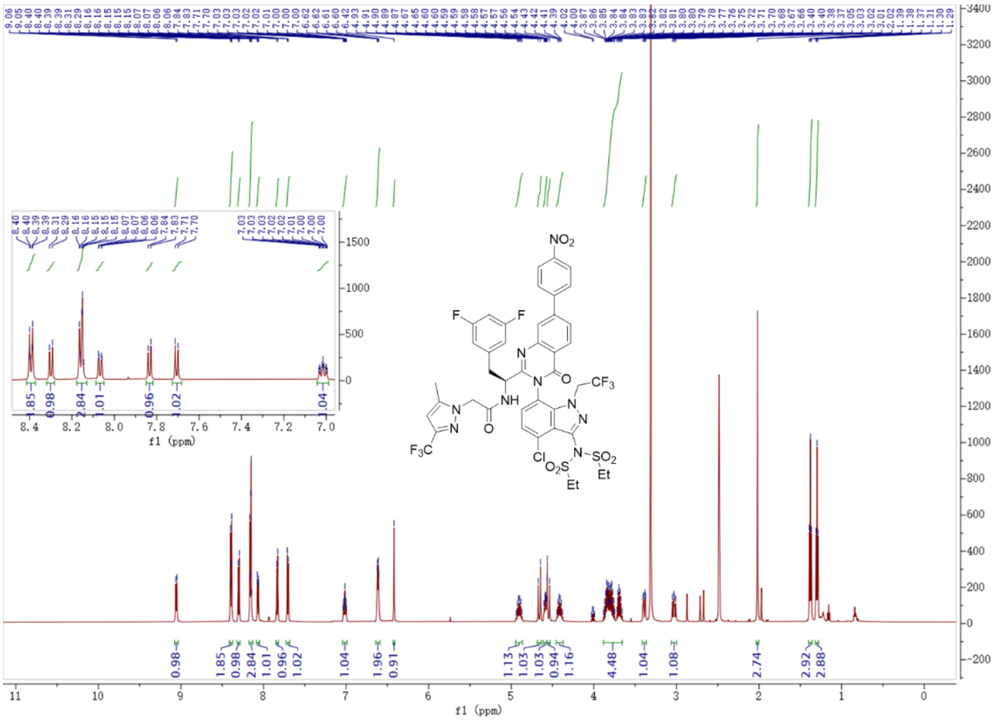

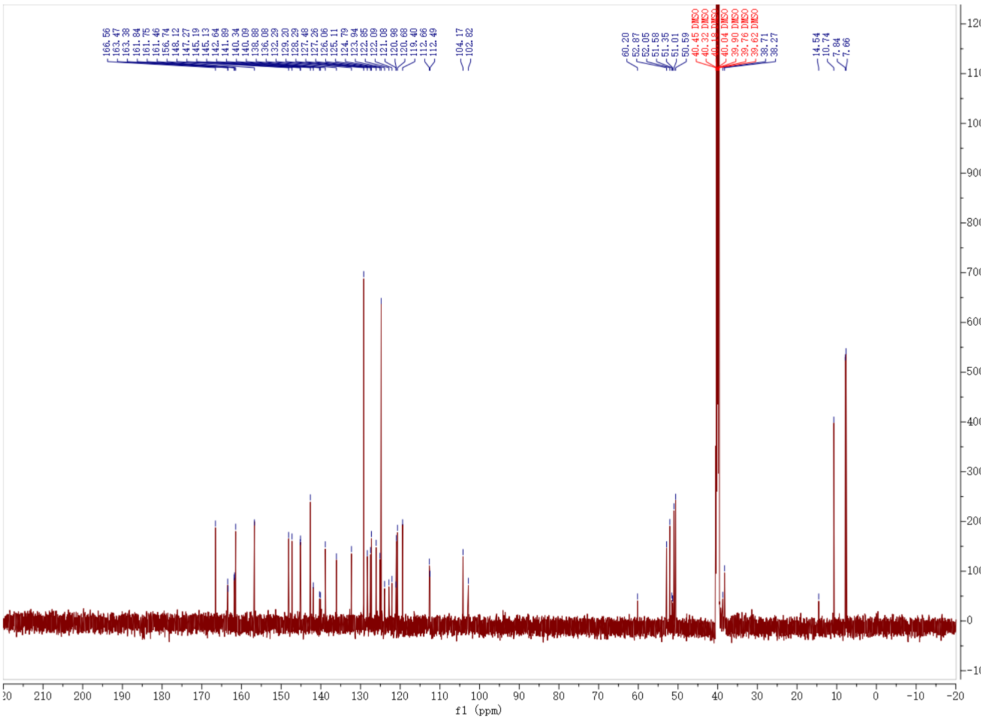

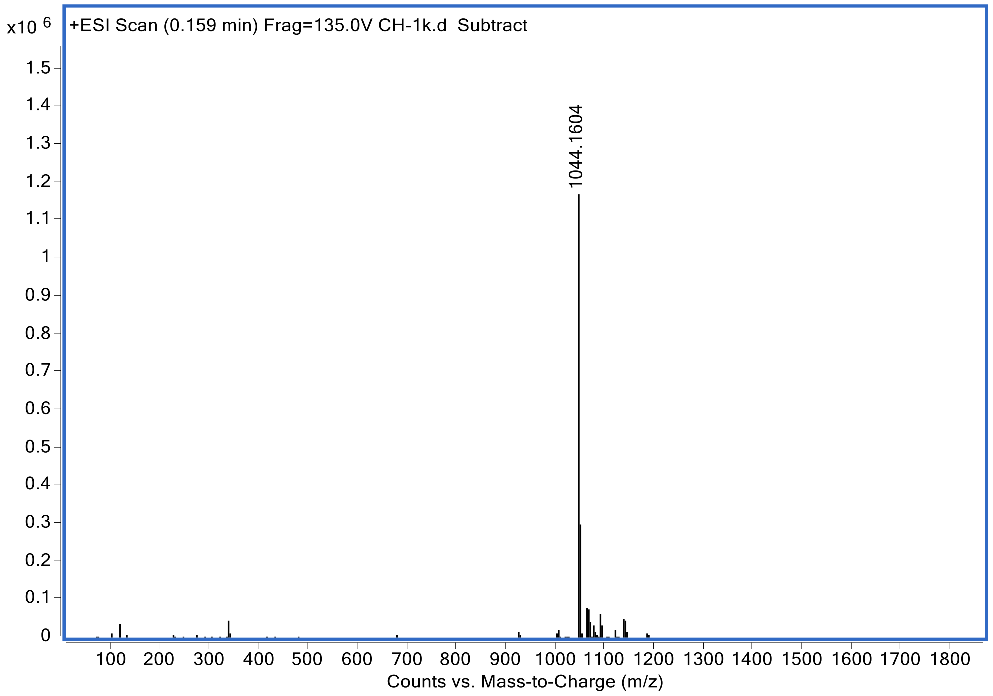

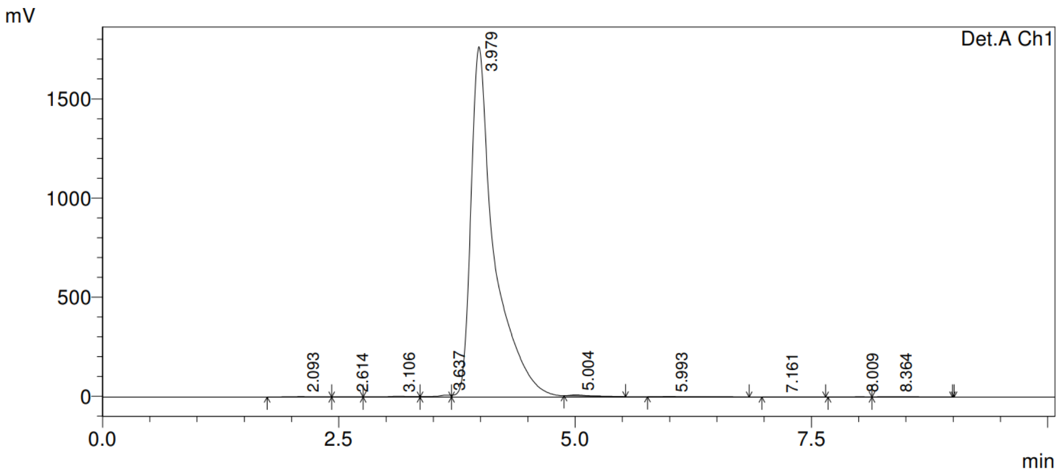

PeakTable

¼ì²âÆ÷ A Ch1 254nm

Peak#	Ret. Time	Area	Height	Area %	Height %
1	2.093	38953	2146	0.126	0.120
2	2.614	16500	895	0.053	0.050
3	3.106	78058	3546	0.253	0.198
4	3.637	106474	9820	0.345	0.548
5	3.979	30546307	1766524	98.838	98.636
6	5.004	69647	5568	0.225	0.311
7	5.993	27103	1277	0.088	0.071
8	7.161	1357	60	0.004	0.003
9	8.009	4311	274	0.014	0.015
10	8.364	16839	849	0.054	0.047
Total		30905549	1790960	100.000	100.000

^{1}H NMR, ^{13}C NMR, COSY, HSQC HRMS and HPLC of IB-2a

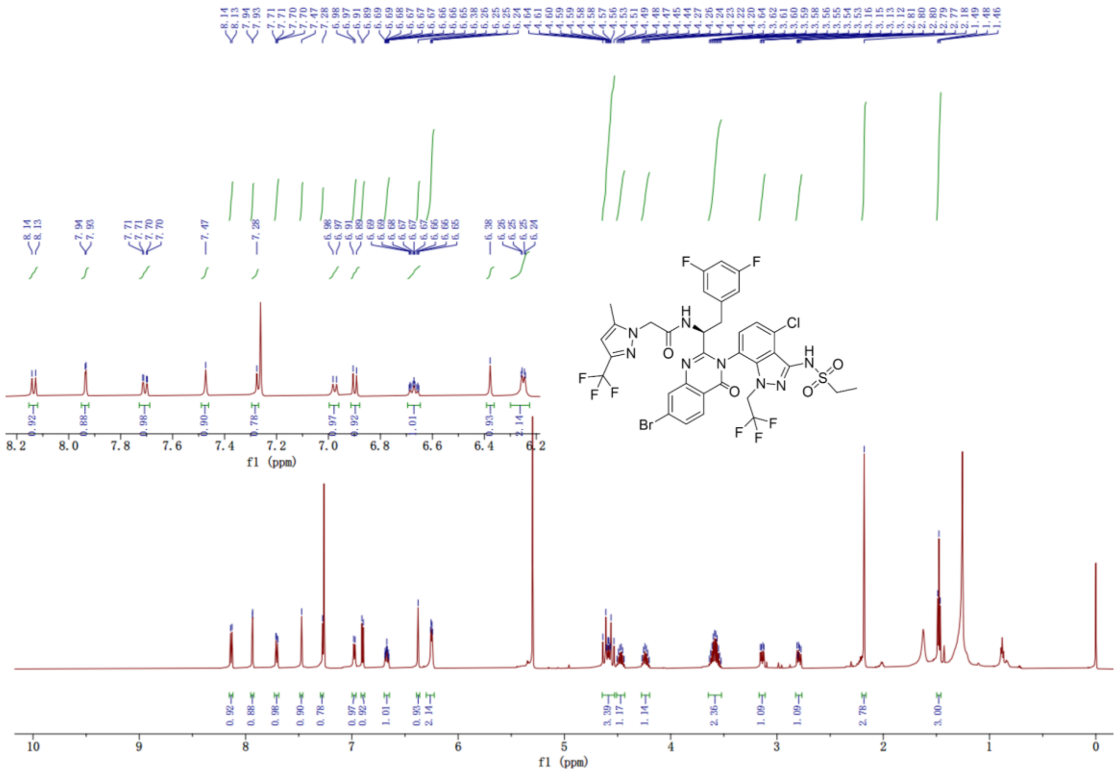

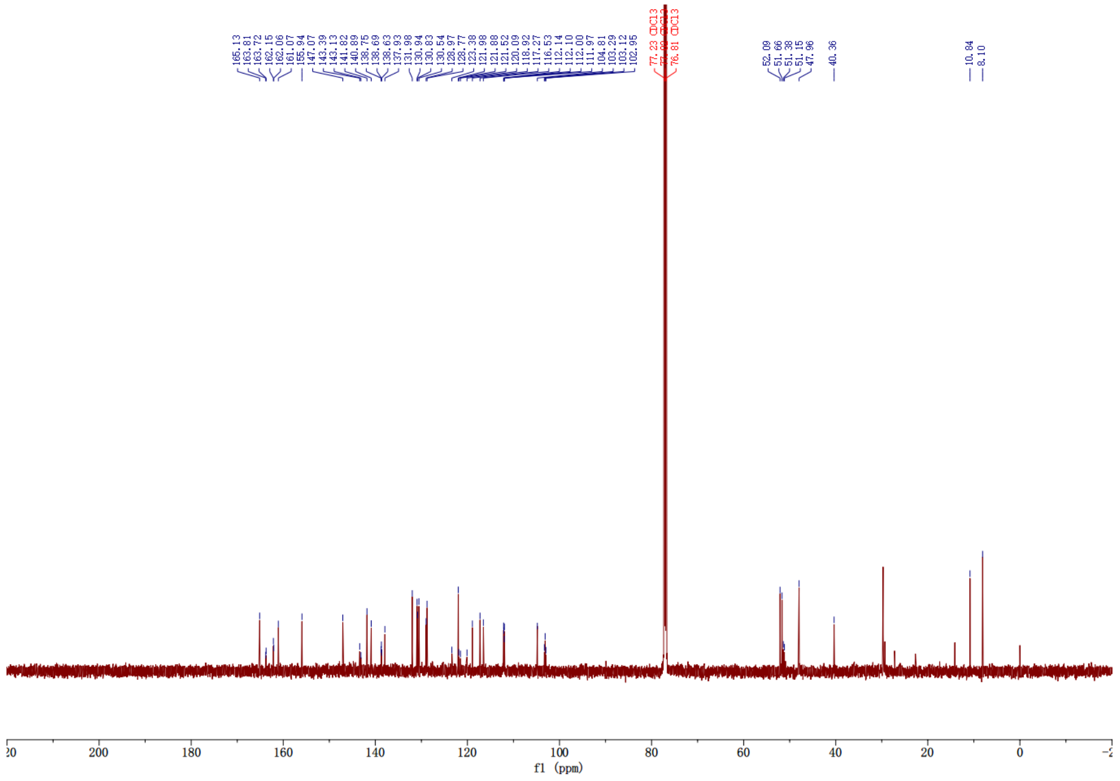

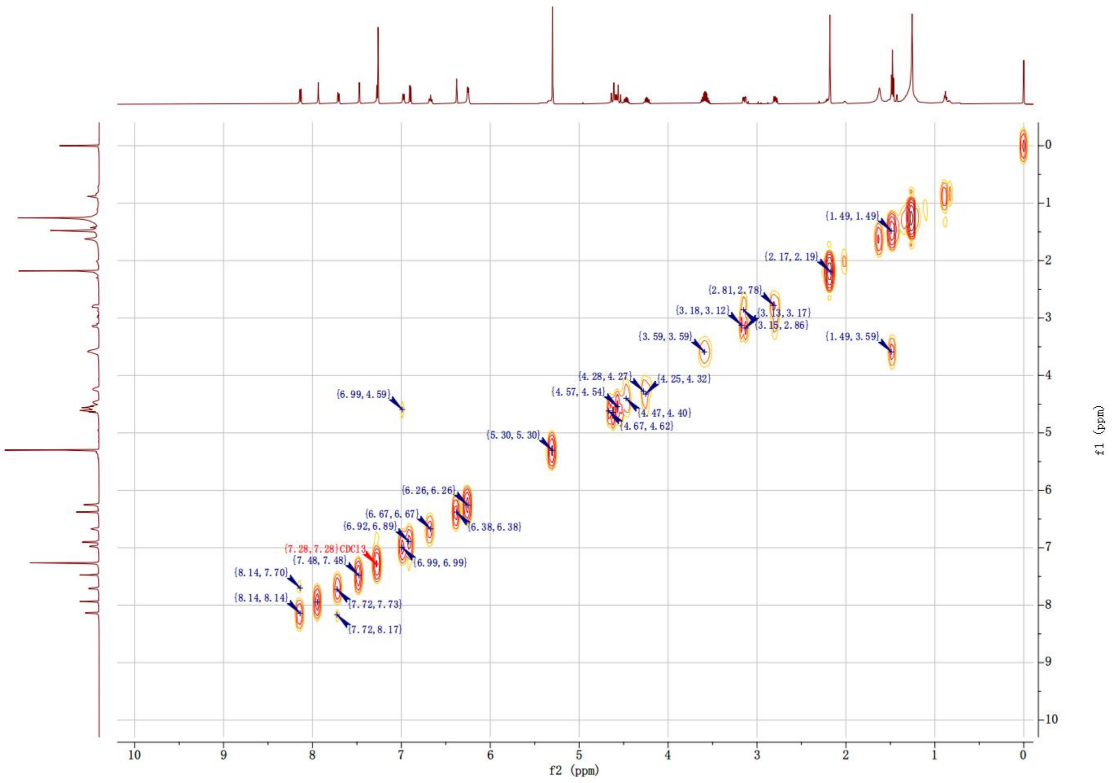
{1.49, 1.49}
{2.17, 2.19}
{2.81, 2.78}
{3.18, 3.12}
{3.13, 3.17}
{3.15, 2.86}
{3.59, 3.59}
{1.49, 3.59}
{4.28, 4.27}
{4.25, 4.32}
{4.57, 4.54}
{4.47, 4.40}
{4.67, 4.62}
{6.99, 4.59}
{5.30, 5.30}
{6.26, 6.26}
{6.67, 6.67}
{6.38, 6.38}
{6.92, 6.89}
{6.99, 6.99}
{7.28, 7.28}CDCl3
{7.48, 7.48}
{8.14, 7.70}
{8.14, 8.14}
{7.72, 7.73}
{7.72, 8.17}
f2 (ppm)
f1 (ppm)

1

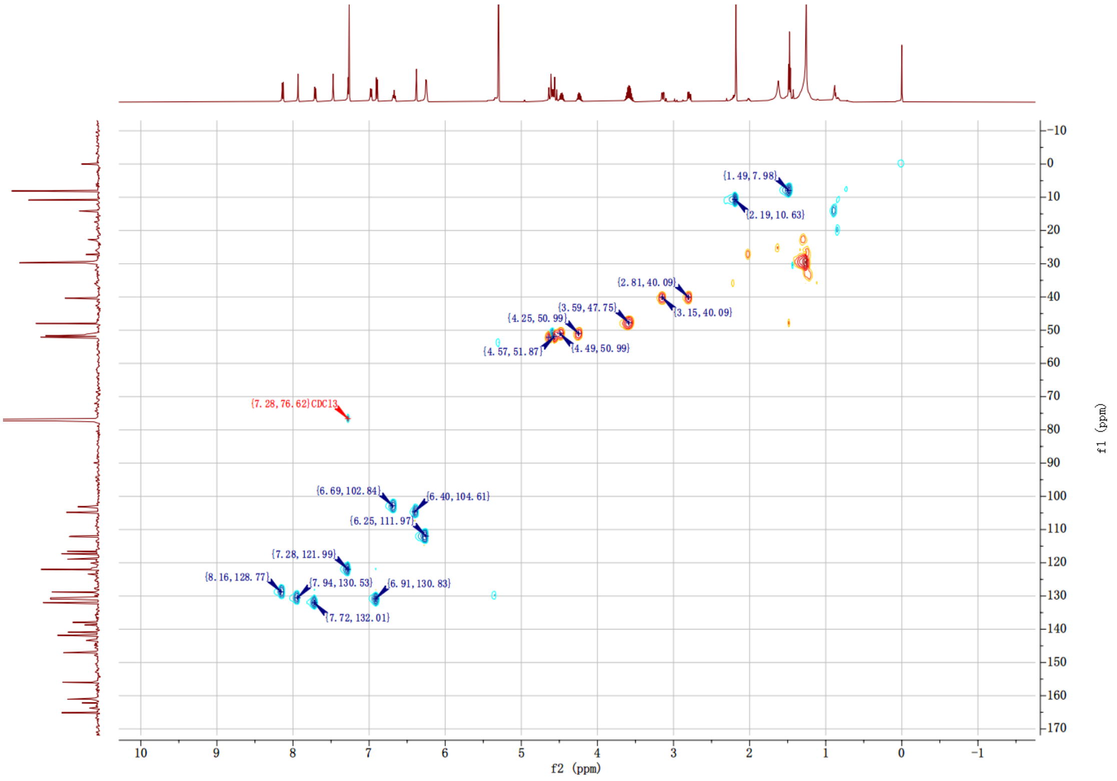
{1.49, 7.98}
{2.19, 10.63}
{2.81, 40.09}
{3.15, 40.09}
{3.59, 47.75}
{4.25, 50.99}
{4.49, 50.99}
{4.57, 51.87}
{7.28, 76.62}CDCl3
{6.69, 102.84}
{6.40, 104.61}
{6.25, 111.97}
{7.28, 121.99}
{8.16, 128.77}
{7.94, 130.53}
{6.91, 130.83}
{7.72, 132.01}
f2 (ppm)
f1 (ppm)

2

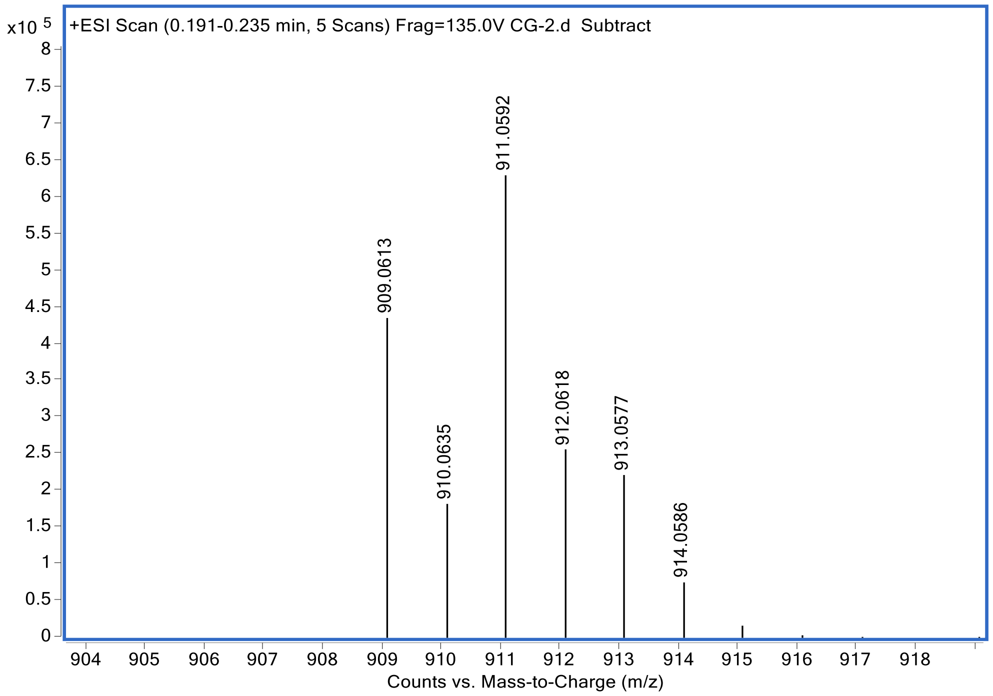

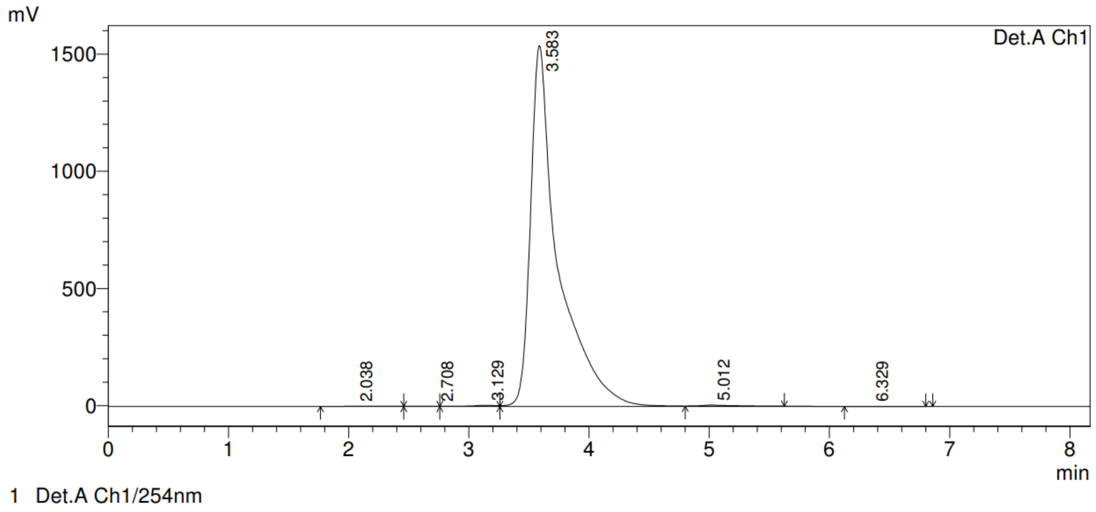

PeakTable

¼ì²âÆ÷ A Ch1 254nm

Peak#	Ret. Time	Area	Height	Area %	Height %
1	2.038	20502	1153	0.083	0.074
2	2.708	12652	865	0.051	0.056
3	3.129	75328	4894	0.303	0.316
4	3.583	24642458	1538557	99.252	99.274
5	5.012	76171	4280	0.307	0.276
6	6.329	1034	60	0.004	0.004
Total		24828145	1549809	100.000	100.000

^{1}H NMR, ^{13}C NMR, ^{19}F NMR, HRMS and HPLC of IB-2d

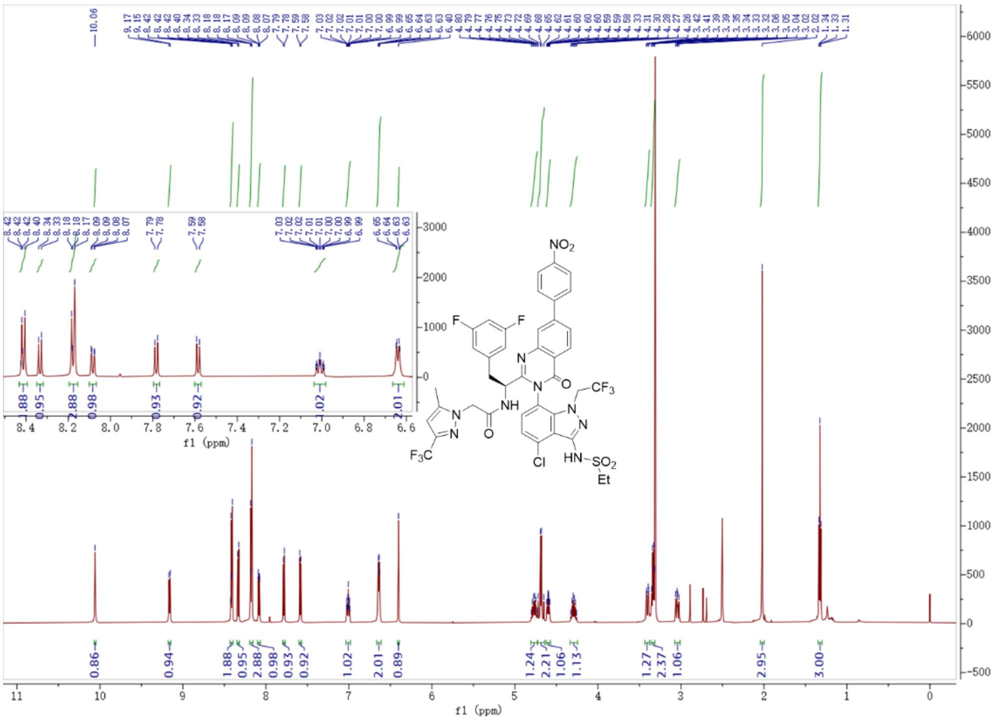

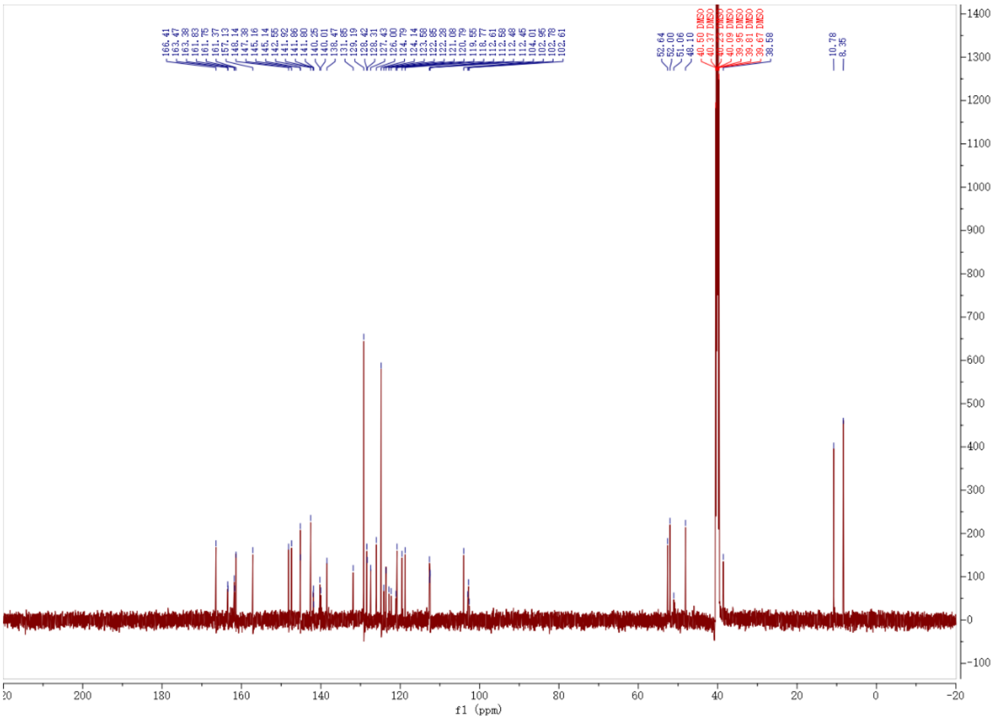

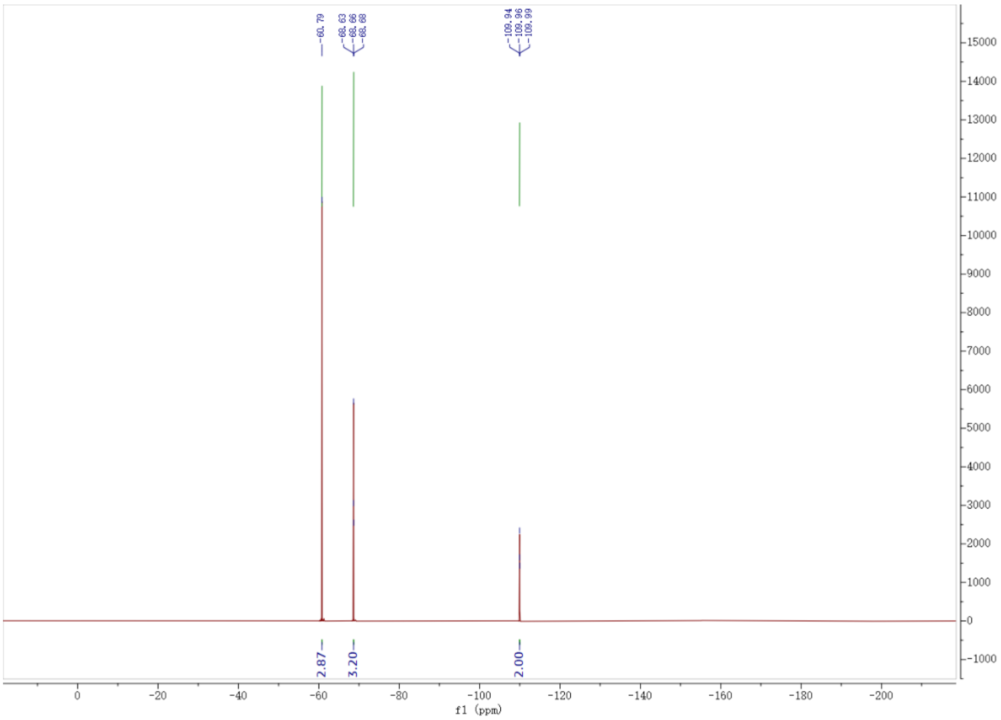
-60.79
-68.63
-68.66
-68.68
-109.94
-109.96
-109.99
2.87
3.20
2.00
f1 (ppm)

1

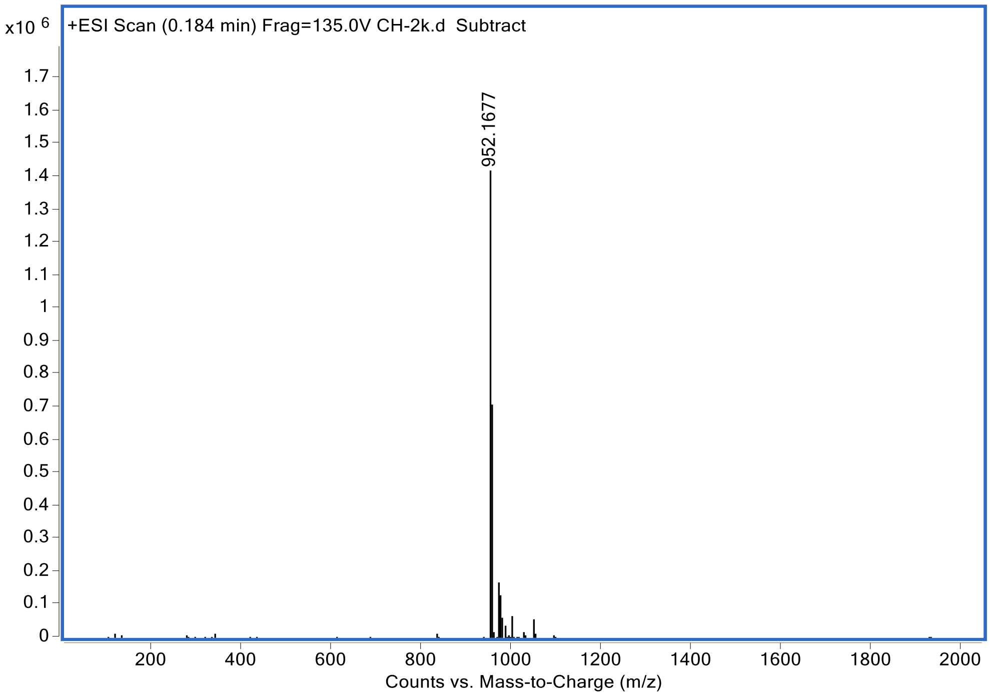
x10 6
+ESI Scan (0.184 min) Frag=135.0V CH-2k.d Subtract
952.1677
Counts vs. Mass-to-Charge (m/z)

2

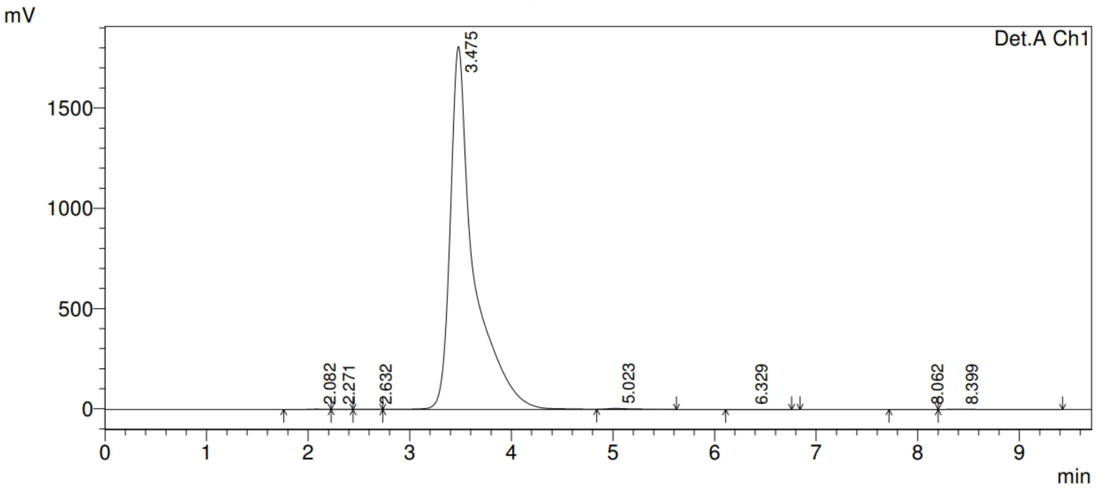

1 Det.A Ch1/254nm

PeakTable

¼ì²âÆ÷ A Ch1 254nm

Peak#	Ret. Time	Area	Height	Area %	Height %
1	2.082	18940	1892	0.065	0.104
2	2.271	11236	969	0.038	0.053
3	2.632	13860	845	0.047	0.046
4	3.475	29186928	1808815	99.569	99.516
5	5.023	61828	4087	0.211	0.225
6	6.329	1679	91	0.006	0.005
7	8.062	4397	276	0.015	0.015
8	8.399	14336	637	0.049	0.035
Total		29313205	1817611	100.000	100.000

^{1}H NMR, ^{13}C NMR, HRMS and HPLC of IC-1a

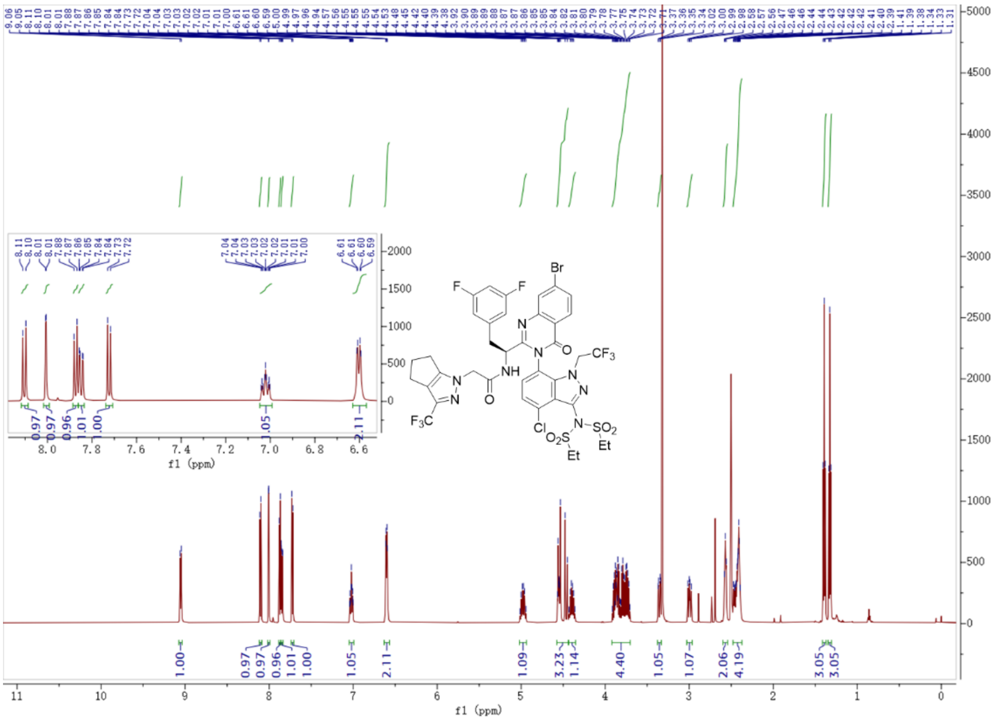

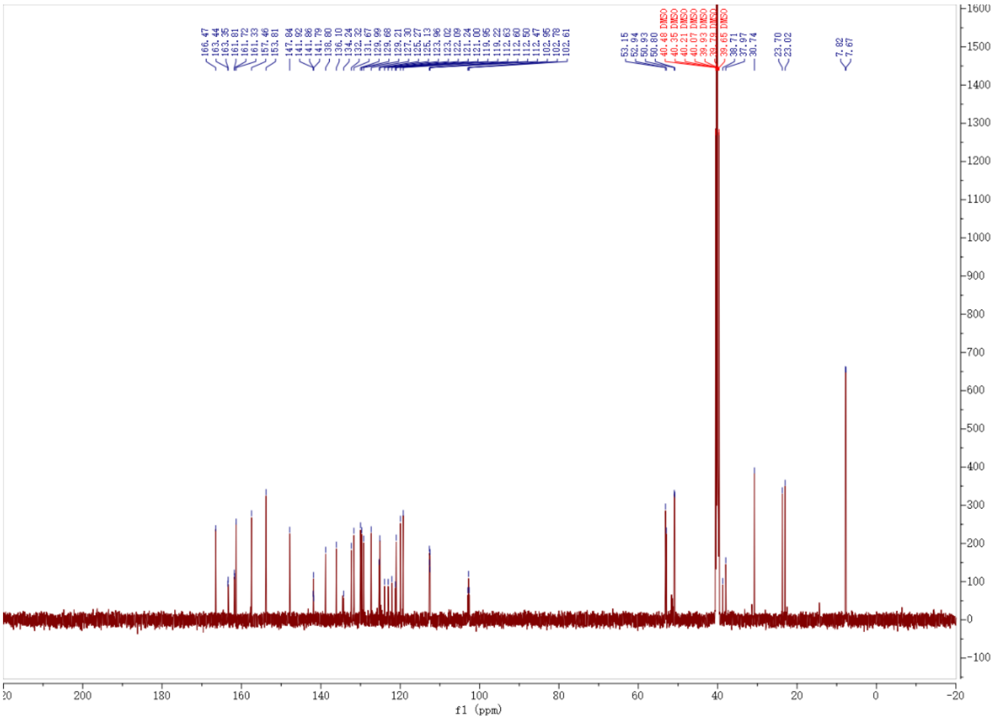

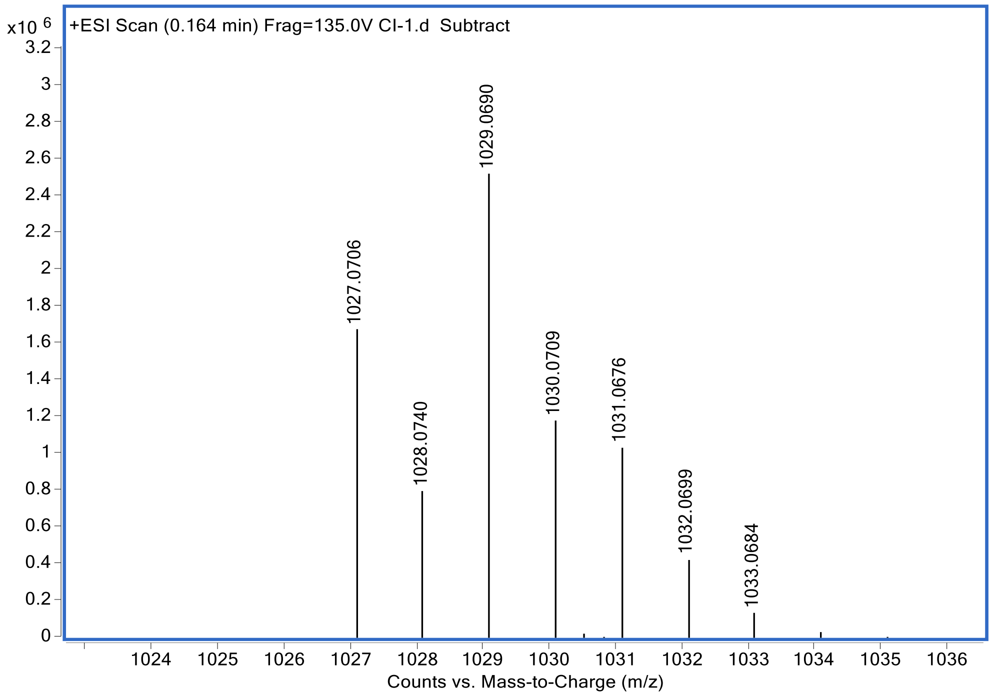

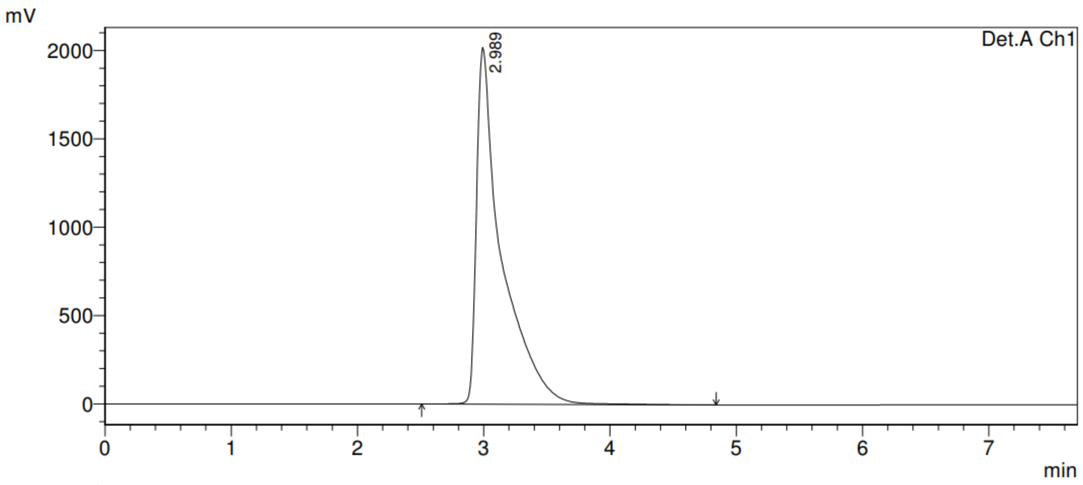

1 Det.A Ch1/254nm

PeakTable

¼ì²âÆ÷ A Ch1 254nm

Peak#	Ret. Time	Area	Height	Area %	Height %
1	2.989	27929236	2018044	100.000	100.000
Total		27929236	2018044	100.000	100.000

^{1}H NMR, ^{13}C NMR, HRMS and HPLC of IC-1b

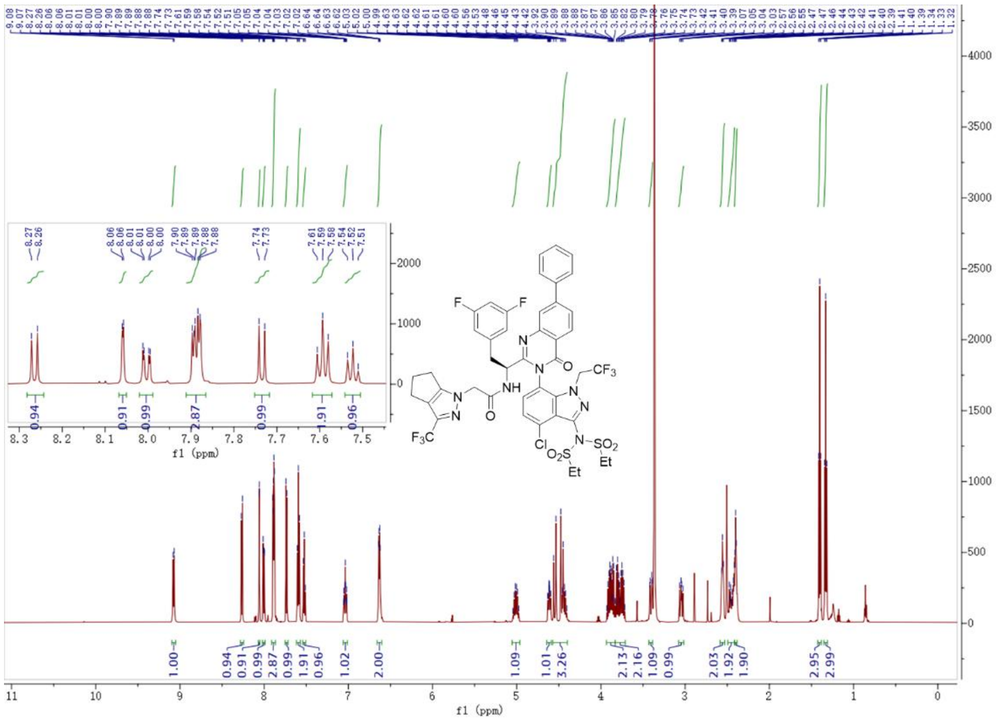

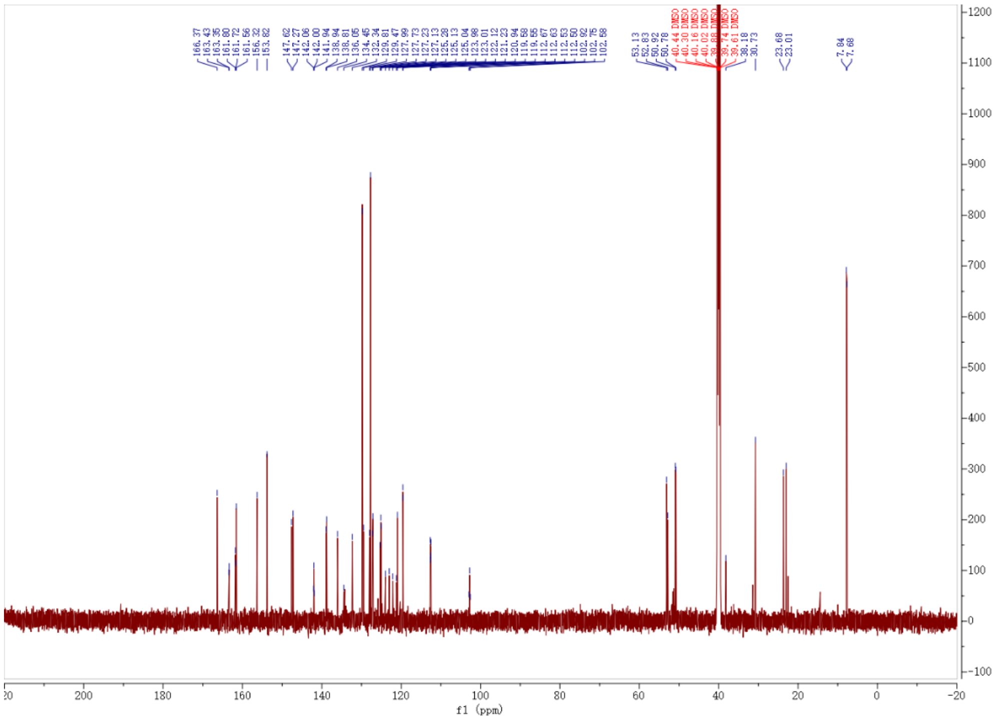

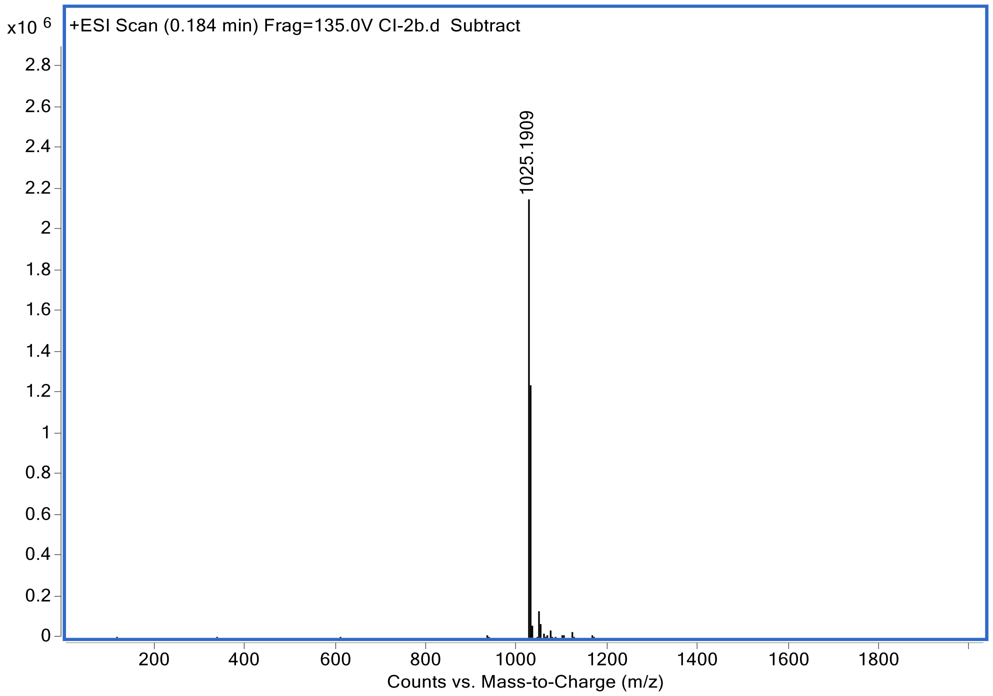

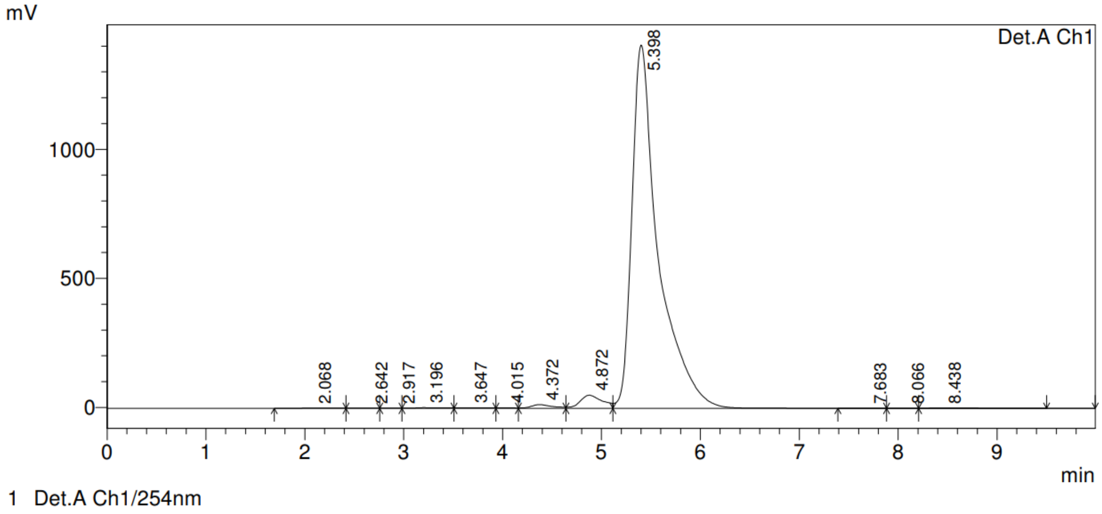

PeakTable

¼ì²âÆ÷ A Ch1 254nm

Peak#	Ret. Time	Area	Height	Area %	Height %
1	2.068	19866	1207	0.073	0.081
2	2.642	13250	733	0.048	0.049
3	2.917	10382	840	0.038	0.057
4	3.196	53495	2556	0.196	0.172
5	3.647	50208	2445	0.184	0.165
6	4.015	21219	1674	0.078	0.113
7	4.372	215039	14155	0.786	0.955
8	4.872	797793	50688	2.916	3.420
9	5.398	26155690	1407184	95.608	94.937
10	7.683	1717	86	0.006	0.006
11	8.066	3045	195	0.011	0.013
12	8.438	15554	464	0.057	0.031
Total		27357258	1482227	100.000	100.000

^{1}H NMR, ^{13}C NMR, HRMS and HPLC of IC-1i

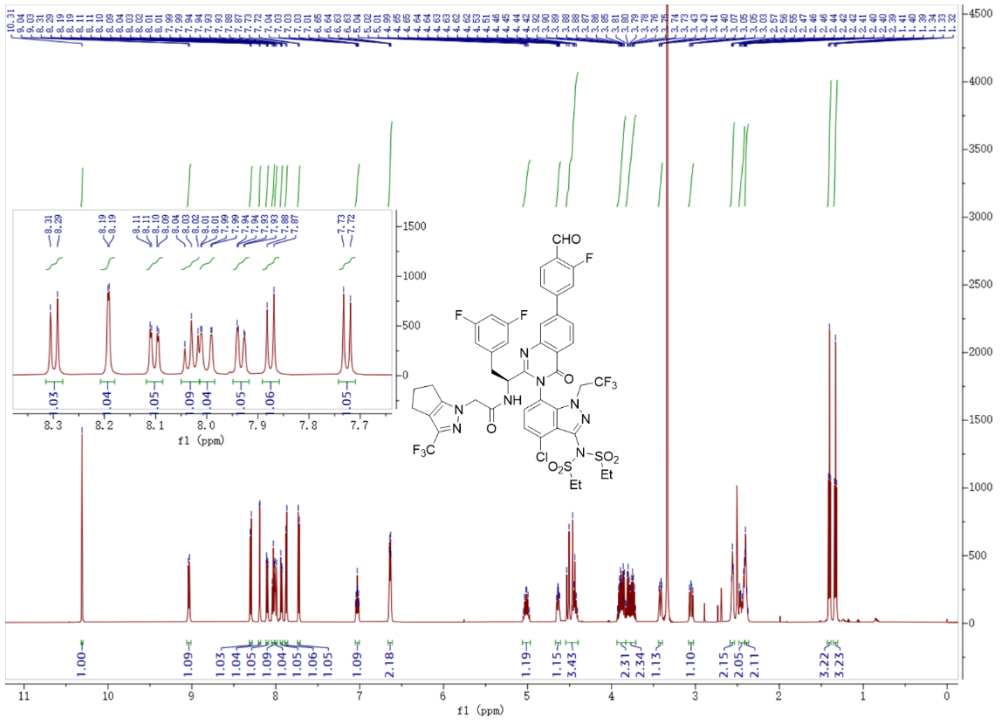

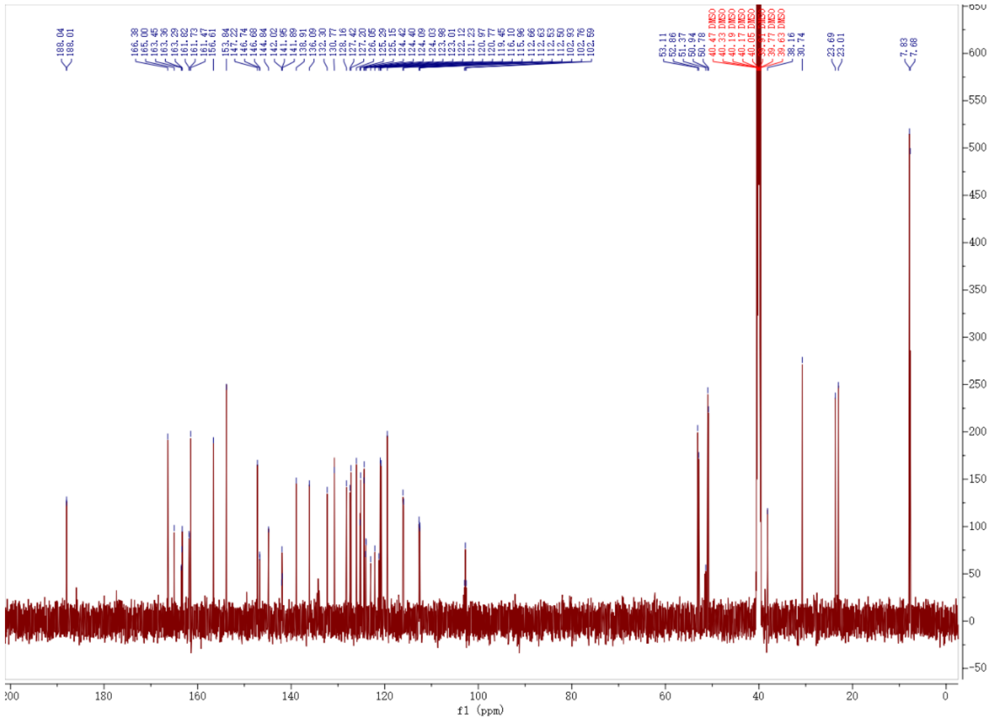

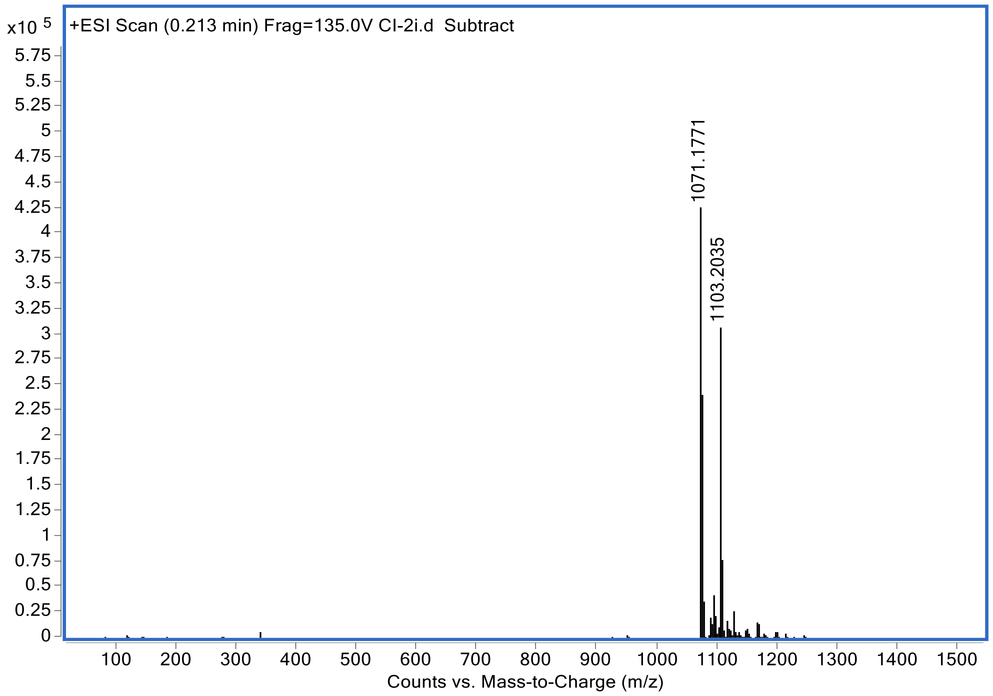

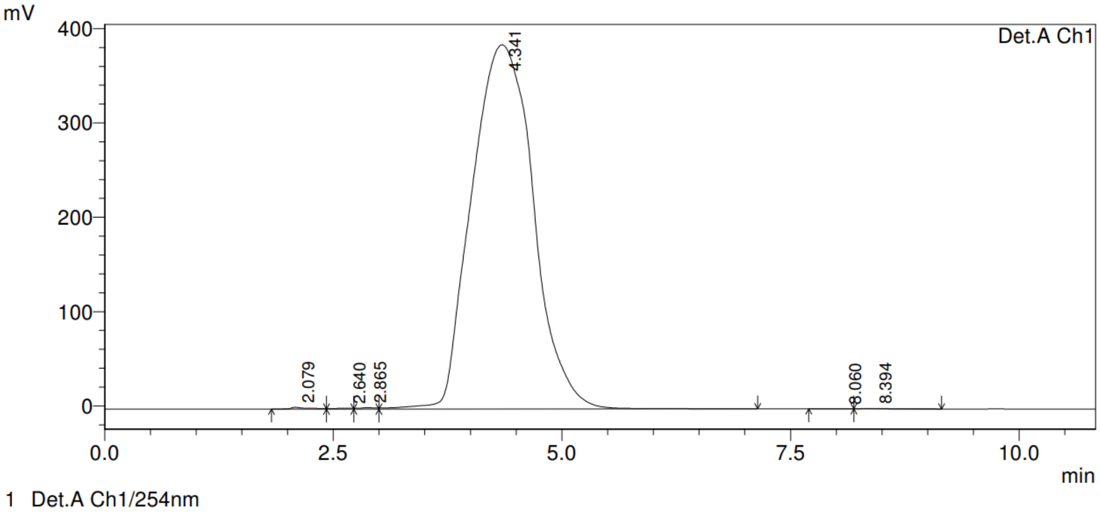

PeakTable

¼ì²âÆ÷ A Ch1 254nm

Peak#	Ret. Time	Area	Height	Area %	Height %
1	2.079	23975	1855	0.132	0.475
2	2.640	11773	714	0.065	0.183
3	2.865	17736	1353	0.097	0.346
4	4.341	18125651	386056	99.623	98.805
5	8.060	3637	231	0.020	0.059
6	8.394	11541	516	0.063	0.132
Total		18194314	390725	100.000	100.000

^{1}H NMR, ^{13}C NMR and HRMS of IC-1k

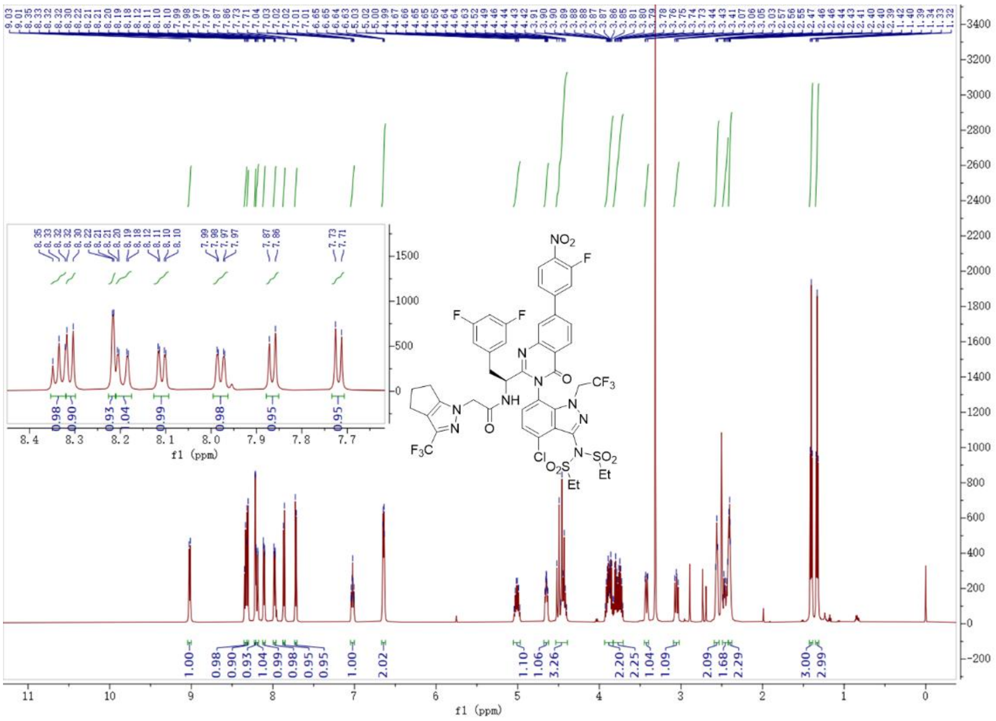

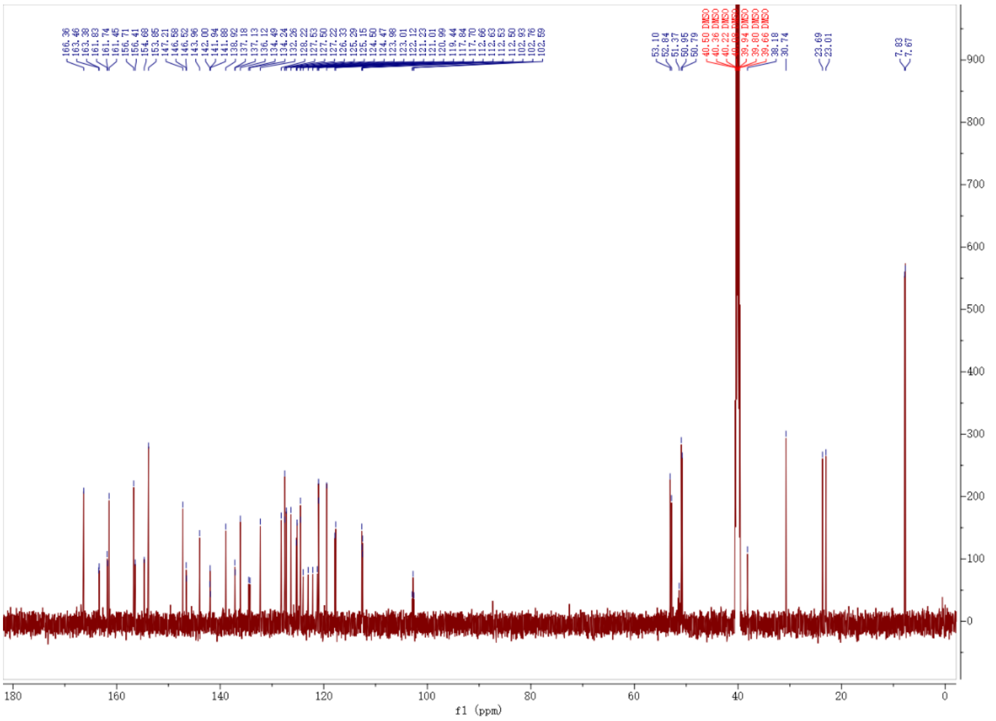

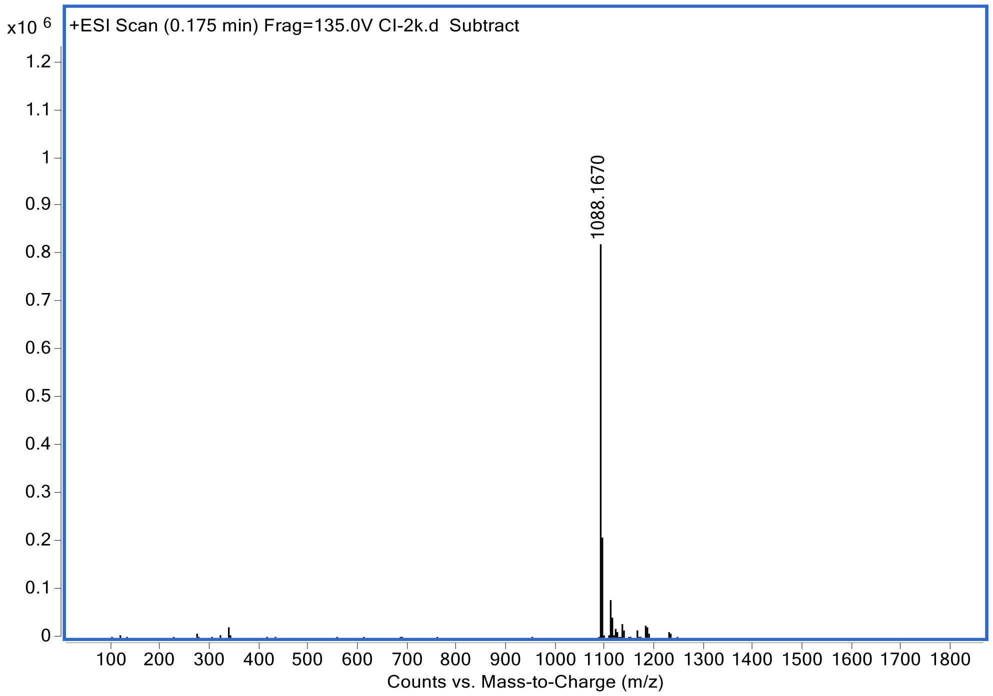

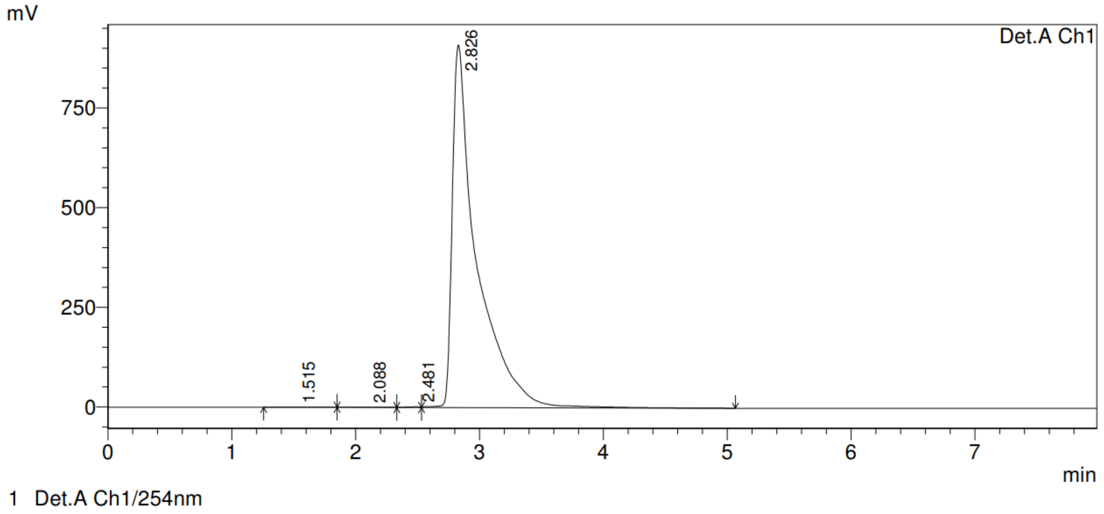

PeakTable

¼ì²âÆ÷ A Ch1 254nm

Peak#	Ret. Time	Area	Height	Area %	Height %
1	1.515	1734	98	0.014	0.011
2	2.088	8606	392	0.069	0.043
3	2.481	13862	1619	0.111	0.178
4	2.826	12461291	909363	99.806	99.769
Total		12485493	911472	100.000	100.000

^{1}H NMR, ^{13}C NMR, ^{19}F NMR, COSY, HSQC, HRMS and HPLC of IC-2a

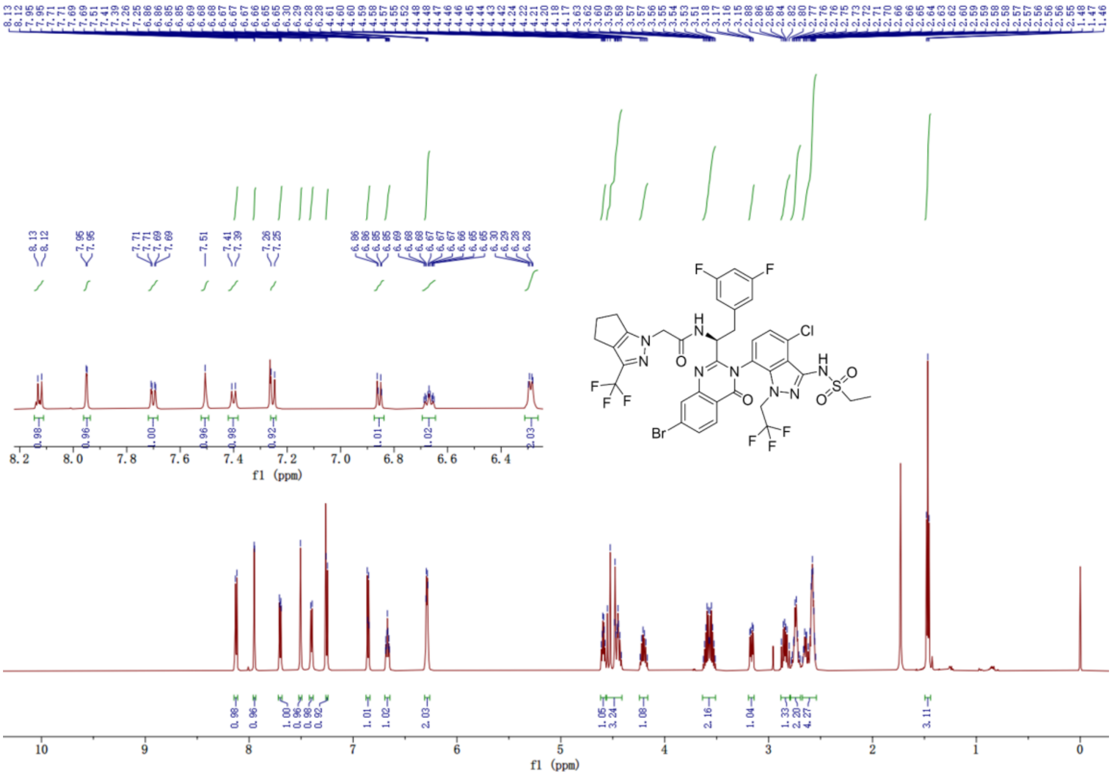

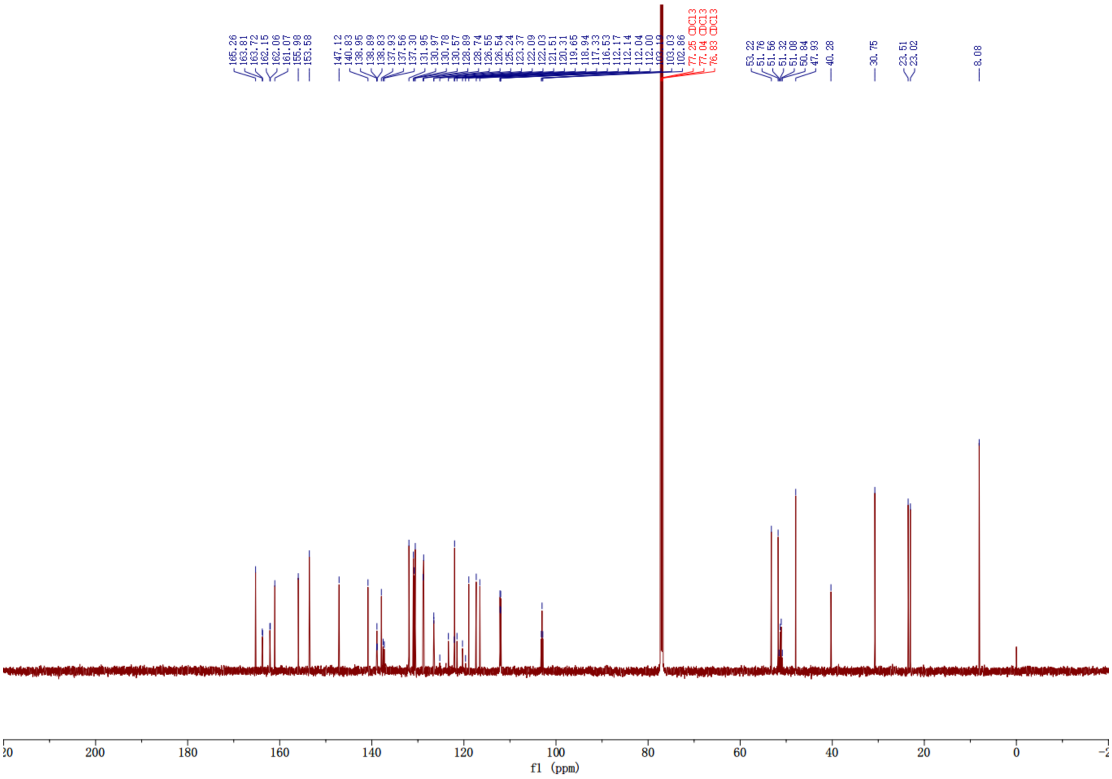

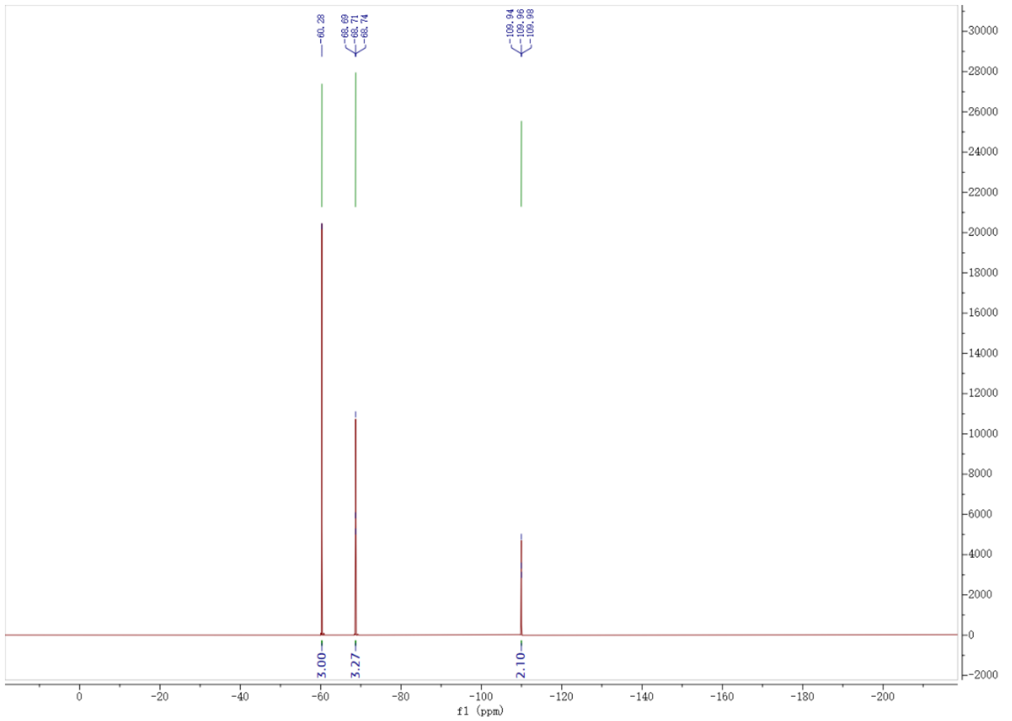

-60.28
-68.69
-68.71
-68.74
-109.94
-109.96
-109.98
3.00
3.27
2.10
f1 (ppm)

1

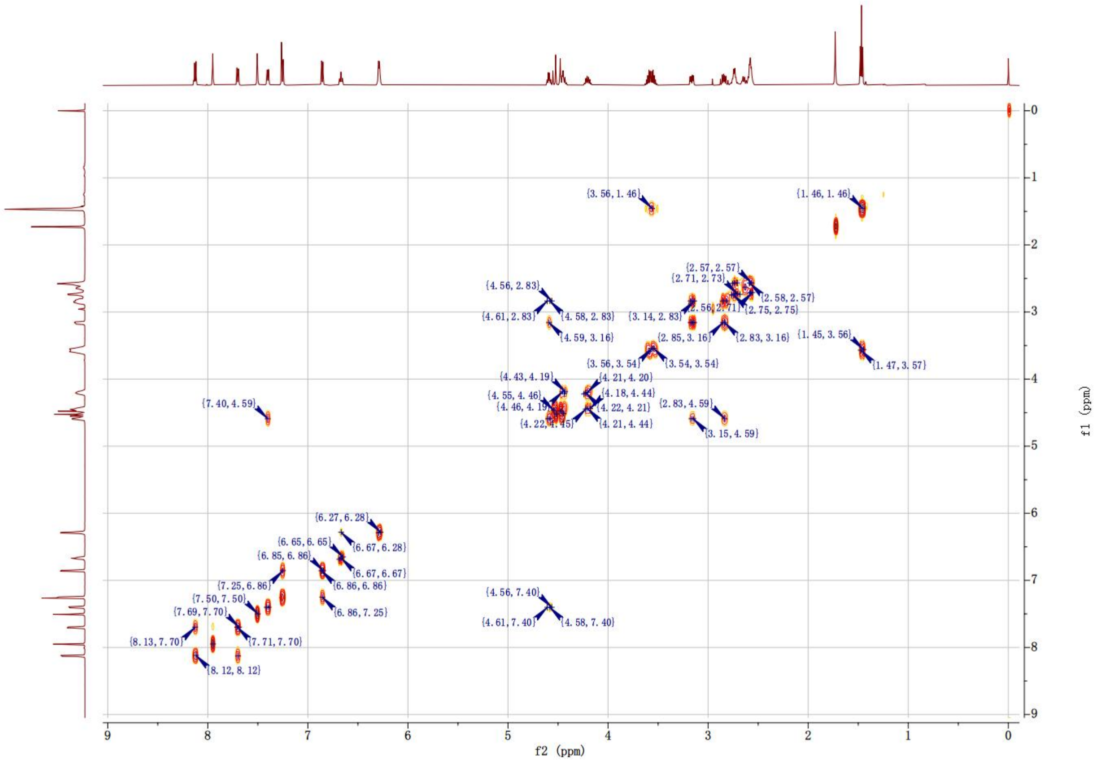

f2 (ppm)
f1 (ppm)

2

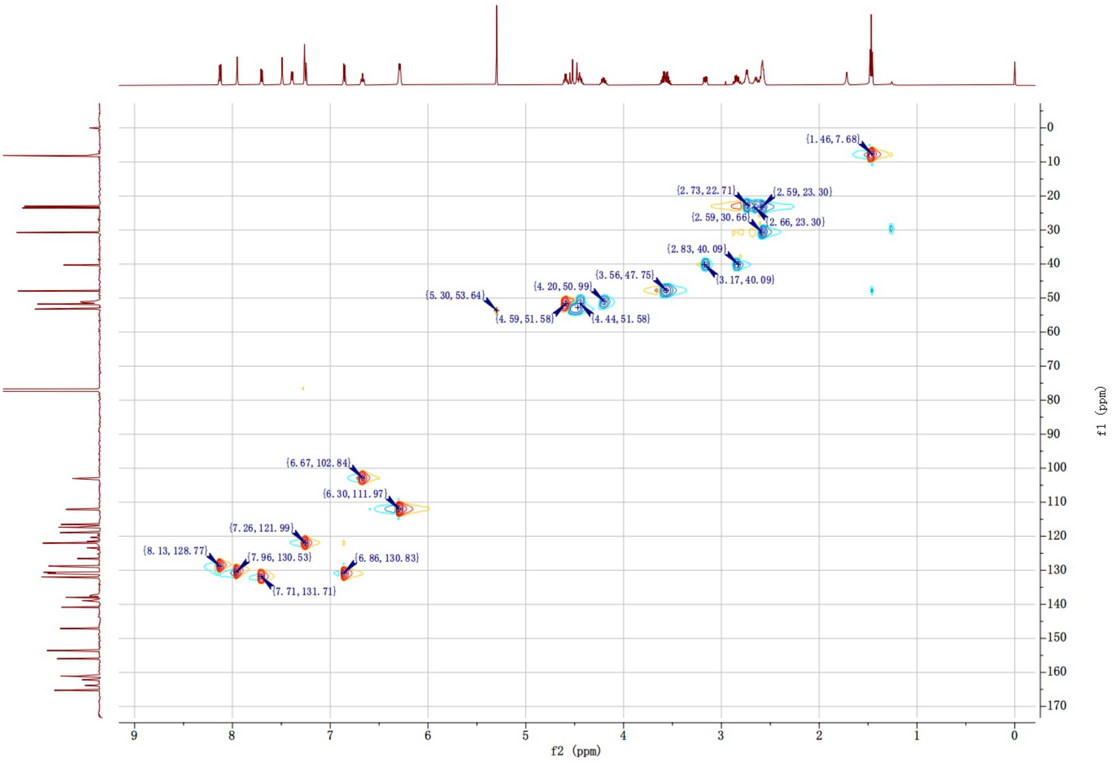
{1.46,7.68}
{2.73,22.71}
{2.59,23.30}
{2.59,30.66}
{2.66,23.30}
{2.83,40.09}
{3.17,40.09}
{3.56,47.75}
{4.20,50.99}
{5.30,53.64}
{4.59,51.58}
{4.44,51.58}
{6.67,102.84}
{6.30,111.97}
{7.26,121.99}
{8.13,128.77}
{7.96,130.53}
{6.86,130.83}
{7.71,131.71}
f2 (ppm)
f1 (ppm)

1

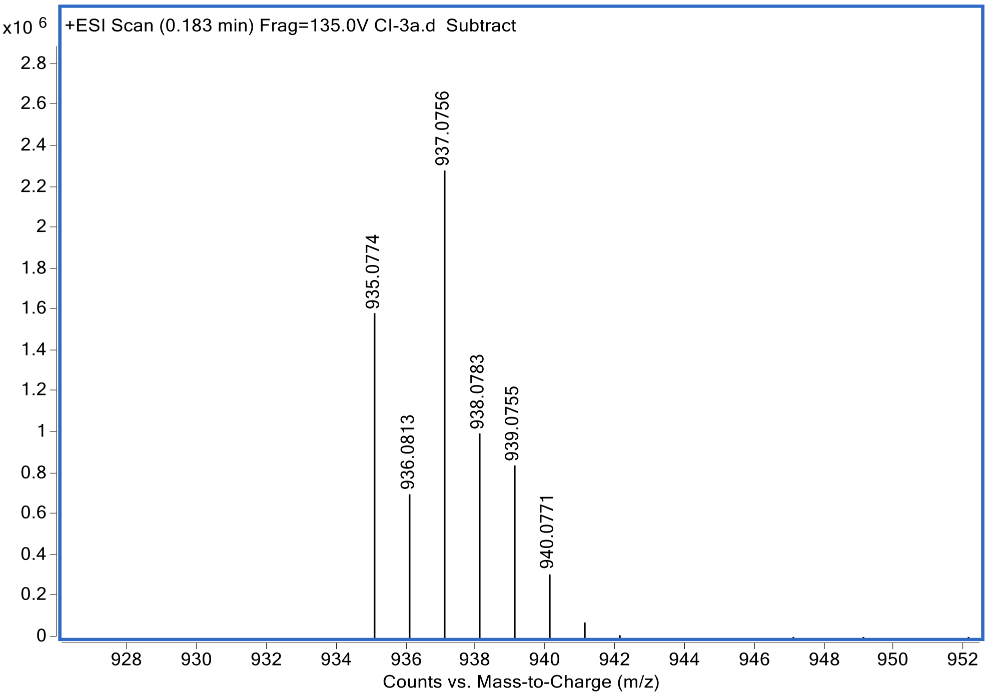
x10 6
+ESI Scan (0.183 min) Frag=135.0V CI-3a.d Subtract
935.0774
936.0813
937.0756
938.0783
939.0755
940.0771
Counts vs. Mass-to-Charge (m/z)

2

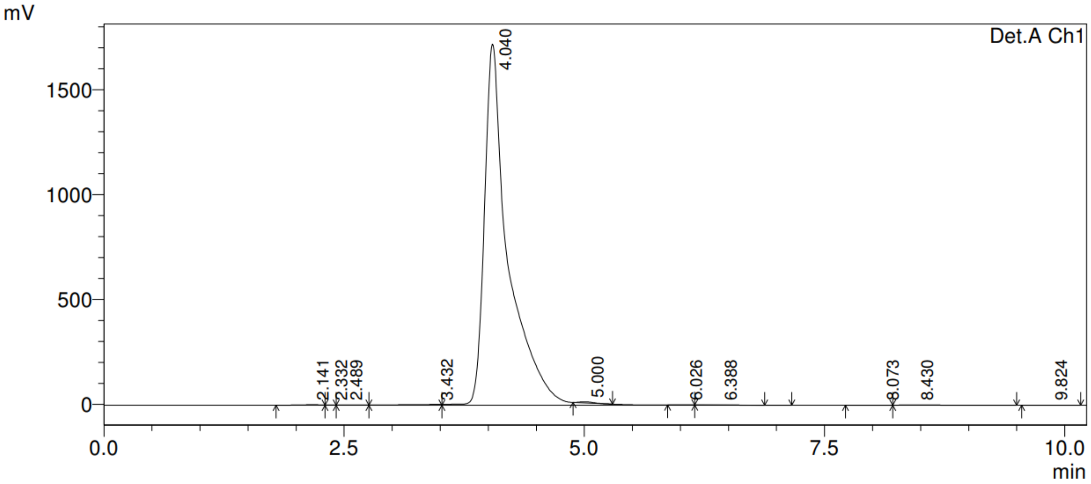

1 Det.A Ch1/254nm

PeakTable

¼ì²âÆ÷ A Ch1 254nm

Peak#	Ret. Time	Area	Height	Area %	Height %
1	2.141	34683	2941	0.115	0.169
2	2.332	9386	1391	0.031	0.080
3	2.489	24345	1297	0.081	0.075
4	3.432	94955	3209	0.316	0.185
5	4.040	29786263	1720821	99.131	99.045
6	5.000	55162	5565	0.184	0.320
7	6.026	1634	144	0.005	0.008
8	6.388	10534	607	0.035	0.035
9	8.073	4664	284	0.016	0.016
10	8.430	23969	1057	0.080	0.061
11	9.824	1715	97	0.006	0.006
Total		30047312	1737413	100.000	100.000

^1H NMR, ^{13}C NMR and HRMS of IC-2b

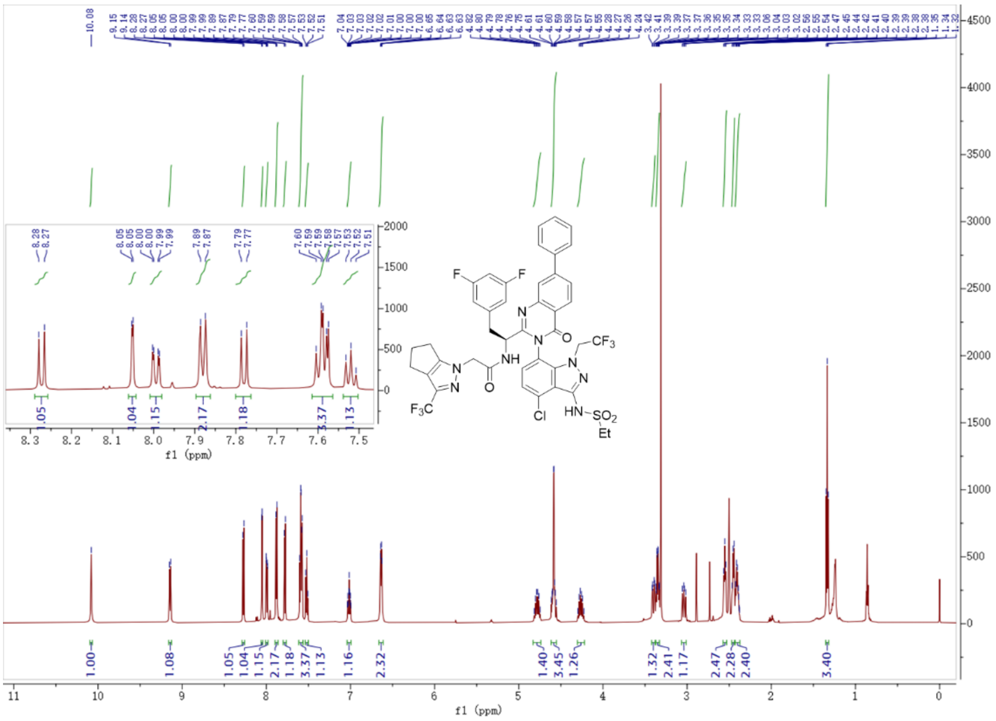

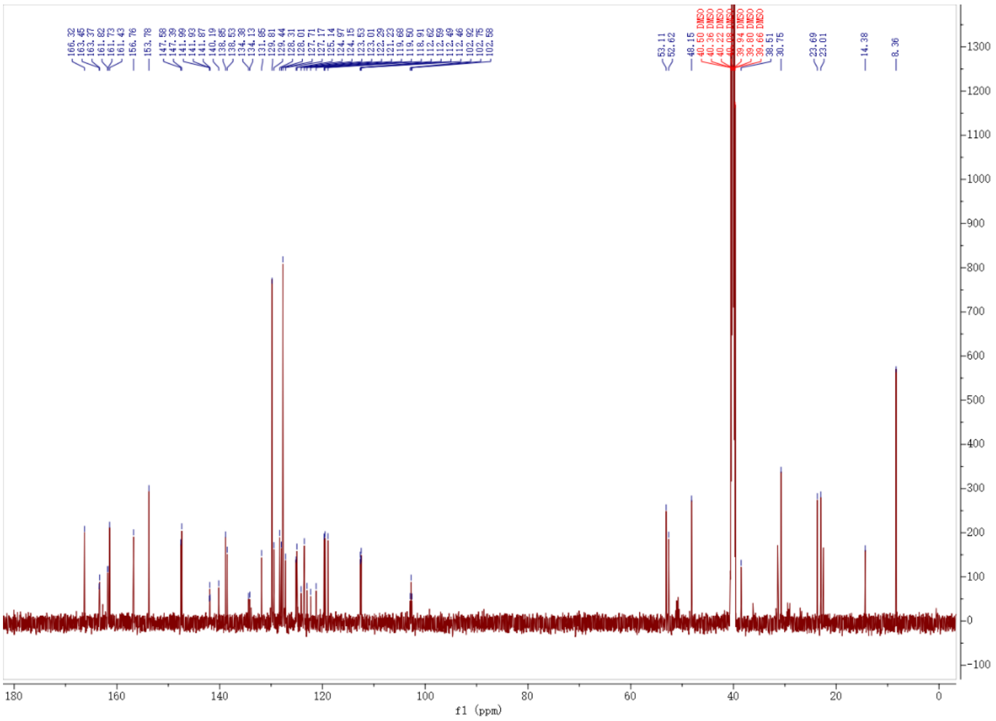

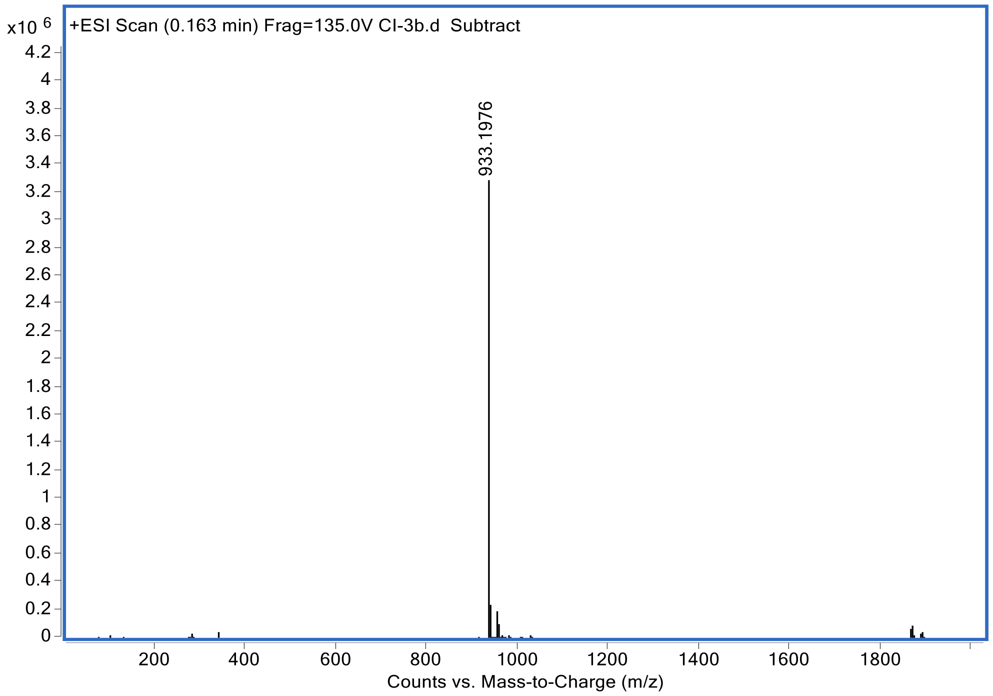

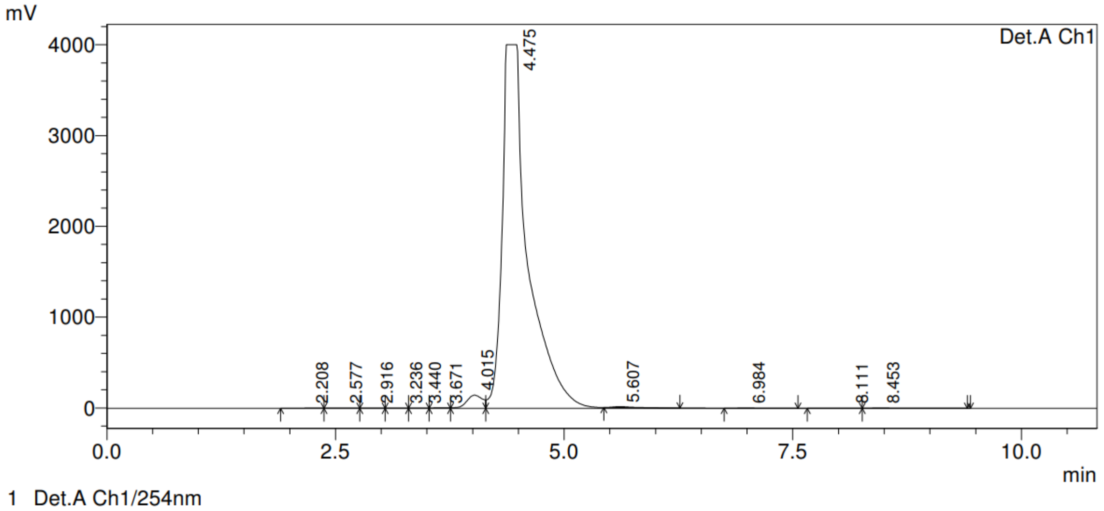

PeakTable

¼ì²âÆ÷ A Ch1 254nm

Peak#	Ret. Time	Area	Height	Area %	Height %
1	2.208	26513	2763	0.033	0.066
2	2.577	35968	1667	0.044	0.040
3	2.916	27960	1865	0.035	0.045
4	3.236	36417	2949	0.045	0.071
5	3.440	43667	3449	0.054	0.082
6	3.671	80882	6992	0.100	0.167
7	4.015	1736425	144228	2.143	3.449
8	4.475	78824368	4003318	97.276	95.745
9	5.607	178246	11960	0.220	0.286
10	6.984	10518	574	0.013	0.014
11	8.111	9690	576	0.012	0.014
12	8.453	21172	906	0.026	0.022
Total		81031827	4181248	100.000	100.000

^{1}H NMR, ^{13}C NMR, ^{19}F NMR, COSY, HSQC, HRMS and HPLC of IC-2i

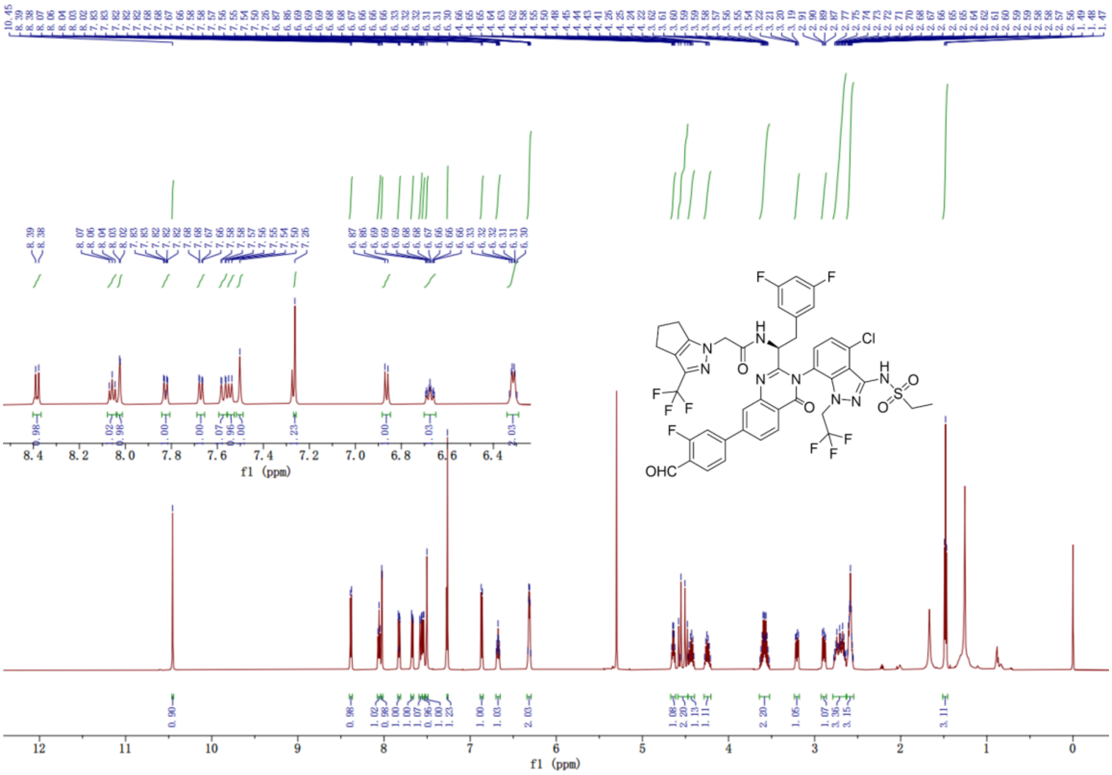

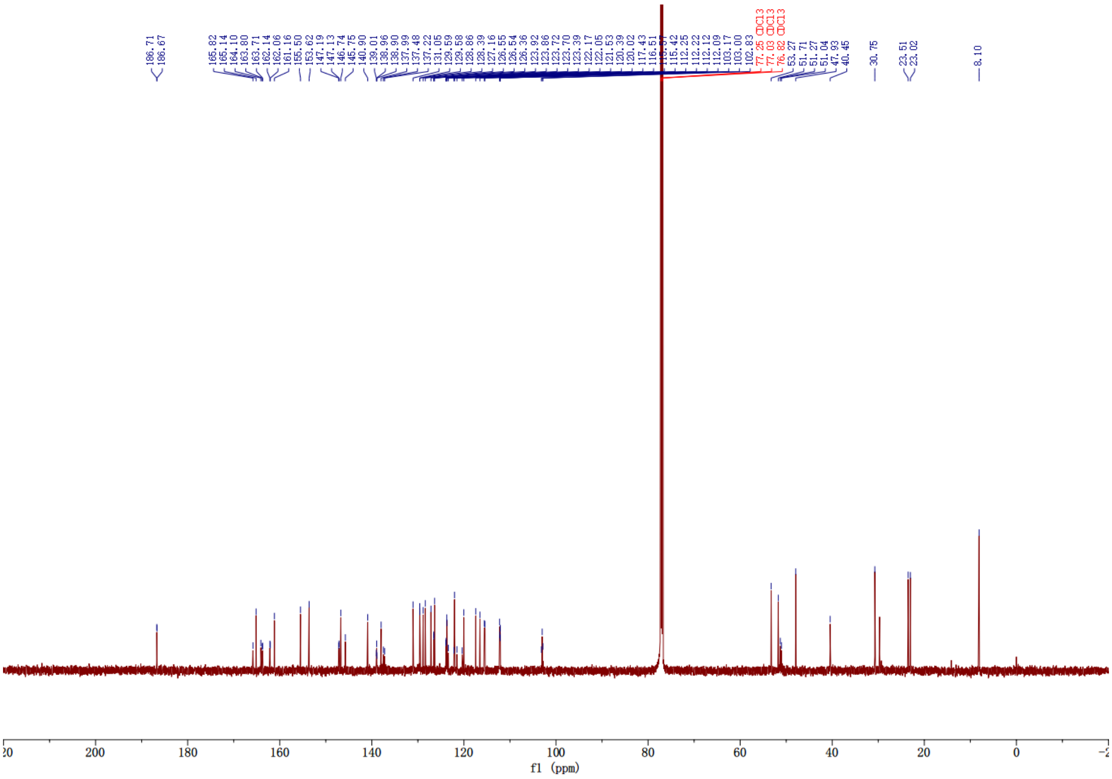

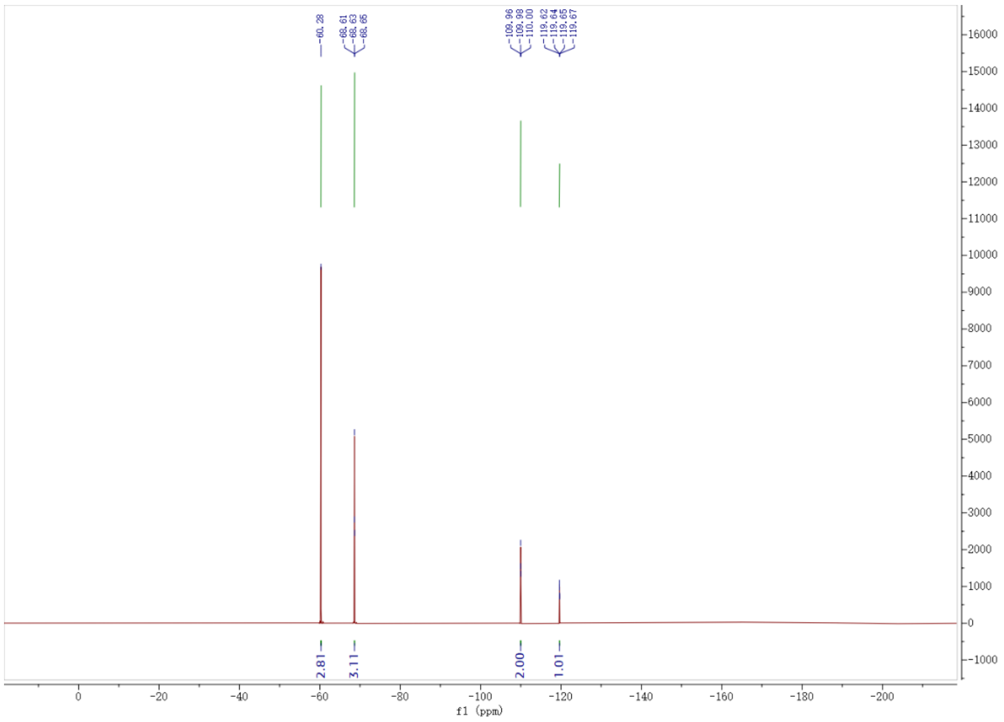
-60.28
-68.61
-68.63
-68.65
-109.96
-109.98
-110.00
-119.62
-119.64
-119.65
-119.67
2.81
3.11
2.00
1.01
f1 (ppm)

1

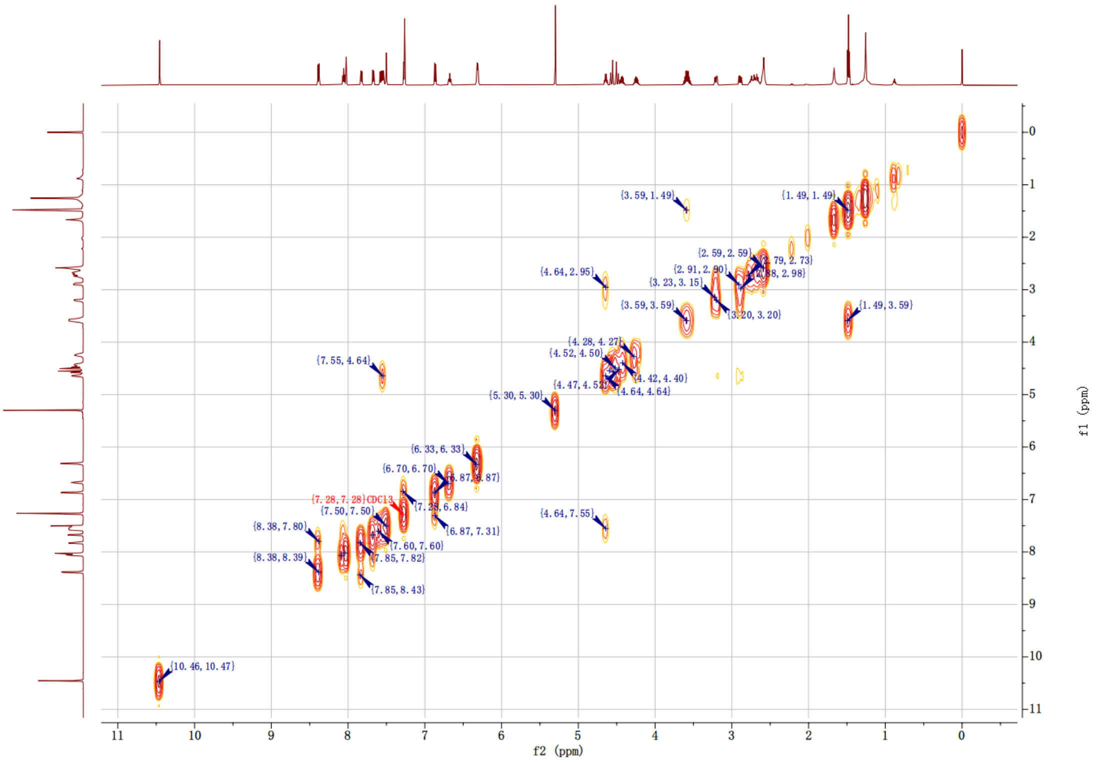
{3.59, 1.49}
{1.49, 1.49}
{2.59, 2.59}
{4.64, 2.95}
{3.23, 3.15}
{3.59, 3.59}
{3.20, 3.20}
{1.49, 3.59}
{4.28, 4.27}
{4.52, 4.50}
{7.55, 4.64}
{4.42, 4.40}
{4.47, 4.52}
{4.64, 4.64}
{5.30, 5.30}
{6.33, 6.33}
{6.70, 6.70}
{7.28, 7.28}CDCl3
{7.50, 7.50}
{4.64, 7.55}
{8.38, 7.80}
{6.87, 7.31}
{7.60, 7.60}
{8.38, 8.39}
{7.85, 7.82}
{7.85, 8.43}
{10.46, 10.47}
f2 (ppm)
f1 (ppm)

2

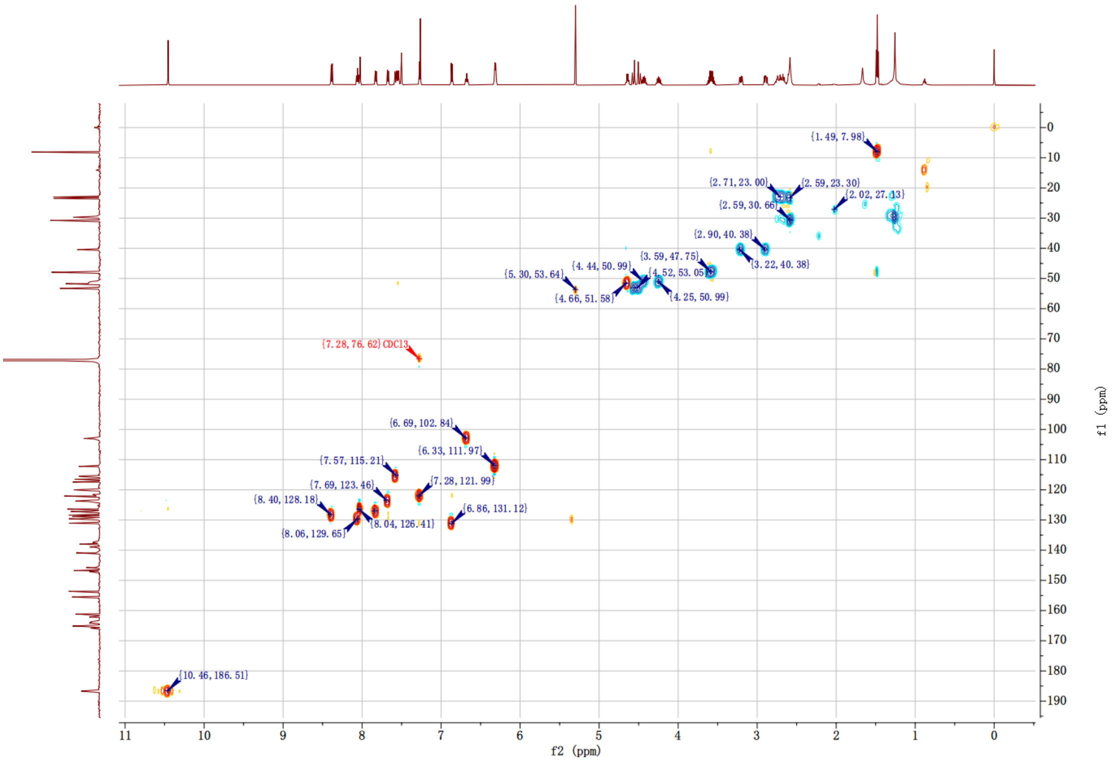
{1.49, 7.98}
{2.71, 23.00}
{2.59, 23.30}
{2.02, 27.13}
{2.59, 30.66}
{2.90, 40.38}
{3.22, 40.38}
{3.59, 47.75}
{4.44, 50.99}
{4.52, 53.05}
{5.30, 53.64}
{4.66, 51.58}
{4.25, 50.99}
{7.28, 76.62} CDCl3
{6.69, 102.84}
{6.33, 111.97}
{7.57, 115.21}
{7.28, 121.99}
{7.69, 123.46}
{6.86, 131.12}
{8.40, 128.18}
{8.04, 126.41}
{8.06, 129.65}
{10.46, 186.51}
f2 (ppm)
f1 (ppm)

1

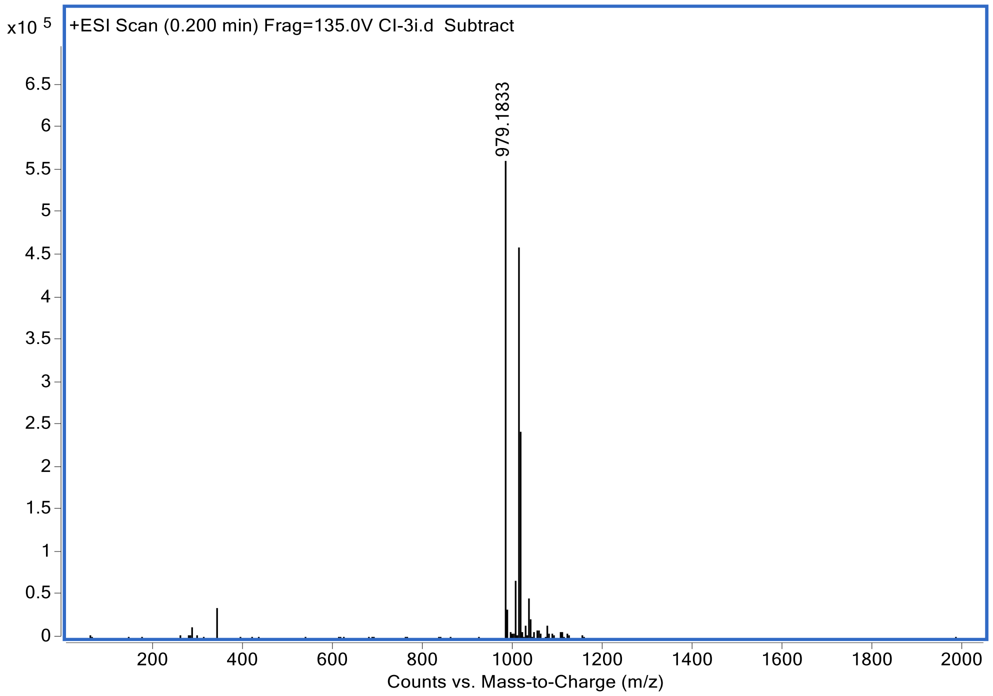
x10 5
+ESI Scan (0.200 min) Frag=135.0V CI-3i.d Subtract
979.1833
Counts vs. Mass-to-Charge (m/z)

2

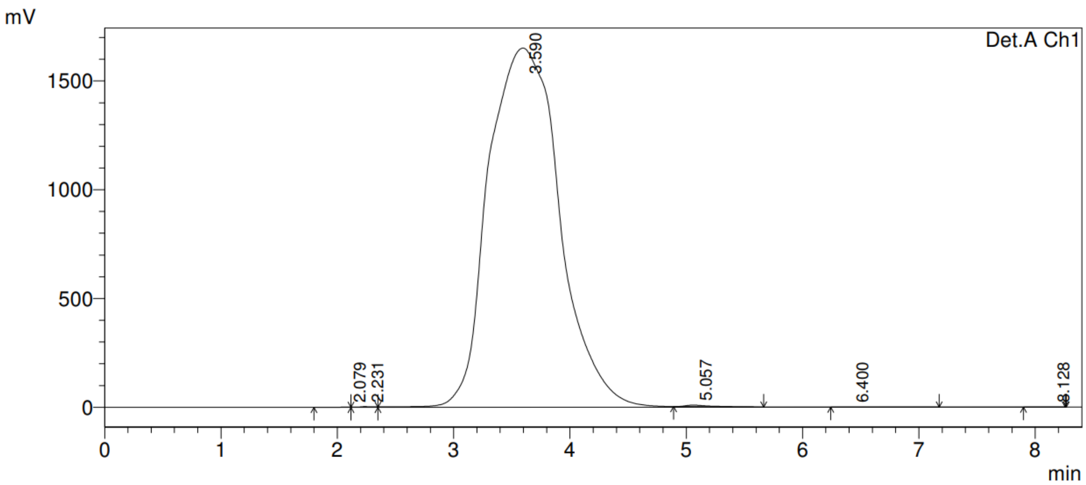

1 Det.A Ch1/254nm

PeakTable

¼ì²âÆ÷ A Ch1 254nm

Peak#	Ret. Time	Area	Height	Area %	Height %
1	2.079	4719	774	0.007	0.047
2	2.231	26685	3208	0.038	0.193
3	3.590	70570470	1651089	99.805	99.321
4	5.057	100844	7021	0.143	0.422
5	6.400	3400	84	0.005	0.005
6	8.128	2229	196	0.003	0.012
Total		70708347	1662371	100.000	100.000

^{1}H NMR, ^{13}C NMR, ^{19}F NMR and HRMS of IC-2k

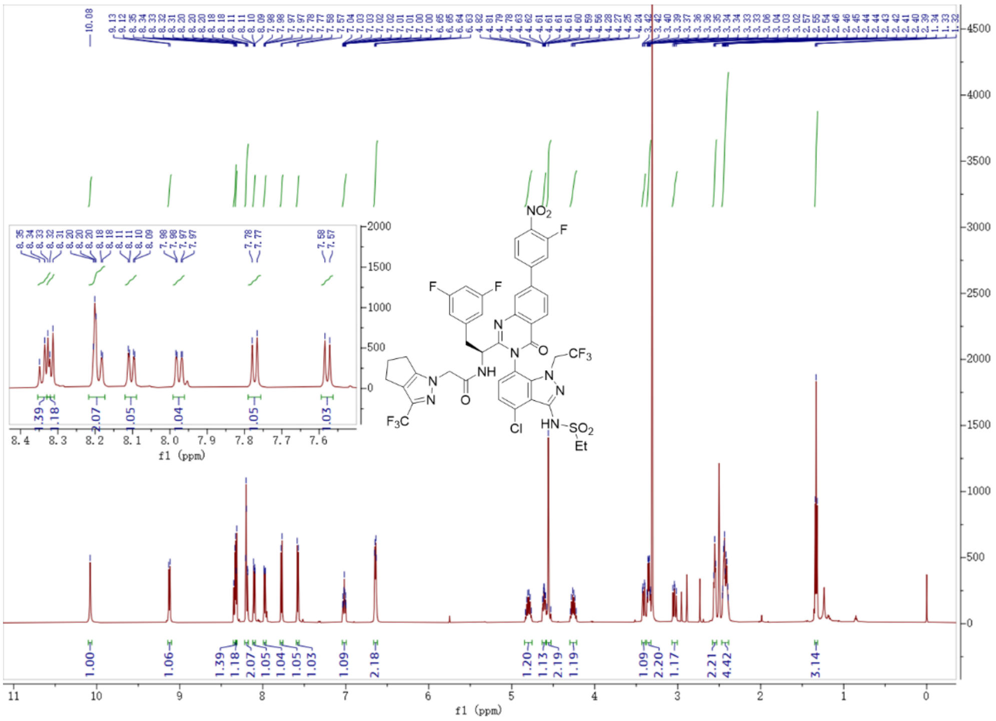

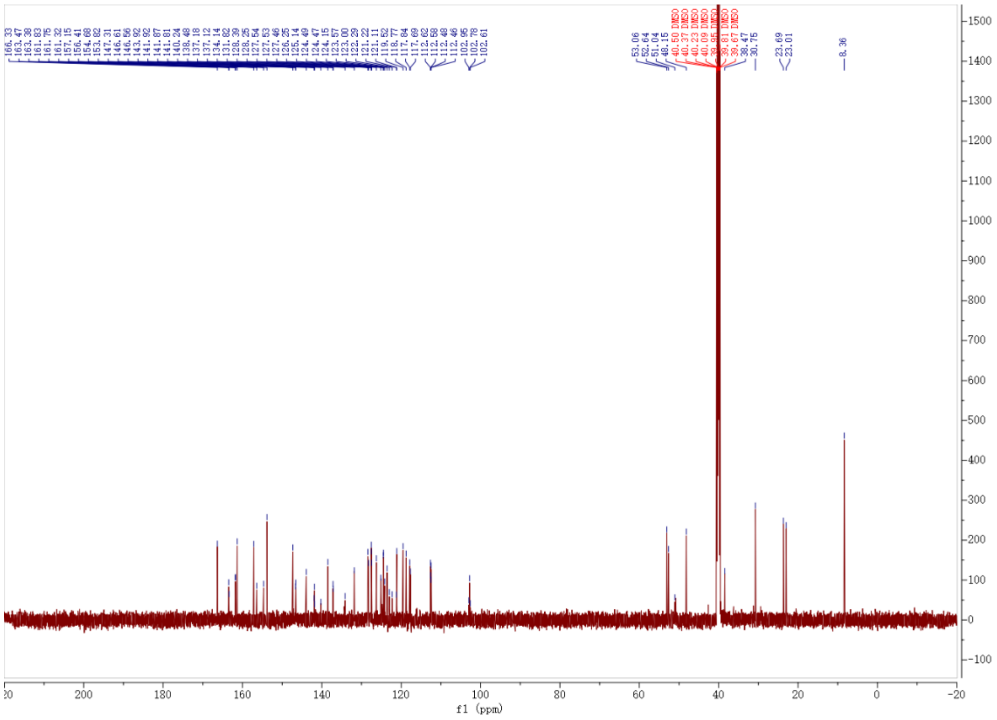

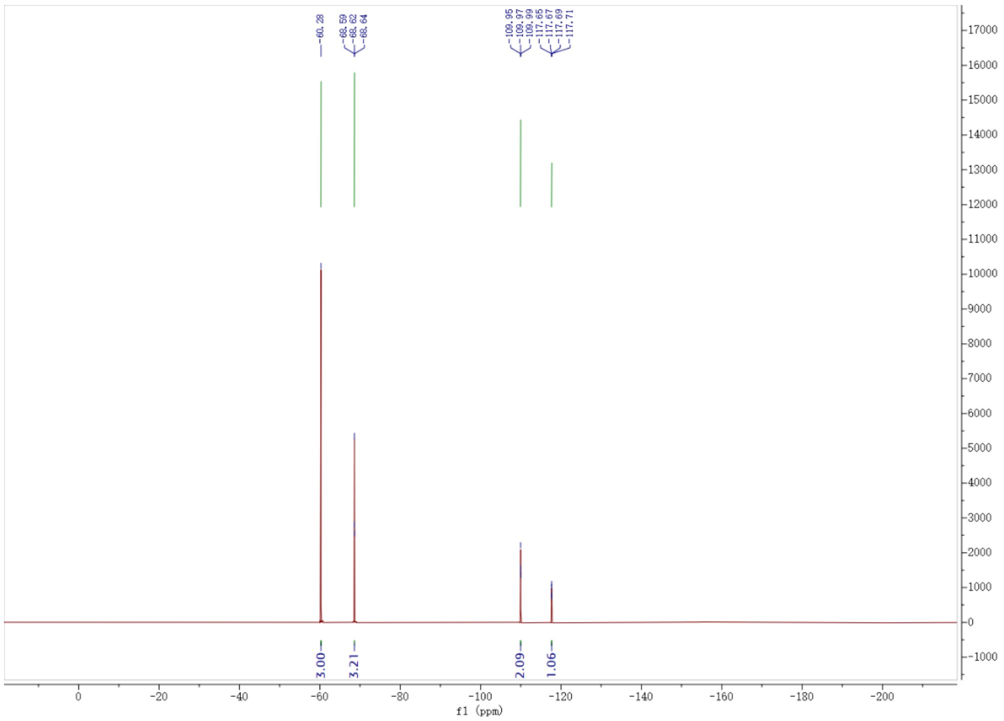

-60.28
-68.59
-68.62
-68.64
-109.95
-109.97
-109.99
-117.65
-117.67
-117.69
-117.71
3.00
3.21
2.09
1.06
f1 (ppm)

1

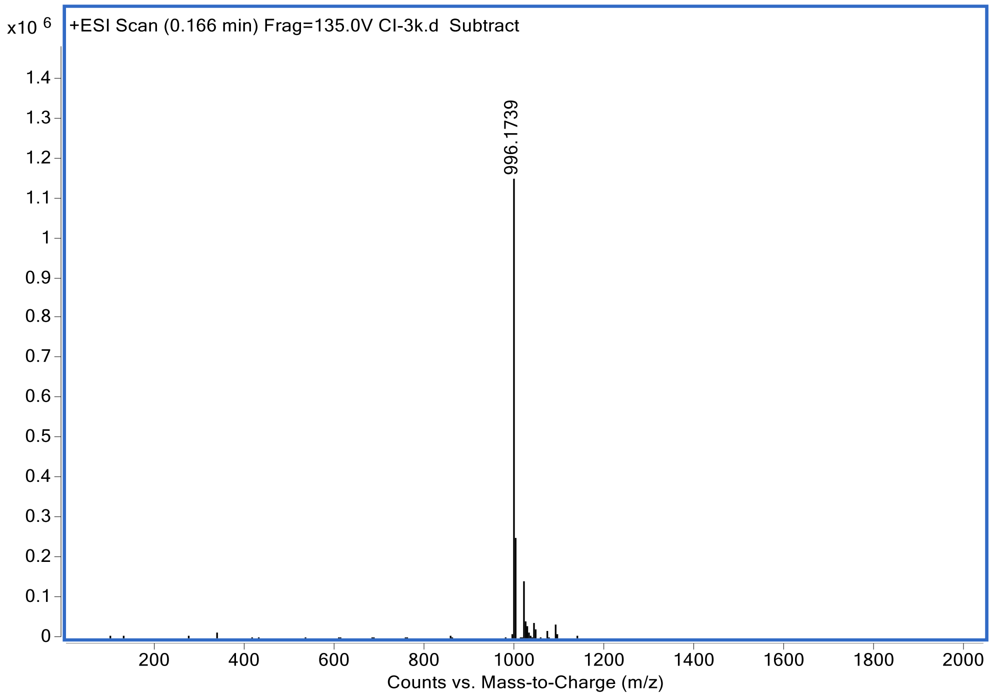

x10 6
+ESI Scan (0.166 min) Frag=135.0V CI-3k.d Subtract
996.1739
Counts vs. Mass-to-Charge (m/z)

2

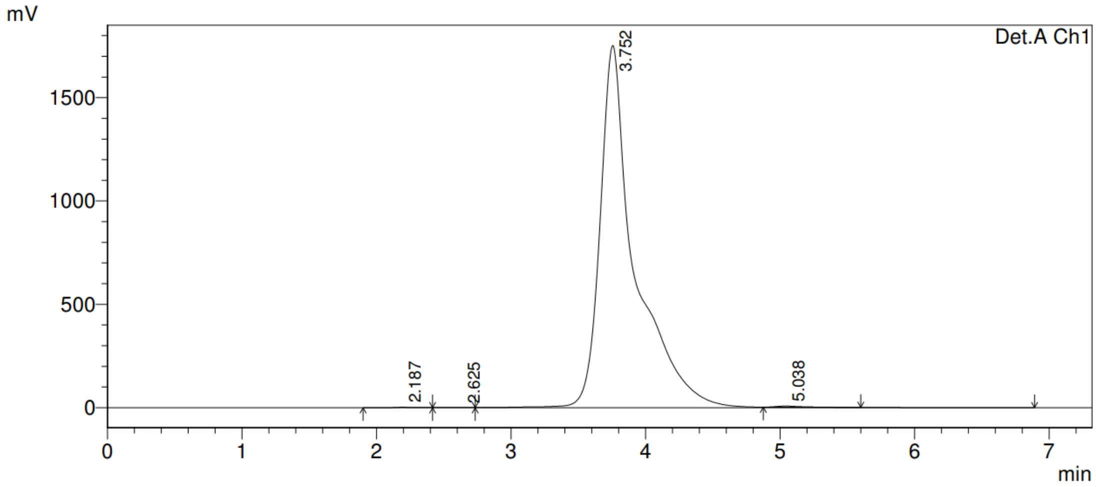

1 Det.A Ch1/254nm

PeakTable

¼ì²âÆ÷ A Ch1 254nm

Peak#	Ret. Time	Area	Height	Area %	Height %
1	2.187	23689	2105	0.075	0.119
2	2.625	19373	1121	0.061	0.064
3	3.752	31604517	1752675	99.622	99.501
4	5.038	76781	5565	0.242	0.316
Total		31724360	1761466	100.000	100.000

SIII. Anti-viral activity of Compounds

Table S1. *In vitro* anti-HIV-1 activity, cytotoxicity, and selective index of the IA series

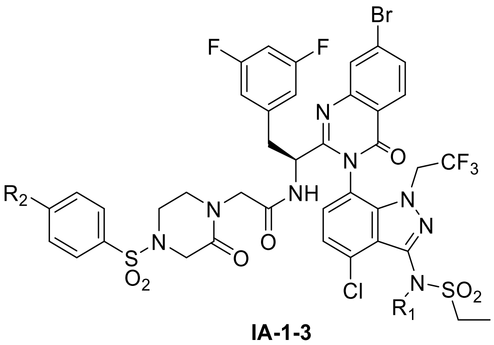

IA-1-3

Cpds.	R_1	R_2	EC_{50} [a](nM)	CC_{50} [b](nM)	SI[c]
**IA-1**	$-SO_2Et$	$-NO_2$	502.94 ± 21.98	> 879.26	> 1.8
**IA-2**	-H	$-NO_2$	*[e]	*	N.D.[d]
**IA-3**	$-SO_2Et$	$-NH_2$	*	*	N.D.
**PF74**	-	-	542.85 ± 129.25	**[f]	N.D.
**11L**	-	-	105.23 ± 25.88	**	N.D.
**LEN**	-	-	0.07 ± 0.01	**	N.D.

[a]EC_{50}: the concentration of the compound required to achieve 50% protection of MT-4 cells against HIV-1-induced cytopathic effect, determined in at least triplicate against HIV-1 in MT-4 cells.

[b]CC_{50}: the compound concentration required to reduce the viability of uninfected cells by 50%, determined in at least triplicate against HIV-1 in MT-4 cells; values were averaged from at least three independent experiments.

[c]SI: selective index, CC_{50}/EC_{50};

[d]N.D.: not determined;

[e]*: For the poor selectivity of compounds, testing was abandoned (SI < 5);

[f]**: The compound did not significantly reduce cell survival at the maximum test concentration (**PF74**: 1000 ng/mL, **11L**: 300 ng/mL, **LEN**: 25 ng/mL).

Table S2. *In vitro* anti-HIV-1 activity, cytotoxicity, and selective index of IB and IC series

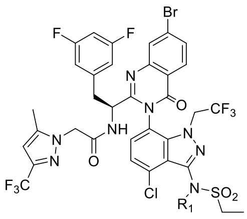

IB-1a / IB-2a

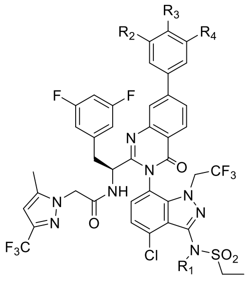

IB-1(b-d) / IB-2(b-d)

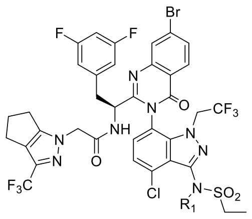

IC-1a / IC-2a

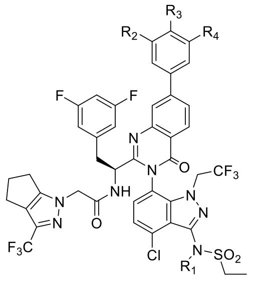

IC-1(b-d) / IC-2(b-d)

Cpds.	R_1	R_2	R_3	R_4	EC_{50} [a](nM)	CC_{50} [b](nM)	SI[c]
**IB-1a**	$-SO_2Et$	-	-	-	113.76 ± 14.97	753.38 ± 65.86	6.6
**IB-1b**	$-SO_2Et$	-H	-CN	-F	119.92 ± 23.98	> 959.37	> 8.0
**IB-1c**	$-SO_2Et$	-F	-CN	-F	306.51 ± 70.73	> 943.09	> 3.1
**IB-1d**	$-SO_2Et$	-H	$-NO_2$	-H	201.08 ± 62.24	> 957.53	> 4.8
**IB-2a**	-H	-	-	-	36.26 ± 7.25	> 1098.85	> 30.3
**IB-2b**	-H	-H	-CN	-F	26.31 ± 7.16	> 1052.37	> 40.0
**IB-2c**	-H	-F	-CN	-F	28.92 ± 5.27	> 1032.81	> 35.7
**IB-2d**	-H	-H	$-NO_2$	-H	22.05 ± 3.78	> 1050.17	> 47.6
**IC-1a**	$-SO_2Et$	-	-	-	21.40 ± 2.72	> 972.58	> 45.5
**IC-1b**	$-SO_2Et$	-H	-H	-H	24.38 ± 6.73	> 975.24	> 40.0
**IC-1c**	$-SO_2Et$	-H	-CN	-H	10.47 ± 2.57	> 952.02	> 90.9
**IC-1d**	$-SO_2Et$	-H	-CN	-F	9.36 ± 1.50	> 935.99	> 100.0
**IC-1e**	$-SO_2Et$	-F	-CN	-F	50.63 ± 8.84	> 920.49	> 18.2
**IC-1f**	$-SO_2Et$	-F	-H	-F	108.35 ± 32.98	> 942.18	> 8.7
**IC-1g**	$-SO_2Et$	-F	-F	-F	66.71 ± 8.15	> 926.47	> 13. 9
**IC-1h**	$-SO_2Et$	-H	-H	-F	17.25 ± 4.12	913.38 ± 71.88	53.0
**IC-1i**	$-SO_2Et$	-H	-CHO	-F	7.01 ± 2.34	> 934.24	> 133.3
**IC-1j**	$-SO_2Et$	-H	$-NO_2$	-H	76.61 ± 25.22	> 934.25	> 12.2
**IC-1k**	$-SO_2Et$	-H	$-NO_2$	-F	37.67 ± 5.70	> 918.81	> 24.4
**IC-2a**	-H	-	-	-	0.67 ± 0.14	> 1068.30	> 1594.5
**IC-2b**	-H	-H	-H	-H	1.66 ±0.16	> 1071.49	> 645.5
**IC-2c**	-H	-H	-CN	-H	1.72 ± 0.26	> 1043.53	> 606.7
**IC-2d**	-H	-H	-CN	-F	5.63 ± 2.25	> 1024.30	> 181.9
**IC-2e**	-H	-F	-CN	-F	4.53 ± 1.11	> 1005.76	> 222.0
**IC-2f**	-H	-F	-H	-F	6.29 ± 0.91	> 1031.71	> 164.0
**IC-2g**	-H	-F	-F	-F	6.69 ± 0.91	690.81 ± 63.81	103.3
**IC-2h**	-H	-H	-H	-F	1.58 ± 0.45	> 1051.23	> 665.3
**IC-2i**	-H	-H	-CHO	-F	0.65 ± 0.27	> 1021.16	> 1571.0

**IC-2j**	-H	-H	$-NO_2$	-H	1.43 ± 0.56	> 1022.21	> 714.8
**IC-2k**	-H	-H	$-NO_2$	-F	0.94 ± 0.08	> 1003.75	> 1067.8
**PF74**	-			-	542.85 ± 129.25	**[e]	N.D.[d]
**11L**	-			-	105.23 ± 25.88	**	N.D.
**LEN**	-			-	0.07 ± 0.01	**	N.D.

[a]EC_{50}: the concentration of the compound required to achieve 50% protection of MT-4 cells against HIV-1-induced cytopathic effect, determined in at least triplicate against HIV-1 in MT-4 cells.

[b]CC_{50}: the compound concentration required to reduce the viability of uninfected cells by 50%, determined in at least triplicate against HIV-1 in MT-4 cells; values were averaged from at least three independent experiments.

[c]SI: selective index, CC_{50}/EC_{50};

[d]N.D.: not determined;

[e]**: The compound did not significantly reduce cell survival at the maximum test concentration (**PF74**: 1000 ng/mL, **11L**: 300 ng/mL, **LEN**: 25 ng/mL).

Table S3. *In vitro* anti-HIV activity, cytotoxicity, and selective index of representative compounds

Cpds	EC_{50} [a]		CC_{50} [b]	SI [c]	
	HIV-1 IIIB (nM)	HIV-2 ROD (μM)	(μM)	HIV-1	HIV-2
**IA-1**	483 ± 16	0.563 ± 0.79	> 110	> 227.3	> 195.3
**IB-1a**	239 ± 90	71.6 ± 33	> 125	> 520.8	> 1.7
**IB-2a**	56.0 ± 29	0.758 ± 0.077	14.1 ± 0.47	251.2	18.6
**IB-1d**	1111 ± 22	> 120	> 120	> 107.8	> 1
**IB-2d**	21.0 ± 7.4	0.284 ± 0.021	> 131	> 6250.0	> 463.0
**IC-1a**	14.6 ± 3.9	8.08 ± 1.9	> 122	> 8333.3	> 15.0
**IC-2a**	3.10 ± 0.75	0.331 ± 0.053	13.7 ± 0.77	4417.2	41.3
**IC-1i**	598 ± 121	49.6 ± 20	> 117	> 195.3	> 2.4
**IC-2i**	5.82 ± 1.6	0.153 ± 0.051	14.9 ± 1.2	2552.6	97
**IC-1k**	395 ± 110	50.4 ± 23	> 115	> 290.7	> 2.3
**IC-2k**	5.82 ± 1.2	0.100 ± 0.030	90.1 ± 19	15469	897.2
**11L**	276 ± 35	0.0136 ± 0.0071	114 ± 3.4	412	8334
**PF74**	1833 ± 73	> 284	283 ± 5.9	155	< 1
**LEN**	0.134 ± 0.0062	0.00300 ± 0.00083	> 129	> 961539	> 43103

[a]EC_{50}: the concentration of the compound required to achieve 50% protection of MT-4 cells against HIV-induced cytotoxicity effect, determined in at least triplicate against HIV in MT-4 cells;

[b]CC_{50}: the concentration of the compound required to reduce the viability of uninfected cells by 50%, determined in at least triplicate against HIV in MT-4 cells; values were averaged from at least three independent experiments;

[c]SI: selectivity index, the ratio of CC_{50}/ EC_{50}.

SIV. Antiviral Activity of IC-2i Against HIV-1 Mutants

Table S4. Antiviral Activity of IC-2i on Mutants

Mutant	IC-2i EC_{50} (nM)	Fold Change	LEN Fold Change[a]
**WT**	0.65 ± 0.27 [a]	-	-
**Q67H**	44.9 ± 14.6	69	~ 5
**N74D**	7.07 ± 1.0	11	~ 20
**K70R**	56.6 ± 9.1	87	little or no affect
**Q67H/K70R**	95.59 ± 18.8	147	~ 20

[a] The data are from HIV drug resistance database[1].

SV. SPR assays

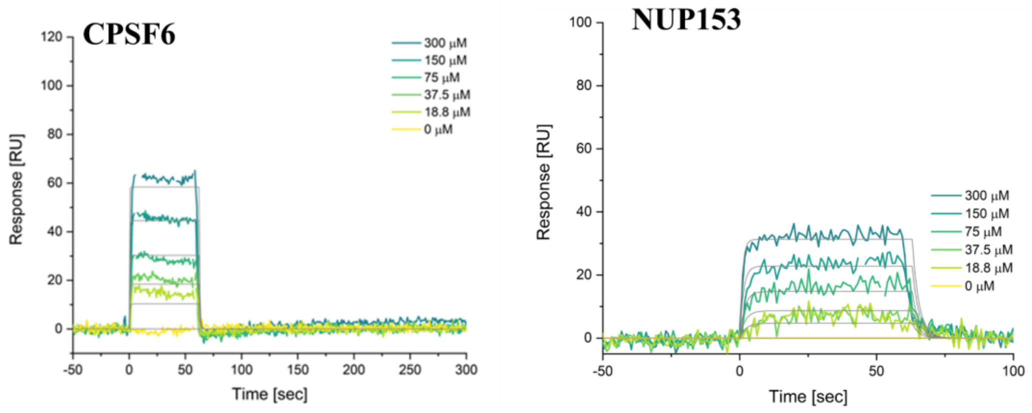

Figure S1. SPR sensorgrams of CPSF6 peptide and NUP153 peptide binding to hexameric HIV-1 CA (n=3)

Table S5. SPR results of test and control compounds binding to CA

Cpds	Hexamer				Monomer			
	k_a (s^{-1})	k_d (s^{-1})	Residence Time (min)	K_D (nM)	k_a (s^{-1})	k_d (s^{-1})	Residence Time (min)	K_D (nM)
**IB-2a**	6.41 $E^7 \pm$ 6.35 E^5	1.08 $E^{-3} \pm$ 4.01 E^{-6}	15.4	23.0 ± 12.6	4.18 $E^5 \pm$ 1.50 E^3	1.21 $E^{-2} \pm$ 1.98 E^{-4}	1.38	30.3 ± 5.8
**IC-2a**	3.71 $E^7 \pm$ 2.88 E^5	2.59 $E^{-4} \pm$ 8.52 E^{-7}	64.4	7.9 ± 4.7	2.33 $E^5 \pm$ 4.14 E^3	7.40 $E^{-3} \pm$ 6.69 E^{-5}	2.25	32.2 ± 3.9
**IC-2i**	3.37 $E^7 \pm$ 1.23 E^5	9.25 $E^{-5} \pm$ 2.66 E^{-7}	180	2.7 ± 0.5	1.86 $E^5 \pm$ 2.01 E^3	3.56 $E^{-3} \pm$ 3.68 E^{-5}	4.69	19.1 ± 1.1
**IC-2k**	2.58 $E^7 \pm$ 2.17 E^5	2.09 $E^{-4} \pm$ 5.77 E^{-7}	79.9	8.2 ± 1.5	9.96 $E^4 \pm$ 1.97 E^3	5.24 $E^{-3} \pm$ 5.71 E^{-5}	3.18	53.7 ± 8.9
**PF74**	5.78 $E^8 \pm$ 1.28 E^7	1.67 $E^{-2} \pm$ 1.85 E^{-4}	0.998	28.9 ± 1	8.95 $E^4 \pm$ 6.19 E^3	2.34 $E^{-1} \pm$ 1.32 E^{-2}	0.07	2.62 $E^3 \pm$ 0.44 E^3
**CPSF6**	5.91 $E^3 \pm$ 5.43 E^2	8.01 $E^{-1} \pm$ 6.74 E^{-2}	-	1.37 $E^5 \pm$ 1.65 E^4	-	-	-	-
**NUP153**	3.73 $E^3 \pm$ 3.81 E^2	5.29 $E^{-1} \pm$ 4.69 E^{-2}	-	1.48 $E^5 \pm$ 2.20 E^4	-	-	-	-
**11l**	1.04 $E^{10} \pm$ 4.43 E^{12}	2.30 $E^1 \pm$ 9.77 E^3	0.000725	2.8 $E^3 \pm$ 0.4 E^3	8.50 $E^4 \pm$ 4.21 E^3	2.21 $E^{-1} \pm$ 8.90 E^{-3}	0.08	2.71 $E^3 \pm$ 0.62 E^3
**LEN**	5.00 $E^7 \pm$ 1.81 E^5	4.66 $E^{-5} \pm$ 3.29 E^{-7}	357	0.94 ± 0.05	2.87 $E^5 \pm$ 3.39 E^3	1.18 $E^{-3} \pm$ 5.76 E^{-6}	14.12	4.1

SVI. The effects of target compounds for assembly of capsid *in vitro*

Table S6. The time-absorbance curve slopes of the first two minutes of HIV-1 CA *in vitro* assembly assay for representative compounds.

Cpds	Subtype A1		Subtype A2		Subtype B		Subtype C		Subtype D	
	Slope (AU/min)	r^2	Slope (AU/min)	r^2	Slope (AU/min)	r^2	Slope (AU/min)	r^2	Slope (AU/min)	r^2
**IB-2a**	0.028 ± 0.001	0.976	0.016 ± 0.001	0.937	0.008 ± 0.000	0.982	0.036 ± 0.001	0.991	0.033 ± 0.003	0.905
**IC-2a**	0.026 ± 0.001	0.972	0.015 ± 0.000	0.997	0.007 ± 0.000	0.977	0.029 ± 0.001	0.980	0.021 ± 0.002	0.909
**IC-2i**	0.013 ± 0.001	0.917	0.011 ± 0.001	0.962	0.003 ± 0.000	0.900	0.022 ± 0.002	0.934	0.023 ± 0.003	0.865
**IC-2k**	0.012 ± 0.002	0.829	0.005 ± 0.001	0.819	0.003 ± 0.000	0.936	0.019 ± 0.002	0.921	0.016 ± 0.002	0.853
**PF74**	0.107 ± 0.009	0.927	0.062 ± 0.002	0.990	0.074 ± 0.001	0.998	0.101 ± 0.008	0.931	0.088 ± 0.007	0.927
**LEN**	0.015 ± 0.003	0.680	0.009 ± 0.002	0.768	0.006 ± 0.001	0.873	0.029 ± 0.002	0.940	0.022 ± 0.002	0.881
**Protein (control)**	0.005 ± 0.000	0.973	0.003 ± 0.000	0.953	0.001 ± 0.000	0.790	0.018 ± 0.001	0.959	0.015 ± 0.002	0.902

SVII. The effects of target compounds for CANC Disassembly

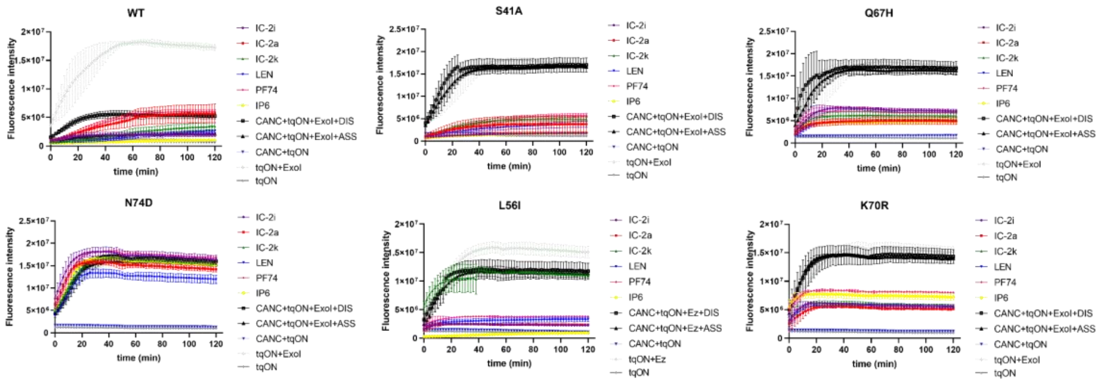

Figure S7. Results of *in vitro* CANC disassembly assays (n=3), ExoI: exonuclease I, ASS: assembly buffer, DIS: disassembly buffer.

SVIII. The effects of target compounds for CANC Assembly

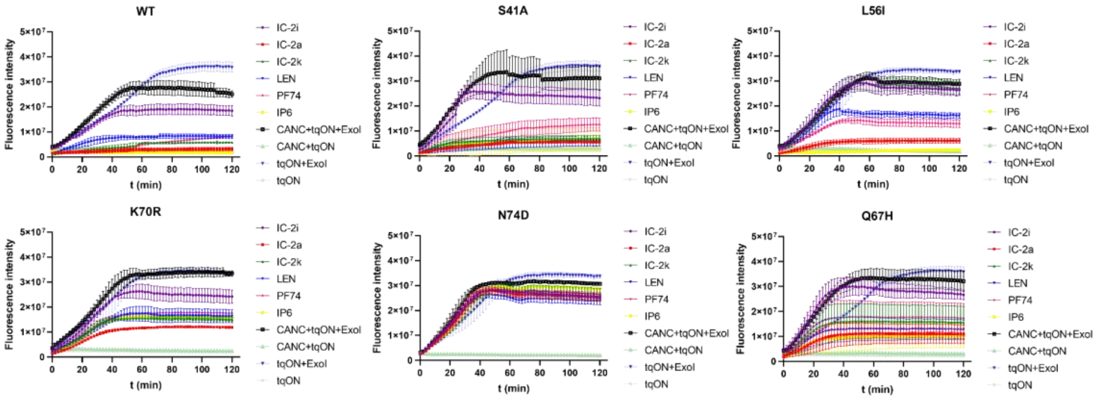

Figure S2. Results of *in vitro* CANC assembly assay (n=3), ExoI: exonuclease I.

SIX. Data collection and refinement statistics in crystallisation research.

Table S7. The Data collection and refinement statistics in crystallisation research

		**HIV-1 Capsid hexamer in complex with IC-2i**
PDBid		**8VRP**
Wavelength (Å)		0.95373
Resolution range (Å)		46.58 - 1.8 (1.84 - 1.8)
Space group		P 6
Unit cell	***a, b, c (Å)***	160.3, 160.3, 57.2
	α, β, γ (°)	90, 90, 120
Total reflections		3298731 (1910734)
Unique reflections		78027 (4429)
Multiplicity		42.3 (43.1)
Completeness (%)		100 (100)
Mean I/sigma(I)		13.0 (1.0)
Wilson B-factor ($Å^2$)		28.2
R-pim		0.049 (2.231)
CC1/2		0.998 (0.441)
Phaser Z-score		13.7
Reflections used in refinement		78010 (7772)
Reflections used for R-free		3904 (391)
R-work		19.2
R-free		22.4
Number of non-hydrogen atoms		5649
macromolecules		5114
ligands		205
solvent		330
Protein residues		663
RMS(bonds)		0.008
RMS(angles)		1.577
Ramachandran favored (%)		98.78
Ramachandran allowed (%)		1.22
Ramachandran outliers (%)		0
Average B-factor		36.28
macromolecules		36.23
ligands		29.81
solvent		41.14

SX. 2D View of Binding mode for IC-2i

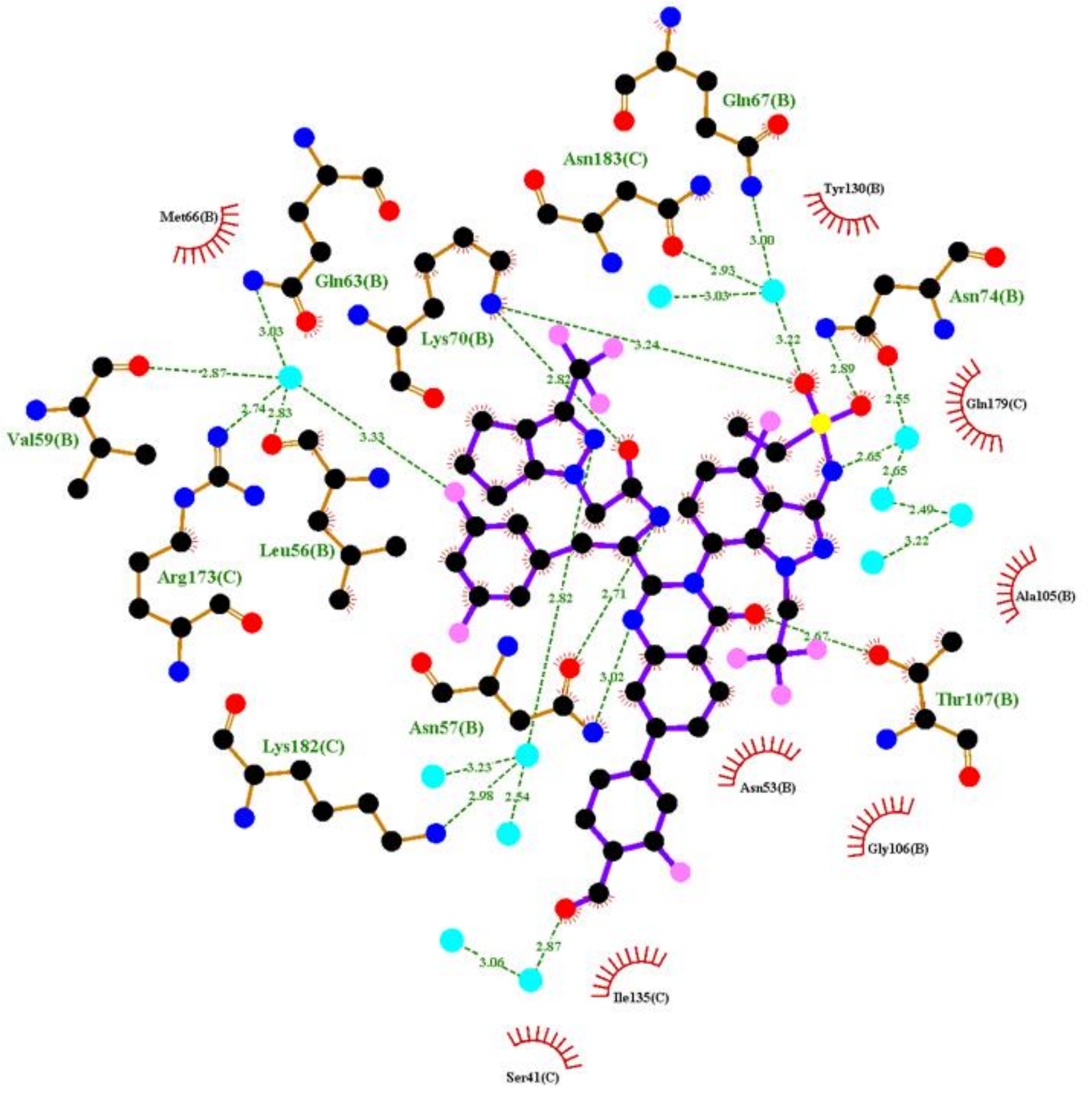

Figure S3. 2D View of Binding mode for IC-2i and distance between residues, water and ligand. Water were shown in light blue round without hydrogen. The figure was generated by LigPlot+[2].

SXII. Results for MD simulation

Table S8. RMSDs and MM/GBSA dG_Bind Energy

Protein	Average Protein RMSD (Å)	Average Ligand RMSD (Å)	Average MMGBSA (kcal/mol)
WT	3.16 ± 0.29	1.18 ± 0.22	-112.661 ± 5.59
K70R	3.80 ± 0.47	1.32 ± 0.24	-121.434 ± 6.91
L56I	3.22 ± 0.51	1.17 ± 0.22	-109.163 ± 7.17
N74D	3.52 ± 0.27	1.21 ± 0.19	-105.469 ± 8.14
Q67H	3.62 ± 0.37	1.18 ± 0.23	-113.195 ± 6.32

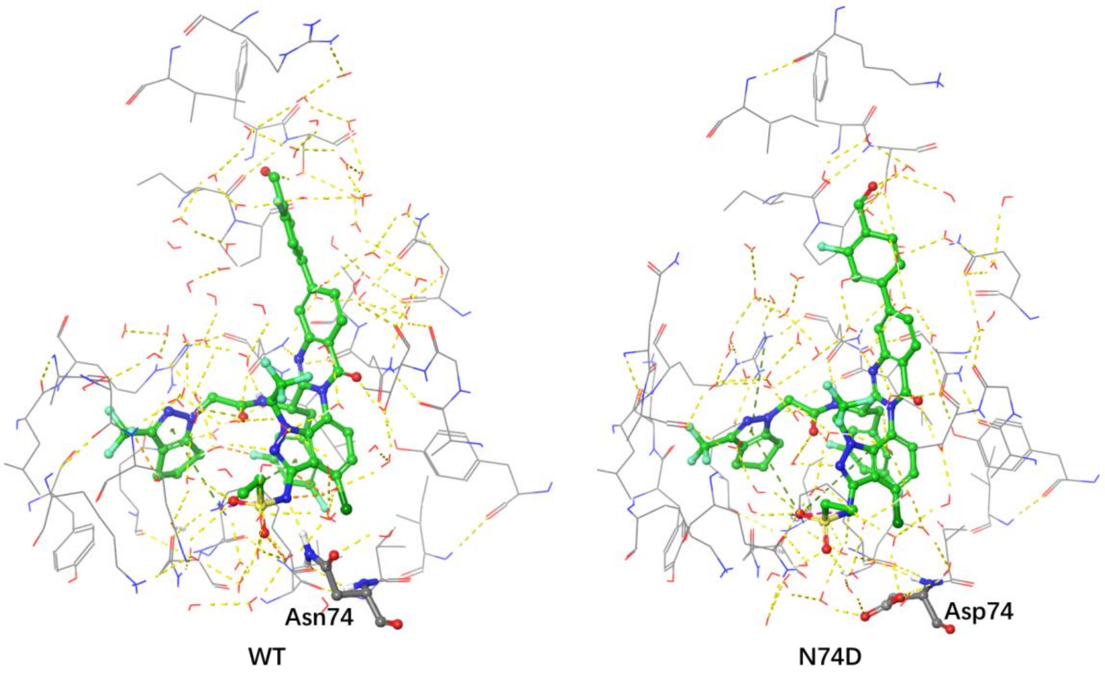

Figure S4. Different binding mode of Asn74 in WT and Asp74 in N74D. Asp74 lost its direct interaction with the sulfone amide and was instead displaced away from the ligand due to charge repulsion.

SXII. Stability in Human Liver Microsomes

1. Procedures

1.1. Test Compound and Control Working Solution Preparation:

1.1.1. Working solution: 5 μL of compound and control stock solution (10 mM in dimethyl sulfoxide (DMSO)) were diluted with 495 μL of acetonitrile (ACN) (intermediate solution concentration: 100 μM, 99% ACN)

1.2. NADPH Cofactor Preparation:

1.2.1. Materials:

NADPH powder: β-Nicotinamide adenine dinucleotide phosphate reduced form, tetrasodium salt; NADPH·4Na (Vendor: BONTAC, Cat. No. BT04)

1.2.2. Preparation Procedure:

The appropriate amount of NADPH powder was weighed and diluted into a 10 mM MgCl2 solution (working solution concentration: 10 mM; final concentration in reaction system: 1 mM)

1.3. Liver Microsomes Preparation:

1.3.1. Materials

Table S9. Liver Microsomes Information

Species	Product Information	Vendor	Abbrevation
Human	Cat No. 452117<br>Lot No. 38297	Corning	HLM

1.3.2. Preparation Procedure:

The appropriate concentrations of microsome working solutions were prepared in 100 mM potassium phosphate buffer

1.4. Stop Solution Preparation:

Cold (4°C) acetonitrile (ACN) containing 200 ng/mL tolbutamide and 200 ng/mL labetalol as internal standards (IS) was used as the stop solution

1.5. Assay Procedure:

1.5.1. Pre-warm empty 'Incubation' plates T60 and NCF60 for 10 min minutes.

1.5.2. Dilute liver microsomes to 0.56 mg/mL in 100 mM phosphate buffer.

1.5.3. Transfer 445 uL microsome working solutions (0.56 mg/mL) into pre-warmed 'Incubation' plates T60 and NCF60, Then pre-incubate 'Incubation' plates T60 and NCF60 for 10 min at 37°C with constant shaking. Transfer 54 µL liver microsomes to blank plate, then add 6 µL NAPDH cofactor to blank plate, and then add 180 µL quenching solution to blank plate.

1.5.4. Add 5 µL compound working solution (100 µM) into 'incubation' plates (T60 and NCF60) containing microsomes and mix 3 times thoroughly.

1.5.5. For the NCF60 plate, add 50 uL of buffer and mix 3 times thoroughly. Start timing; plate will be incubated at 37°C for 60 min while shaking.

1.5.6. In 'Quenching' plate T0, add 180 µL quenching solution and 6 µL NAPDH cofactor. Ensure the plate is chilled to prevent evaporation.

1.5.7. For the T60 plate, mix 3 times thoroughly, and immediately remove 54 µL mixture for the 0-min time point to 'Quenching' plate. Then add 44 µL NAPDH cofactor to incubation plate (T60). Start timing; plate will be incubated at 37°C for 60 min while shaking.

Table S10. Final Concentration of Each Component in Incubation Medium

Component	Concentration
Microsome	0.5 mg protein/mL
Test Compound	1 µM
Control Compound	1 µM
Acetonitrile	0.99%
DMSO	0.01%

1.5.8. At 5, 15, 30, 45, and 60 min, add 180 µL quenching solution to 'Quenching' plates, mix once, and serially transfer 60 µL sample from T60 plate per time point to 'Quenching' plates.

Table S11. Reaction Plates Incubation

Time Point	Start Time	End Time
Blank	1:00:00	0:00:00
T60	1:00:00	0:00:00

T45	0:45:00	0:00:00
T30	0:30:00	0:00:00
T15	0:15:00	0:00:00
T5	0:05:00	0:00:00
T0	mix 3 times and remove out to 'Quenching' plate	

1.5.9. For NCF60: mix once, and transfer 60 µL sample from the NCF60 incubation to 'Quenching' plate containing quenching solution at the 60-min time point.

Table S12. NCF60 Incubation

Time Point	Start Time	End Time
NCF60	1:00:00	0:00:00

1.5.10. All sampling plates are shaken for 10 min, then centrifuged at 4000 rpm for 20 minutes at 4°C.

1.5.11. Transfer 80 µL supernatant into 240 µL HPLC water, and mix by plate shaker for 10 min.

1.5.12. Each bioanalysis plate was sealed and shaken for 10 minutes prior to LC-MS/MS analysis.

2. Data Analysis

The equation of first order kinetics was used to calculate T1/2 and CLint(mic) (µL/min/mg):

Equation of first order kinetics:

$$C_t = C_0 \cdot e^{-k_c \cdot t}$$

$$\text{When } C_t = \frac{1}{2} C_0$$

$$T_{1/2} = \frac{Ln2}{k_e} = \frac{0.963}{k_e}$$

$$CL_{int(mic)} = \frac{0.963}{in\ vivo\ T_{1/2}} \cdot \frac{1}{mg/ml\ mirosomal\ protein\ in\ reaction\ system}$$

$$CL_{int(liver)} = CL_{int(mic)} \cdot \frac{mg\ microsomes}{g\ liver} \cdot \frac{g\ liver}{kg\ body\ weight}$$

3. Results Summary

Table S13. Stability of representative compounds in human liver microsomes

**Cpds**	**R^2**	**$T_{1/2}$ (min)**	**$CL_{int(mic)}$ (mL/min/kg)**	**$CL_{int(liver)}$ (mL/min/kg)**	**Remaining (T=60min)**	**Remaining (*NCF=60min)**
**IC-2a**	0.9306	14.0	99.3	89.4	4.3%	107.7%
**IC-2i**	0.9439	13.8	100.2	90.2	4.5%	78.4%
**LEN**	0.8398	>145	<9.6	<8.6	73.2%	97.1%
**PF74**	1.0000	0.6	2314.9	2083.4	0.0%	96.6%
**Testosterone**	0.8926	13.3	104.0	93.6	4.1%	88.5%
**Diclofenac**	0.9988	6.2	222.3	200.0	0.1%	94.5%
**Propafenone**	0.9073	8.5	163.8	147.4	0.5%	99.0%

NCF: abbreviation of no co-factor. No NADPH is added to NCF samples (replaced by buffer) during the 60 minute incubation. If the NCF remaining is less than 60%, then possibly non-NADPH dependent metabolism occurs;

R^2: correlation coefficient of the linear regression for the determination of kinetic constant (see raw data worksheet);

$T_{1/2}$: half life;

$CL_{int(mic)}$: intrinsic clearance;

$CL_{int(mic)}$ = 0.693/$T_{1/2}$/mg microsome protein per mL;

$CL_{int(liver)}$ = CLint(mic) * mg microsomal protein/g liver weight * g liver weight/kg body weight.

SXIII. Stability in Human Plasma

1.Materials

1.1 Test Compounds and Stock Solutions

Table S14. Test Compounds and Stock Solutions

Compound ID	Batch	MW	FW	Conc. (mM)	Final Conc.(μM)
**PF74**	PF74	425.53	425.53	10	2
**LEN**	LEN	968.28	968.28	10	2
**IC-2i**	IC-2i	979.28	979.28	10	2
**IC-2a**	IC-2a	936.07	936.07	10	2
**Propantheline bromide**	140419	448.39	448.39	10	2

1.2. Test Compound and Control Working Solution Preparation

Test compound: 5 μL of compound stock solution (10 mM in dimethyl sulfoxide (DMSO)) were diluted with 495 μL of DMSO (Working solution concentration: 100 uM, 100% DMSO).

Propantheline bromide Working solution: 5 μL of Propantheline bromide stock solution (10 mM in H_2O) were diluted with 495 μL of H_2O (Working solution concentration: 100 uM, 100% H_2O).

1.3 Test System

Species / Matrix	Minimum No. of Individuals	Anticoagulant Used	Vendor	Cat#	Batch
Human Plasma	3 Male & 3 Female	EDTA-K2	BioreclamationIVT	HUMANPLK2P2N	HMN761043

2.Methods

2.1. The pooled frozen plasma was thawed in a water bath at 37°C prior to experiment. Plasma was centrifuged at 4000 rpm for 5 min and the clots were removed if any. The pH will be adjusted to 7.4 ± 0.1 if required.

2.2. Using an Apricot automation workstation, 98 μL/well of blank plsma were added to all 96-well reaction plates. (Blank, T0, T10, T30, T60 and T120)

2.3. An Apricot automation workstation was used to add 2 µL/well of working solution (100 µM) to all reaction plates except Blank. (T0, T10, T30, T60 and T120)

2.4. All reaction plates containing mixtures of compound and plasma were incubated at 37°C in water bath.

2.5. The reaction plates were incubated at 37°C, and timer was started.

Table S15. Reaction Plates Incubation

Time Point	Start Time	End Time
Blank	0:00:00	0:00:00
T120	2:00:00	0:00:00
T60	1:00:00	0:00:00
T30	0:30:00	0:00:00
T10	0:10:00	0:00:00
T0		

2.6 At the end of incubation, added 500 µL of stop solution (200 ng/mL tolbutamide and 200 ng/mL labetalol in ACN) to precipitate protein. Mixed thoroughly.

2.7. Each plate was sealed and shaken for 20 minutes.

2.8. After shaking, each plate was centrifuged at 4000 rpm and 4°C for 20 minutes.

2.9. After centrifugation, an Apricot automation workstation was used to transfer 150 µL of supernatant from each reaction plate to its corresponding bioanaylsis plate.

2.10. Each bioanalysis plate was sealed and shaken for 10 minutes prior to LC-MS/MS analysis.

3. Data Analysis

The % remaining of test compound after incubation in plasma was calculated using following equation:

% Remaining= 100 x (PAR at appointed incubation time / PAR at T0 time)

where PAR is the peak area ratio of analyte versus internal standard (IS)

The appointed incubation time points are T0 (0 min), Tn (n=0, 10, 30, 60, 120min)

The half-life ($T_{1/2}$) was calculated from a log linear plot of concentration versus time.

$$C_t = C_0 \cdot e^{-k_c \cdot t} \qquad lnC_t = lnC_0 - k \cdot e \cdot t$$

$$\text{When } C_t = \frac{1}{2}C_0, T_{1/2} = \frac{Ln2}{k_e} = \frac{0.963}{k_e}$$

3. Results Summary

Table S16. Human plasma metabolism data for representative compounds

Cpds	$T_{1/2}$ (min)
**IC-2a**	186.2
**IC-2i**	>289.1[a]
**PF74**	>289.1
**LEN**	140.2
**Propantheline bromide**	9.3

[a] When the %remaining value at the maximal incubation time, which was 120 min in this study, was higher than 75%, it is considered to be within the acceptable experimental variation. Therefore, a corresponding $T_{1/2}$ of > 289.1 min was reported.

SXIV. *In Vivo* Pharmacokinetics Study

Table S17. Pharmacokinetic parameters of **IC-2i** in rats

Parameter	Units	i.v.	p.o.	s.c.
$T_{1/2}$	h	2.37 ± 0.448	1.60 ± 0.47	19.9 ± 14.2
Tmax	h	0.0833 ± 0.00	2.67 ± 1.15	1.33 ± 0.577
Cmax	ng/mL	2077 ± 323	37.7 ± 11.0	45.9 ± 14.8
C0	ng/mL	4255 ± 523	-	-
$AUC_{0\text{-}t}$	h*ng/mL	744 ± 141	146 ± 8.60	700 ± 178
$AUC_{0\text{-}\infty}$	h*ng/mL	759 ± 139	159 ± 14.7	1171 ± 308
Vz	mL/kg	9477 ± 3703	-	-
Cl	mL/h/kg	2704 ± 555	-	-
$MRT_{0\text{-}t}$	h	0.609 ± 0.0557	3.57 ± 0.300	9.20 ± 1.45
$MRT_{0\text{-}\infty}$	h	0.825 ± 0.0582	4.16 ± 0.203	28.2 ± 21.0
F	%	-	4.20 ± 0.388	61.73 ± 16.1

References

1. Shafer RW. Rationale and uses of a public HIV drug-resistance database. *J Infect Dis*. 2006;194 Suppl 1(Suppl 1):S51-8.

2. Laskowski RA, Swindells MB. LigPlot+: multiple ligand-protein interaction diagrams for drug discovery. *J Chem Inf Model*. 2011;51(10):2778-86.
